# Exploration of C–H and N–H-bond functionalization towards 1-(1,2-diarylindol-3-yl)tetrahydroisoquinolines

**DOI:** 10.3762/bjoc.10.226

**Published:** 2014-09-15

**Authors:** Michael Ghobrial, Marko D Mihovilovic, Michael Schnürch

**Affiliations:** 1 Institute of Applied Synthetic Chemistry, Vienna University of Technology, 1060 Vienna, Austria

**Keywords:** Buchwald–Hartwig coupling, C–C coupling, C–H functionalization, iron catalysis, regioselective arylation

## Abstract

The synthesis of 1,2,3-trisubstituted indoles was investigated. More specifically, straightforward synthetic routes towards 1-(1,2-diarylindol-3-yl)-*N*-PG-THIQs (PG = protecting group, THIQ = tetrahydroisoquinoline) employing transition metal-catalyzed C–H and N–H-bond functionalization were explored. It was found that the synthesis of the target compounds is strongly dependent on the order of events. Hence, depending on the requirements of a synthetic problem the most suitable and promising pathway can be chosen. Additionally, a new synthetic approach towards 1,2-diarylindoles starting from 1-arylindole could be established in the course of our investigation by using a palladium-catalyzed protocol. Such 1,2-diarylindoles were successfully reacted with *N*-Boc-THIQ to furnish 1,2,3-trisubstituted indoles as target compounds. Furthermore, regioselective N-arylation of protected and unprotected 1-(indol-3-yl)-THIQs was successfully conducted using either simple iron or copper salts as catalysts.

## Introduction

**Figure 1:**
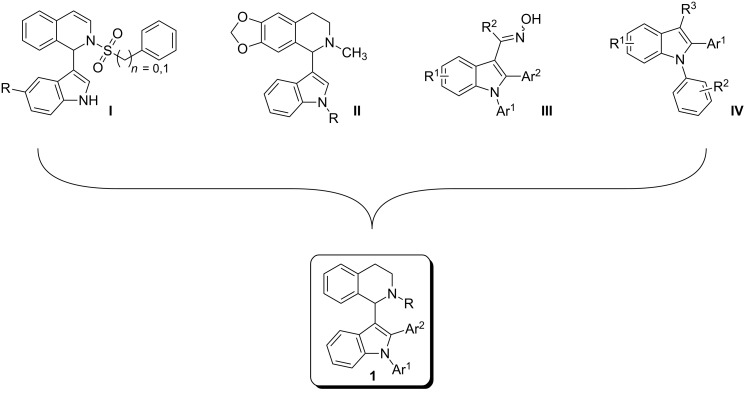
General structures of biologically active dihydroisoquinolines, THIQs and 1,2-diarylindoles.

1,2,3,4-Tetrahydroisoquinolines (THIQs) are common substructures in natural products [1]. The structural motif of 1-(indol-3-yl)-THIQ is also found in compounds with biological activity, for example activity against cancer cells by inhibition of Rad51 – a protein which interacts with the tumor suppressor BRCA2 (Figure 1, 1-(indol-3-yl)dihydroisoquinoline **I**) [2]. Furthermore, 1-(indol-3-yl)-THIQs are also investigated as agents against neurodegenerative diseases (Figure 1, **II**) [3]. Moreover, the indole moiety is considered as a privileged structure since it is encountered in many bioactive molecules and arylindoles [4] are particularly active when substituted in 2-position [5]. 1,2-Diarylindoles have been reported to display interesting pharmacological activities, e.g., as estrogen receptor ligands (Figure 1, **III**) [6], having potential in the treatment of Alzheimer's disease (Figure 1, **IV**) [7], or in treatment of diseases associated with defects in vesicular (axonal) transport (Figure 1, **IV**) [8].

Interestingly, 1-(indol-3-yl)-THIQs carrying additional aryl substituents on the indole ring have not been reported as bioactive molecules so far. Actually, this compound class is underrepresented in literature with only three examples of 1-(1-arylindol-3-yl)-THIQs being reported [9–10]. No 1-(2-arylindol-3-yl)-THIQs or 1-(1,2-diarylindol-3-yl)-THIQs **1** have been disclosed in literature up to date. The latter compound class can be considered as a combination of structural features of general structures **I**–**IV** which would lead to **1** (Figure 1), the target compounds of the present contribution. It was our aim to develop a facile synthetic route towards compounds **1** due to their prospect to display certain pharmaceutical properties.

**Scheme 1:**
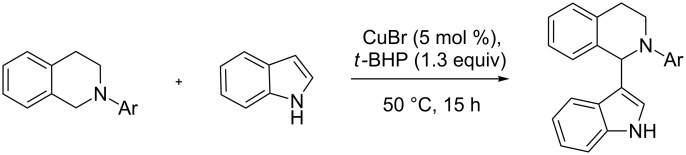
Li’s THIQ indolation protocol.

Several synthetic methods for the preparation of 1-(indol-3-yl)-THIQs have been reported: In the year 2004, Venkov et al. described a metal-free procedure for the formation of 1-(indol-3-yl)-THIQs by addition of indole to a dihydroisoquinoline iminium salt [11]. This synthesis has been streamlined by cross dehydrogenative coupling (CDC) – a powerful method for C–C-bond formation via the C–H bonds of a pro-nucleophile and a pro-electrophile [12–14]. A landmark contribution published by Li and co-workers reported the successful introduction of functionalized indoles into position 1 of N-arylated THIQ in presence of *tert*-butylhydroperoxide (*t*-BHP) using copper(I) bromide as catalyst (Scheme 1) [15]. Inspired by this report the method has been expanded by others in terms of oxidant, catalyst and substrate scope [16–21]. In our group we could address one important shortcoming of the THIQ indolation: the requirement for an N-phenyl group on THIQ. We could demonstrate that the indolation can also be carried out with the easily removable Boc group and even in absence of any protecting group [22–23].

The synthesis of arylated indoles is well established via a series of transformations. The direct arylation via C–H functionalization represents a very efficient approach in this regard [24–31] with methods available to either decorate position 2 or 3 regioselectively [32–45]. However, these methods have not been applied on complex molecules leading to sterically crowded compounds. Additionally, N-arylation of indole is well established as well [25,46–49].

Within this contribution we report a comprehensive investigation of strategies to prepare 1-(1,2-diarylindol-3-yl)-*N*-protected-THIQs **1** by employing palladium, copper, and iron-catalyzed methods.

## Results and Discussion

**Scheme 2:**
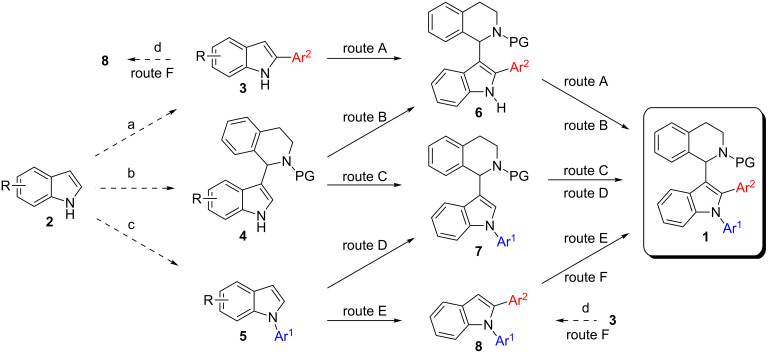
Possible strategies for the synthesis of target structure **1**. Dashed arrows indicate literature-known pathways consequently not investigated in this contribution. a: see [32–45]; b: see [15–23]; c: see [46–49]; d: see [50–51].

Catalyst price and availability were important considerations for selecting transformations to be used in this project. Whenever possible, iron or copper catalysis was envisioned since these two metals are most desirable out of economic and environmental reasons. However, in cases where these metals cannot give satisfactory results, palladium should be used to solve the problem. Analyzing the target compound class, several synthetic routes can be drafted. As a premise, we wanted to use direct functionalization either via C–H activation or cross dehydrogenative coupling for C–C-bond-forming reactions avoiding the use of two prefunctionalized building blocks. Naturally, C–N-bond formation should proceed via Buchwald–Hartwig coupling. The target molecules can be considered as 1,2,3-trisubstituted indoles and we decided to synthesize them starting from (substituted) indole **2**. Hence, six synthetic routes are proposed which differ only in the order of bond-forming events. Considering the location of bond formations at indole, the positions can be decorated in the following orders: 1-2-3 (route E), 1-3-2 (route D), 2-1-3 (route F), 2-3-1 (route A), 3-1-2 (route C), and 3-2-1 (route B) (Scheme 2). It was our aim to investigate all of these routes in order to assess efficiency towards key intermediates and target structures. Initially pathways towards intermediates **6**–**8** will be presented before the final step from these compounds towards target structure **1** will be discussed.

### Pathways towards intermediates **6–8**

#### Synthesis of intermediate **6** through routes A and B

Various reaction conditions have been reported to carry out N-arylation [46–49] and direct C2-arylation of [32–45] indole. Additionally, we and others have reported the CDC reaction between C3 of indole and C1 of THIQ with either a permanent, a removable, or without a substituent on the THIQ nitrogen [15–23]. Hence, intermediates **3**–**5** are all available via established routes and their synthesis was not investigated within this contribution. Additionally, starting from 2-arylindoles **3**, formation of 1,2-diarylindoles **8** is well known [50–51] as well and was not investigated herein. Consequently, our research started with further converting these intermediates to the target products.

The introduction of THIQ in position 3 of 2-arylated indoles **3** has not been disclosed previously (Scheme 2, first step of route A). Li and coworkers reported that 2-methylindole did give the indolated THIQ in 61% isolated yield [15] which indicates that some steric bulk is tolerated in this position. We set out to test whether aryl substituents in position 2 would be tolerated as well. As model reaction 2-phenylindole (**3a**) was reacted with *N*-Boc-THIQ **9** and either Fe(NO_3_)_3_·9H_2_O or Cu(NO_3_)_2_·3H_2_O were applied as catalysts as reported in our previously disclosed indolation protocol (Table 1) [23].

**Table 1:** Reaction scope for reactions of indoles **3a**–**d** with *N*-Boc-THIQ **9**.

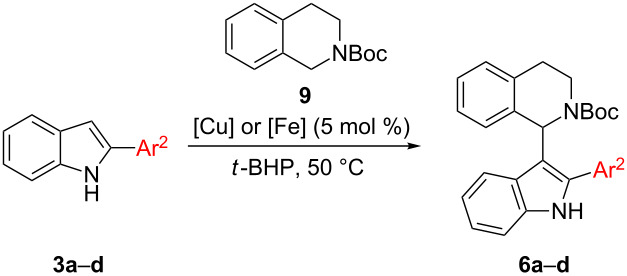				

Entry	Products	Ar^2^	Yield [%]	
			Cu(NO_3_)_2_	Fe(NO_3_)_3_

1	**6a**	C_6_H_5_	44	56
2	**6b**	4-Me-C_6_H_4_	n.a.^a^	20
3	**6c**	4-MeO-C_6_H_4_	n.a.^a^	14
4	**6d**	3-NO_2_-C_6_H_4_	n.a.^a^	dec.^b^

^a^n.a.: not attempted; ^b^dec.: decomposition.

Unfortunately, the transformation proved to be of limited success independent on the metal salt applied. In case of 2-phenylindole (**3a**) a reasonable yield of 56% of the desired product **6a** was obtained employing iron(III) nitrate as the catalyst. In case of copper, the yield decreased to 44% (Table 1, entry 1). Thus, further reactions were carried out using the iron catalyst. Placing an electron-withdrawing nitro group on the phenyl ring afforded only decomposition products (Table 1, entry 4). The 4-tolyl- and 4-methoxyphenyl-substituted products **6b** and **6c** were obtained in poor yields of 20 and 14%, respectively (Table 1, entries 2 and 3). Hence, route A suffers from a low yield already in the first step. Since the aryl substituent in position 2 exhibits significant steric hindrance, this can account for the low efficiency of this transformation. It has to be mentioned that compounds of type **6** have not been disclosed in literature so far, and even though yields are poor and the substrate scope is limited, three examples of a new compound class **6a**–**c** were made accessible.

An alternative way to get to structures **6** is by inverting the order of bond-forming events, i.e., starting with the introduction of THIQ in position 3 and subsequent C2-arylation of the indole core (i.e., Scheme 2, route B). We and others have previously reported the synthesis of 1-(indol-3-yl)-*N*-PG-THIQs **4** [15,22–23], so we could start to investigate C2-arylation immediately.

Although a broad range of (hetero)arenes undergo C–H-arylation under copper catalysis, heterocycles possessing acidic N–H bonds react at the nitrogen preferentially [52–53]. Moreover, directing groups such as acetyl (in combination with a hypervalent iodine aryl source) [42], or 2-pyridinyl attached to the nitrogen of the indole, are required to facilitate copper-catalyzed C2-arylations [54]. We intended to avoid a directing group on the indole since this would require two additional reaction steps to install and cleave such a group. Due to these limitations, palladium was considered as catalyst instead of copper since palladium has been widely recognized as powerful transition metal catalyst involved in C2-arylations of azoles [25,33,46–47,55–58]. C2-Arylation of 1-(indol-3-yl)-*N*-PG-THIQs **4** was expected to be challenging since the C3 position of the indole is blocked by the bulky THIQ residue. The group of Sames reported a ligand-free palladium-catalyzed protocol for C2-arylation of 3-substituted indoles [35]. However, this method did not give any conversion to C2-arylated products on our substrates. Yang et al. reported a mild palladium-catalyzed C2-arylation at room temperature under acidic conditions, employing boronic acids as aryl source [59]. Using this method the desired product **6a** could be obtained but only in a low yield of 36% (Table 2, entry 1). Besides **6a** also 12% of biphenyl could be isolated. Increasing the temperature to 50 °C proved to be counterproductive, as decomposition was observed due to partial cleavage of the Boc group. Addition of 1 equiv of copper(II) acetate facilitated the reaction and 51% of the desired product **6a** were obtained (Table 2, entry 1). Further screening of reaction parameters (e.g., prolonged reaction time and other acids such as TFA) did not lead to an improvement in yield. It was also observed that the water content in the reaction had a significant influence. By thoroughly drying the AcOH (distillation under argon atmosphere and addition of grinded molecular sieves to the reaction mixture) considerably slowed down the reaction. Formation of trimeric boronic acid anhydride was observed via GC–MS, a species less reactive in arylation reactions. When small amounts of water were added on purpose the reaction solution turned black immediately, most likely due to the formation of Pd black which is inactive in the present transformation. Obviously the steric bulk of the THIQ substituent in 3-position makes this transformation quite difficult. Hence it was decided to stick with the so far best conditions to investigate the scope of C2-arylation, the first step of route B (Scheme 2).

**Table 2:** Scope of palladium-catalyzed, regioselective C2-arylation of 1-(indol-3-yl)-*N*-PG-THIQ **4**.

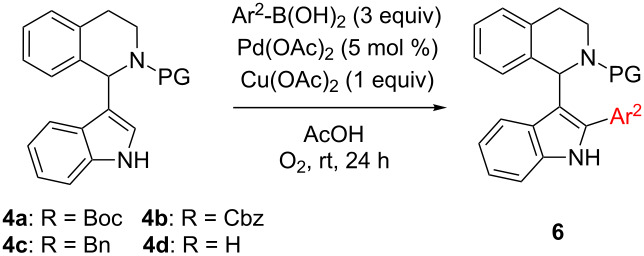				

Entry	Products	Ar^2^	PG	Yield [%]

1	**6a**	C_6_H_5_	Boc	51/36^a^
2	**6b**	4-Me-C_6_H_4_	Boc	49
3	**6c**	4-MeO-C_6_H_4_	Boc	34
4	**6e**	4-NO_2_-C_6_H_4_	Boc	7^b^
5	**6f**	1-naphthyl	Boc	14^b^
6	**6g**	C_6_H_5_	Cbz	51
7	**6h**	C_6_H_5_	Bn	n.c.^c^
8	**6i**	C_6_H_5_	H	traces

^a^Without Cu(OAc)_2_; ^b^conversion according to GC–MS, not isolated; ^c^no conversion.

Using substituted boronic acids led to decreased yields in most cases. Only 4-tolylboronic acid furnished the desired product **6b** in the same yield range as compared to phenylboronic acid (49%, Table 2, entry 2). The yield dropped to 34% when employing a +M-substituent such as 4-methoxy (Table 2, entry 3). C2-arylation in presence of a strongly electron-withdrawing group such as 4-nitrophenyl afforded a poor yield of 7% (**6e**, Table 2, entry 4). The sterically demanding 1-naphthyl group furnished product **6f** in unsatisfactory 14% yield. Using phenylboronic acid the yield was the same no matter whether Boc or Cbz were used as protecting group (Table 2, entries 1 and 6). A carbonyl protective group on the nitrogen of THIQ proved to be crucial, as only traces of the correct *m*/*z* for desired product **6i** were observed (GC–MS) in case of unprotected THIQ **4d** (Table 2, entry 8) and also benzyl-protected **4c** showed no reaction (**6h**, Table 2, entry 7).

#### Pathways to intermediate **7** through routes C and D

As it was the case for the synthesis of **6**, compounds of type **4** can be used as starting material for the synthesis of intermediates **7** (Scheme 2, first step in route C). In this case we expected N-arylation to proceed favorably under iron catalysis due to a largely relieved steric situation. N-Arylation of **4** was initially attempted under the iron-catalyzed N-arylation conditions reported by Bolm [60].

Gratifyingly, the reaction of **4a** with iodobenzene afforded product **7a** in a good yield of 72% (86% based on re-isolated **4a**) after flash chromatography (Table 3, entry 1). Product **7g** was obtained in slightly better yield when replacing the Boc-PG with Cbz (Table 3, entry 7, 77%). Since both protective groups performed well, it was decided to use the Boc-PG for further investigations out of two reasons: i) the Boc group proved to be most efficient for iron-catalyzed indolation of *N*-PG-THIQ; ii) a facile protocol for deprotection of the Boc group using TMSCl/MeOH was already established within a previous study [22]. Hence, the scope of arylation was investigated on 1-(indol-3-yl)-*N*-Boc-THIQ **4a**. The best yield was obtained when employing electron-rich 2-iodothiophene yielding **7c** in 85% (Table 3, entry 3). 1-Fluoro-4-iodobenzene showed a similar result (84% of **7d**), indicating that a –I-substituent is well accepted when employing an iron catalyst for N-arylation (Table 3, entry 4). A reasonable yield of 68% was obtained when performing the arylation with 4-iodoanisole (Table 3, entry 2), which is in the same range as for the phenyl-substituted product. Placing a nitro group in position 4 of the aryl motif significantly reduced the yield (Table 3, entry 5, 50% of **7e**).

**Table 3:** Scope of iron-catalyzed N-arylation of **4a,b,d**.

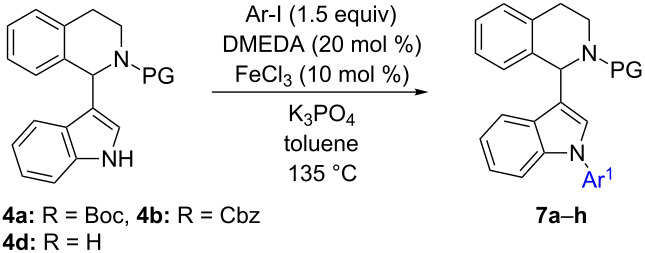				

Entry	Products	Ar^1^	PG	Yield [%]^a^

1	**7a**	C_6_H_5_	Boc	72 (86)
2	**7b**	4-MeO-C_6_H_4_	Boc	68 (84)
3	**7c**	2-thienyl	Boc	85
4	**7d**	4-F-C_6_H_4_	Boc	84
5	**7e**	4-NO_2_-C_6_H_4_	Boc	50 (74)
6	**7f**	2-fluoropyridin-3-yl	Boc	n.c.
7	**7g**	C_6_H_5_	Cbz	77
8	**7h**	C_6_H_5_	H	dec.

^a^Yields in parentheses are based on reisolated starting material.

**Scheme 3:**
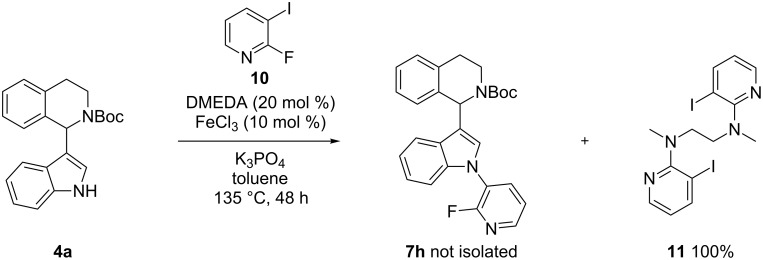
Nucleophilic substitution of DMEDA with 2-fluoro-3-iodopyridine (**10**).

Electron-poor heterocycles with additional steric hindrance such as 2-fluoro-3-iodopyridine (**10**) were ineffective under these conditions. Instead, nucleophilic substitution by the *N*,*N*'-dimethylethylenediamine (DMEDA) ligand took place, affording product **11** quantitatively (Scheme 3).

**Scheme 4:**
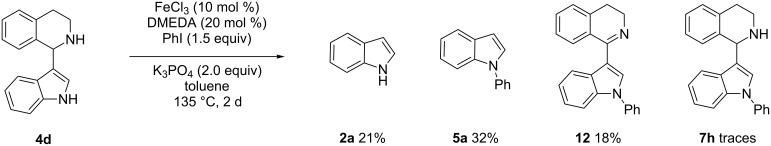
Decomposition of 1-(indol-3-yl)-THIQ **4d** during N-arylation (monitored by GC–MS).

Depending on the synthetic problem indole N-arylation of unprotected THIQ can be a desirable transformation as well and thus was investigated further. However, attempts to N-arylate **4d** did not give a clean transformation (Table 3, entry 8) but ended up in decomposition products observed by TLC and detected by GC–MS (Scheme 4).

Similar results were obtained when replacing the iron catalyst with copper(I) iodide, keeping the other parameters constant. In this reaction, 1-phenylindole (**5a**) was isolated as major product in 45% yield. These results suggest that in these reactions the temperature was too high, resulting in decomposition of starting material **4d** and product **7h**. This was confirmed by heating **4d** in isooctane (bp: 99 °C) leading to decomposition as well. Thus, an alternative N-arylation method had to be established, employing milder conditions, especially at lower reaction temperature. Phillips et al. demonstrated that N-arylation can be carried out using CsF instead of strong alkali metal bases at 60 °C employing various heterocyclic substrates including indole [61]. Inspired by this report, reaction parameters were changed to 2.5 equiv CsF instead of K_3_PO_4_ and acetonitrile instead of toluene. Most importantly 70 °C were applied instead of 135 °C using **4d** as substrate (Table 4).

**Table 4:** CsF-mediated, copper-catalyzed regioselective N-arylation.

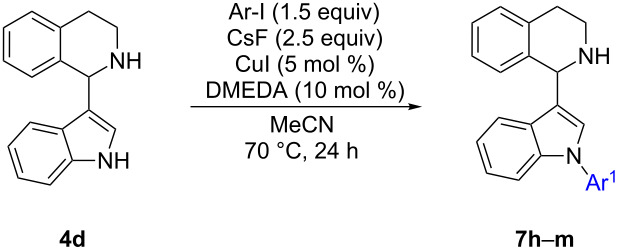			

Entry	Products	Ar^1^	Yield [%]

1	**7h**	C_6_H_5_	73/62^a^/n.c^b^
2	**7i**	4-MeO-C_6_H_4_	52^c^
3	**7j**	2-thienyl	79
4	**7k**	4-F-C_6_H_4_	58
5	**7l**	3-NO_2_-C_6_H_4_	74
6	**7m**	4-COOEt-C_6_H_4_	63

^a^100 °C, 2 h; ^b^without CsF; ^c^11% elimination byproduct isolated.

In a first attempt using iodobenzene as aryl donor, the lowered reaction temperature (70 °C) resulted in product formation with minimal decomposition according to TLC and GC–MS and a good yield of **7h** (73%) was obtained (Table 4, entry 1). ^1^H NMR and ^13^C NMR confirmed that arylation occurred regioselectively at the nitrogen of the indole moiety. Carrying out the reaction in the absence of cesium fluoride resulted in no conversion. At 100 °C the reaction time could be reduced to 2 hours, however, the yield dropped to 62% due to increased decomposition. Hence, the reaction scope was investigated at 70 °C. Electron-rich 2-iodothiophene afforded a good yield of 79% of **7j** (Table 4, entry 3). This time, 4-iodoanisole was not as effective opposed to the N-arylation of **4b**, but still gave a satisfactory yield of 52% of **7i** (Table 4, entry 2). Besides **7i**, also 11% of 1-[(4-methoxyphenyl)-indol-3-yl]-DHIQ were isolated as byproduct, which originates from oxidation of the corresponding THIQ. An ester functionality was well tolerated, furnishing 63% of product **7m** (Table 4, entry 6) and also 4-fluorophenyl and 3-nitrophenyl groups did not hamper the reaction (Table 4, entries 4 and 5). Arylation of the nitrogen of the THIQ core was not observed in any case.

Alternatively, intermediates **7** can be formed by N-arylindolation of *N*-PG-THIQ **9** (Scheme 2, first step of route D, Table 5). Thus, in the beginning, indole was N-arylated according to literature conditions giving substrates **5a–e** [62–63]. The obtained intermediates were then reacted with *N*-Boc-THIQ **9**. Gratifyingly, in both cases our previously established iron- or copper-catalyzed indolation protocols [22–23] afforded the corresponding products **7**. Hence, N-substituted indoles **5** are efficient substrates in this transformation. Still, a significant difference in yields was observed when applying copper or iron as the catalyst (Table 5).

**Table 5:** Scope of the reaction of N-arylindoles **5a–e** with *N*-Boc-THIQ **9**.

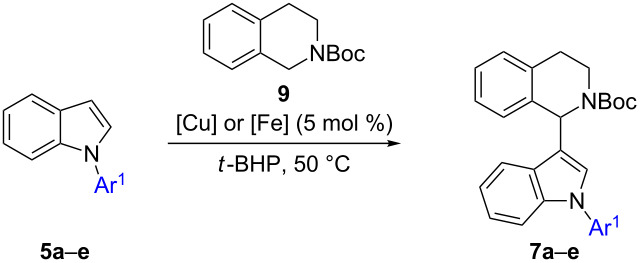				

Entry	Products	Ar^1^	Yield [%]	
			Cu(NO_3_)_2_	Fe(NO_3_)_3_

1	**7a**	C_6_H_5_	83	49
2	**7b**	4-MeO-C_6_H_4_	69	40
3	**7c**	2-thienyl	78	n.a.^a^
4	**7d**	4-F-C_6_H_4_	65	n.a.^a^
5	**7e**	4-NO_2_-C_6_H_4_	45^b^	n.a.^a^

^a^n.a: not attempted; ^b^2.6 equiv *t*-BHP, 10 mol % [Cu], 80 °C, 2 d.

When performing the reaction under copper(II) nitrate catalysis, a good yield of 83% of **7a** was obtained when 1-phenylindole (**5a**) was used as N-arylindole coupling partner (Table 5, entry 1). On the other hand, a significant decrease in product yield was observed when employing iron(III) nitrate as the catalyst (Table 5, entry 1, 49%). The same trend in catalyst activity was confirmed when switching to 1-(4-methoxyphenyl)indole (**5b**) as substrate furnishing the corresponding product **7b** in 69% (Cu, Table 5, entry 2) and 40% (Fe, Table 5, entry 2). Obviously, copper is superior to iron in this particular transformation. Thus, the remaining N-arylindoles **5c**–**e** were reacted under copper catalysis exclusively. Again, the electron-rich thiophen-2-yl-substituted substrate gave **7c** in a good yield of 78% (Table 5, entry 3). Also the 4-fluorophenyl precursor **5d** performed well, furnishing the desired product **7d** in 65% yield (Table 5, entry 4).

**Scheme 5:**
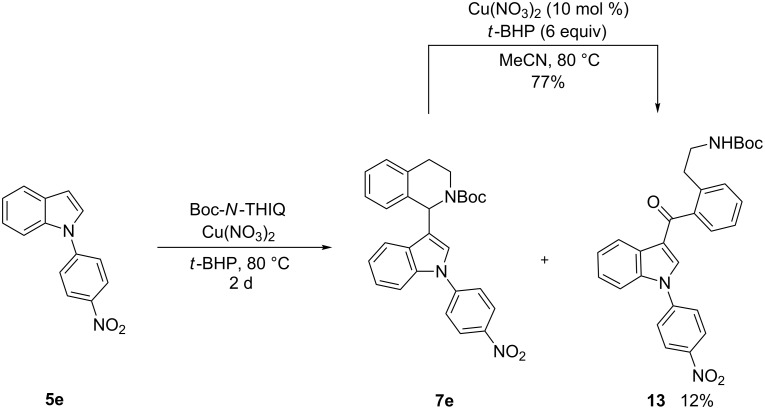
Formation of byproduct **13** via benzylic oxidation.

In case of the 4-nitrophenyl group as in **5e**, which reflects a −M/−I-substituent, no conversion was observed under standard indolation conditions (5 mol % Cu(NO_3_)_2_·3H_2_O, 1.3 equiv *t*-BHP, 50 °C, 15 h). Hence, another batch (1.3 equiv) of *t*-BHP and Cu(NO_3_)_2_·3H_2_O (5 mol %) was added and the temperature was increased to 80 °C. Gratifyingly, the desired product **7e** was obtained, however a lower yield of 45% was achieved (Table 5, entry 5). Besides the desired product **7e**, also a byproduct could be isolated and identified as benzylic oxidized product **13** (Scheme 5). This compound is formed from **7e** since submitting pure **7e** to the reaction conditions led to formation of **13** in 77% yield, explaining the decreased yield for **5e** (Table 5, entry 5).

The question remained whether copper is required for the formation of **13**. Thus, **7e** was reacted in presence and in absence of a copper catalyst. Interestingly, the byproduct **13** was formed in 83% conversion (GC–MS) without adding a catalyst to the reaction under neat conditions.

#### Pathways to intermediate **8** for routes E and F

The third potential substrates for formation of target structure **1** are 1,2-diarylindoles **8** which can be used for indolation of PG-*N*-THIQs. Basically, there are two routes towards the synthesis of 1,2-diarylindoles **8**: Either through N-arylation of 2-arylindoles **3** (Scheme 2, first step of route F), which is the more common approach [50–51], or by regioselective C2-arylation of 1-arylindoles **5** (Scheme 2, first step of route E). The latter strategy is rare in literature [64–65]. Two examples towards 1,2-diarylindoles **8** were initially reported by the group of Sames using a palladium-catalyzed protocol employing phenyl iodide [64]. However, this C2-arylation protocol requires phosphine ligands and high reaction temperatures. Thus, we considered the C2-arylation conditions reported by Yang et al. to be the better choice since mild conditions can be applied and only oxygen is required as oxidant (Table 6) [59]. Additionally, Pd(OAc)_2_ is a readily available and relatively inexpensive palladium catalyst. Even though these conditions were not tested on 1-arylindoles such as **5** we expected good results from this procedure and these results are summarized in Table 6.

**Table 6:** Scope of regioselective C2-arylation of 1-arylindoles **5**.

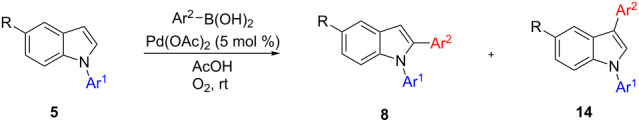							

Entry	Products	R	Ar^1^	Ar^2^	*t*	**8**:**14**^a^	Yield [%]

1	**8a**	H	C_6_H_5_	C_6_H_5_	24 h	**8a** only	96
2	**8b**	H	C_6_H_5_	4-Cl-C_6_H_4_	2 d	91:1	11
3	**8c**	H	C_6_H_5_	4-MeO-C_6_H_4_	24 h	48:1	94
4	**8d**	H	C_6_H_5_	4-Me-C_6_H_4_	24 h	38:1	87
5	**8e**	H	C_6_H_5_	4-CF_3_-C_6_H_4_	2 d	11:1	91^b^
6	**8f**	H	C_6_H_5_	3-NO_2_-C_6_H_4_	3 d	6:1	34^c^
7	**8g**	H	C_6_H_5_	1-naphthyl	132 h	4:1	34^b^
8	**8h**	H	C_6_H_5_	2-Me-C_6_H_4_	3 d	45:55	94^b^
9	**8i**	H	C_6_H_5_	2-thienyl	n.c.	–	–
10	**8j**	OMe	C_6_H_5_	C_6_H_5_	24 h	63:1	82
11	**8k**	H	4-MeO-C_6_H_4_	C_6_H_5_	5 d	**8k** only	54
12	**8l**	H	2-thienyl	C_6_H_5_	n.c.	–	–
13	**8m**	H	5-phenyloxazol-2-yl	C_6_H_5_	18 d	4.7:1	37^d^
14	**8n**	NO_2_	C_6_H_5_	C_6_H_5_	3 d	34:1	45
15	**8o**	H	4-NO_2_-C_6_H_4_	C_6_H_5_	4 d	26:1	59
16	**8p**	H	4-F-C_6_H_4_	C_6_H_5_	3 d	23:1	77
17	**8q**	H	1-naphthyl	C_6_H_5_	3 d	5:1	75

^a^Ratio of **8** and **14** determined by GC–MS; ^b^overall yield of C2:C3 product mixture; ^c^**14f** isolated in 10% yield; ^d^conversion according to GC–MS, but could not be isolated.

Considering the aryl donors, excellent yield of products **8** were obtained in case of phenyl (**5a**, 96%, Table 6, entry 1), 4-tolyl (**8d**, 87%, Table 6, entry 4), and 4-methoxyphenyl (**8c**, 94%, Table 6, entry 3) substituents. In case of the phenyl substituent, formation of the corresponding 1,3-diarylindole **14a** was below the detection limit of the GC–MS. In the other two cases 3-arylated byproduct **14** was detected but in negligible amounts (**8**:**14** = 48:1 and 38:1 respectively). The CF_3_ group as electron-withdrawing substituent was well tolerated (Table 6, entry 5). On the other hand, a nitro group in 3-position of the aryl donor furnished 1,2-diarylindole **8f** in a decreased yield of 34% (Table 6, entry 6). Besides **8f**, 10% of 3-(3-nitrophenyl)-1-phenylindole (**14f**) could be isolated as well. Sterically demanding substrates such as 1-naphthyl-substituted indole **5g** (Table 6, entry 7) and 2-tolylindole (**5h**, Table 6, entry 8) afforded the desired products after prolonged reaction time of 3 and 5.5 days, respectively. In case of **5h** an almost quantitative yield of 94% was achieved, but unfortunately containing an inseparable mixture of 1,2-diaryl- **8h** and 1,3-diarylindole isomer **14h** in almost identical amount (Table 6, entry 8). The 1-naphthyl-substituted indole **5g** furnished the product **8g** in the same yield of 34% (Table 6, entry 7) as the 3-nitrophenyl group, again as an inseparable mixture of C2 and C3 isomers. 2-Thiopheneboronic acid as prototype heterocyclic reaction partner was not tolerated at all (Table 6, entry 9). Functional groups on the indole also significantly affected C2-arylation: In case of the electron poorer 5-nitroindole (**5n**) the yield dropped to 45% and required an increased reaction time of 3 days (Table 6, entry 14). On the other hand a good yield of 82% of **8j** was obtained in the reaction with electron-rich 5-methoxyindole (**5j**, Table 6, entry 10). 1-Phenyl-7-azaindole was not applicable as substrate under these conditions. Next, the influence of the aryl group attached to the nitrogen of indole was investigated using phenylboronic acid as aryl source in all cases. Again, the 2-thienyl group was not tolerated (Table 6, entry 12); eventually, catalyst poisoning can be the reason for this observation. 5-Phenyloxazole as N-substituent (**5m**) gave only 37% conversion according to GC–MS after an extremely long reaction time of 18 days (Table 6, entry 13). Respectable yields were obtained with 4-fluorophenyl- (77%, Table 6, entry 16), 4-nitrophenyl- (59%, Table 6, entry 15), and 4-methoxyphenyl- (54%, Table 6, entry 11) substituted indoles. Notably, a bulky substituent such as 1-naphthyl gave a good yield of 75% of **8q** when attached to the nitrogen of the indole (Table 6, entry 17).

**Scheme 6:**
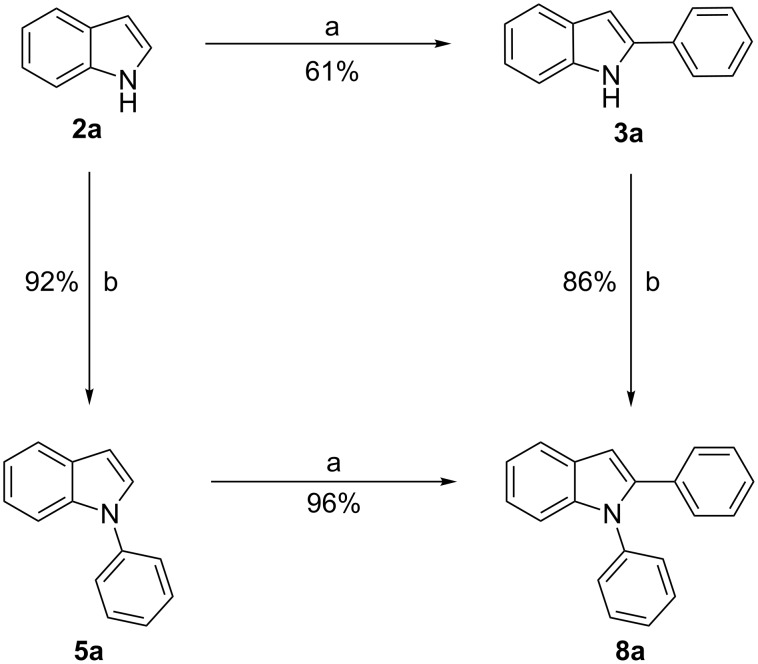
Routes towards 1,2-diarylindoles starting from indole; a: PhB(OH)_2_ (3 equiv), Pd(OAc)_2_ (5 mol %), AcOH, O_2_, rt, 12 h; b: CuI (10 mol %), DMEDA (20 mol %), K_3_PO_4_ (4 equiv), toluene, 135 °C, 12 h.

The other way to synthesize compounds **8** would be an inverse order of arylation, namely C2-arylation before N-arylation (Scheme 2, route F). To compare both routes we prepared **8a** through this second route as well. Starting from indole **2a**, **3a** was prepared in 61% yield, again via the conditions established by Yang (Scheme 6) [59]. Subsequent N-arylation was achieved successfully in 86% yield giving **8a** in an overall yield of 52%. On the other hand, initial N-arylation followed by C2-arylation gave **8a** in 88% overall yield making the latter sequence the more efficient one.

#### Summary of the synthesis of precursors **6–8**

Our efforts towards intermediates **6**–**8** are compiled in Table 7. In summary, both pathways towards intermediates **6** suffer from significant limitations making the synthesis of **1** through **6** an inefficient method. Hence, routes A and B will be of low efficiency, no matter how good the final step will work. Synthesis of intermediate **7** works quite well with good functional group tolerance independent of the starting material **4** or **5**. The same is true for the formation of **8**, both ways, starting from **3** or **5**, seem to be well working. We investigated only C2-arylation of **5** in detail since N-arylation of **3** is well documented [50–51]. In a single example, the synthesis of **8a**, we compared both ways and found the route through compound **5** to be higher yielding (route E). This is an interesting finding since the preparation of 1,2-diarylindoles via initial N-arylation is rarely reported. Independent of the final reaction step towards **1** it is already obvious that a strategy starting from indole derivatives **2** via intermediate **6** will not be the preferred route and that routes C–F look more promising at this point. Next, the conversion of intermediates **6**–**8** to **1** was investigated.

**Table 7:** Overview of synthetic efforts towards intermediates **6**–**8**.

Substrate	Product		

	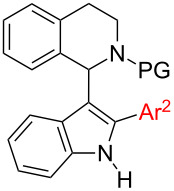 **6**	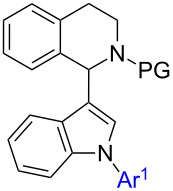 **7**	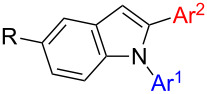 **8**

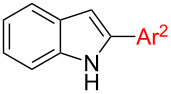 **3**	- Limited scope - FGs groups not well tolerated - Only phenyl gives acceptable yield		Not investigated in detail but formation of **8a** is higher yielding starting from **5** than from **3**
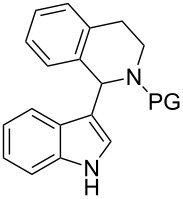 **4**	- Limited scope - FGs groups not well tolerated - Only electron neutral arenes give acceptable yield	- Works well - FGs well accepted - Boc or Cbz as R - If R = H decomposition	
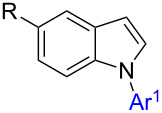 **5**		Works well under Cu catalysis FGs well accepted 45-83% yield	- Works well under Pd catalysis - Good substrate scope - C3-arylation as minor byproduct

### Approaches to target compound **1**

#### Converting **6** into **1** – final step of routes A and B

Even though the synthetic approaches towards **6** were of limited success, N-arylation of this compound class was investigated to assess access to target compounds **1** via intermediates of type **6**. Multiple protocols for N-arylation of indole are reported in literature and have been reviewed [25,46,66]. In this context, iron-catalyzed arylations gained prominence in recent years [67–69]. Especially one of these protocols drew our attention: The group of C. Bolm reported a facile, iron-catalyzed protocol for N-arylation of various N-heterocycles including one example on indole (phenylation in 60% yield), employing aryliodides, *N*,*N*'-dimethylethylenediamine (DMEDA) as ligand, iron(III) chloride as cheap catalyst and potassium phosphate as base in toluene as solvent [60]. As we have previously disclosed a manuscript reporting on iron-catalyzed indolation of *N*-protected-THIQs [22], it would be of high synthetic value to apply the same catalyst for indolation and for subsequent N-arylation or vice versa in a one-pot protocol. Unfortunately, only traces of the desired product **1a** were observed when 1-(2-phenylindol-3-yl)-*N*-Boc-THIQ **6a** was used as starting material in presence of iron(III) chloride as catalyst. Hence, alternative N-arylation conditions were applied, employing CuI instead. The use of CuI has been reported in literature, however, not in context of indole N-arylation [62–63]. In order to drive the reaction to completion, 3 equiv of aryl iodide were added in two portions of 1.5 equiv each (Table 8). Using lower amounts of halide did not lead to complete conversion.

**Table 8:** Scope of copper catalyzed N-arylation of **6a**.

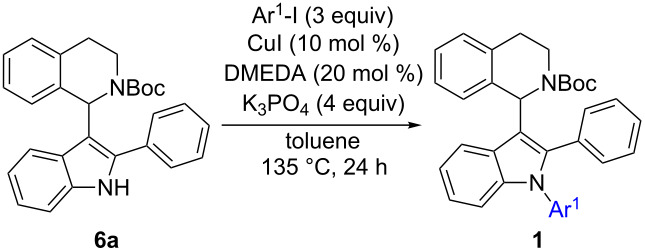			

Entry	Products	Ar^1^	Yield [%]

1	**1a**	C_6_H_5_	68
2	**1b**	4-MeO-C_6_H_4_	84
3	**1c**	2-thienyl	72
4	**1d**	4-NO_2_-C_6_H_4_	54

Good yields were obtained in all four cases investigated using this protocol. Electron-rich iodides gave the best results (Table 8, entries 2 and 3) whereas electron-poor 4-iodonitrobenzene gave somewhat lower yield. Even though 3 equiv of aryl iodide had to be used to achieve completion, it could be demonstrated that most of the excess could be re-isolated (2.14 equiv of 4-iodoanisole were recovered) after column chromatography which is important in cases where expensive aryl halides are employed.

Overall it could be demonstrated that 1-(1,2-diarylindol-3-yl)-*N*-PG-THIQs **1** can be prepared in principal by performing the indole-arylation as last step in the synthetic sequence of routes A and B. Unfortunately, the preparation of 1-(2-aryl-indol-3-yl)-*N*-PG-THIQs **6** is inefficient since indole C2-arylation of 1-(indol-3-yl)-*N*-PG-THIQ **4** is low yielding. CDC coupling of 2-arylated indoles **3** and *N*-Boc-THIQ **9** was even less efficient under the applied reaction conditions. Therefore, routes A and B do not give satisfying results overall, due to problems in only one of the reaction steps. Hence, an alternative strategy had to be developed.

#### Converting **7** into **1** – final step of routes C and D

**Scheme 7:**
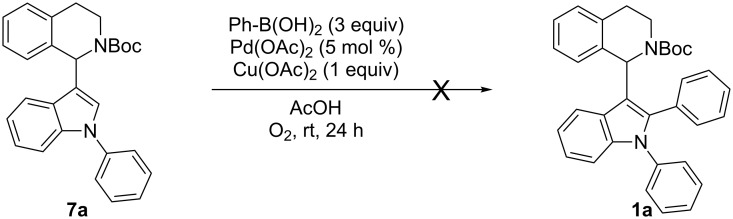
Palladium-catalyzed C2-arylation attempt of 1-(1-phenylindol-3-yl)-*N*-Boc-THIQ.

Since two routes to 1-(1-arylindol-3-yl)-THIQs **7** were successfully established, the final C2-arylation step towards 1-(1,2-diarylindol-3-yl)-THIQs **1** was examined. Initially, 1-(1-phenylindol-3-yl)-*N*-Boc-THIQ **7a** was subjected to C2-arylation conditions (Scheme 7). However, the desired product **1a** was not detected and only starting material was recovered no matter whether **7a** or **7h** was used as starting material. Steric hindrance is the most obvious reason for this failure. Another problem we encountered was that **1a** (prepared from another route) and **7a** have identical *R*_f_ values which would make purification of the final product extremely difficult. Hence, it was decided to change to another sequence instead of further optimizing this procedure.

#### Converting **8** into **1** – final step of routes E and F

Starting materials **8** were subjected to standard indolation conditions, using copper(II) nitrate as catalyst (Table 9). This also applies to the isolated, inseparable C2- (**8**) and C3-substituted (**14**) mixtures, since the C3-arylated compounds remain unreacted in the present transformation and can be separated from the desired product easily.

**Table 9:** Substrate scope for reactions of 1,2-diarylindoles **8** with *N*-Boc-THIQ **9**.

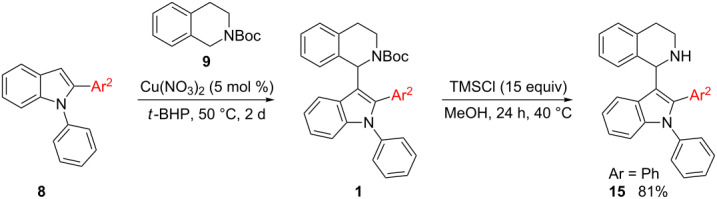			

Entry	Products	Ar^2^	Yield [%]

1	**1a**	C_6_H_5_	48
2	**1e**	4-MeO-C_6_H_4_	63
3	**1f**	4-Me-C_6_H_4_	59
4	**1g**	4-CF_3_-C_6_H_4_	46
5	**1h**	3-NO_2_-C_6_H_4_	41
6	**1i**	1-naphthyl	30^a^
7	**1j**	2-Me-C_6_H_4_	37^b^

^a^5 days instead of 2 days; 45% **8g** recovered. ^b^5 days instead of 2 days; 62% **8h** recovered.

In case of 1,2-diphenylindole (**8a**), 48% of desired product **1a** were obtained under copper catalysis (Table 9, entry 1). Good yields with respect to indolation were obtained in case of 4-methoxyphenyl- (**1e**) and 4-tolyl- (**1f**) substituents (63% and 59% respectively, Table 9, entries 2 and 3). Deactivated substrates such as 4-(trifluoromethyl)phenyl-substituted **8d** and 3-nitrophenyl-substituted **8e** afforded the corresponding products in decreased yield (46%, Table 9, entry 4 and 41%, Table 9, entry 5). For sterically demanding substrates such as **8g** and **8h** (Table 9, entries 6 and 7) the standard indolation conditions had to be modified in order to increase conversion to the desired product **1** (see Table 9, note a). Still, only a moderate yield of 37% was achieved in case of 2-tolyl- (**1j**, Table 9, entry 7) and 30% with the 1-naphthyl-substituted product **1i** (Table 9, entry 6). As proof of concept, the Boc-protective group could be removed via our previously established protocol by employing TMSCl as mild reagent for deprotection giving 81% of **15** [22]. Hence, routes E and F were completed successfully with the former giving the best results of all six sequences.

## Conclusion

The results of the final steps towards target structure **1** are compiled in Table 10, the results for the synthesis of intermediates **6**–**8** in Table 7.

**Table 10:** Comparison of the final reaction step towards **1** starting from intermediates **6**–**8**.

Substrate	Product	

	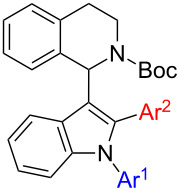 **1**	

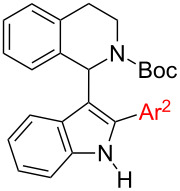 **6**	Ar^1^ = Ar^2^ = C_6_H_5_ Ar^1^ = 4-MeO-C_6_H_4_, Ar^2^ = C_6_H_5_ Ar^1^ = 2-thienyl, Ar^2^ = C_6_H_5_ Ar^1^ = 4-NO_2_-C_6_H_4_, Ar^2^ = C_6_H_5_	68% **1a** 84% **1b** 72% **1c** 54% **1d**
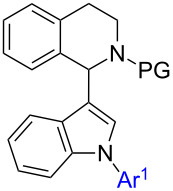 **7**	Not successful	
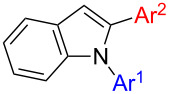 **8**	Ar^1^ = Ar^2^ = C_6_H_5_ Ar^1^ = C_6_H_5_, Ar^2^ = 4-MeO-C_6_H_4_ Ar^1^ = C_6_H_5_, Ar^2^ = 4-Me-C_6_H_4_ Ar^1^ = C_6_H_5_, Ar^2^ = 4-CF_3_-C_6_H_4_ Ar^1^ = C_6_H_5_, Ar^2^ = 3-NO_2_-C_6_H_4_ Ar^1^ = C_6_H_5_, Ar^2^ = 1-naphtyl Ar^1^ = C_6_H_5_, Ar^2^ = 2-Me-C_6_H_4_	48% **1a** 63% **1e** 59% **1f** 46% **1g** 41% **1h** 30% **1i**^a^ 37% **1j**^b^

^a^45% **8g** recovered; ^b^62% **8h** recovered.

From these tables it can be seen that synthetic routes towards target compounds **1** are unsuitable in cases where either intermediate **6** or **7** is involved (routes A–D). The problem with intermediate **6** is that its formation is low yielding which cannot be compensated by the well-working conversion of **6** to **1**. Intermediate **7** on the other hand can be synthesized efficiently; however, it cannot be converted to the target compounds **1**. Hence, only strategies involving 1,2-diarylindole intermediate **8** lead to the target compounds **1** reliably (routes E and F).

It has to be mentioned that we limited ourselves to iron, copper, and palladium-catalyzed protocols in this study. Hence, further screening of other reaction conditions could also lead to better results in the other approaches.

On the way to the target compounds an efficient synthesis of 1,2-diarylindoles **8** was developed via initial N-arylation and subsequent C2-arylation, a route which was largely neglected in literature, so far. In this reaction sequence the substrate scope was investigated thoroughly, as well. Additionally, 1-aryl-3-THIQ-indoles **7** and 2-aryl-3-THIQ-indoles **6** were synthesized on the way to the target compounds. From the former compound class only two derivatives have been reported [9], the latter has not been disclosed previously.

The final products obtained combine structural motifs from compound classes showing diverse biological activity. It is hoped that also the synthesized products will display activity which will be investigated in the near future.

## Supporting Information

File 1 Experimental procedures, analytical data, and copies of NMR spectra of compounds unknown in the literature.

## References

[1] Scott J D, Williams R M (2002). Chem Rev.

[2] Lee W-H, Chen P-L, Zhou L, Zhu J (2007). Compositions and methods related to RAD51 inactivation in the treatment of neoplastic diseases, and especially CML. WO Patent Application.

[3] Zhao D, Cheng M, Huang W, Song S, Jiang Z (2011). 6,7-Methylene-dioxy-1,2,3,4-tetrahydroisoquinoline derivative and preparation method and application thereof. Chinese Patent Application.

[4] Lal S, Snape T J (2012). Curr Med Chem.

[5] Biswal S, Sahoo U, Sethy S, Kumar H K S, Banerjee M (2012). Asian J Pharm Clin Res.

[6] Rhoennstad P, Apelqvist T, Wennerstaal M, Cheng A, Gordon S (2010). Novel estrogen receptor ligands. WO Patent Application.

[7] Slade R, Klimova Y, Halter R J, Yungai A J, Weiner W S, Walton R J, Willardsen J A, Anderson M B, Zavitz K (2008). Compounds for Alzheimer´s disease. U.S. Patent Application.

[8] Klein C, Gassman A D, Bhoite L, Manfredi J (2007). Compounds for diseases and disorders. WO Patent Application.

[9] Wang M-Z, Zhou C-Y, Wong M-K, Che C-M (2010). Chem – Eur J.

[10] Okada H (2009). Electrophotographic photoreceptor. Japanese Patent Application.

[11] Venkov A P, Statkova-Abeghe S M, Donova A K (2004). Cent Eur J Chem.

[12] Li Z, Li C-J (2005). J Am Chem Soc.

[13] Li C-J (2009). Acc Chem Res.

[14] Girard S A, Knauber T, Li C-J (2014). Angew Chem, Int Ed.

[15] Li Z, Li C-J (2005). J Am Chem Soc.

[16] Alagiri K, Kumara G S R, Prabhu K R (2011). Chem Commun.

[17] Su W, Yu J, Li Z, Jiang Z (2011). J Org Chem.

[18] Liu P, Zhou C-Y, Xiang S, Che C-M (2010). Chem Commun.

[19] Boess E, Schmitz C, Klussmann M (2012). J Am Chem Soc.

[20] So M-H, Liu Y, Ho C-M, Che C-M (2009). Chem – Asian J.

[21] Shirakawa E, Yoneda T, Moriya K, Ota K, Uchiyama N, Nishikawa R, Hayashi T (2011). Chem Lett.

[22] Ghobrial M, Harhammer K, Mihovilovic M D, Schnürch M (2010). Chem Commun.

[23] Ghobrial M, Schnürch M, Mihovilovic M D (2011). J Org Chem.

[24] McGlacken G P, Bateman L M (2009). Chem Soc Rev.

[25] Joucla L, Djakovitch L (2009). Adv Synth Catal.

[26] Godula K, Sames D (2006). Science.

[27] Yeung C S, Dong V M (2011). Chem Rev.

[28] Wencel-Delord J, Dröge T, Liu F, Glorius F (2011). Chem Soc Rev.

[29] Ackermann L (2011). Chem Rev.

[30] Jazzar R, Hitce J, Renaudat A, Sofack-Kreutzer J, Baudoin O (2010). Chem – Eur J.

[31] Schnürch M, Dastbaravardeh N, Ghobrial M, Mrozek B, Mihovilovic M D (2011). Curr Org Chem.

[32] Lebrasseur N, Larrosa I (2012). Adv Heterocycl Chem.

[33] Boorman T C, Larrosa I (2011). Prog Heterocycl Chem.

[34] Beck E M, Gaunt M J (2010). Top Curr Chem.

[35] Wang X, Gribkov D V, Sames D (2007). J Org Chem.

[36] Ackermann L, Barfüßer S (2009). Synlett.

[37] Cusati G, Djakovitch L (2008). Tetrahedron Lett.

[38] Zhang Z, Hu Z, Yu Z, Lei P, Chi H, Wang Y, He R (2007). Tetrahedron Lett.

[39] Bellina F, Benelli F, Rossi R (2008). J Org Chem.

[40] Cornella J, Lu P, Larrosa I (2009). Org Lett.

[41] Miyasaka M, Fukushima A, Satoh T, Hirano K, Miura M (2009). Chem – Eur J.

[42] Phipps R J, Grimster N P, Gaunt M J (2008). J Am Chem Soc.

[43] Chen S, Liao Y, Zhao F, Qi H, Liu S, Deng G-J (2014). Org Lett.

[44] Miao T, Li P, Wang G-W, Wang L (2013). Chem – Asian J.

[45] Huang Y, Ma T, Huang P, Wu D, Lin Z, Cao R (2013). ChemCatChem.

[46] Xu H (2009). Mini-Rev Org Chem.

[47] Swapna K, Murthy S N, Nageswar Y V D (2010). Eur J Org Chem.

[48] Verma A K, Singh J, Larock R C (2009). Tetrahedron.

[49] Periasamy M, Vairaprakash P, Dalai M (2008). Organometallics.

[50] Old D W, Harris M C, Buchwald S L (2000). Org Lett.

[51] Grasa G A, Viciu M S, Huang J, Nolan S P (2001). J Org Chem.

[52] Daugulis O, Do H-Q, Shabashov D (2009). Acc Chem Res.

[53] Do H-Q, Khan R M K, Daugulis O (2008). J Am Chem Soc.

[54] Sagnes C, Fournet G, Joseph B (2009). Synlett.

[55] Ackermann L, Dell'Acqua M, Fenner S, Vicente R, Sandmann R (2011). Org Lett.

[56] Daly S, Hayden K, Malik I, Porch N, Tang H, Rogelj S, Frolova L V, Lepthien K, Kornienko A, Magedov I V (2011). Bioorg Med Chem Lett.

[57] Nadres E T, Lazareva A, Daugulis O (2011). J Org Chem.

[58] Wu M, Luo J, Xiao F, Zhang S, Deng G-J, Luo H-A (2012). Adv Synth Catal.

[59] Yang S-D, Sun C-L, Fang Z, Li B-J, Li Y-Z, Shi Z-J (2008). Angew Chem, Int Ed.

[60] Correa A, Bolm C (2007). Angew Chem, Int Ed.

[61] Phillips D P, Zhu X-F, Lau T L, He X, Yang K, Liu H (2009). Tetrahedron Lett.

[62] Kwong F Y, Klapars A, Buchwald S L (2002). Org Lett.

[63] Antilla J C, Klapars A, Buchwald S L (2002). J Am Chem Soc.

[64] Lane B S, Sames D (2004). Org Lett.

[65] Liang Z, Yao B, Zhang Y (2010). Org Lett.

[66] Cacchi S, Fabrizi G (2005). Chem Rev.

[67] Correa A, Garcia Mancheño O, Bolm C (2008). Chem Soc Rev.

[68] Taillefer M, Xia N, Ouali A (2007). Angew Chem, Int Ed.

[69] Bolm C, Legros J, Le Paih J, Zani L (2004). Chem Rev.

