# Supplementary material for: Exploration of C–H and N–H-bond functionalization towards 1-(1,2-diarylindol-3-yl)tetrahydroisoquinolines
Source: Beilstein J Org Chem. 2014 Sep 15;10:2186–99. doi: 10.3762/bjoc.10.226 (PMC4168884; doi:10.3762/bjoc.10.226)
Supplement: File 1 — Experimental procedures, analytical data, and copies of NMR spectra of compounds unknown in the literature. [file Beilstein_J_Org_Chem-10-2186-s001.pdf]

Supporting Information

for

Exploration of C–H and N–H-bond functionalization towards 1-(1,2-diarylindol-3-yl)-tetrahydroisoquinolines

Michael Ghobrial, Marko D. Mihovilovic and Michael Schnürch*

Address: Institute of Applied Synthetic Chemistry, Vienna University of Technology, 1060 Vienna, Austria

Email: Michael Schnürch - michael.schnuerch@tuwien.ac.at

* Corresponding author

Experimental procedures, analytical data, and copies of NMR spectra of compounds unknown in the literature

Table of contents

1. General S2
   1.1. Caution S2
   1.2. Instrumentation S2
2. Experimental S2
3. Copies of ^{1}H and ^{13}C NMR spectra of compounds S18

1. General

Unless otherwise noted, chemicals were purchased from commercial suppliers and used without further purification. Flash column chromatography was performed on silica gel (40-63 µm). For thin layer chromatography (TLC) aluminum coated silica gel was used and signals were visualized with UV light (254 nm).

1.1. Caution

Mixing a metal salt and peroxide can cause explosion. See: Jones, A. K.; Wilson, T. E.; Nikam, S. S. In Encyclopedia of Reagents for Organic Synthesis, Paquette, L. A. Ed.; John Wiley & Sons, Inc. 1995, 2, 880.

1.2. Instrumentation

GC-MS runs were performed using a standard capillary column (30m x 0.32 mm ID), applying the following standardized temperature profile: 2 minutes at 80 °C, 15 °C/min until 320°C, 7 minutes at 320 °C. All samples subjected to HR-MS were analyzed by LC-IT-TOF-MS in only positive ion detection mode upon recording of MS and MS/MS spectra. For the evaluation in the following, only positive ionization spectra were used (where the quasi-molecular ion is the one of $[M+H]^+$), and further data or information were not taken into consideration. Microwave reactions were performed on a BIOTAGE InitiatorTM sixty microwave unit. Melting points were determined using a Kofler-type hot stage microscope and are uncorrected. ^{1}H-NMR and ^{13}C-NMR spectra were recorded either on 200 MHz or 400 MHz spectrometer. Chemical shifts are reported as ppm downfield from TMS (tetramethylsilane) as internal standard with multiplicity, number of protons, allocation, and coupling constant(s) in Hertz.

2. Experimental

General notes and instrumentation are provided in the supporting information as well as NMR-spectra of all new compounds. Compounds **3a-d** [1], **4a-d** [2,3], **9** [1] and N-arylated indoles **10a-q** [4,5] were synthesized according to literature procedures, and analytical data are in agreement with literature reports (see supporting info) [1-7].

General procedure A: N-Arylation of 6a towards 1a-d (see Table 8). Substrate **6a** (100 mg, 0.287 mmol, 1.0 equiv.), copper(I)-iodide (4.7 mg, 28.7 µmol, 0.10 equiv.), and K_3PO_4 (100 mg, 0.471 mmol, 2.0 equiv.) were placed in an oven-dried glass vial (2 mL), and aryliodide (0.353 mmol, 1.5 equiv.) and DMEDA (4.6 µL, 47.1 µmol) were added under an argon atmosphere, followed by dry toluene (300 µL). The tube was sealed under argon, and the mixture was heated to 135 °C and stirred at this

temperature for 12 hours. In order to achieve full conversion, another batch of aryliodide (0.353 mmol, 1.5 equiv.) and base (100 mg, 0.471 mmol, 2.0 equiv.) were added and the reaction mixture stirred 12 hours further. The reaction mixture was cooled down to room temperature, diluted with DCM and directly subjected to column chromatography using PE:EtOAc =100:0 → 0:30 (90 minutes) to afford the desired product **1a-d**.

General procedure B: 1,2-diarylindolation of N-Boc-THIQ 9 towards 1a,b,e-i (see table 9): Substrate **9** (100 mg, 0.429 mmol, 1.0 equiv.), copper(II)-nitrate trihydrate (5.2 mg, 21.5 µmol), and 1,2-arylindole derivative **8** (0.515 mmol, 1.2 equiv.) were placed into a 5 mL glass vial. Then, tert-butyl hydroperoxide (112 µL, 0.558 mmol in decane, 1.3 equiv.) was added dropwise at 0 °C. The reaction mixture was capped and stirred for 10 minutes at 0 °C and then slowly heated to 50 °C and stirred for 2 days, unless stated differently. The reaction mixture was cooled to room temperature, diluted with DCM and directly subjected to column chromatography using PE:EtOAc =100:0 →0:30 (90 minutes) to afford the desired products **1a,b,e-i**.

1,1-Dimethylethyl 1-(1,2-diphenyl-1*H*-indol-3-yl)-3,4-dihydroisoquinoline-2(1*H*)-carboxylate (1a). *General procedure* A, table 8: Yield: 98 mg (68 %) *General procedure B:* Yield: 205 mg (48 %); white powder; M.p.: 78-81 °C; TLC: R_f(PE:Et_2O=3:1) = 0.43; GC/MS (EI+): m/z (rel. Intensity): 430 (M^+-100, 69), 399 (100), 397 (19), 384 (14), 323 (32), 295 (26), 294 (20), 269 (25), 267 (17), 200 (28), 199 (10), 165 (16), 132 (12), 131 (12), 130 (32), 103 (12), 77 (20); HRMS (ESI$^+$): exact mass calculated for $C_{34}H_{32}N_2O_2$: 523.2356. Found: 523.2363 $[M+Na]^+$; ^{1}H NMR (200 MHz, *CDCl$_3$*): δ = 1.21 (s, 9H), 2.47 (ddd, ^2J = 16.4 Hz, 3.7 Hz, 1.3 Hz, 1H), 2.89 (ddd, ^2J = 16.6 Hz, ^3J = 12.1 Hz, ^3J = 6.0 Hz, 1H), 3.22 (ddd, ^2J = 13.2 Hz, ^3J = 12.3 Hz, ^3J = 4.0 Hz, 1H), 4.00 (dd, ^2J = 13.4 Hz, ^3J = 5.8 Hz, 1H), 6.61 (s, 1H), 6.87-7.21 (m, 17H), 7.27 (dd, ^3J = 7.3 Hz, ^4J = 1.2 Hz, 1H); ^{13}C NMR (50 MHz, *CDCl$_3$*): δ = 28.2 (t), 28.3 (q, 3C), 38.6 (t), 51.2 (d), 79.5 (s), 110.4 (d), 116.2 (s), 120.0 (d), 120.3 (d), 122.1 (d), 125.9 (d), 126.4 (d), 127.0 (d), 127.5 (d, 3C overlapping), 127.8 (s), 128.20 (d), 128.24 (d, 2C), 128.72 (d), 128.76 (d, 2C), 131.3 (d, 2C), 131.8 (s), 134.8 (s), 137.16 (s), 137.23 (s), 137.8 (s), 139.4 (s), 154.3 (s)

1,1-Dimethylethyl 1-[1-(4-methoxyphenyl)-2-phenyl-1*H*-indol-3-yl]-3,4-dihydroisoquinoline-2(1*H*)-carboxylate (1b). *General procedure A,* table 8: Yield: 128 mg (84 %); colorless solid; M.p.: 88-92 °C; TLC: R_f(PE:Et_2O=3:1) = 0.22; GC/MS (EI$^+$): m/z (rel. Intensity): 430 (M^+-100, 78), 429 (100), 428 (25), 427 (70), 414 (18), 353 (36), 325 (21), 299 (16), 254 (17), 216 (24), 215 (24), 191 (20), 177 (22), 165 (18), 132 (32), 131 (31), 130 (64), 117 (10), 103 (18), 96 (11), 77 (25); HRMS (ESI$^+$): exact mass calculated for $C_{35}H_{34}N_2O_3$: 553.2462. Found: 553.2465 $[M+Na]^+$; ^{1}H NMR (200 MHz, *CDCl$_3$*): δ = 1.35 (s, 9H), 2.60 (ddd, ^2J = 16.2 Hz, ^3J = 3.6 Hz, ^3J = 1.2 Hz, 1H), 3.03 (ddd, ^2J = 16.2 Hz, ^3J = 12.2 Hz, ^3J = 6.0 Hz, 1H), 3.36 (dt, ^2J = 13.0 Hz, ^3J = 12.7 Hz, ^3J = 3.7 Hz, 1H), 3.78 (s, 3H), 4.13 (dd, ^2J = 13.3 Hz, ^3J = 5.7 Hz, 1H), 6.73 (s, 1H), 6.82 (d, ^3J = 8.9 Hz, 2H), 7.00-7.24 (m, 14H), 7.40 (d, ^3J = 7.7 Hz, 1H); ^{13}C NMR (50 MHz, *CDCl$_3$*): δ = 28.2 (t), 28.3 (q, 3C), 38.5 (t), 51.2 (d), 55.2 (q), 79.4 (s), 110.4 (d), 114.0 (d, 2C), 115.8 (s), 119.9 (d), 120.2 (d), 121.9 (d), 125.9 (d), 126.3 (d), 127.45 (d), 127.52 (d, 2C), 127.6 (s), 128.2 (d), 128.7 (d), 129.4 (d, 2C), 130.5 (s), 131.3 (d, 2C), 131.9 (s), 134.8 (s), 137.3 (s), 137.5 (s), 139.7 (s), 154.3 (s), 158.3 (s)

1,1-Dimethylethyl 1-[2-phenyl-1-(thiophen-2-yl)-1*H*-indol-3-yl]-3,4-dihydroisoquinoline-2(1*H*)-carboxylate (1c). Prepared according to *general procedure A,* table 8*:* Yield: 86 mg (72 %); white powder; M.p.: 87-89 °C; TLC: R_f(PE:EtOAc=5:1) = 0.54; GC/MS (EI+): m/z (rel. Intensity): 406 (M^+-100,

83), 405 (100), 404 (11), 403 (31), 329 (34), 301 (20), 275 (17), 241 (12), 216 (17), 203 (21), 186 (46), 178 (16), 171 (22), 165 (18), 164 (21), 151 (11), 132 (30), 131 (20), 130 (52), 117 (13), 115 (10), 105 (13), 103 (19), 77 (18); HRMS (ESI^+): exact mass calculated for $C_{32}H_{30}N_2O_2S$: 529.1920. Found: 529.1926 $[M+Na]^+$; ^{1}H NMR (200 MHz, *CDCl₃*): δ = 1.32 (s, 9H), 2.54 (ddd, ^2J = 16.5 Hz, ^3J = 3.9 Hz, ^3J = 1.6 Hz, 1H), 2.97 (ddd, ^2J = 16.6 Hz, ^3J = 11.8 Hz, ^3J = 6.1 Hz, 1H), 3.28 (ddd, ^2J = 13.1 Hz, ^3J = 12.3 Hz, ^3J = 4.0 Hz, 1H), 4.08 (dd, ^2J = 13.4 Hz, ^3J = 5.7 Hz, 1H), 6.67 (s, 1H), 6.80 (dd, ^3J = 3.7 Hz, ^4J = 1.5 Hz, 1H), 6.85 (dd, ^3J = 5.4 Hz, ^3J = 3.7 Hz, 1H), 6.96-7.24 (m, 12H), 7.29 (dd, ^3J = 7.7 Hz, ^4J = 1.4 Hz, 1H), 7.41 (dd, ^3J = 7.6 Hz, ^4J = 1.1 Hz, 1H); ^{13}C NMR (101 MHz, *$CDCl_3$*): δ = 28.1 (t), 28.3 (q, 3C), 38.5 (t), 51.1 (d), 79.6 (s), 110.7 (d), 117.1 (s), 120.0 (d), 120.9 (d), 122.6 (d), 124.5 (d), 125.2 (d), 125.7 (d), 126.0 (d), 126.5 (d), 127.5 (d, 2C), 127.9 (d), 128.2 (d), 128.7 (d), 131.25 (d, 2C), 131.32 (s), 134.8 (s), 136.9 (s), 138.4 (s), 139.2 (s), 140.3 (s), 154.3 (s); (1 quaternary C missing due to potential overlap of signals)

1,1-Dimethylethyl 1-[1-(4-nitrophenyl)-2-phenyl-1*H*-indol-3-yl]-3,4-dihydroisoquinoline-2(1*H*)-carboxylate (1d). Prepared according to *general procedure A,* table 8*:* Yield: 70 mg (54 %); shining yellow powder; M.p.: 117-119 °C; TLC: R_f(PE:EtOAc=5:1) = 0.57; GC/MS (EI+): m/z (rel. Intensity): 445 (M^+-100, 78), 444 (100), 443 (10), 442 (27), 429 (12), 398 (19), 368 (37), 340 (20), 339 (13), 293 (19), 292 (17), 267 (18), 223 (18), 217 (11), 199 (31), 191 (35), 184 (25), 177 (33), 165 (15), 146 (13), 132 (45), 131 (39), 130 (74), 117 (12), 105 (15), 103 (22), 77 (18); HRMS (ESI^+): exact mass calculated for $C_{34}H_{31}N_3O_4$: 546.2387. Found: 546.2375 $[M+H]^+$; ^{1}H NMR (200 MHz, *$CDCl_3$*): δ = 1.30 (s, 9H), 2.54 (ddd, ^2J = 16.3 Hz, ^3J = 3.6 Hz, ^3J = 1.6 Hz, 1H), 2.95 (ddd, ^2J = 16.4 Hz, ^3J = 12.0 Hz, ^3J = 5.9 Hz, 1H), 3.23 (ddd, ^2J = 13.1 Hz, ^3J = 12.3 Hz, ^3J = 3.8 Hz, 1H), 4.05 (dd, ^2J = 13.3 Hz, ^3J = 5.5 Hz, 1H), 6.66 (s, 1H), 6.93-7.29 (m, 14H), 7.40 (dd, ^3J = 7.5 Hz, ^4J = 0.9 Hz, 1H), 8.12 (d, ^3J = 8.9 Hz, 2H); ^{13}C NMR (101 MHz, *$CDCl_3$*): δ = 28.2 (t), 28.3 (q, 3C), 38.8 (t), 51.0 (d), 79.7 (s), 110.0 (d), 118.3 (s), 120.6 (d), 121.4 (d), 123.0 (d), 124.4 (d, 2C), 126.1 (d), 126.6 (d), 128.0 (d, 2C), 128.14 (d), 128.16 (d), 128.3 (d, 2C), 128.5 (s), 128.9 (d), 131.1 (s), 131.3 (d, 2C), 134.9 (s), 136.5 (s), 136.7 (s), 138.7 (s), 143.8 (s), 145.7 (s), 154.3 (s).

1,1-Dimethylethyl 1-[2-(4-methoxyphenyl)-1-phenyl-1*H*-indol-3-yl]-3,4-dihydroisoquinoline-2(1*H*)-carboxylate (1e). *General procedure B, table 9:* Yield: Cu: 143 mg (63 %), Fe: n.a.; colorless solid; M.p.: 93-95 °C; TLC: R_f(PE:EtOAc=5:1) = 0.43; GC/MS (EI+): m/z (rel. Intensity): 430 (M^+-100, 76), 429 (100), 427 (44), 414 (19), 325 (16), 323 (24), 299 (19), 294 (17), 284 (14), 254 (18), 215 (50), 191 (23), 184 (20), 177 (22), 170 (16), 132 (26), 131 (26), 130 (59), 105 (12), 103 (16), 77 (30); HRMS (ESI^+): exact mass calculated for $C_{35}H_{34}N_2O_3$: 553.2462. Found: 553.2470 $[M+Na]^+$; ^{1}H NMR (200 MHz, *$CDCl_3$*): δ = 1.32 (s, 9H), 2.58 (ddd, ^2J = 16.3 Hz, ^3J = 3.8 Hz, ^3J = 1.6 Hz, 1H), 2.99 (ddd, ^2J = 16.5 Hz, ^3J = 12.1 Hz, ^3J = 6.0 Hz, 1H), 3.30 (ddd, ^2J = 13.1 Hz, ^3J = 12.1 Hz, ^3J = 3.7 Hz, 1H), 3.72 (s, 3H), 4.08 (ddd, ^2J = 13.4 Hz, ^3J = 5.8 Hz, ^3J = 1.2 Hz, 1H), 6.63 (d, ^3J = 8.8 Hz, 2H), 6.66 (s, 1H), 6.93 (d, ^3J = 8.7 Hz, 2H), 6.97-7.38 (m, 13H); ^{13}C NMR (50 MHz, *$CDCl_3$*): δ = 28.2 (t), 28.3 (q, 3C), 38.6 (t), 51.3 (d), 55.1 (q), 79.5 (s), 110.3 (d), 113.1 (d, 2C), 116.1 (s), 119.9 (d), 120.3 (d), 121.9 (d), 124.1 (s), 126.0 (d), 126.3 (d), 126.9 (d), 127.8 (s), 128.2 (d), 128.3 (d, 2C), 128.7 (d), 128.8 (d, 2C), 132.5 (d, 2C), 134.8 (s), 137.1 (s), 137.4 (s), 137.9 (s), 139.3 (s), 154.4 (s), 158.8 (s).

1,1-Dimethylethyl 1-[2-(4-methylphenyl)-1-phenyl-1*H*-indol-3-yl]-3,4-dihydroisoquinoline-2(1*H*)-carboxylate (1f). Prepared according to *general procedure B, table 9:* Yield: Cu: 131 mg (59 %), Fe: n.a.; white powder; M.p.: 91-93 °C; TLC: R_f(PE:EtOAc=5:1) = 0.62; GC/MS (EI+): m/z (rel. Intensity): 414 (M^+-100, 72), 413 (100), 398 (17), 323 (36), 309 (20), 294 (24), 283 (26), 191 (18), 177 (16), 165

(19), 132 (22), 131 (21), 130 (52), 103 (15), 77 (27); HRMS (ESI^+): exact mass calculated for $C_{35}H_{34}N_2O_2$: 515.2693. Found: 515.2697 $[M+Na]^+$; ^1H NMR (200 MHz, *CDCl₃*): δ = 1.29 (s, 9H), 2.25 (s, 3H), 2.60 (ddd, ^2J = 16.3 Hz, ^3J = 3.8 Hz, ^3J = 1.7 Hz, 1H), 3.00 (ddd, ^2J = 16.6 Hz, ^3J = 12.0 Hz, ^3J = 6.0 Hz, 1H), 3.33 (ddd, ^2J = 13.2 Hz, ^3J = 12.2 Hz, ^3J = 4.0 Hz, 1H), 4.09 (ddd, ^2J = 13.5 Hz, ^3J = 4.7 Hz, ^3J = 1.1 Hz, 1H), 6.65 (s, 1H), 6.87-7.37 (m, 17H); ^{13}C NMR (50 MHz, *$CDCl_3$*): δ = 21.2 (q), 28.2 (q, 3C), 28.4 (t), 38.7 (t), 51.4 (d), 79.5 (s), 110.4 (d), 116.1 (s), 119.9 (d), 120.3 (d), 121.9 (d), 126.0 (d), 126.3 (d), 126.9 (d), 127.8 (s), 128.2 (d), 128.3 (d, 2C), 128.4 (d, 2C), 128.7 (d), 128.8 (d, 2C), 131.1 (d, 2C), 134.9 (s), 137.2 (s, 2C overlapping), 137.4 (s), 137.9 (s), 139.6 (s), 154.4 (s); (1 C missing due to potential overlap of signals); ^1H NMR (200 MHz, *acetone-d_6*): δ = 1.31 (s, 9H), 2.25 (s, 3H), 2.69 (ddd, ^2J = 16.4 Hz, ^3J = 4.0 Hz, ^3J = 1.6 Hz, 1H), 2.97 (ddd, ^2J = 16.6 Hz, ^3J = 11.9 Hz, ^3J = 6.1 Hz, 1H), 3.42 (ddd, ^2J = 13.5 Hz, ^3J = 12.0 Hz, ^3J = 4.3 Hz, 1H), 4.10 (ddd, ^2J = 13.3 Hz, ^3J = 5.9 Hz, ^3J = 1.1 Hz, 1H), 6.65 (s, 1H), 6.90-7.42 (m, 17H); ^{13}C NMR (50 MHz, *acetone-d_6*): δ = 22.2 (q), 29.4 (q, 3C), 29.8 (t), 40.6 (t), 53.2 (d), 80.7 (s), 112.1 (d), 117.9 (s), 121.8 (d), 122.0 (d), 123.8 (d), 127.8 (d), 128.3 (d), 129.1 (d), 129.7 (s), 129.9 (d), 130.2 (d, 2C), 130.3 (d, 2C), 130.7 (d), 130.8 (s), 130.9 (d, 2C), 133.2 (d, 2C), 136.7 (s), 139.1 (s), 139.3 (s), 139.5 (s), 139.9 (s), 141.5 (s), 155.9 (s)

1,1-Dimethylethyl 1-[1-phenyl-2-[4-(trifluoromethyl)-phenyl]-1*H*-indol-3-yl]-3,4-dihydroisoquinoline-2(1*H*)-carboxylate (1g). Prepared according to *general procedure B, table 9:* Yield: Cu: 111 mg (46 %), Fe: n.a.; white powder; M.p.: 88-89 °C; TLC: R_f (PE:EtOAc=5:1) = 0.58; GC/MS (EI+): m/z (rel. Intensity): 468 (M^+-100, 60), 467 (81), 363 (18), 337 (14), 323 (31), 294 (23), 234 (39), 217 (12), 216 (13), 191 (18), 165 (22), 132 (49), 131 (52), 130 (100), 105 (18), 104 (18), 103 (35), 96 (22), 77 (52); HRMS (ESI^+): exact mass calculated for $C_{35}H_{31}F_3N_2O_2$: 569.2410. Found: 569.2410 $[M+H]^+$; ^1H NMR (200 MHz, *acetone-d_6*): **δ** = 1.34 (s, 9H), 2.66 (ddd, ^2J = 16.5 Hz, ^3J = 4.2 Hz, ^3J = 1.3 Hz, 1H), 2.97 (ddd, ^2J = 16.7 Hz, ^3J = 12.0 Hz, ^3J = 6.2 Hz, 1H), 3.43 (ddd, ^2J = 13.5 Hz, ^3J = 12.2 Hz, ^3J = 4.3 Hz, 1H), 4.10 (ddd, ^2J = 13.7 Hz, ^3J = 6.1 Hz, ^3J = 1.3 Hz, 1H), 6.71 (s, 1H), 6.94-7.55 (m, 17H); ^{13}C NMR (50 MHz, *acetone-d_6*): **δ** = 29.5 (q, 3C), 29.7 (t), 40.4 (t), 52.9 (d), 80.8 (s), 112.2 (d), 118.7 (s), 122.2 (d), 122.3 (d), 124.4 (d), 126.17 (d, 2C, q, $^3J_{CF}$ = 3.8 Hz), 126.22 (s, q, $^1J_{CF}$ = 271 Hz), 127.9 (d), 128.5 (d), 129.5 (d), 130.1 (d), 130.3 (d, 2C), 130.7 (d), 131.0 (d, 2C), 134.0 (d, 2C), 136.7 (s), 138.0 (s, q, $^5J_{CF}$ = 1.3 Hz), 138.5 (s), 139.4 (s), 139.7 (s), 139.9 (s), 155.7 (s); (1 C missing due to potential overlap of signals)

1,1-Dimethylethyl 1-[2-(3-nitrophenyl)-1-phenyl-1*H*-indol-3-yl]-3,4-dihydroisoquinoline-2(1*H*)-carboxylate (1h). Prepared according to *general procedure B, table 9:* Yield: Cu: 96 mg (41 %), Fe: n.a.; shining yellow powder; M.p.: 212-213 °C; TLC: R_f(PE:EtOAc=5:1) = 0.38; GC/MS (EI+): m/z (rel. Intensity): 445 (M^+-100, 22), 444 (35), 443 (35), 442 (100), 428 (12), 396 (19), 323 (13), 294 (17), 267 (14), 253 (11), 197 (41), 191 (56), 190 (68), 177 (33), 132 (40), 131 (34), 130 (60), 103 (21), 96 (38), 77 (35), 73 (19); HRMS (ESI^+): exact mass calculated for $C_{34}H_{31}N_3O_4$: 568.2207. Found: 568.2216 $[M+Na]^+$; ^1H NMR (200 MHz, *$CDCl_3$*): δ = 1.40 (s, 9H), 2.54 (dd, ^2J = 16.6, ^3J = 3.2 Hz, 1H), 2.98 (ddd, ^2J = 16.7 Hz, ^3J = 12.4 Hz, ^3J = 6.3 Hz, 1H), 3.35 (dt, ^2J = 13.2 Hz, ^3J = 13.0 Hz, ^3J = 4.1 Hz, 1H), 4.12 (ddd, J = 13.6 Hz, ^3J = 4.9 Hz, ^3J = 1.1 Hz, 1H), 6.76 (s, 1H), 6.87-7.35 (m, 14H), 7.59 (dd, ^3J = 7.4 Hz, ^4J = 1.2 Hz, 1H), 7.78 (dd, ^4J = 2.3 Hz, ^4J = 1.5 Hz, 1H), 7.94 (ddd, ^3J = 7.9 Hz, ^4J = 2.0 Hz, ^4J = 1.5 Hz, 1H); ^{13}C NMR (50 MHz, *$CDCl_3$*): δ = 28.0 (t), 28.4 (q, 3C), 38.4 (t), 51.0 (d), 79.8 (s), 110.5 (d), 117.3 (s), 120.5 (d), 120.8 (d), 122.4 (d), 123.0 (d), 126.1 (d), 126.3 (d), 126.6 (d), 127.5 (s), 127.6 (d), 128.2 (d), 128.3 (d), 128.4 (d, 2C), 128.7 (d), 129.2 (d, 2C), 133.7 (s), 134.6 (s, 2C overlapping), 136.0 (s), 136.6 (s), 137.1 (d), 137.5 (s), 147.1 (s), 154.2 (s)

1,1-Dimethylethyl 1-[2-(naphth-1-yl)-1-phenyl-1*H*-indol-3-yl]-3,4-dihydroisoquinoline-2(1*H*)-carboxylate (1i). Prepared according to *general procedure B, table 9:* Yield: Cu: 70 mg (30 %); white powder; M.p.: 165-167 °C; TLC: R_f(PE:EtOAc=5:1) = 0.49; GC/MS (EI+): m/z (rel. Intensity): 450 (M^+-100, 70), 449 (100), 433 (15), 345 (14), 330 (26), 321 (17), 294 (12), 253 (19), 225 (28), 217 (17), 191 (22), 177 (18), 165 (19), 133 (30), 132 (28), 131 (24), 130 (62), 105 (14), 103 (24), 96 (40), 77 (32); HRMS (ESI^+): exact mass calculated for $C_{38}H_{34}N_2O_2$: 573.2513. Found: 573.2516 $[M+Na]^+$; ^{1}H NMR (200 MHz, *acetone-d$_6$*): δ = 1.01 (s, 9H), 2.67 (ddd, ^2J = 16.9 Hz, ^3J = 4.4 Hz, ^3J = 1.5 Hz, 1H), 2.83-3.00 (m, 1H), 3.48 (dt, ^2J = 13.6 Hz, ^3J = 4.4 Hz, 1H), 4.04 (dd, ^2J = 13.5 Hz, ^3J = 6.2 Hz, 1H), 6.64 (s, 1H), 6.86-7.51 (m, 17H), 7.66-7.83 (m, 3H); ^{13}C NMR (50 MHz, *acetone-d$_6$*): δ = 29.4 (q, 3C), 29.5 (t), 40.4 (t), 53.2 (d), 80.4 (s), 112.2 (d), 119.6 (s), 121.8 (d), 122.2 (d), 123.9 (d), 126.8 (d), 127.55 (d), 127.61 (d), 127.8 (d), 128.3 (d), 128.4 (d), 129.2 (d), 129.8 (d, 3C overlapping), 130.1 (d), 130.6 (d, 2C), 130.67 (d), 130.74 (d), 133.1 (d), 135.1 (s), 135.2 (s), 136.7 (s), 139.2 (s), 139.4 (s), 139.5 (s), 139.7 (s), 154.6 (s); (2 quaternary C`s missing due to potential overlap of signals)

1,1-Dimethylethyl 1-[2-(2-methylphenyl)-1-phenyl-1*H*-indol-3-yl]-3,4-dihydroisoquinoline-2(1*H*)-carboxylate (1j). Prepared according to *general procedure B, table 9:* Yield: Cu: 81 mg (37 %); white powder; M.p.: 87-90 °C; TLC: R_f(PE:EtOAc=5:1)=0.57; GC/MS (EI+): m/z (rel. Intensity): 414 (M^+-100, 79), 413 (100), 399 (49), 397 (48), 384 (12), 323 (26), 295 (31), 294 (45), 217 (17), 216 (14), 191 (17), 178 (20), 165 (18), 152 (12), 132 (39), 131 (27), 130 (64), 117 (11), 105 (16), 103 (20), 91 (11), 77 (31); HRMS (ESI^+): exact mass calculated for $C_{35}H_{34}N_2O_2$: 515.2693. Found: 515.2692 $[M+H]^+$; ^{1}H NMR (200 MHz, *CDCl$_3$*): δ = 1.30 (s, 9H), 2.00 (s, 3H), 2.46-2.70 (m, 1H), 2.78-3.12 (m, 2H), 3.91-4.09 (m, 1H), 6.48 (s, 1H), 6.85-7.33 (m, 17H); ^{13}C NMR (50 MHz, *CDCl$_3$*): δ = 19.9 (q), 28.3 (q, 3C), 28.4 (t), 38.7 (t), 51.3 (d), 79.4 (s), 110.4 (d), 116.8 (s), 119.7 (d), 120.3 (d), 121.8 (d), 125.0 (d), 126.0 (d), 126.4 (d), 126.9 (d), 127.7 (d, 2C), 128.1 (d), 128.2 (s), 128.5 (d), 128.7 (d, 3C overlapping) 129.5 (d), 131.4 (s), 132.5 (d), 134.9 (s), 136.8 (s), 137.6 (s, 2C overlapping), 137.9 (s), 138.4 (s), 154.4 (s).

5-(1*H*-Indol-1-yl)-2-phenyloxazole (5m, see table 6). Indole (47 mg, 0.400 mmol, 1 equiv.), CuI (7.6 mg, 40 µmol, 0.1 equiv.), and K_3PO_4 (171 mg, 0.800 mmol, 2 equiv.) were placed in an oven-dried tube, and 5-iodo-2-phenyloxazole (163 mg, 0.600 mmol, 1.5 equiv.) and DMEDA (9 µL, 80 µmol, 0.2 equiv.) were added under an argon atmosphere, followed by dry toluene (400 µL). The tube was sealed under argon, and the mixture was heated up to 135 °C and stirred at this temperature for 12 hours. The reaction mixture was cooled to room temperature, diluted with DCM and directly subjected to column chromatography using PE:EtOAc =100:0 → 90:10 (20 minutes) to afford the desired product. Yield: 71 mg (68 %); colorless solid; M.p.: 131-133 °C; TLC: Rf(PE:EtOAc=10:1) = 0.38; GC/MS (EI+): m/z (rel. Intensity): 260 (M+, 57), 231 (10), 144 (53), 116 (100), 89 (48), 63 (14); ^{1}H NMR (200 MHz, $CDCl_3$ δ = 6.64 (dd, 3J = 3.4 Hz, 4J = 0.6 Hz, 1H), 7.02 (s, 1H), 7.09-7.25 (m, 2H), 7.28 (d, 3J = 3.5 Hz, 1H), 7.32-7.43 (m, 3H), 7.52 (d, 3J = 7.8 Hz, 1H), 7.58 (dd, 3J = 7.2 Hz, 4J = 1.2 Hz, 1H), 7.91-8.03 (m, 2H); ^{13}C NMR (50 MHz, $CDCl_3$ δ = 106.0 (d), 111.0 (d), 114.8 (d), 121.3 (d), 121.7 (d), 123.6 (d), 126.0 (d, 2C), 126.3 (d), 127.0 (s), 128.9 (d, 2C), 129.1 (s), 130.4 (d), 135.6 (s), 145.4 (s), 157.5 (s).

General procedure C: C2-Arylation of 4a towards 6a-c (see table 2), 1-(Indol-3-yl)-N-Boc-THIQ **4a** (200 mg, 0.574 mmol, 1.0 equiv.), aryl boronic acid (288 mg, 0.861 mmol, 1.5 equiv.), $Cu(OAc)_2$ (104 mg, 0.574 mmol) and $Pd(OAc)_2$ (6.4 mg, 28.7 µmol, 0.05 equiv.) were placed in a 5 ml glass vial. After AcOH (2.0 mL) was added by syringe, the resulting solution was purged with oxygen for 30 seconds and stirred for 12 hours at room temperature in an oxygen atmosphere. Then, another 1.5 equiv. of

phenylboronic acid was added and the reaction stirred for another 12 hours. The black solution was poured onto ice-cold saturated sodium carbonate and extracted four times with EtOAc. The collected organic layers were washed twice with brine, dried over sodium sulfate, filtered and evaporated. The crude products **3a-c** were purified by flash chromatography, using gradient elution with PE:EtOAc=100:0 → 80:20 (45 minutes).

General procedure D: 2-Arylindolation of N-Boc-THIQ 9 towards 6a-c (see table 1): A mixture of *N*-Boc-THIQ **9** (200 mg, 0.858 mmol, 1.0 equiv.), catalyst ($Cu(NO_3)_2$ x $3H_2O$: 10.4 mg, 42.9 µmol, 0.05 equiv.; $FeCl_3$: 7.0 mg, 42.9 µmol, 0.05 equiv.), and 2-arylindole **3** (1.03 mmol, 1.2 equiv.) were placed into a 5 mL glass vial. Then, *t*BHP (223 µL, 1.12 mmol in decane) was added dropwise at 0 °C. The reaction mixture was capped and stirred for 10 minutes, keeping the temperature constant using a cryostat. Then the reaction mixture was slowly heated to 50 °C and stirred for 48 hours. The reaction mixture was cooled to room temperature, diluted with DCM and directly subjected to column chromatography using PE:EtOAc =100:0 → 0:40 (60 minutes) to afford the desired products **6a-c**.

1,1-Dimethylethyl 1-(2-phenyl-1*H*-indol-3-yl)-3,4-dihydroisoquinoline-2(1*H*)-carboxylate (6a). Yield: General procedure D: Fe: 205 mg (56 % table 1, Cu: 159 mg (44 %, table 1), General procedure C: 125 mg (51 %, table 2); colorless solid; M.p.: 89-92 °C; TLC: R_f(PE:EtOAc=5:1)=0.40; GC/MS (EI+): m/z (rel. Intensity): 324 (M^+-100, 58), 323 (100), 321 (11), 308 (20), 294 (12), 247 (20), 219 (27), 218 (24), 217 (16), 204 (10), 193 (42), 165 (14), 147 (20), 139 (11), 132 (18), 131 (15), 130 (30), 103 (14), 77 (13); HRMS (ESI^+): exact mass calculated for $C_{28}H_{28}N_2O_2$: 447.2043. Found: 447.2060 $[M+Na]^+$; ^1H NMR (200 MHz, *CDCl₃*): δ = 1.15 (s, 9H), 2.54 (dd, ^2J = 16.3 Hz, ^3J = 2.2 Hz, 1H), 2.91 (ddd, ^2J = 16.6 Hz, ^3J = 12.1 Hz, ^3J = 6.0 Hz, 1H), 3.26 (dt, ^2J = 13.1 Hz, ^3J = 12.8 Hz, ^3J = 3.9 Hz, 1H), 4.02 (dd, ^2J = 13.4 Hz, ^3J = 5.5 Hz, 1H), 6.58 (s, 1H), 6.77-7.28 (m, 13H), 8.33 (s, 1H); ^{13}C NMR (50 MHz, *$CDCl_3$*): δ = 28.2 (q, 3C), 28.3 (t), 38.8 (t), 51.1 (d), 79.6 (s), 110.7 (d), 114.2 (s), 119.7 (d), 120.1 (d), 121.7 (d), 126.0 (d), 126.4 (d), 127.9 (d), 128.1 (d), 128.2 (d, 2C), 128.7 (d), 129.3 (d, 2C), 133.2 (s), 134.8 (s), 135.4 (s), 137.0 (s), 137.3 (s), 154.5 (s); (1 C missing due to potential overlap of signals); ^1H NMR (200 MHz, *acetone-D6*): δ = 1.31 (s, 9H), 2.79 (ddd, ^2J = 16.4 Hz, ^3J = 4.0 Hz, ^3J = 1.4 Hz, 1H), 3.05 (ddd, ^2J = 16.7 Hz, ^3J = 12.0 Hz, ^3J = 6.1 Hz, 1H), 3.50 (ddd, ^2J = 13.4 Hz, ^3J = 12.2 Hz, ^3J = 4.2 Hz, 1H), 4.16 (ddd, ^2J = 13.5 Hz, ^3J = 4.7 Hz, ^3J = 1.2 Hz, 1H), 6.72 (s, 1H), 6.90-6.78 (m, 1H), 6.95-7.66 (m, 12H), 10.44 (s, 1H); ^{13}C NMR (50 MHz, *acetone-D6*): δ = 29.4 (q, 3C), 30.0 (t, overlaps with acetone signal), 40.8 (t), 53.2 (d), 80.7 (s), 112.9 (d), 115.5 (s), 121.1 (d), 121.8 (d), 123.2 (d), 127.8 (d), 128.3 (d), 129.6 (d), 129.8 (d), 130.2 (d, 2C), 130.7 (d), 131.3 (d, 2C), 135.3 (s), 136.8 (s), 138.1 (s), 139.1 (s), 139.6 (s), 156.1 (s); (1 C missing due to potential overlap of signals)

1,1-Dimethylethyl 1-(2-(4-methylphenyl)-1*H*-indol-3-yl)-3,4-dihydroisoquinoline-2(1*H*)-carboxylate (6b). Yield: General procedure D: Fe: 74 mg (20 %, table 1), Cu: n.a., General procedure C: 123 mg (49 %, table 2); white powder; M.p.: 79-82 °C; TLC: R_f (PE:EtOAc=5:1) = 0.41; GC/MS (EI+): m/z (rel. Intensity): 338 (M^+-100, 62), 337 (100), 336 (11), 335 (29), 322 (26), 308 (11), 247 (25), 233 (24), 218 (33), 217 (21), 191 (10), 169 (34), 160 (21), 153 (22), 146 (26), 139 (17), 132 (25), 131 (24), 130 (48), 103 (23), 96 (11), 89 (11), 77 (15); HRMS (ESI^+): exact mass calculated for $C_{29}H_{30}N_2O_2$: 461.2199. Found: 461.2206 $[M+Na]^+$; ^1H NMR (200 MHz, *$CDCl_3$*): δ = 1.29 (s, 9H), 2.38 (s, 3H), 2.70 (ddd, ^2J = 16.5 Hz, ^3J = 3.9 Hz, ^3J = 1.4 Hz, 1H), 3.06 (ddd, ^2J = 17.0 Hz, ^3J = 12.1 Hz, ^3J = 6.1 Hz, 1H), 3.40 (ddd, ^2J = 13.4 Hz, ^3J = 12.3 Hz, ^3J = 4.0 Hz, 1H), 4.07-4.23 (m, 1H), 6.67 (s, 1H), 6.89-6.99 (m, 1H), 7.03-7.21 (m, 8H), 7.27-7.34 (m, 3H), 8.13 (bs, 1H); ^1H NMR (200 MHz, *acetone-D6*): δ = 1.30 (s, 9H), 2.38 (s, 3H),

2.74-2.89 (m, 1H), 3.05 (ddd, ^2J = 16.4 Hz, ^3J = 12.0 Hz, ^3J = 6.0 Hz, 1H), 3.50 (ddd, ^2J = 13.5 Hz, ^3J = 12.0 Hz, ^3J = 4.3 Hz, 1H), 4.15 (ddd, ^2J = 13.5 Hz, ^3J = 4.6 Hz, ^3J = 1.2 Hz, 1H), 6.67 (s, 1H), 6.76-6.86 (m, 1H), 6.95-7.20 (m, 6H), 7.25 (d, ^3J = 8.0 Hz, 2H), 7.37 (dd, ^3J = 7.6 Hz, ^4J = 1.5 Hz, 1H), 7.49 (d, ^3J = 8.1 Hz, 2H), 10.38 (bs, 1H); ^{13}C NMR (50 MHz, *CDCl$_3$*): δ = 21.1 (q), 28.2 (q, 3C), 28.4 (t), 38.9 (t), 51.2 (d), 79.5 (s), 110.7 (d), 114.0 (s), 119.6 (d), 120.0 (d), 121.6 (d), 126.1 (d), 126.3 (d), 128.1 (d), 128.4 (s), 128.7 (d), 129.0 (d, 2C), 129.2 (d, 2C), 130.3 (s), 134.9 (s), 135.4 (s), 137.1 (s), 137.4 (s), 137.7 (s), 154.6 (s)

1,1-Dimethylethyl 1-(2-(4-methoxyphenyl)-1*H*-indol-3-yl)-3,4-dihydroisoquinoline-2(1*H*)-carboxylate (6c). Yield: General procedure D: Fe: 55 mg (14 %, table 1), Cu: n.a., General procedure C: 88 mg (34 %, table 2); Off colorless solid; M.p.: 76-78 °C; TLC: R_f (PE:EtOAc=5:1) = 0.32; GC/MS (EI$^+$): m/z (rel. Intensity): 354 (M$^+$-100, 37), 353 (62), 352 (35), 351 (100), 338 (20), 308 (12), 249 (15), 223 (34), 218 (20), 217 (17), 208 (42), 191 (18), 177 (28), 176 (35), 169 (19), 168 (22), 161 (18), 153 (31), 146 (28), 132 (28), 131 (25), 130 (42), 126 (13), 103 (18), 96 (31), 77 (20), 73 (12); HRMS (ESI$^+$): exact mass calculated for $C_{29}H_{30}N_2O_3$: 477.2149. Found: 477.2158 [M+Na]$^+$; ^{1}H NMR (200 MHz, *CDCl$_3$*): δ = 1.28 (s, 9H), 2.64 (ddd, ^2J = 16.4 Hz, ^3J = 3.8 Hz, ^3J = 1.5 Hz, 1H), 3.01 (ddd, ^2J = 16.6 Hz, ^3J = 12.1 Hz, ^3J = 6.0 Hz, 1H), 3.35 (dt, ^2J = 13.2 Hz, ^3J = 12.8 Hz, ^3J = 3.9 Hz, 1H), 3.76 (s, 3H), 4.03-4.18 (m, 1H), 6.64 (s, 1H), 6.80 (d, ^3J = 8.7 Hz, 2H), 6.92 (ddd, ^3J = 7.9 Hz, ^3J = 7.1 Hz, ^4J = 0.9 Hz, 1H), 7.19-6.98 (m, 6H), 7.29-7.19 (m, 3H), 8.28 (s, 1H); ^{1}H NMR (200 MHz, *acetone-D6*): δ = 1.32 (s, 9H), 2.87-2.72 (m, 1H), 3.05 (ddd, ^2J = 16.2 Hz, ^3J = 12.1 Hz, ^3J = 6.0 Hz, 1H), 3.49 (ddd, ^2J = 13.5 Hz, ^3J = 12.0 Hz, ^3J = 4.3 Hz, 1H), 3.85 (s, 3H), 4.14 (ddd, ^2J = 13.5 Hz, ^3J = 4.6 Hz, ^3J = 1.3 Hz, 1H), 6.81 (ddd, ^3J = 7.8 Hz, ^3J = 7.0 Hz, ^4J = 1.1 Hz), 6.98 (d, ^3J = 8.9 Hz, 2H), 7.01-7.29 (m, 6H), 7.32-7.40 (m, 1H), 7.50 (d, ^3J = 8.9 Hz, 2H), 10.34 (bs, 1H); ^{13}C NMR (50 MHz, *CDCl$_3$*): δ = 28.2 (q, 3C), 28.4 (t), 38.8 (t), 51.2 (d), 55.2 (q), 79.6 (s), 110.6 (d), 113.8 (d, 2C), 114.0 (s), 119.7 (d), 120.0 (d), 121.6 (d), 125.6 (s), 126.0 (d), 126.3 (d), 128.1 (d), 128.4 (s), 128.7 (d), 130.6 (d, 2C), 134.8 (s), 135.2 (s), 136.8 (s), 137.4 (s), 154.6 (s), 159.3 (s).

Phenylmethyl 1-(2-phenyl-1*H*-indol-3-yl)-3,4-dihydroisoquinoline-2(1*H*)-carboxylate (6g, table 2). 1-(Indol-3-yl)-N-Cbz-THIQ **4b** (245 mg, 0.641 mmol, 1.0 equiv.), phenylboronic acid (117 mg, 0.961 mmol, 1.5 equiv.), $Cu(OAc)_2$ (116 mg, 0.641 mmol) and $Pd(OAc)_2$ (7.2 mg, 32.0 µmol, 0.05 equiv.) were placed in a 5 ml glass vial. After acetic acid (4.0 mL) was added by syringe, the resulting solution was degassed twice and refilled with O_2 (1.0 atm.). The mixture was stirred for 12 h at room temperature. Then, another 1.5 equiv. of phenylboronic acid was added and the reaction stirred for another 12 hours. The reaction mixture was poured onto saturated sodium carbonate and extracted four times with EtOAc. The collected organic layers were washed once with saturated sodium carbonate, dried over sodium sulfate, filtered and evaporated. Flash chromatography using PE:Et_2O=100:0 → 0:50 (1h 30 minutes) afforded the desired product **6g**. Yield: 151 mg (51 %); colorless solid; M.p.: 76-78 °C; TLC: R_f(PE:EtOAc=5:1)=0.28; GC/MS (EI$^+$): m/z (rel. Intensity): 458 (M$^+$, 2), 367 (1), 324 (24), 323 (100), 218 (12), 217 (10), 193 (14), 165 (10), 130 (20), 103 (9), 91 (79), 77 (8), 65 (9); HRMS (ESI$^+$): exact mass calculated for $C_{31}H_{26}N_2O_2$: 481.1886. Found: 481.1891 [M+Na]$^+$; ^{1}H NMR (200 MHz, *CDCl$_3$*): δ = 2.69 (dd, ^2J = 16.3 Hz, ^3J = 1.8 Hz, 1H), 2.96 (ddd, ^2J = 16.3 Hz, ^3J = 12.3 Hz, ^3J = 5.7 Hz, 1H), 3.40 (dt, ^2J = 13.1 Hz, ^3J = 12.9 Hz, ^3J = 3.6 Hz, 1H), 4.26 (dd, ^2J = 13.3 Hz, ^3J = 5.0 Hz, 1H), 4.49 (bs, 1H), 4.93 (d, ^2J = 12.4 Hz, 1H), 6.71 (s, 1H), 6.88 (d, ^3J = 7.2 Hz, 1H), 6.94-7.26 (m, 15H), 7.28-7.41 (m, 2H), 8.44 (s, 1H); ^{13}C NMR (50 MHz, *CDCl$_3$*): δ = 28.8 (t), 39.2 (t), 51.1 (d), 66.6 (t), 110.8 (d), 113.7 (s), 119.8 (d), 120.1 (d), 121.8 (d), 126.3 (d), 126.5 (d), 127.6 (d), 127.7 (d, 2C), 127.85

(d), 127.90 (d), 128.1 (d, 2C), 128.3 (d, 2C), 128.7 (d), 128.9 (d, 2C), 133.2 (s), 134.6 (s), 135.6 (s), 136.6 (s), 136.8 (s), 137.4 (s), 155.3 (s); (1 C missing due to potential overlap of signals).

General procedure E: N-Arylation of 4 towards 7a-e,g (table 3). 1-(Indol-3-yl)-N-Boc-THIQ **4a** (100 mg, 0.287 mmol, 1.0 equiv.), iron(III)-chloride (4.7 mg, 28.7 µmol, 0.1 equiv.), and K_3PO_4 (122 mg, 0.574 mmol, 2.0 equiv.) were placed in a 2 mL glass vial, and aryliodide (0.431 mmol, 1.5 equiv.) and DMEDA (5.6 µL, 57.4 µmol, 0.20 equiv.) were added followed by dry toluene (300 µL). The reaction mixture was purged with argon for 30 seconds, the vial sealed and heated to 135 °C and stirred at this temperature for 24 hours. The black slurry was cooled down to rt, diluted with DCM and filtered through a plug of celite. The solvent of the filtrate was evaporated and the crude product directly subjected to column chromatography using gradient elution with PE:EtOAc=100:0 → 0:40 (50 minutes) to afford the desired products **7a-e,g**.

General procedure F for N-arylindolation of N-Boc-THIQ 9 (table 5). To a mixture of N-Boc-THIQ **5** (200 mg, 0.858 mmol, 1.0 equiv.), catalyst ($Cu(NO_3)_2$ x $3H_2O$: 10.4 mg, 42.9 µmol, 0.05 equiv.; $FeCl_3$: 7.0 mg, 42.9 µmol, 0.05 equiv.), and N-Arylindole **5a** (1.03 mmol, 1.2 equiv.) placed into a 5 mL glass vial, tert-butyl hydroperoxide (223 µL, 1.11 mmol in decane, 1.3 equiv.) was added dropwise at 0 °C. The reaction mixture was capped and stirred for 10 minutes at 0 °C and then slowly heated to 50 °C and stirred for 24 hours, unless stated differently. The dark brown slurry was cooled down to rt, diluted with DCM and directly subjected to column chromatography using gradient elution with PE:EtOAc=100:0 → 0:30 (75 minutes) to afford the desired product **7**.

1,1-Dimethylethyl 1-(1-phenyl-1*H*-indol-3-yl)-3,4-dihydroisoquinoline-2(1*H*)-carboxylate (7a). Yield: General procedure F: Fe: 178 mg (49 %), Cu: 303 mg (83 %), General procedure E: 351 mg (72 %, table 3); colorless solid; M.p.: 123-125 °C; TLC: R_f(PE:EtOAc=5:1)=0.57; GC/MS (EI^+): m/z (rel. Intensity): 324 (M^+-100, 62), 323 (100), 295 (18), 294 (27), 232 (13), 219 (10), 217 (13), 216 (12), 193 (16), 165 (10), 162 (14), 132 (13), 131 (22), 130 (41), 103 (12), 77 (16); HRMS (ESI^+): exact mass calculated for $C_{23}H_{18}N_2$: 323.1543. Found: 323.1534. $[M-C_5H_{10}O_2+H]^+$; ^{1}H NMR (200 MHz, *CDCl₃*): δ = 1.56 (s, 9H), 2.76 (dd, ^2J = 16.5 Hz, ^3J = 2.4 Hz, 1H), 2.94-3.35 (m, 2H), 4.08 (bs, 1H), 6.74 (s, 1H), 6.82 (bs, 1H), 7.12-7.24 (m, 6H), 7.27-7.51 (m, 5H), 7.55 (dd, ^3J = 7.1 Hz, ^4J = 1.5 Hz, 1H), 7.93 (bs, 1H); ^{13}C NMR (50 MHz, APT, *$CDCl_3$*): δ = 28.4 (t), 28.5 (q, 3C), 37.7 (t), 50.3 (d), 79.7 (s), 110.4 (d), 120.1 (s), 120.3 (d), 120.7 (d), 122.7 (d), 124.2 (d, 2C), 125.7 (d), 126.4 (d), 126.7 (d), 127.9 (s), 128.4 (d, 2C overlapping), 129.1 (d), 129.5 (d, 2C), 135.0 (s), 136.0 (s), 136.3 (s), 139.4 (s), 154.2 (s)

1,1-Dimethylethyl 1-[1-(4-methoxyphenyl)-1*H*-indol-3-yl]-3,4-dihydroisoquinoline-2(1*H*)-carboxylate (7b). Yield: General procedure F: Fe: 155 mg (40 %), Cu: 270 mg (69 %), General procedure E: 89 mg (68 %, table 3); colorless solid; M.p.: 71-72 °C; TLC: R_f(PE:EtOAc=5:1)=0.48; GC/MS (EI^+): m/z (rel. Intensity): 354 (M^+-100, 69), 353 (100), 325 (18), 324 (20), 232 (12), 216 (21), 177 (26), 133 (22), 132 (24), 131 (36), 130 (58), 77 (21); HRMS (ESI^+): exact mass calculated for $C_{29}H_{30}N_2O_3$: 455.2329. Found: 455.2340. $[M+H]^+$; ^{1}H NMR (200 MHz, *$CDCl_3$*): δ = 1.54 (s, 9H), 2.74 (dd, ^2J = 16.4 Hz, ^3J = 2.3 Hz, 1H), 2.93-3.32 (m, 2H), 3.84 (s, 3H), 4.05 (bs, 1H), 6.66 (s, 1H, H1), 6.80 (bs, 1H), 6.96 (d, ^3J = 8.9 Hz, 2H), 7.08-7.26 (m, 6H), 7.29 (d, ^3J = 8.9 Hz, 2H), 7.42 (dd, ^3J = 7.2 Hz, ^4J = 1.4 Hz, 1H), 7.88 (bs, 1H); ^{13}C NMR (50 MHz, APT, *$CDCl_3$*): δ = 28.4 (t), 28.6 (q, 3C), 37.6 (t), 50.4 (d), 55.5 (q), 79.6 (s), 110.3 (d), 114.6 (d, 2C), 119.4 (s), 120.0 (d), 120.6 (d), 122.5 (d), 125.7 (d), 125.8 (d, 2C),

126.6 (d), 127.6 (s), 128.4 (d), 128.7 (d), 129.0 (d), 132.4 (s), 135.0 (s), 136.2 (s), 136.7 (s), 154.2 (s), 158.1 (s)

1,1-Dimethylethyl 1-[1-(thiophen-2-yl)-1*H*-indol-3-yl]-3,4-dihydroisoquinoline-2(1*H*)-carboxylate (7c). Yield: General procedure F: Cu: 286 mg (78 %), General procedure E: 105 mg (85 %, table 3); colorless solid; M.p.: 69-71 °C; TLC: R_f(PE:EtOAc=5:1)=0.53; GC/MS (EI^+): m/z (rel. Intensity): 330 (M^+, 67), 329 (100), 301 (18), 300 (13), 232 (16), 217 (11), 216 (17), 199 (22), 198 (14), 165 (28), 148 (15), 133 (77), 132 (37), 131 (34), 130 (70), 103 (22), 77 (20); HRMS (ESI^+): exact mass calculated for $C_{26}H_{26}N_2O_2S$: 453.1607. Found: 453.1624 $[M+Na]^+$; ^1H NMR (200 MHz, *acetone-D$_6$*): δ = 1.53 (s, 9H), 2.78 (ddd, ^2J = 16.3 Hz, ^3J = 4.1 Hz, ^3J = 2.2 Hz, 1H), 2.99 (ddd, ^2J = 16.2 Hz, ^3J = 11.8 Hz, ^3J = 5.7 Hz, 1H), 3.23 (ddd, ^2J = 13.1 Hz, ^3J = 11.8 Hz, ^3J = 4.3 Hz, 1H), 4.07 (d, ^2J = 13.1 Hz, 1H), 6.73 (s, 1H), 6.79 (bs, 1H), 7.00-7.35 (m, 9H), 7.55 (dd, ^3J = 7.8 Hz, ^4J = 1.1 Hz, 1H), 7.86 (bs, 1H); ^{13}C NMR (50 MHz, APT, *acetone-D$_6$*): δ = 29.7 (q, 3C), 30.0 (t), 39.2 (t), 52.3 (d), 81.1 (s), 112.3 (d), 122.3 (d, 2C overlapping), 122.6 (d), 122.7 (s), 123.7 (d), 125.1 (d), 127.6 (d), 128.1 (d), 128.7 (d), 129.7 (s), 130.0 (d), 131.0 (d), 131.1 (d), 136.9 (s), 137.6 (s), 139.3 (s), 142.7 (s), 155.8 (s)

1,1-Dimethylethyl 1-[1-(4-fluorophenyl)-1*H*-indol-3-yl]-3,4-dihydroisoquinoline-2(1*H*)-carboxylate (7d). Yield: General procedure F: Cu: 245 mg (65 %), General procedure E: 107 mg (84 %, table 3); colorless solid; M.p.: 122-124 °C; TLC: R_f(PE:EtOAc=5:1)=0.54; GC/MS (EI^+): m/z (rel. Intensity): 342 (M^+-100, 69), 341 (100), 313 (21), 312 (28), 232 814), 217 (16), 216 (17), 171 (31), 132 (32), 131 (48), 130 (82), 117 (10), 115 (11), 103 (22), 95 (14), 77 (16); HRMS (ESI^+): exact mass calculated for $C_{28}H_{27}FN_2O_2$: 443.2129. Found: 443.2143 $[M+H]^+$; ^1H NMR (200 MHz, *CDCl$_3$*): δ = 1.44 (s, 9H), 2.63 (dd, ^2J = 16.4 Hz, ^3J = 2.2 Hz, 1H), 2.81-3.22 (m, 2H), 3.97 (bs, 1H), 6.58 (s, 1H), 6.70 (bs, 1H), 6.94-7.18 (m, 8H), 7.23 (dd, ^3J = 8.9 Hz, ^4J = 4.8 Hz, 2H), 7.32 (dd, ^3J = 7.1 Hz, ^4J = 1.6 Hz, 1H), 7.81 (bs, 1H); ^{13}C NMR (50 MHz, APT, *CDCl$_3$*): δ = 28.4 (t), 28.5 (q, 3C), 37.6 (t), 50.4 (d), 79.7 (s), 110.1 (d), 116.3 (d, 2C, d, $^2J_{CF}$ = 22.8 Hz), 120.1 (s), 120.4 (d), 120.8 (d), 122.8 (d), 125.7 (d), 126.0 (d, 2C, d, $^3J_{CF}$ = 8.4 Hz), 126.7 (d), 127.8 (s), 128.4 (d, 2C overlapping), 129.1 (d), 135.0 (s), 135.5 (s, d, $^4J_{CF}$ = 3.0 Hz), 136.0 (s), 136.5 (s), 154.2 (s), 160.9 (s, d, $^1J_{CF}$ = 246 Hz)

1,1-Dimethylethyl 1-[1-(4-nitrophenyl)-1*H*-indol-3-yl]-3,4-dihydroisoquinoline-2(1*H*)-carboxylate (7e). To a mixture of N-Boc-THIQ **9** (200 mg, 0.858 mmol, 1.0 equiv.), catalyst (10.4 mg, 42.9 µmol, 0.05 equiv.), and 1-(4-nitrophenyl)indole **5e** (245 mg, 1.03 mmol, 1.2 equiv.) placed into a 5 mL glass vial, tBHP (223 µL, 1.11 mmol in decane) was added dropwise at 0 °C. The reaction mixture was capped and stirred for 10 minutes, keeping the temperature constant using a cryostat. Then the reaction mixture was slowly heated up to 50 °C and stirred for 24 hours. No conversion could be detected by GC/MS. Thus, another 5 mol% catalyst and 1.3 equiv. of tBHP was added at 0 °C. The reaction mixture was slowly heated to 80 °C and stirred for another 24 hours under air. Workup and purification was carried out according to the general procedure (General procedure F). Yield: General procedure F Cu: 179 mg (45 %, table 5), General procedure E: 68 mg (50 %, table 3); shining yellow powder; M.p.: 97-99 °C; TLC: R_f(PE:EtOAc=5:1)=0.30; GC/MS (EI^+): m/z (rel. Intensity): 369 (M^+-100, 58), 368 (98), 340 (21), 339 (29), 336 (22), 322 (20), 293 (16), 232 (22), 217 (21), 216 (24), 191 (31), 145 (34), 132 (59), 131 (82), 130 (100), 117 (18), 103 (29), 96 (23), 77 (22); HRMS (ESI^+): exact mass calculated for $C_{23}H_{19}N_3O_2$: 370.1556. Found: 370.1544. $[M-C_5H_{10}O_2+H]^+$; ^1H NMR (200 MHz, $CDCl_3$): δ = 1.54 (s, 9H), 2.76 (d, ^2J = 14.0 Hz, 1H), 2.95-3.29 (m, 2H), 4.10 (bs, 1H), 6.77 (bs, 2H), 7.14-7.36 (m, 6H), 7.56 (d, ^3J = 9.0 Hz, 2H), 7.61 (dd, ^3J = 7.6 Hz, ^4J = 1.4 Hz, 1H), 7.92 (bs, 1H), 8.32 (d, ^3J = 8.9 Hz,

2H); ^{13}C NMR (50 MHz, *CDCl$_3$*): δ = 28.4 (t), 28.5 (q, 3C), 37.8 (t), 50.3 (d), 80.0 (s), 110.4 (d), 121.2 (d), 121.6 (d), 122.7 (s), 123.2 (d, 2C), 123.7 (d), 125.4 (d, 2C), 125.9 (d), 126.9 (d), 127.3 (d), 128.3 (d), 128.8 (s), 129.2 (d), 135.0 (s), 135.5 (s), 135.7 (s), 144.9 (s, 2C overlapping), 154.2 (s)

Phenylmethyl 1-(1-phenyl-1*H*-indol-3-yl)-3,4-dihydroisoquinoline-2(1*H*)-carboxylate (7g, table 3). N-Cbz-THIQ-Ind **4b** (110 mg, 0.287 mmol, 1.0 equiv.), iron(III)-chloride (4.7 mg, 28.7 µmol, 0.10 equiv.), and K_3PO_4 (122 mg, 0.574 mmol, 2.0 equiv.) were placed in an oven-dried tube, and 2-iodothiophene (90.5 mg, 0.431 mmol, 1.5 equiv.) and DMEDA (5.6 µL, 57.4 µmol, 0.20 equiv.) were added under an argon atmosphere, followed by dry toluene (300 µL). The tube was sealed under argon, and the mixture was heated up to 135 °C and stirred at this temperature for 48 hours. Yield: 102 mg (77 %); off colorless solid; M.p.: 58-60 °C; TLC: R_f(PE:EtOAc=5:1)=0.43; GC/MS (EI+): m/z (rel. Intensity): 458 (M+, 3), 367 (5), 324 (24), 323 (100), 294 (10), 193 (10), 130 (23), 103 (12), 91 (68), 77 (12); HRMS (ESI$^+$): exact mass calculated for $C_{31}H_{26}N_2O_2$: 481.1886. Found: 481.1892 [M+Na]$^+$; ^{1}H NMR (200 MHz, *CDCl$_3$*): δ = 2.78 (dd, ^2J = 16.3 Hz, ^3J = 3.3 Hz, 1H), 3.06 (bs, 1H), 3.31 (dt, ^2J = 12.6 Hz, ^3J = 3.7 Hz, 1H), 4.17 (bs, 1H), 5.08-5.52 (m, 2H), 6.75 (s, 1H), 6.86 (bs, 1H), 7.08-7.62 (m, 17H), 7.88 (bs, 1H); ^{13}C NMR (50 MHz, APT, *CDCl$_3$*): δ = 28.6 (t), 37.6 (t), 51.3 (d), 67.1 (t), 110.4 (d), 119.6 (s), 120.5 (d), 120.7 (d), 122.7 (d), 124.2 (d, 2C), 125.9 (d), 126.4 (d), 126.8 (d), 128.0 (d, 2C overlapping), 128.5 (d, 3C overlapping), 129.0 (d), 129.5 (d, 2C), 134.6 (s), 135.7 (s, 2C overlapping), 136.2 (s), 136.7 (s), 139.4 (s), 154.9 (s); ^{1}H NMR (200 MHz, *acetone-D$_6$*): δ = 2.81 (dd, ^{2}J = 16.0 Hz, ^{3}J = 2.2 Hz, 1H), 3.02 (ddd, ^{2}J = 16.6 Hz, ^{3}J = 11.9 Hz, ^{3}J = 5.9 Hz, 1H), 3.34 (ddd, ^{2}J = 13.3 Hz, ^{3}J = 11.8 Hz, ^{3}J = 4.4 Hz, 1H), 4.14 (d, ^{2}J = 13.3 Hz, 1H), 5.23 (s, 2H), 6.80 (bs, 1H), 6.88 (s, 1H), 6.97-7.95 (m, 18H); ^{13}C NMR (50 MHz, APT, *acetone-D$_6$*): δ = (CH_2-group at 28.6 ppm in $CDCl_3$ overlaps with acetone signal), 39.3 (t), 53.1 (d), 68.6 (t), 112.2 (d), 121.6 (s), 122.20 (d), 122.27 (d), 124.6 (d), 125.8 (d, 2C), 127.7 (d), 128.3 (d), 128.7 (d), 129.7 (d, 2C overlapping), 130.06 (d), 130.16 (d), 130.3 (d), 131.0 (d), 131.5 (d, 2C), 136.7 (s, 2C overlapping), 137.6 (s), 138.1 (s), 139.2 (s), 141.2 (s), 156.5 (s)

General procedure G: Regioselective N-arylation of 4d towards 7h-m (table 4). Substrate **4d** (100 mg, 0.805 mmol, 1.0 equiv.), copper(I)-iodide (3.9 mg, 20.1 µmol, 0.05 equiv.), and CsF (306 mg, 2.01 mmo, 2.5 equiv.) were placed in an oven-dried tube 2 mL glass vial, and aryliodide (0.604 mmol, 1.5 equiv.) and dmeda (4 µL, 40.3 µmol, 0.30 equiv.) were added, followed by dry acetonitrile (1 mL). The tube was degassed with argon for 30 seconds and stirred for 4 hours at room temperature. Since no significant product formation was observed by TLC, the reaction was heated up to 70 °C, and magnesiumsulfate (49 mg, 0.403 mmol, 0.50 equiv.) was added to trap traces of water and stirred for another 20 hours. The reaction mixture was poured onto saturated sodium carbonate and extracted three times with EtOAc. The collected organic layer was washed twice with brine, dried over sodium sulfate, filtered and evaporated. The crude products **7h-m** were purified by flash chromatography, using gradient elution with PE:EtOAc=100:0 → 0:100 (90 minutes).

1-(1-Phenyl-1*H*-indol-3-yl)-1,2,3,4-tetrahydroisoquinoline (7h, table 4). Yield: 95 mg (73 %); white powder; M.p.: 51-53 °C; TLC: R_f(EtOAc:MeOH=5:1) = 0.38; GC/MS (EI+): m/z (rel. Intensity): 324 (M+, 62), 323 (100), 295 (17), 294 (25), 232 (11), 217 (10), 193 (15), 162 (16), 132 (10), 131 (15), 130 (26), 77 (11); ^{1}H NMR (200 MHz, *CDCl$_3$*): δ = 2.39 (s, 1H), 2.77-3.37 (m, 4H), 5.55 (s, 1H), 7.00-7.36 (m, 8H), 7.41-7.49 (m, 4H), 7.52-7.60 (m, 2H); ^{13}C NMR (50 MHz, APT, *CDCl$_3$*): δ = 29.7 (t), 41.6 (t), 53.6 (d), 110.6 (d), 119.8 (d), 120.2 (d), 120.6 (s), 122.5 (d), 124.1 (d, 2C), 125.6 (d, C6), 126.19 (d), 126.22 (d), 127.4 (d), 127.8 (s), 127.9 (d), 129.0 (d), 129.5 (d, 2C), 135.2 (s), 136.4 (s), 137.9 (s), 139.5 (s)

1-(1-(4-Methoxyphenyl)-1*H*-indol-3-yl)-1,2,3,4-tetrahydroisoquinoline (7i, table 4). Yield: 74 mg (52 %); off colorless solid; M.p.: 49-51 °C; TLC: R_f (EtOAc:MeOH=10:1) = 0.11; GC/MS (EI+): m/z (rel. Intensity): 354 (M^+, 69), 353 (100), 337 (10), 325 (13), 324 (17), 232 (12), 216 (12), 177 (17), 133 (12), 132 (16), 131 (26); HRMS (ESI^+): exact mass calculated for $C_{24}H_{22}N_2O$: 355.1805. Found: 355.1791 $[M+H]^+$; ^1H NMR (200 MHz, *CDCl$_3$*): δ = 2.30 (bs, 1H), 2.78-3.41 (m, 4H), 3.86 (s, 3H), 5.56 (s, 1H), 6.94-7.24 (m, 9H), 7.37 (d, ^3J = 8.9 Hz, 2H), 7.46 (dd, ^3J = 8.0 Hz, ^4J = 1.0 Hz, 1H), 7.56 (dd, ^3J = 7.4 Hz, ^4J = 1.1 Hz, 1H); ^{13}C NMR (50 MHz, APT, *CDCl$_3$*): δ = 29.7 (t), 41.6 (t), 53.7 (d), 55.5 (q), 110.5 (d), 114.6 (d, 2C), 119.7 (d), 120.0 (d), 122.3 (d), 125.6 (d), 125.8 (d, 2C), 126.2 (d), 127.4 (s), 127.85 (d), 127.92 (d), 129.0 (d), 132.6 (s), 135.2 (s), 136.9 (s), 138.0 (s), 158.1 (s), (1 quaternary C missing due to potential overlap of signals)

1-(1-(Thiophen-2-yl)-1*H*-Indol-3-yl)-1,2,3,4-tetrahydroisoquinoline (7j, table 4). Yield: 105 mg (79 %); colorless solid; M.p.: 46-47 °C; TLC: R_f(EtOAc:MeOH=10:1) = 0.44; GC/MS (EI+): m/z (rel. Intensity): 330 (M^+, 88), 329 (100), 301 (17), 300 (11), 232 (14), 199 (12), 165 (12), 133 (28), 130 (30), 103 (10); HRMS (ESI^+): exact mass calculated for $C_{21}H_{18}N_2S$: 331.1263. Found: 331.1254 $[M+H]^+$; ^1H NMR (200 MHz, *CDCl$_3$*): δ = 2.05 (bs, 1H), 2.78-3.37 (m, 4H), 5.50 (s, 1H), 6.96-7.29 (m, 10H), 7.55 (t, ^3J = 7.9 Hz, 2H); ^{13}C NMR (50 MHz, APT, *CDCl$_3$*): δ = 29.7 (t), 41.8 (t), 53.7 (d), 110.8 (d), 119.9 (d), 120.1 (d), 120.7 (d), 121.3 (s), 121.4 (d), 123.0 (d), 125.6 (d), 125.9 (d), 126.2 (d), 127.6 (s), 127.8 (d), 128.6 (d), 129.0 (d), 135.2 (s), 137.7 (s), 137.8 (s), 141.5 (s)

1-(1-(4-Fluorophenyl)-1*H*-indol-3-yl)-1,2,3,4-tetrahydroisoquinoline (7k, table 4). Yield: 80 mg (58 %); off colorless solid; M.p.: 50-52 °C; TLC: R_f(EtOAc:EtOH=10:1) = 0.27; GC/MS (EI^+): m/z (rel. Intensity): 342 (M^+, 60), 341 (100), 313 (17), 312 (25), 232 (10), 211 (11), 171 (11), 132 (10), 131 (16), 130 (29); HRMS (ESI^+): exact mass calculated for $C_{23}H_{19}FN_2$: 343.1605. Found: 343.1595 $[M+H]^+$; ^1H NMR (200 MHz, *CDCl$_3$*): δ = 2.07 (bs, 1H), 2.79-3.39 (m, 4H), 5.56 (s, 1H), 7.00-7.24 (m, 9H), 7.36-7.51 (m, 3H), 7.59 (d, ^3J = 7.6 Hz, 1H); ^{13}C NMR (50 MHz, APT, *CDCl$_3$*): δ = 29.7 (t), 41.7 (t), 53.7 (d), 110.3 (d), 116.4 (d, *d,* $^2J_{CF}$ = 22.8 Hz), 119.9 (d), 120.3 (d), 120.8 (s), 122.7 (d), 125.6 (d), 126.0 (d, d, $^3J_{CF}$ = 8.4 Hz), 126.2 (d), 127.5 (d), 127.7 (s), 127.8 (d), 129.0 (d), 135.3 (s), 135.7 (s, d, $^4J_{CF}$ = 3.0 Hz), 136.6 (s), 138.0 (s), 160.9 (s, *d,* $^1J_{CF}$ = 246 Hz)

1-(1-(3-Nitrophenyl)-1*H*-indol-3-yl)-1,2,3,4-tetrahydroisoquinoline (7l, table 4). Yield: 110 mg (74 %); pale yellow solid; M.p.: 59-61 °C; TLC: R_f (EtOAc:EtOH=5:1) = 0.54; GC/MS (EI^+): m/z (rel. Intensity): 369 (M^+, 57), 368 (100), 340 (24), 339 (30), 322 (23), 321 (14), 232 (25), 217 (21), 161 (21), 147 (29), 146 (38), 132 (46), 131 (59), 130 (78), 103 (20); HRMS (ESI^+): exact mass calculated for $C_{23}H_{19}N_3O_2$: 370.1550. Found: 370.1542 $[M+H]^+$; ^1H NMR (200 MHz, *CDCl$_3$*): δ = 2.09 (bs, 1H), 2.82-3.39 (m, 4H), 5.57 (s, 1H), 7.02-7.09 (m, 2H), 7.13 (s, 1H), 7.14-7.35 (m, 4H), 7.55-7.65 (m, 2H), 7.67 (t, ^3J = 8.1 Hz, 1H), 7.83 (ddd, ^3J = 8.0 Hz, ^4J = 2.0 Hz, ^4J = 1.1 Hz, 1H), 8.17 (ddd, ^3J = 8.1 Hz, ^4J = 2.1 Hz, ^4J = 1.1 Hz, 1H), 8.36 (t, ^4J = 2.1 Hz, 1H); ^{13}C NMR (50 MHz, APT, *CDCl$_3$*): δ = 29.7 (t), 41.7 (t), 53.7 (d), 110.2 (d), 118.6 (d), 120.4 (d), 120.6 (d), 121.2 (d), 122.5 (s), 123.4 (d), 125.8 (d), 126.4 (d), 126.6 (d), 127.7 (d), 128.3 (s), 129.1 (d), 129.5 (d), 130.5 (d), 135.2 (s), 136.1 (s), 137.6 (s), 140.7 (s), 149.1 (s)

1-(1-(4-Ethoxycarbonylphenyl)-1*H*-indol-3-yl)-1,2,3,4-tetrahydroisoquinoline (7m, table 4). Yield: 101 mg (63 %); colorless solid; M.p.: 51-53 °C; TLC: R_f(EtOAc:EtOH=5:1) = 0.35; GC/MS (EI^+): m/z (rel. Intensity): 396 (M^+, 66), 395 (100), 367 (31), 366 (14), 232 (20), 175 (15), 160 (18), 132 (18), 131 (29), 130 (46), 103 (12); HRMS (ESI^+): exact mass calculated for $C_{26}H_{24}N_2O_2$: 397.1911 Found: 397.1910

$[M+H]^+$; ^{1}H NMR (200 MHz, *CDCl$_3$*): δ = 1.42 (t, ^{3}J = 7.1 Hz, 3H, CH_3), 1.92 (bs, 1H, 2-NH), 2.81-3.40 (m, 4H, H3 & H4), 4.41 (q, ^{3}J = 7.1 Hz, 2H, O-CH_2), 5.55 (s, 1H, H1), 7.02-7.31 (m, 7H), 7,55 (d, ^3J = 8.6 Hz, 2H, H2´´), 7.56-7.68 (m, 2H), 8.17 (d, ^3J = 8.6 Hz, 2H, H3´´); ^{13}C NMR (50 MHz, APT, *CDCl$_3$*): δ = 14.3 (q), 29.7 (t), 41.8 (t), 53.6 (d), 61.1 (t), 110.7 (d), 120.1 (d), 120.8 (d), 122.0 (s), 122.97 (d, 2C), 123.02 (d), 125.6 (d), 126.3 (d), 126.7 (d), 127.7 (d), 128.3 (s), 129.0 (d), 131.1 (d, 2C), 135.2 (s), 136.0 (s), 137.8 (s), 143.4 (s), 165.8 (s), one quartenary carbon overlapping.

General procedure H: Preparation of 2-aryl-1-phenylindoles 8a-q (table 6): 1-Arylindole **5** (1.50 mmol, 1.0 equiv.), arylboronic acid (2.25 mmol, 1.5 equiv.), and $Pd(OAc)_2$ (16.8 mg, 75 µmol, 0.05 equiv.) were placed in a 25 mL glass vial. After AcOH (10 mL) was added by syringe, the resulting solution was purged with O_2 (1.0 atm.) for 1 minute. The mixture was stirred for 12 h at room temperature in an oxygen atmosphere. Another 1.5 equiv. of arylboronic acid were added and the reaction mixture stirred until full consumption of 1-phenylindole starting material was observed (monitored by GC/MS and TLC), unless stated differently. The reaction mixture was poured onto ice-cold, aqueous 2N NaOH and extracted three times with EtOAc. The collected organic layers were washed once with brine, dried over sodium sulfate, filtered and evaporated. Unless indicated differently, the crude product mixture was subjected to flash column chromatography using gradient elution with PE:EtOAc (100:0 to 90:10) to afford the desired products **8a-h,j,k,n-q**.

1,2-Diphenyl-1*H*-indole (8a, table 6). yield: 388 mg (96 %); colorless solid; M.p.: 80-82 °C; (Lit.: M.p.: 78-80 °C); TLC: R_f (PE:EtOAc=10:1) = 0.73; GC/MS (EI+): m/z (rel. Intensity): 269 (M^+, 100), 268 (45), 267 (35), 266 (16), 165 (28), 133 (35), 132 (14), 127 (15), 121 (13); ^{1}H NMR (200 MHz, *CDCl$_3$*): δ = 6.78 (s, 1H), 6.99-7.46 (m, 13H), 7.60-7.72 (m, 1H); ^{13}C NMR (50 MHz, APT, *CDCl$_3$*): δ = 103.7 (d), 110.6 (d), 120.5 (d), 120.7 (d), 122.3 (d), 127.1 (d), 127.2 (d), 128.0 (d, 2C), 128.1 (d, 2C), 128.2 (s), 128.8 (d, 2C), 129.2 (d, 2C), 132.5 (s), 138.5 (s), 139.0 (s), 140.7 (s)

2-(4-Chlorophenyl)-1-phenyl-1*H*-indole (8b, table 6). yield: 51 mg (11 %); colorless solid; M.p.: 170-172 °C; (Lit.: M.p.: 174-176 °C); TLC: Rf(PE) = 0.19; GC/MS (EI+): m/z (rel. Intensity): 305 (M^++2, 30), 303 (M+,100), 267 (26), 266 (11), 165 (22), 134 (61), 132 (25), 121 (10); ^{1}H NMR (200 MHz, $CDCl_3$): δ = 6.80 (bs, 1H), 7.11-7.53 (m, 12H), 7.62-7.74 (m, 1H); ^{13}C NMR (50 MHz, APT, $CDCl_3$): δ = 104.0 (d), 110.6 (d), 120.6 (d), 120.8 (d), 122.6 (d), 127.4 (d), 128.0 (d, 2C), 128.1 (s), 128.4 (d, 2C), 129.4 (d, 2C), 130.0 (d, 2C), 131.0 (s), 133.3 (s), 138.2 (s), 139.1 (s), 139.4 (s)

2-(4-Methoxyphenyl)-1-phenyl-1*H*-indole (8c, table 6). yield: 424 mg (94 %); colorless solid; M.p.: 109-117 °C; (Lit.: M.p.: 119-121 °C); TLC: R_f(PE:EtOAc=10:1) = 0.54; GC/MS (EI+): m/z (rel. Intensity): 299 (M^+, 100), 284 (43), 256 (11), 255 (12), 254 (29), 150 (8), 127 (14); HRMS (ESI^+): exact mass calculated for $C_{21}H_{17}NO$: 300.1383. Found: 300.1384. $[M+H]^+$, ^{1}H NMR (200 MHz, *CDCl$_3$*): δ = 3.73 (s, 3H), 6.72 (bs, 1H), 6.75 (d, ^3J = 8.9 Hz, 2H), 7.10-7.45 (m, 10H), 7.59-7.71 (m, 1H); ^{13}C NMR (50 MHz, *CDCl$_3$*): δ = 55.1 (q), 102.7 (d), 110.5 (d), 113.6 (d, 2C), 120.2 (d), 120.6 (d), 122.0 (d), 125.0 (s), 127.1 (d), 128.0 (d, 2C), 128.3 (s), 129.2 (d, 2C), 130.1 (d, 2C), 138.5 (s), 138.8 (s), 140.6 (s), 158.9 (s)

2-(4-Methylphenyl)-1-phenyl-1*H*-indole (8d, table 6). yield: 371 mg (87 %); colorless needles; M.p.: 131-133 °C; TLC: R_f (PE:EtOAc=20:1) = 0.55; GC/MS (EI+): m/z (rel. Intensity): 283 (M^+, 100), 277 (18), 165 (12), 140 (11), 133 (24), 132 (10); HRMS (ESI+): exact mass calculated for $C_{21}H_{17}N$: 284.1434. Found: 284.1433 $[M+H]^+$; ^{1}H NMR (200 MHz, $CDCl_3$): δ = 2.28 (s, 3H), 6.76 (bs, 1H), 7.02 (d, ^3J = 7.8 Hz, 2H), 7.07-7.45 (m, 10H), 7.57-7.72 (m, 1H); ^{13}C NMR (50 MHz $CDCl_3$): δ = 21.2 (q), 103.2 (d), 110.5 (d),

120.4 (d), 120.6 (d), 122.1 (d), 127.1 (d), 128.0 (d, 2C), 128.3 (s), 128.7 (d, 2C), 128.8 (d, 2C), 129.2 (d, 2C), 129.6 (s), 137.1 (s), 138.6 (s), 138.9 (s), 140.8 (s)

1-Phenyl-2-[4-(trifluoromethyl)phenyl]-1*H*-indole (8e, table 6). yield: 462 mg (91 %); pale yellow solid; TLC: R_f(PE:EtOAc=10:1) = 0.65; GC/MS (EI+): m/z (rel. Intensity): 337 (M^+, 100), 336 (23), 335 (12), 267 (18), 165 (34), 134 (19), 133 (42), 132 (17); HRMS (ESI^+): exact mass calculated for $C_{21}H_{14}F_3N$: 338.1151. Found: 338.1156 $[M+H]^+$; ^{1}H NMR (200 MHz, *CDCl$_3$*): δ = 6.87 (s, 1H), 7.03-7.97 (m, 13H); ^{13}C NMR (50 MHz, *CDCl$_3$*): δ = 105.1 (d), 110.7 (d), 120.9 (d), 121.0 (d), 123.1 (d), 124.1 (s, q, $^1J_{C,F}$ = 272 Hz), 125.1 (d, 2C, q, $^3J_{C,F}$ = 3.8 Hz), 127.5 (d), 127.9 (d, 2C), 128.1 (s), 128.7 (d, 2C), 128.9 (s, q, $^2J_{C,F}$ = 32.5 Hz), 129.4 (d, 2C), 136.0 (s, q, $^5J_{C,F}$ = 1.2 Hz), 138.1 (s), 138.8 (s), 139.4 (s); mixture of C2:C3=11:1 also confirmed by ^{13}C-NMR

2-(3-Nitrophenyl)-1-phenyl-1*H*-indole (8f, table 6). yield: 160 mg (34 %); shining yellow solid; M.p.: 134-135 °C; TLC: R_f(PE:EtOAc=10:1) = 0.44; GC/MS (EI+): m/z (rel. Intensity): 314 (M^+, 100), 268 (34), 267 (44), 266 (22), 265 (16), 239 (6), 134 (10), 132 (18), 121 (9); HRMS (ESI^+): exact mass calculated for $C_{20}H_{14}N_2O_2$: 315.1128. Found: 315.1131 $[M+H]^+$; ^{1}H NMR (200 MHz, *CDCl$_3$*): δ = 6.93 (s, 1H), 7.13-7.54 (m, 10H), 7.65-7.75 (m, 1H), 8.04 (ddd, ^3J = 8.0 Hz, ^4J = 2.0 Hz, ^4J = 1.0 Hz, 1H), 8.16 (t, ^4J = 1.8 Hz, 1H); ^{13}C NMR (50 MHz, *CDCl$_3$*): δ = 105.2 (d), 110.8 (d), 120.9 (d), 121.1 (d), 121.8 (d), 123.3 (d, 2C overlapping), 127.8 (d), 128.0 (d, 2C), 129.1 (d), 129.6 (d, 2C), 134.17 (s), 134.21 (d), 137.77 (s), 137.80 (s), 139.4 (s), 148.1 (s); (1 C missing due to potential overlap of signals); ^{13}C NMR (50 MHz, *acetone-d$_6$*): δ = 107.0 (d), 112.4 (d), 122.8 (d, 2C overlapping), 123.6 (d), 124.8 (d), 125.0 (d), 129.7 (d), 129.9 (d, 2C), 130.0 (s), 131.4 (d), 131.6 (d, 2C), 136.0 (s), 136.4 (d), 139.79 (s), 139.84 (s), 141.5 (s), 150.0 (s)

2-(Naphth-1-yl)-1-phenyl-1*H*-indole (8g, table 6). yield: 161 mg (34); colorless solid; M.p.: 181-183 °C; TLC: R_f(PE:EtOAc=10:1) = 0.69 ; GC/MS (EI+): m/z (rel. Intensity): 319 (M^+, 100), 318 (45), 317 (17), 242 (38), 241 (16), 215 (10), 159 (17), 158 (18), 157 (22), 152 (13); ^{1}H NMR (200 MHz, *CDCl$_3$*): δ = 6.83 (bs, 1H), 7.09-7.27 (m, 7H), 7.28-7.44 (m, 5H), 7.70-7.83 (m, 3H), 8.00-8.08 (m, 1H); ^{13}C NMR (50 MHz, *CDCl$_3$*): δ = 105.9 (d), 110.7 (d), 120.5 (d), 120.7 (d), 122.3 (d), 124.8 (d), 125.8 (d), 126.16 (d), 126.22 (d), 126.8 (d), 127.3 (d, 2C), 128.1 (d), 128.2 (s), 128.4 (d), 128.9 (2C, d), 129.2 (d), 130.4 (s), 132.6 (s), 133.4 (s), 138.0 (s), 138.2 (s), 138.6 (s)

2-(2-Methylphenyl)-1-phenyl-1*H*-indole (8h, table 6). yield: 401 mg (94 %); colorless oil; TLC: R_f(PE:EtOAc=20:1) = 0.58; GC/MS (EI+): m/z (rel. Intensity): 283 (M^+, 100), 282 (48), 280 (15), 267 (14), 206 (30), 204 (28), 178 (18), 165 (10), 140 (14), 139 (10), 133 (35), 132 (15); HRMS (ESI^+): exact mass calculated for $C_{21}H_{17}N$: 284.1434. Found: 284.1421; ^{1}H NMR (200 MHz, *CDCl$_3$*): δ = 2.02 (s, 3H), 6.56 (bs, 1H), 6.94-7.48 (m, 12H), 7.54-7.66 (m, 1H); ^{13}C NMR (50 MHz, *CDCl$_3$*): δ = 20.3 (q), 104.4 (d), 110.5 (d), 120.4 (d), 120.5 (d), 122.0 (d), 125.2 (d), 126.7 (d), 127.3 (d, 2C), 128.2 (d), 128.3 (s), 128.9 (d, 2C), 129.9 (d), 131.5 (d), 132.5 (s), 137.5 (s), 137.6 (s), 138.2 (s), 140.1 (s) - The desired product was isolated as mixture containing the 1,3-diarylindole byproduct (GC/MS: C2:C3=45:55).

5-Methoxy-1,2-diphenyl-1*H*-indole (8j, table 6). yield: 103 mg (82 %); colorless solid; M.p.: 131-134 °C; TLC: R_f(PE:EtOAc=10:1)=0.62; GC/MS (EI+): m/z (rel. Intensity): 299 (M^+, 100), 284 (28), 256 (37), 254 (24), 127 (13), 77 (11); ^{1}H NMR (200 MHz, *CDCl$_3$*): δ = 3.85 (s, 3H), 6.72 (s, 1H), 6.82 (dd, ^3J = 8.9 Hz, ^4J = 2.5 Hz, 1H), 7.08-7.43 (m, 12H); ^{13}C NMR (50 MHz, *CDCl$_3$*): δ = 55.8 (q), 102.0 (d), 103.4 (d),

111.4 (d), 112.4 (d), 127.0 (d), 127.2 (d), 127.9 (d, 2C), 128.1 (d, 2C), 128.6 (s), 128.8 (d, 2C), 129.2 (d, 2C), 132.5 (s), 134.3 (s), 138.6 (s), 141.1 (s), 154.8 (s)

1-(4-Methoxyphenyl)-2-phenyl-1*H*-indole (8k, table 6). yield: 68 mg (54 %); pale yellow solid; M.p.: 138-140 °C; TLC: R_f(PE:EtOAc=10:1) = 0.59; GC/MS (EI+): m/z (rel. Intensity): 299 (M^+, 100), 284 (25), 256 (12), 255 (13), 254 (23), 165 (14), 150 (11), 133 (12), 127 (15); ^{1}H NMR (200 MHz, *CDCl$_3$*): δ = 3.73 (s, 3H), 6.70 (d, ^4J = 0.5 Hz, 1H), 6.83 (d, ^3J = 8.9 Hz, 2H), 7.01-7.26 (m, 10H), 7.53-7.66 (m, 1H); ^{13}C NMR (50 MHz, *CDCl$_3$*): δ = 55.4 (q), 103.1 (d), 110.6 (d), 114.4 (d, 2C), 120.4 (d), 120.5 (d), 122.1 (d), 127.2 (d), 128.0 (s), 128.1 (d, 2C), 128.9 (d, 2C), 129.1 (d, 2C), 131.3 (s), 132.6 (s), 139.3 (s), 140.8 (s), 158.5 (s)

5-Nitro-1,2-diphenyl-1*H*-indole (8n, table 6). yield: 59 mg (45 %); yellow solid; M.p.: 182-185 °C; TLC: R_f(PE:EtOAc=10:1) = 0.47; GC/MS (EI+): m/z (rel. Intensity): 314 (M^+, 100), 268 (34), 267 (42), 266 (21), 133 (12), 77 (10); ^{1}H NMR (200 MHz, *CDCl$_3$*): δ = 6.86 (s, 1H), 7.11-7.25 (m, 8H), 7.31-7.43 (m, 3H), 7.99 (dd, ^3J = 9.1 Hz, ^4J = 2.2 Hz, 1H), 8.55 (d, ^4J = 2.2 Hz, 1H); ^{13}C NMR (50 MHz, *CDCl$_3$*): δ = 105.0 (d), 110.6 (d), 117.5 (d), 117.8 (d), 127.4 (s), 127.8 (d, 2C), 128.20 (d), 128.25 (d), 128.4 (d, 2C), 128.9 (d, 2C), 129.6 (d, 2C), 131.2 (s), 137.3 (s), 141.6 (s), 142.4 (s), 144.0 (s)

1-(4-Nitrophenyl)-2-phenyl-1*H*-indole (8o, table 6). yield: 78 mg (59 %); yellow solid; M.p.: 139-141 °C; TLC: Rf(PE:EtOAc=10:1) = 0.57; GC/MS (EI+): m/z (rel. Intensity): 314 (M^+, 100), 268 (39), 267 (36), 266 (28), 265 (15), 191 (10), 165 (10), 134 (17), 133 (12); ^{1}H NMR (200 MHz, *CDCl$_3$*): δ = 6.74 (s, 1H), 7.05-7.22 (m, 8H), 7.27 (d, ^3J = 8.9 Hz, 2H), 7.54-7.64 (m, 1H), 8.14 (d, ^3J = 8.9 Hz, 2H); ^{13}C NMR (50 MHz, *CDCl$_3$*): δ = 105.9 (d), 110.1 (d), 121.0 (d), 121.7 (d), 123.2 (d), 124.7 (d, 2C), 127.9 (d), 128.0 (d, 2C), 128.5 (d, 2C), 128.7 (s), 128.9 (d, 2C), 131.7 (s), 138.1 (s), 140.3 (s), 144.2 (s), 145.8 (s)

1-(4-Fluorophenyl)-2-phenyl-1*H*-indole (8p, table 6). yield: 93 mg (77 %); colorless solid; Mp.: 122-124 °C; TLC: R_f(PE:EtOAc=10:1) = 0.68; GC/MS (EI+): m/z (rel. Intensity): 287 (M^+, 100), 286 (29), 285 (20), 183 (11), 165 (14); ^{1}H NMR (200 MHz, *CDCl$_3$*): δ = 6.92 (s, 1H), 7.12-7.24 (m, 2H), 7.25-7.41 (m, 10H), 7.75-7.88 (m, 1H); ^{13}C NMR (50 MHz, *CDCl$_3$*): δ = 103.7 (d), 110.4 (d), 116.1 (d, d, $^3J_{CF}$ = 22.8 Hz), 120.7 (d, d, $^4J_{CF}$ = 10.6 Hz), 122.4 (d), 127.4 (d), 128.2 (d, 2C), 128.9 (d, 2C), 129.5 (d), 129.7 (d), 132.3 (s), 134.5 (s, d, $^4J_{CF}$ = 3.1 Hz), 139.1 (s), 140.7 (s), 161.4 (s, d, $^2J_{CF}$ = 247 Hz) - one quaternary carbon missing due to potential overlap

1-(Naphth-1-yl)-2-phenyl-1*H*-indole (8q, table 6). yield: 100 mg (75 %); pale yellow solid; M.p.: 48-52 °C (Lit. 73-74 °C); TLC: Rf(PE:EtOAc=10:1)=0.70; GC/MS (EI+): m/z (rel. Intensity): 319 (M^+, 100), 318 (39), 317 (22), 241 (11), 165 (10), 158 (12), 152 (10); ^{1}H NMR (200 MHz, *CDCl$_3$*): δ = 6.70 (dd, ^3J = 8.1 Hz, ^4J = 0.9 Hz, 1H), 6.83 (d, ^4J = 0.7 Hz, 1H), 6.89-7.40 (m, 12H), 7.63 (d, ^3J = 7.5 Hz, 1H), 7.69-7.84 (m, 2H); ^{13}C NMR (50 MHz, *CDCl$_3$*): δ = 103.2 (d), 111.2 (d), 120.5 (d), 120.6 (d), 122.2 (d), 123.5 (d), 125.5 (d), 126.5 (d), 127.0 (d), 127.1 (d), 127.3 (d), 128.07 (d, 2C), 128.13 (d, 2C), 128.2 (d), 128.5 (d), 131.3 (s), 132.5 (s), 134.3 (s), 135.2 (s), 140.2 (s), 142.1 (s); one quaternary carbon missing due to potential overlap.

***N*^{1},*N*^{2}-Bis(3-iodopyridin-2-yl)-*N*^{1},*N*^{2}-dimethylethane-1,2-diamine (11, Scheme 3).** Formed as by-product in the attempted synthesis of **7h**. Yield: 42 mg (quant.); pale yellow needles, TLC: R_f(EtOAc:EtOAc=5:1) = 0.34, HRMS (ESI^+): exact mass calculated for $C_{14}H_{16}I_2N_4$: 494.9537 Found: 494.9550 $[M+H]^+$; ^{1}H NMR (200 MHz, *CDCl$_3$*): δ = 2.90 (s, 6H), 3.49 (s, 4H), 6.50 (dd, ^3J = 7.6 Hz, ^3J = 4.7

Hz, 2H), 7.95 (dd, ^3J = 7.6 Hz, ^4J = 1.6 Hz, 2H), 8.14 (dd, J = 4.7, ^4J = 1.6 Hz, 2H); ^{13}C NMR (50 MHz, APT, *CDCl₃*): δ = 40.4 (q, 2C), 52.2 (t, 2C), 86.4 (s, 2C), 118.4 (d, 2C), 146.7 (d, 2C), 149.2 (d, 2C), 162.6 (s, 2C).

1-(1-Phenyl-1*H*-indol-3-yl)-3,4-dihydroisoquinoline (12, Scheme 4). Isolated as by-product. Yield: 23 mg (18 %); colorless solid; M.p.: 49-51 °C; GC/MS (EI+): m/z (rel. Intensity): 323 (M^+, 10), 322 (52), 321 (100), 294 (15), 293 (10), 217 (10), 216 (10); HRMS (ESI+): exact mass calculated for $C_{23}H_{18}N_2$: 323.1543. Found: 323.1537 $[M+H]^+$; ^{1}H NMR (200 MHz, *CDCl₃*): δ = 2.68 (dd, ^3J = 7.8 Hz, ^3J = 6.4 Hz, 1H), 3.75 (dd, ^3J = 7.8 Hz, ^3J = 6.4 Hz, 1H), 7.58-7.01 (m, 14H), 7.98-7.85 (m, 1H); ^{13}C NMR (50 MHz, APT, *CDCl₃*): δ = 26.3 (t), 47.0 (t), 110.4 (d), 116.3 (s), 121.2 (d), 122.1 (d), 122.9 (d), 124.4 (d, 2C), 126.5 (d), 126.8 (d), 127.2 (d), 127.5 (d), 127.7 (s), 129.4 (s), 129.5 (d, 2C), 130.0 (d), 130.3 (d), 136.4 (s), 138.9 (s), 139.1 (s), 161.8 (s)

1,1-Dimethylethyl 2-[1-(4-nitrophenyl)-1*H*-indole-3-carbonyl]-phenethylcarbamate (13, Scheme 5). Procedure A: Isolated according to the general procedure for N-arylindolation of BocNTHIQ, Procedure B: The starting material **7e** (20 mg, 0.0426 mmol) and $Cu(NO_3)_2$ x $3H_2O$ (1.0 mg, 4.2 µmol) were placed into a 5 mL glass vial and tBHP (26 µL, 0.128 mmol) added in one portion at room temperature. The mixture was heated to 80 °C under air for 17 hours. Another 3 equiv. of tBHP were added dropwise, and the reaction mixture stirred for another 11 hours. The reaction mixture was cooled down to room temperature, diluted with DCM and directly subjected to column chromatography to afford the desired product in good yield. Yield: Method A: 50 mg (12 %), Method B: 16 mg (77 %); shining yellow powder; M.p.:78-82 °C; TLC: R_f (PE:EtOAc=5:1) = 0.11; HRMS (ESI^+): exact mass calculated for $C_{28}H_{27}N_3O_5$: 508.1843. Found: 508.1839 $[M+Na]^+$; ^{1}H NMR (200 MHz, *CDCl₃*): δ = 1.37 (s, 9H), 2.93 (t, ^3J = 6.7 Hz, 2H), 3.34-3.37 (m, 2H), 5.12 (bs, 1H), 7.28-7.68 (m, 8H), 7.74 (d, ^3J = 9.0 Hz, 2H), 8.41 (d, ^3J = 9.0 Hz, 2H), 8.45-8.54 (m, 1H); ^{13}C NMR (50 MHz, APT, *CDCl₃*): δ = 28.4 (q, 3C), 33.2 (t), 42.1 (t), 78.9 (s), 110.6 (d), 120.3 (s), 123.3 (d), 124.1 (d), 124.8 (d, 2C), 125.1 (d), 125.5 (d, 2C), 125.8 (d), 127.5 (s), 128.2 (d), 130.1 (d), 131.0 (d), 136.5 (s), 137.0 (d), 137.8 (s), 140.4 (s), 143.6 (s), 146.4 (s), 156.0 (s), 193.0 (s)

3-(3-Nitrophenyl)-1-phenyl-1*H*-indole (14f, table 6). yield: 48 mg (10 %); shining yellow solid; M.p.: 126-127 °C; TLC: R_f(PE:EtOAc=10:1) = 0.42; GC/MS (EI+): m/z (rel. Intensity): 314 (M^+, 100), 268 (34), 267 (30), 266 (14), 265 (9), 239 (5), 199 (11), 165 (20), 164 (13), 163 (10), 134 (10); 1H NMR (200 MHz, *CDCl₃*): δ = 7.21-7.65 (m, 10H), 7.89-8.06 (m, 2H), 8.11 (dd, ^3J = 8.2 Hz, ^4J = 1.8 Hz, 1H), 8.53 (t, ^4J = 2.0 Hz, 1H); 13C NMR (50 MHz, *CDCl₃*): δ = 111.1 (d), 116.7 (s), 119.5 (d), 120.7 (d), 121.5 (d), 121.8 (d), 123.3 (d), 124.5 (d, 2C), 126.4 (d), 127.1 (d), 129.6 (d), 129.8 (d, 2C), 133.0 (d), 136.8 (s), 137.0 (s), 139.0 (s), 148.8 (s) one quaternary C overlapping; 13C NMR (50 MHz, *acetone-D₆*): δ = 112.9 (d), 118.1 (s), 121.3 (d), 122.3 (d), 123.0 (d), 123.4 (d), 125.1 (d), 126.3 (d, 2C), 128.4 (s), 128.9 (d), 129.2 (d), 131.7 (d, 2C), 131.9 (d), 134.9 (d), 138.6 (s), 139.0 (s), 140.9 (s), 150.8 (s)

1-(1,2-Diphenyl-1*H*-indol-3-yl)-1,2,3,4-tetrahydroisoquinoline (15, table 9). TMSCl (204 mg, 1.88 mmol, 5.0 equiv.) was added dropwise to a solution of **1a** (188 mg, 0.375 mmol, 1.0 equiv.) in dry MeOH (3 mL) at room temperature. The reaction mixture was degassed with argon for 30 seconds and stirred for 2 hours in an argon atmosphere. Since the conversion was very low (TLC), the reaction mixture was heated to 40 °C and another 5 equiv. of TMSCl were added dropwise. After 10 hours another portion (5 equiv.) of TMSCl was added and the reaction mixture was stirred for additional 12

hours. The reaction mixture was poured onto ice-cold 2N aqueous sodium hydroxide solution and extracted three times with EtOAc. The collected organic layers were washed once with brine, dried over sodium sulfate, filtered and evaporated. Product **15** was obtained by flash chromatography using PE:EtOAc =100:0 → 0:100 (75 minutes). Yield: 122 mg (82 %); colorless solid; M.p.: 208-210 °C; TLC: Rf(EtOAc)=0.32; HRMS (ESI$^+$): exact mass calculated for $C_{29}H_{24}N_2$: 401.2012. Found: 401.2013 $[M+H]^+$; GC/MS (EI$^+$): m/z (rel. Intensity): 400 (M^+, 71), 399 (100), 398 (14), 397 (36), 384 (16), 323 (43), 295 (32), 294 (26), 269 (30), 268 (17), 267 (20), 217 (12), 216 (11), 200 (69), 199 (28), 191 (19), 177 (19), 165 (28), 132 (21), 131 (22), 130 (52), 103 (17), 77 (29); ^{1}H NMR (200 MHz, *CDCl$_3$*): δ = 1.90 (bs, 1H), 2.71-2.92 (m, 1H), 3.02-3.30 (m, 2H), 3.31-3.48 (m, 1H), 5.37 (s, 1H), 6.88-7.41 (m, 18H); ^{13}C NMR (50 MHz, *CDCl$_3$*): δ = 30.2 (t), 44.1 (t), 54.5 (d), 110.4 (d), 117.2 (s), 120.2 (d), 120.9 (d), 122.3 (d), 125.93 (d), 125.95 (d), 126.3 (s), 126.8 (d), 127.4 (d), 127.7 (d), 128.0 (2C, d), 128.1 (2C, d), 128.9 (d), 129.0 (2C, d), 130.6 (2C, d), 131.5 (s), 135.2 (s), 138.16 (s), 138.24 (s), 139.1 (s), 139.2 (s)

References:

1. Yang, S.-D.; Sun, C.-L.; Fang, Z.; Li, B.-J.; Li, Y.-Z.; Shi, Z.-J. *Angew. Chem., Int. Ed.* **2008**, *47*, 1473-1476.

2. Ghobrial, M.; Harhammer, K.; Mihovilovic, M. D.; Schnürch, M. *Chem. Commun.* **2010**, *46*, 8836-8838.

3. Ghobrial, M.; Schnürch, M.; Mihovilovic, M. D. *J. Org.Chem.***2011**, *76*, 8781-8793.

4. Kwong, F. Y.; Klapars, A.; Buchwald, S. L. *Org. Lett.* **2002**, *4*, 581-584.

5. Antilla, J. C.; Klapars, A.; Buchwald, S. L. *J. Am. Chem. Soc.* **2002**, *124*, 11684-11688.

6. Tang, B.-X.; Guo, S.-M.; Zhang, M.-B.; Li, J.-H. *Synthesis* **2008**, 1707-1716.

7. Rao, R. K.; Naidu, A. B.; Jaseer, E. A.; Sekar, G. *Tetrahedron* **2009**, *65*, 4619-4624.

3. Copies of ^{1}H and ^{13}C-NMR spectra of compounds

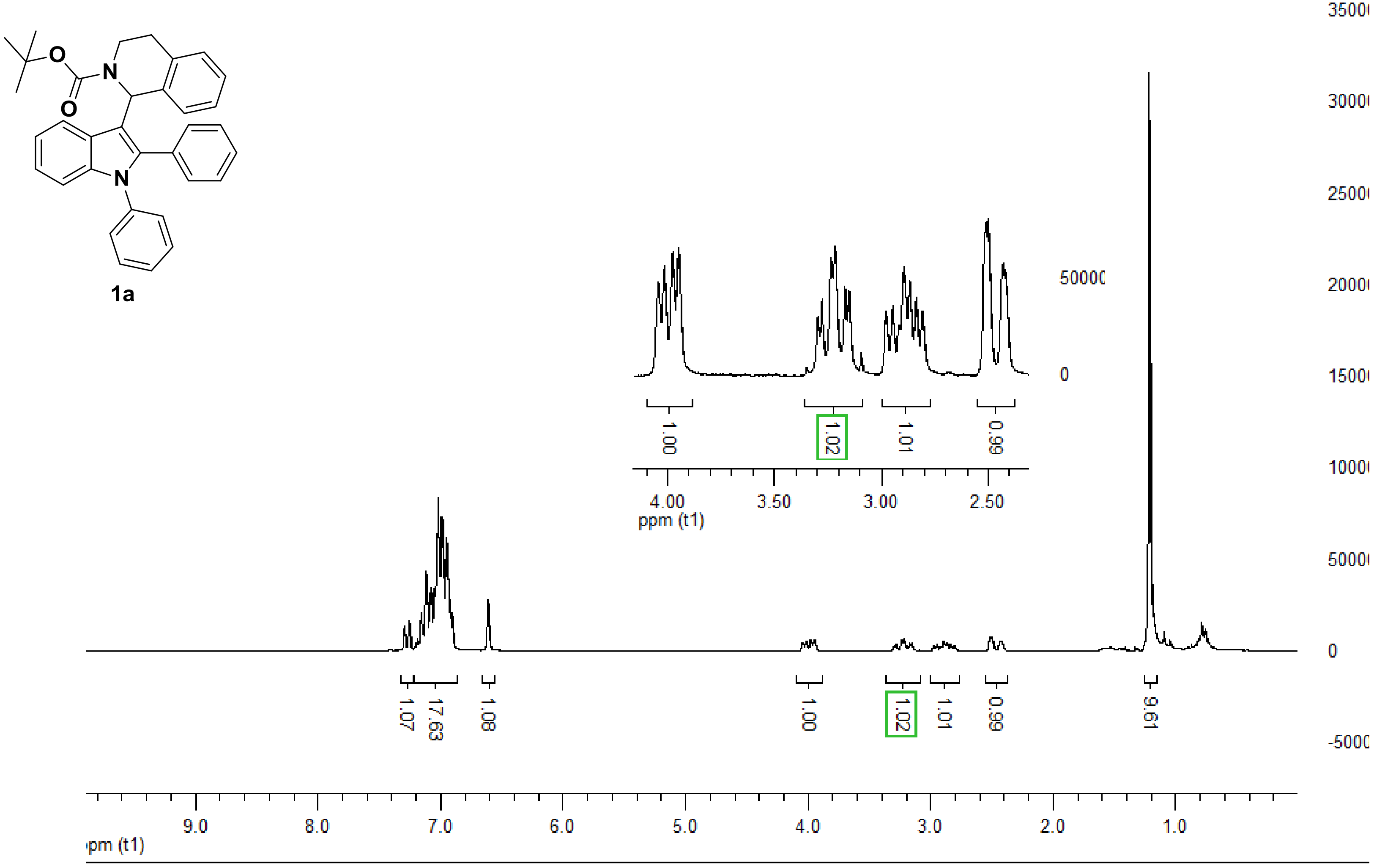

1a

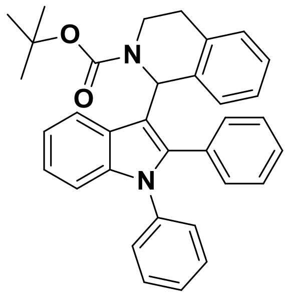

O
N
O
N

1a

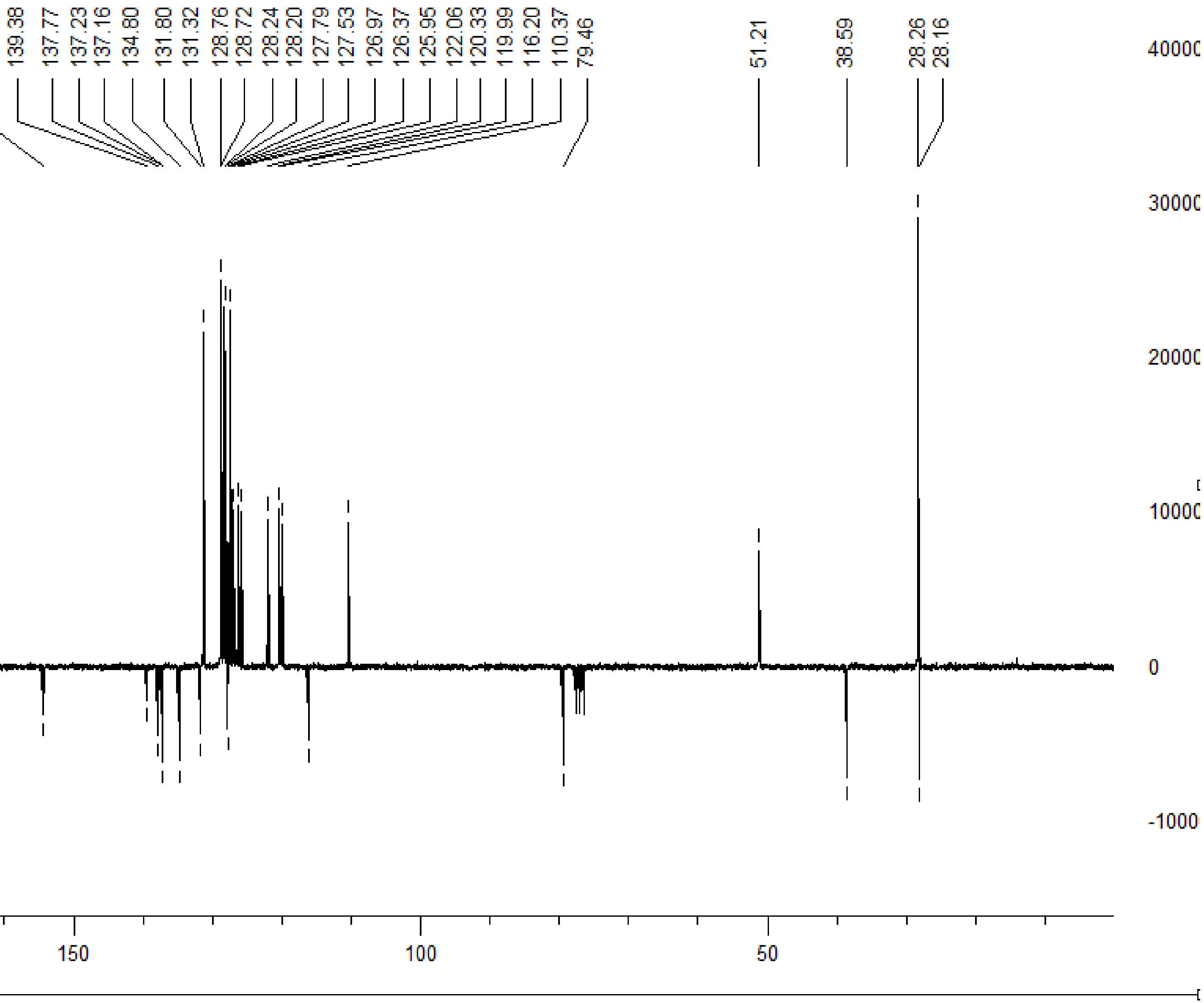

154.32
139.38
137.77
137.23
137.16
134.80
131.80
131.32
128.76
128.72
128.24
128.20
127.79
127.53
126.97
126.37
125.95
122.06
120.33
119.99
116.20
110.37
79.46
51.21
38.59
28.26
28.16
40000
30000
20000
10000
0
-1000
150
100
50
ppm (t1)

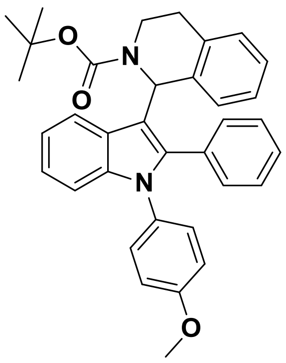

1b

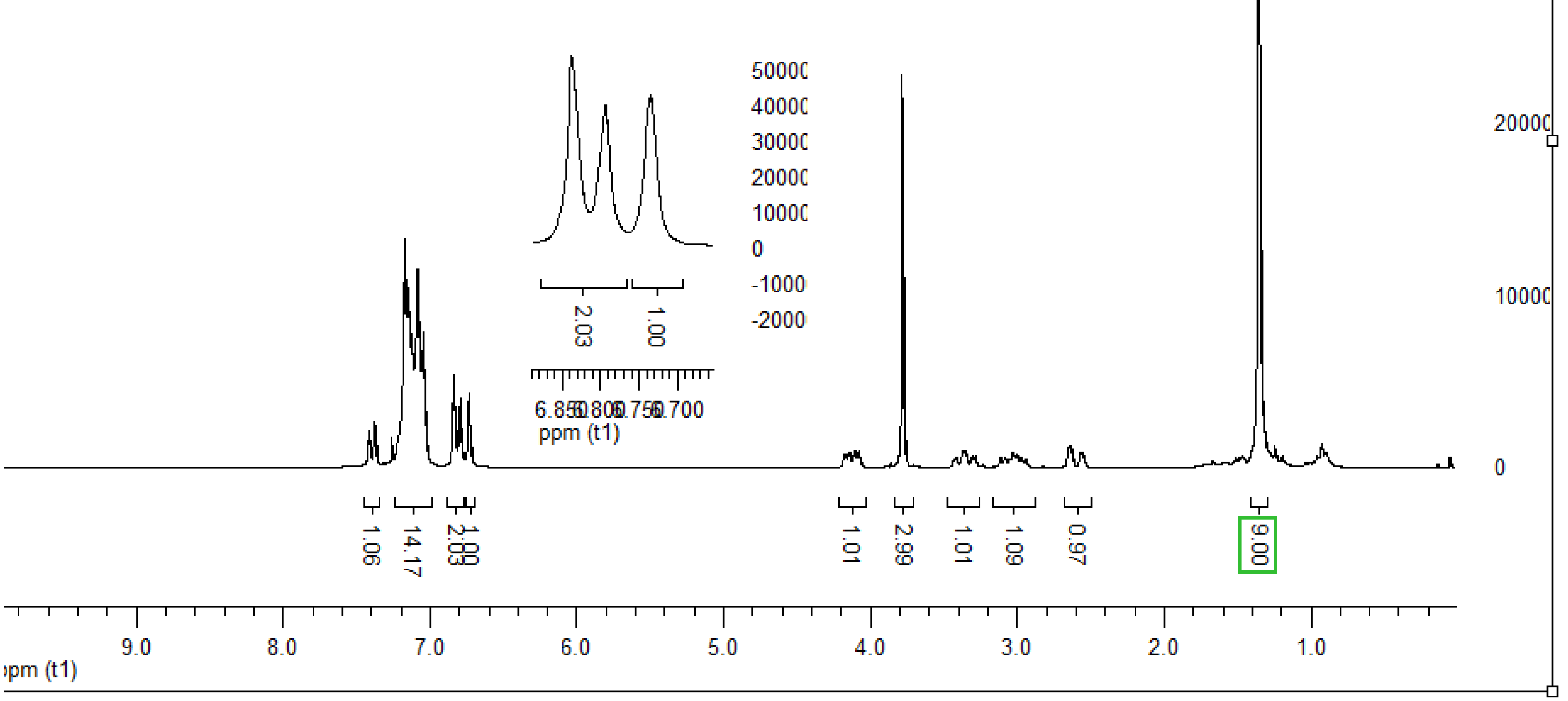

ppm (t1)
9.0
8.0
7.0
6.0
5.0
4.0
3.0
2.0
1.0
1.06
14.17
2.83
1.00
1.01
2.99
1.01
1.09
0.97
9.00
2.03
1.00
6.850
6.800
6.750
6.700
ppm (t1)

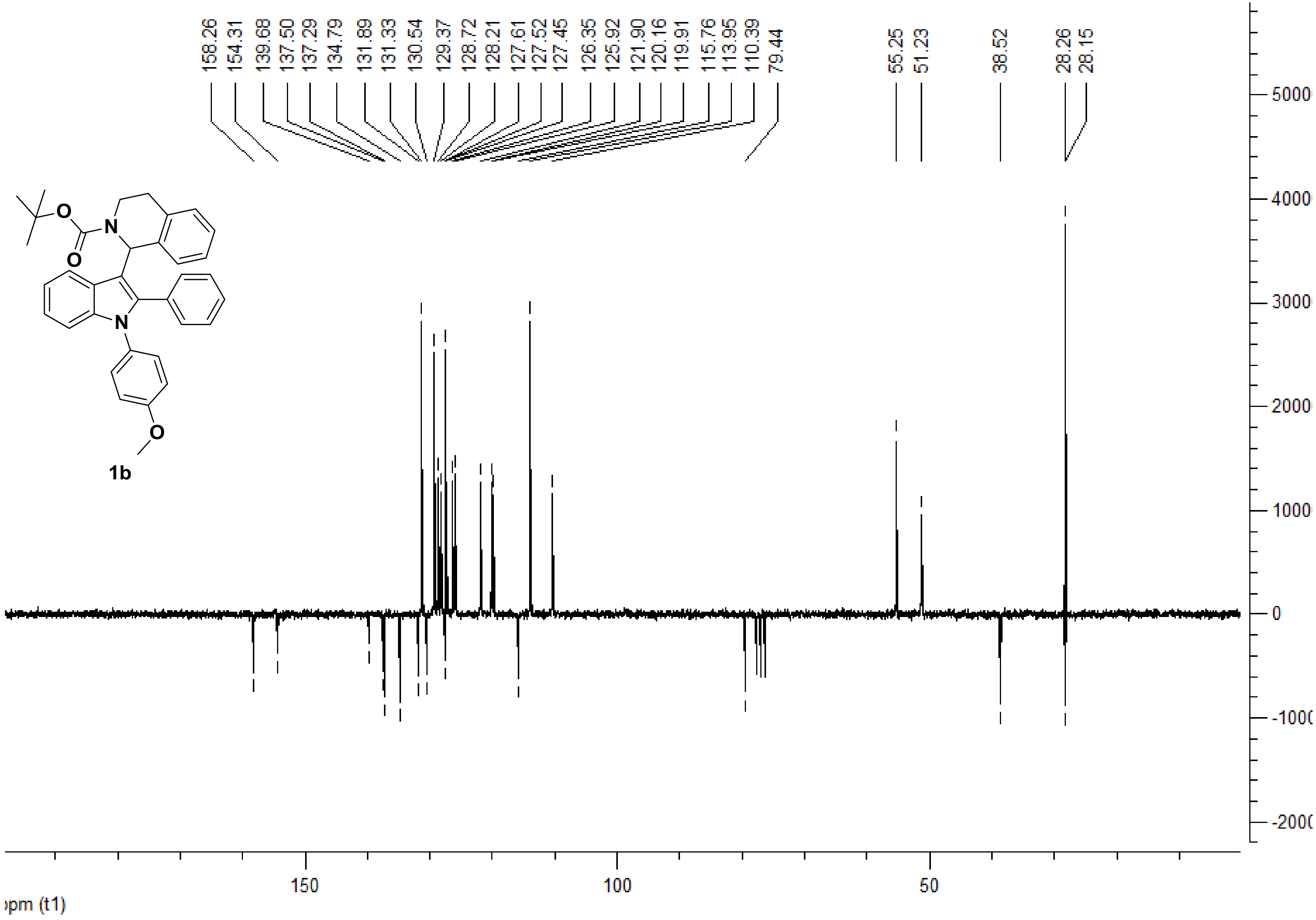

158.26
154.31
139.68
137.50
137.29
134.79
131.89
131.33
130.54
129.37
128.72
128.21
127.61
127.52
127.45
126.35
125.92
121.90
120.16
119.91
115.76
113.95
110.39
79.44
55.25
51.23
38.52
28.26
28.15
O
N
O
N
O
1b
5000
4000
3000
2000
1000
0
-1000
-2000
150
100
50
ppm (t1)

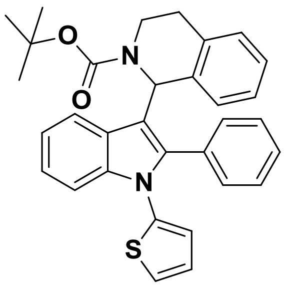

1c

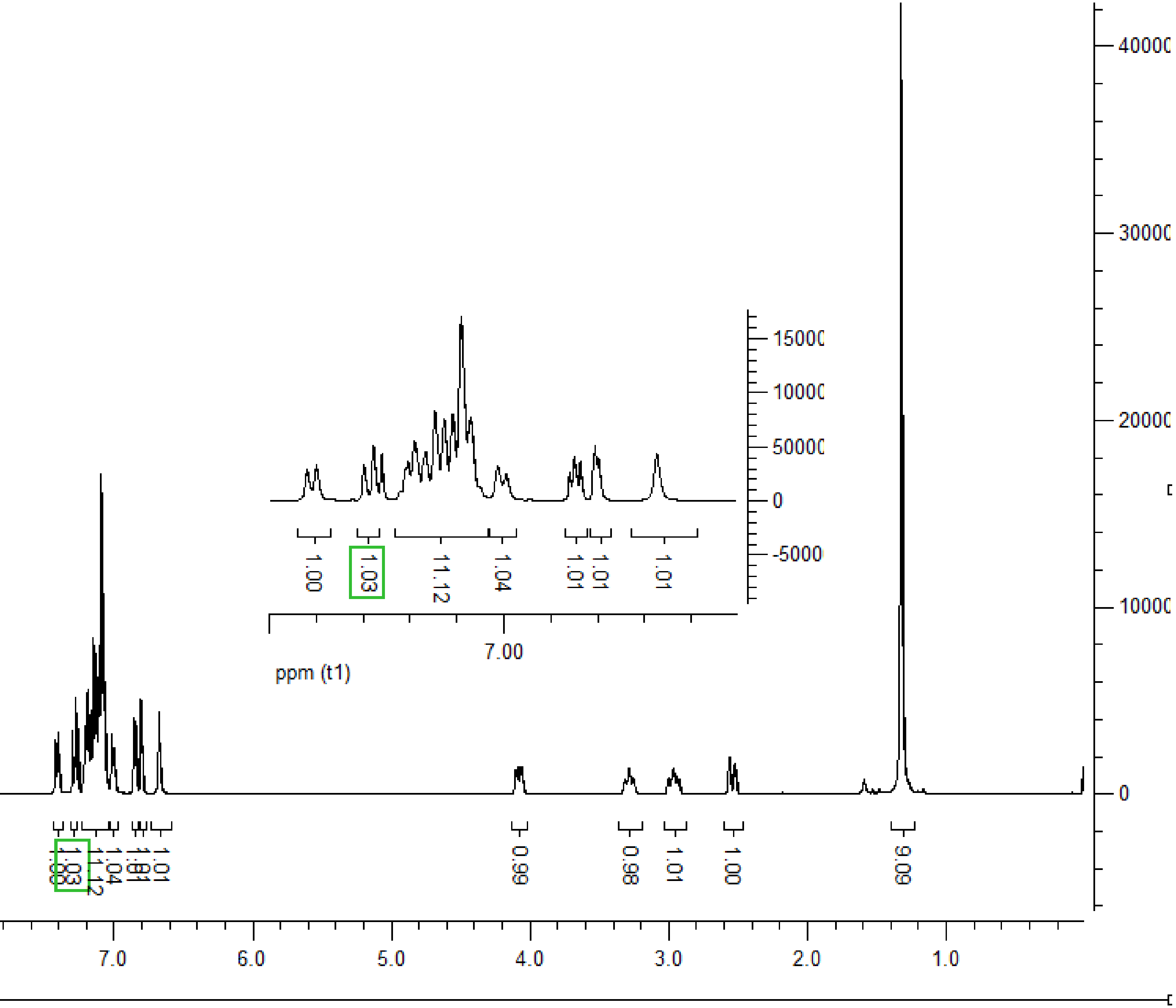

ppm (t1)
7.00
1.00
1.03
11.12
1.04
1.01
1.01
1.01
15000
10000
50000
0
-5000
9.0
8.0
7.0
6.0
5.0
4.0
3.0
2.0
1.0
0.99
0.98
1.01
1.00
9.09
40000
30000
20000
10000

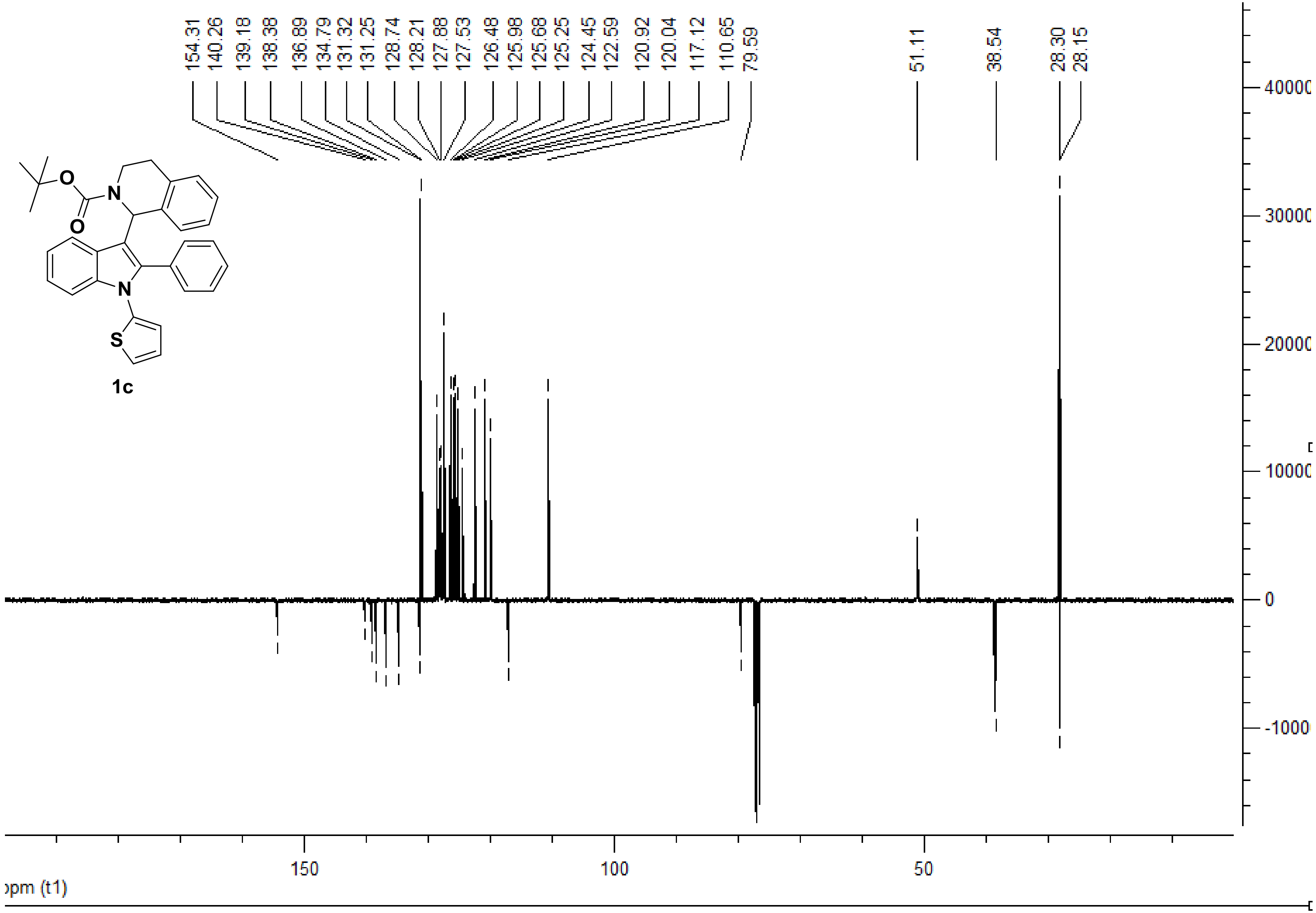
154.31
140.26
139.18
138.38
136.89
134.79
131.32
131.25
128.74
128.21
127.88
127.53
126.48
125.98
125.68
125.25
124.45
122.59
120.92
120.04
117.12
110.65
79.59
51.11
38.54
28.30
28.15
O
N
O
N
S
1c
4000
3000
2000
1000
0
-1000
150
100
50
ppm (t1)

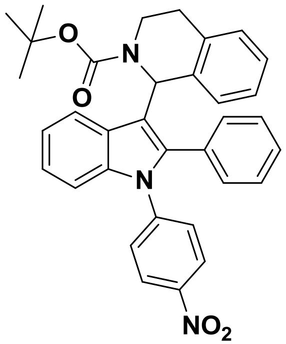
NO2

1d

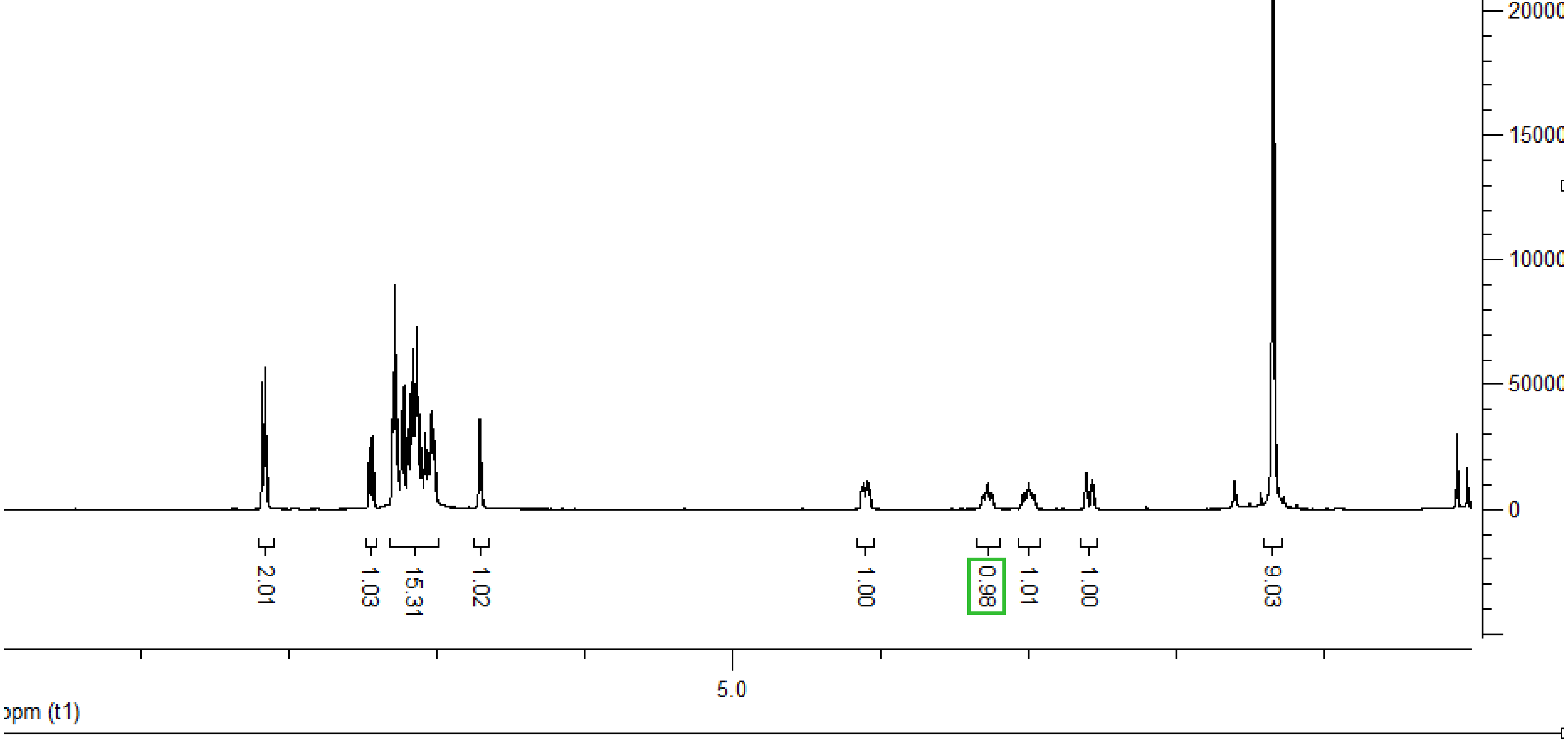
30000
25000
20000
15000
10000
50000
0
2.01
1.03
15.31
1.02
1.00
0.98
1.01
1.00
9.03
5.0
ppm (t1)

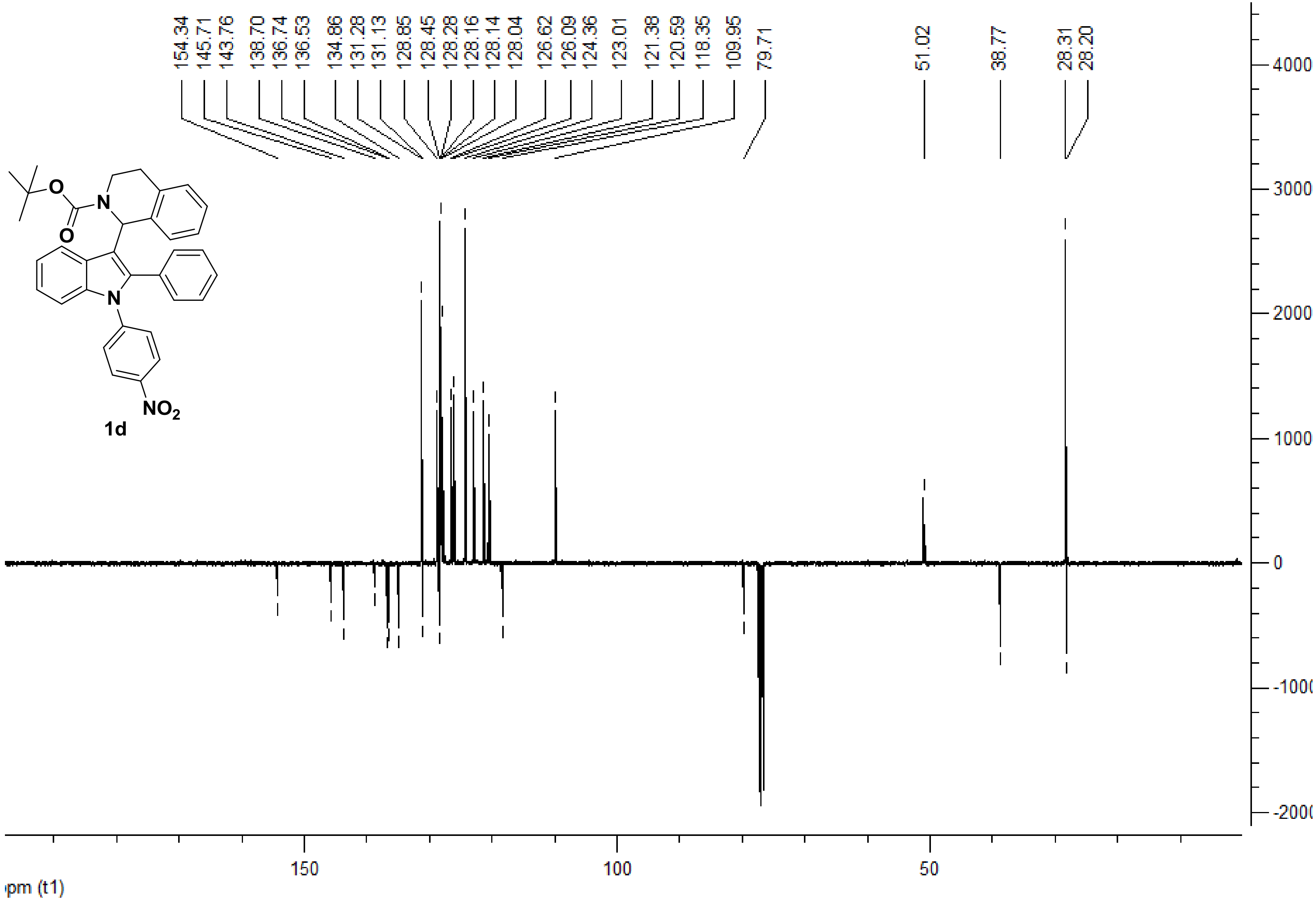
154.34
145.71
143.76
138.70
136.74
136.53
134.86
131.28
131.13
128.85
128.45
128.28
128.16
128.14
128.04
126.62
126.09
124.36
123.01
121.38
120.59
118.35
109.95
79.71
51.02
38.77
28.31
28.20
O
N
O
N
NO2
1d
4000
3000
2000
1000
0
-1000
-2000
150
100
50
ppm (t1)

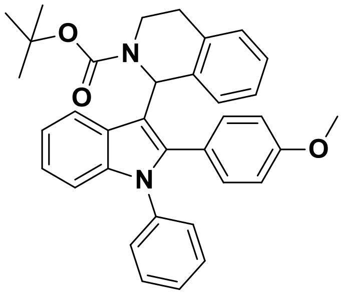
1e

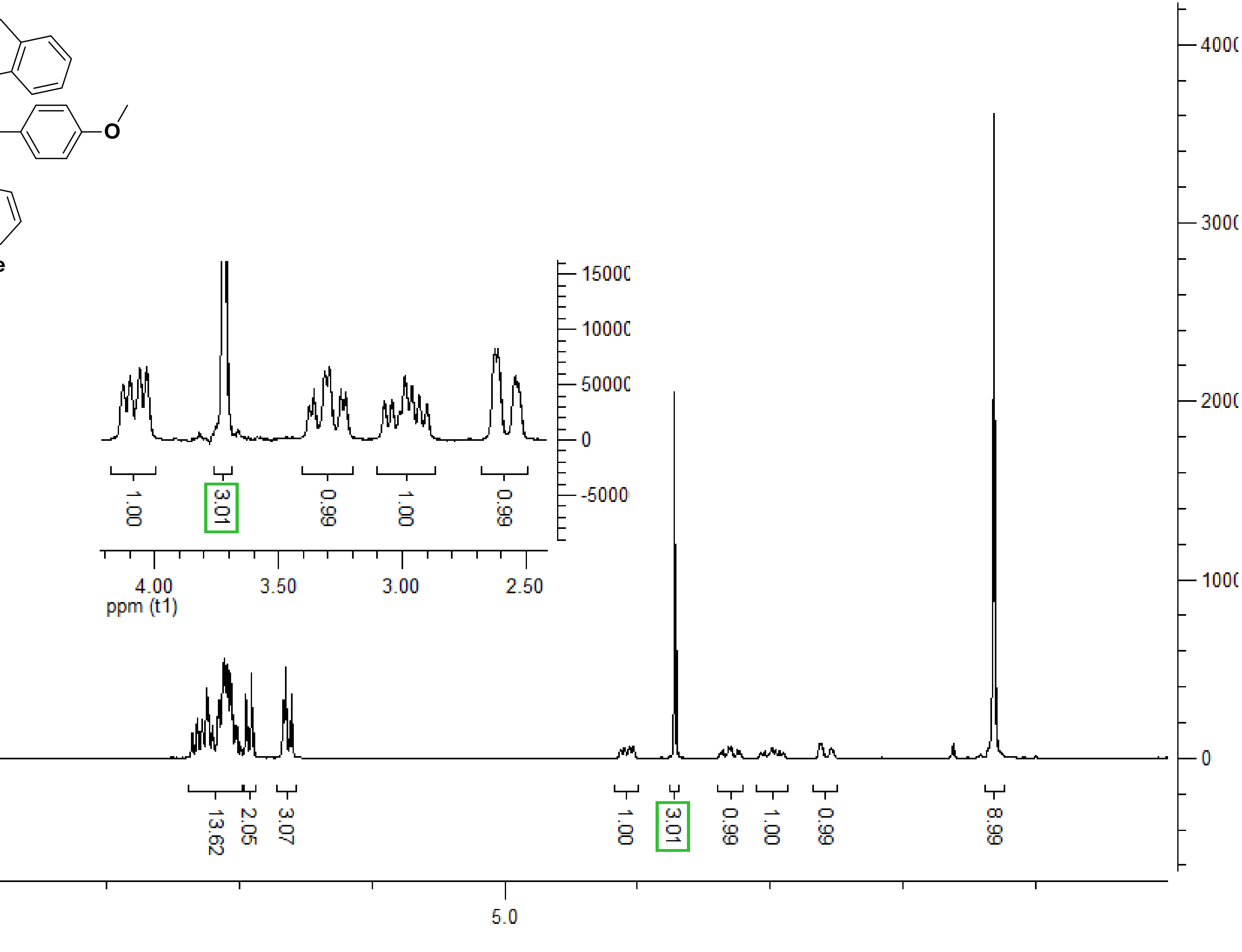
15000
10000
5000
0
-5000
1.00
3.01
0.99
1.00
0.99
4.00
3.50
3.00
2.50
ppm (t1)
4000
3000
2000
1000
0
13.62
2.05
3.07
1.00
3.01
0.99
1.00
0.99
8.99
5.0
ppm (t1)

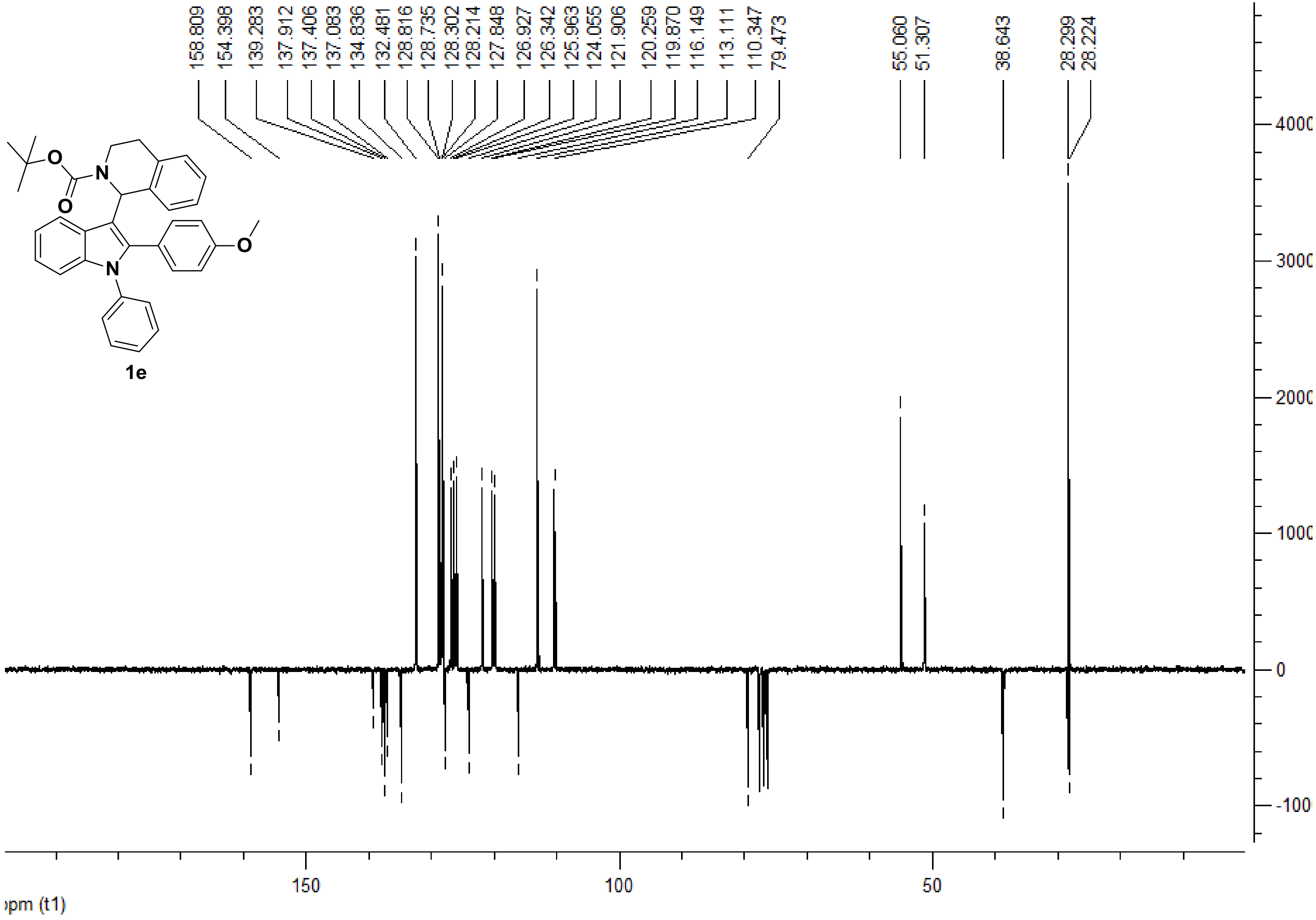

158.809
154.398
139.283
137.912
137.406
137.083
134.836
132.481
128.816
128.735
128.302
128.214
127.848
126.927
126.342
125.963
124.055
121.906
120.259
119.870
116.149
113.111
110.347
79.473
55.060
51.307
38.643
28.299
28.224
O
N
O
N
O
1e
ppm (t1)
150
100
50
4000
3000
2000
1000
0
-100

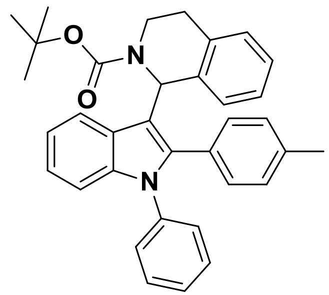

1f

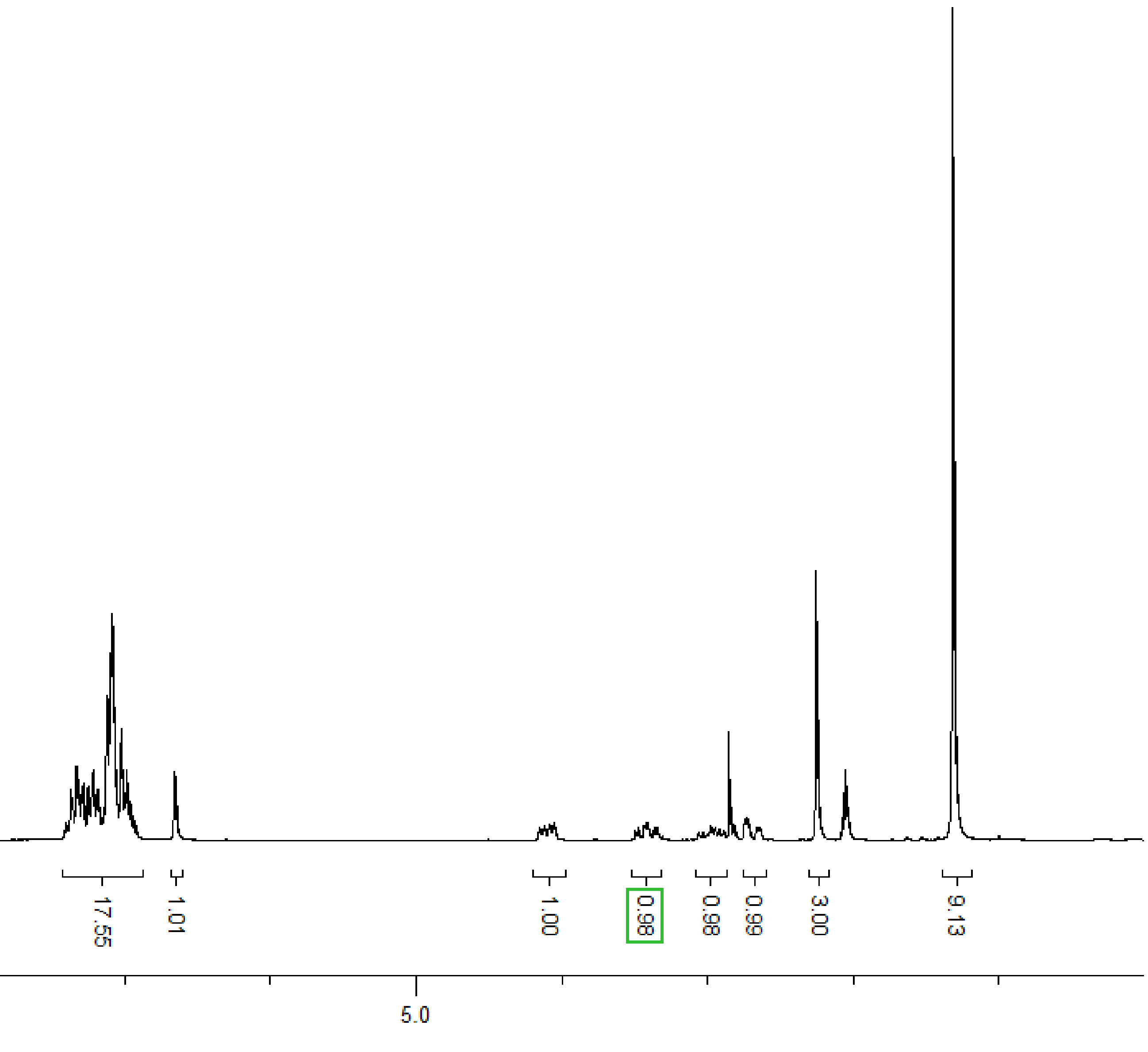

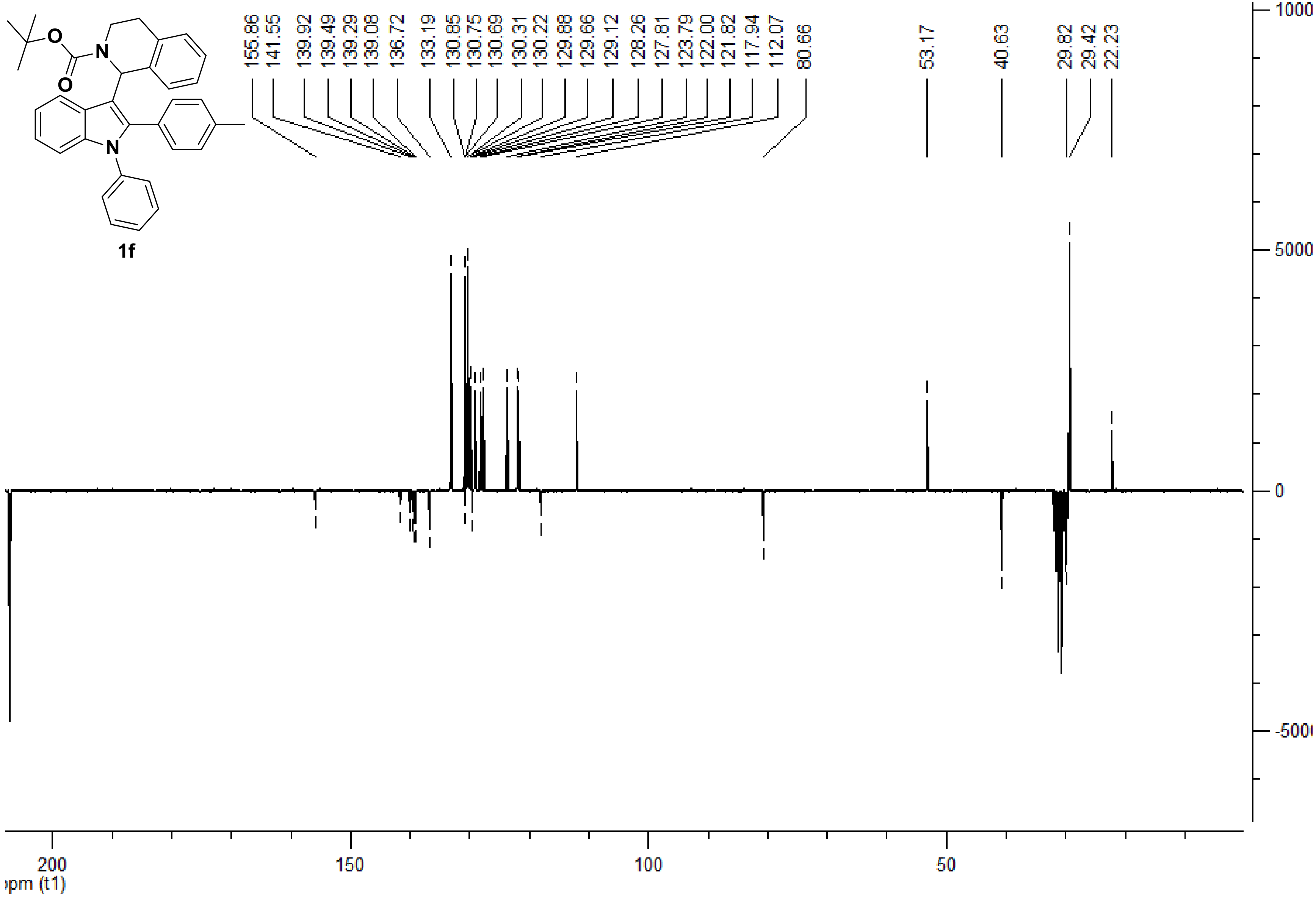

155.86
141.55
139.92
139.49
139.29
139.08
136.72
133.19
130.85
130.75
130.69
130.31
130.22
129.88
129.66
129.12
128.26
127.81
123.79
122.00
121.82
117.94
112.07
80.66
53.17
40.63
29.82
29.42
22.23
1000
5000
0
-500
200
150
100
50
ppm (t1)

1f

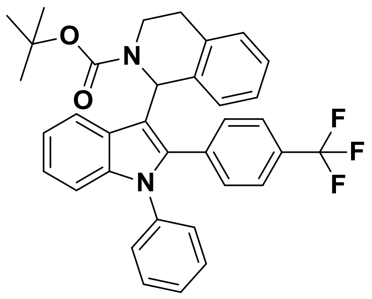

O
N
O
F
F
F
N

1g

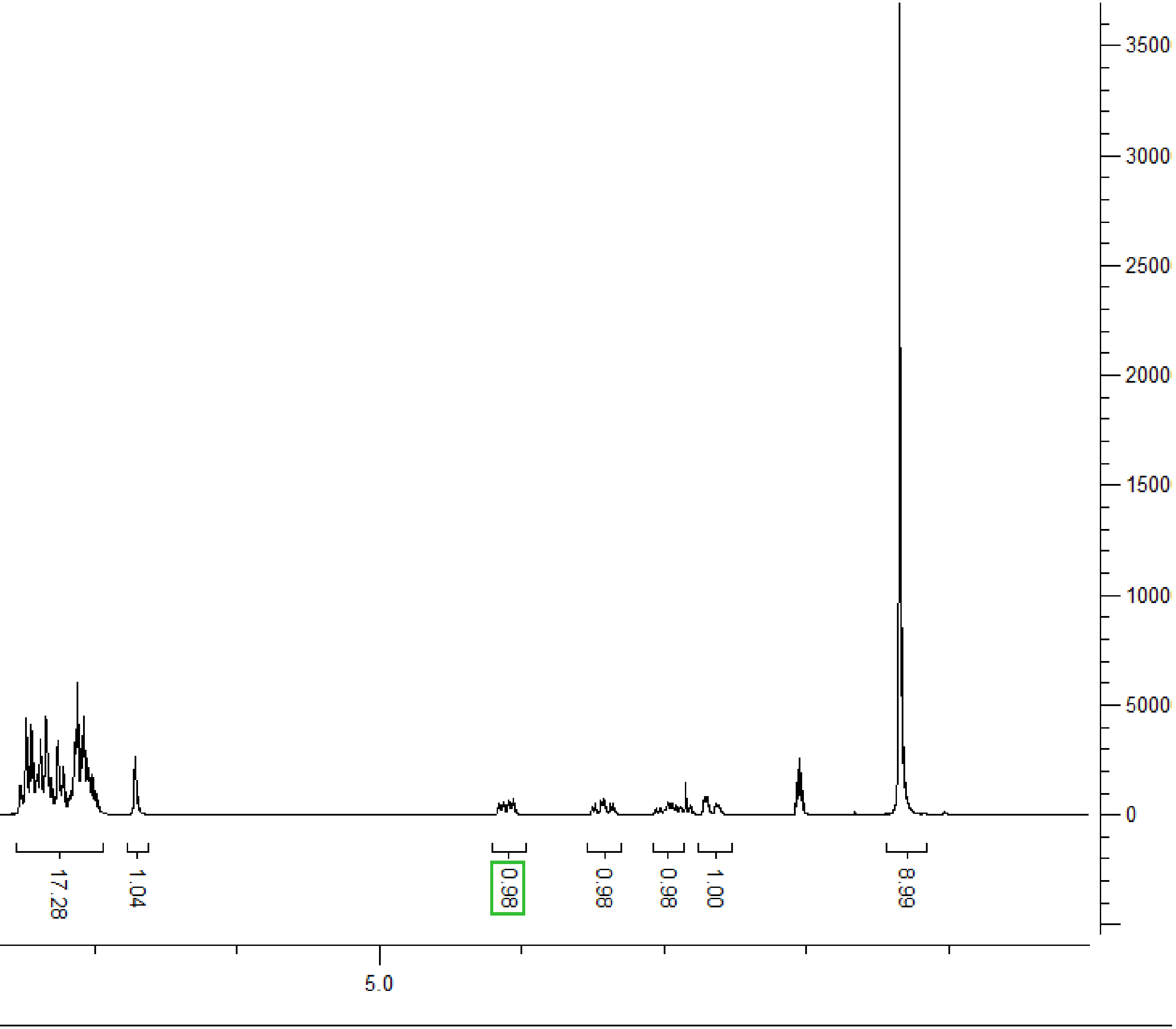

3500
3000
2500
2000
1500
1000
5000
0
17.28
1.04
0.98
0.98
0.98
1.00
8.99
5.0
ppm (t1)

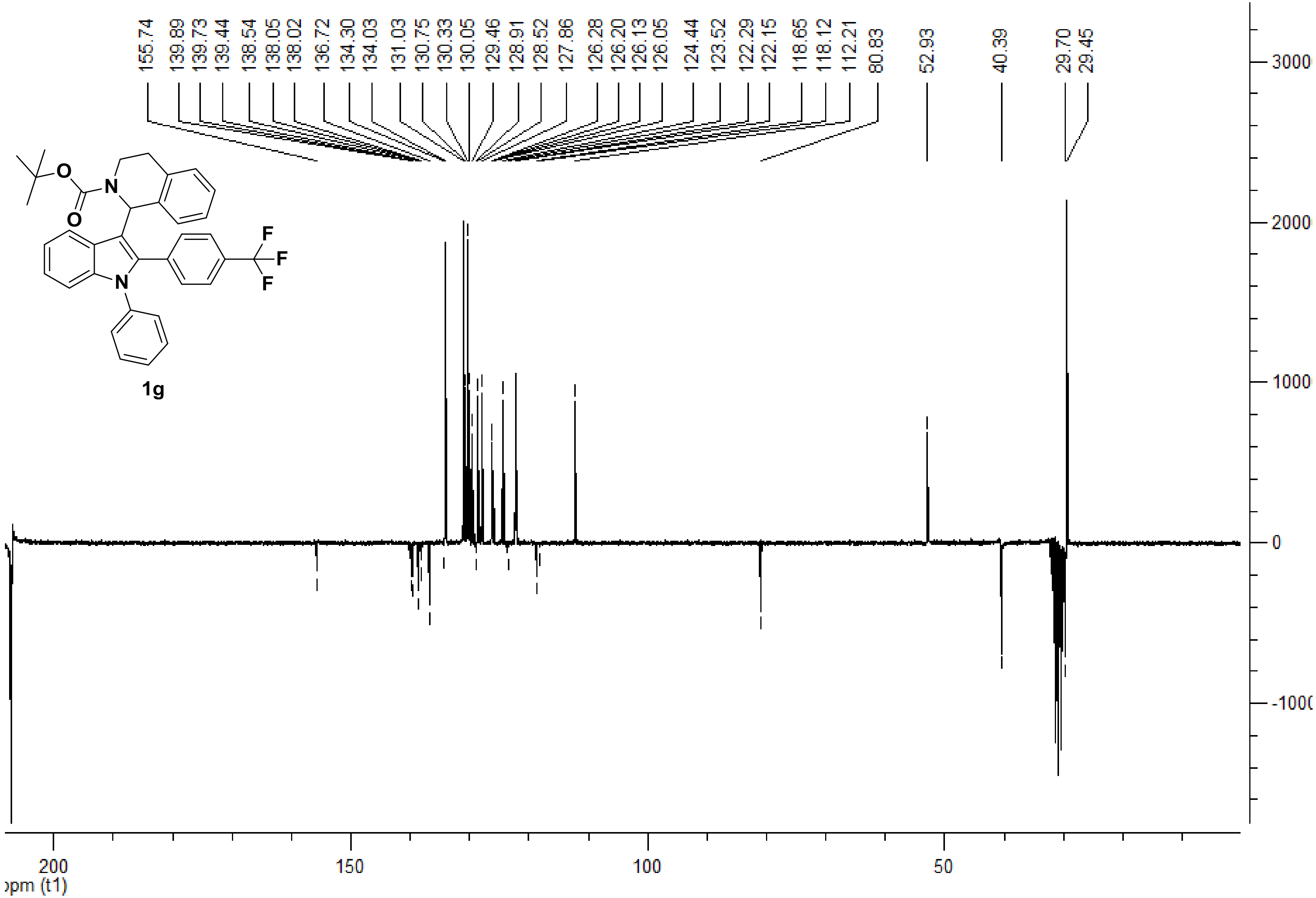

155.74
139.89
139.73
139.44
138.54
138.05
138.02
136.72
134.30
134.03
131.03
130.75
130.33
130.05
129.46
128.91
128.52
127.86
126.28
126.20
126.13
126.05
124.44
123.52
122.29
122.15
118.65
118.12
112.21
80.83
52.93
40.39
29.70
29.45
1g
3000
2000
1000
0
-1000
200
150
100
50
ppm (t1)

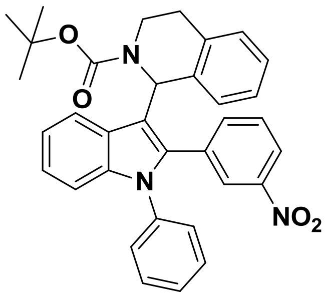

1h

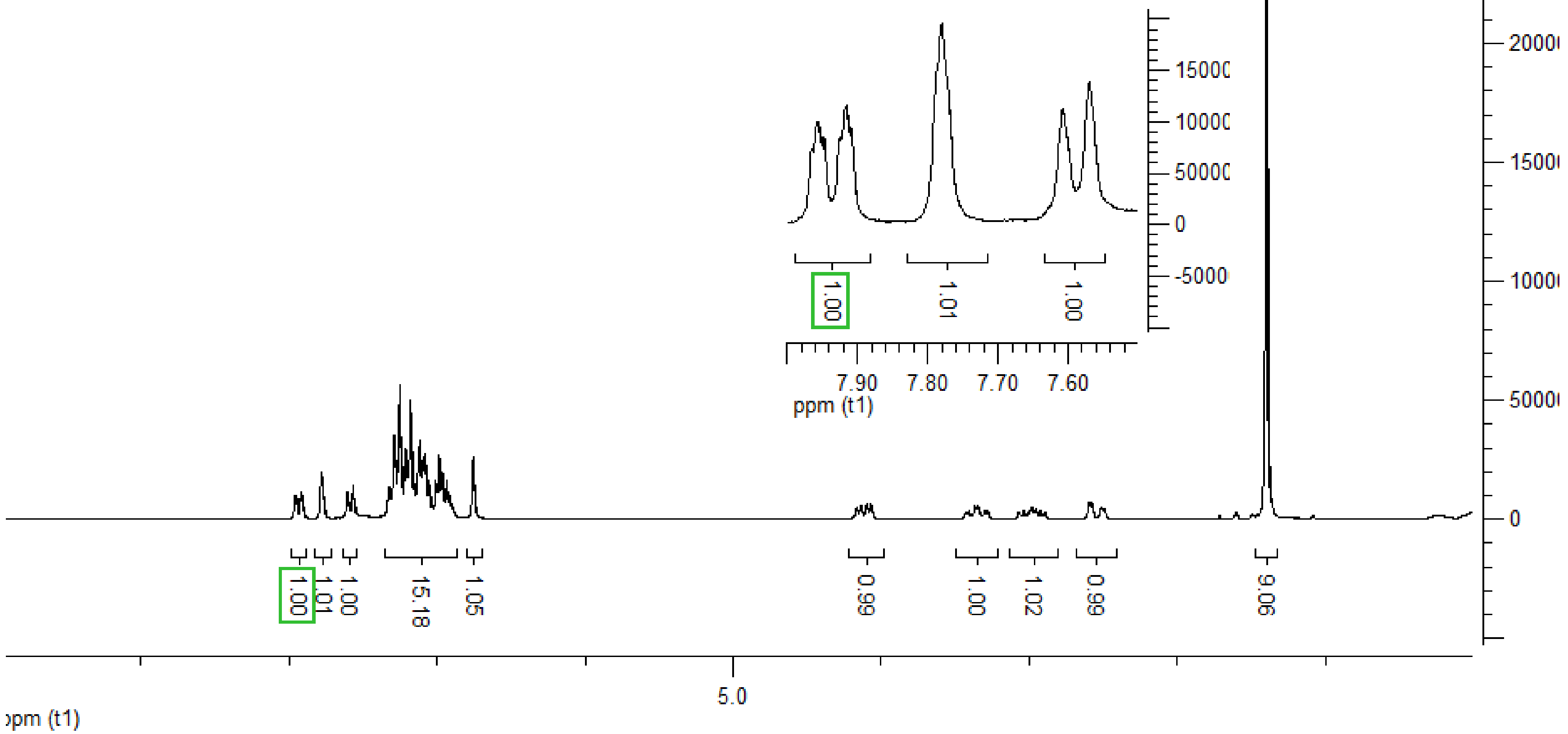

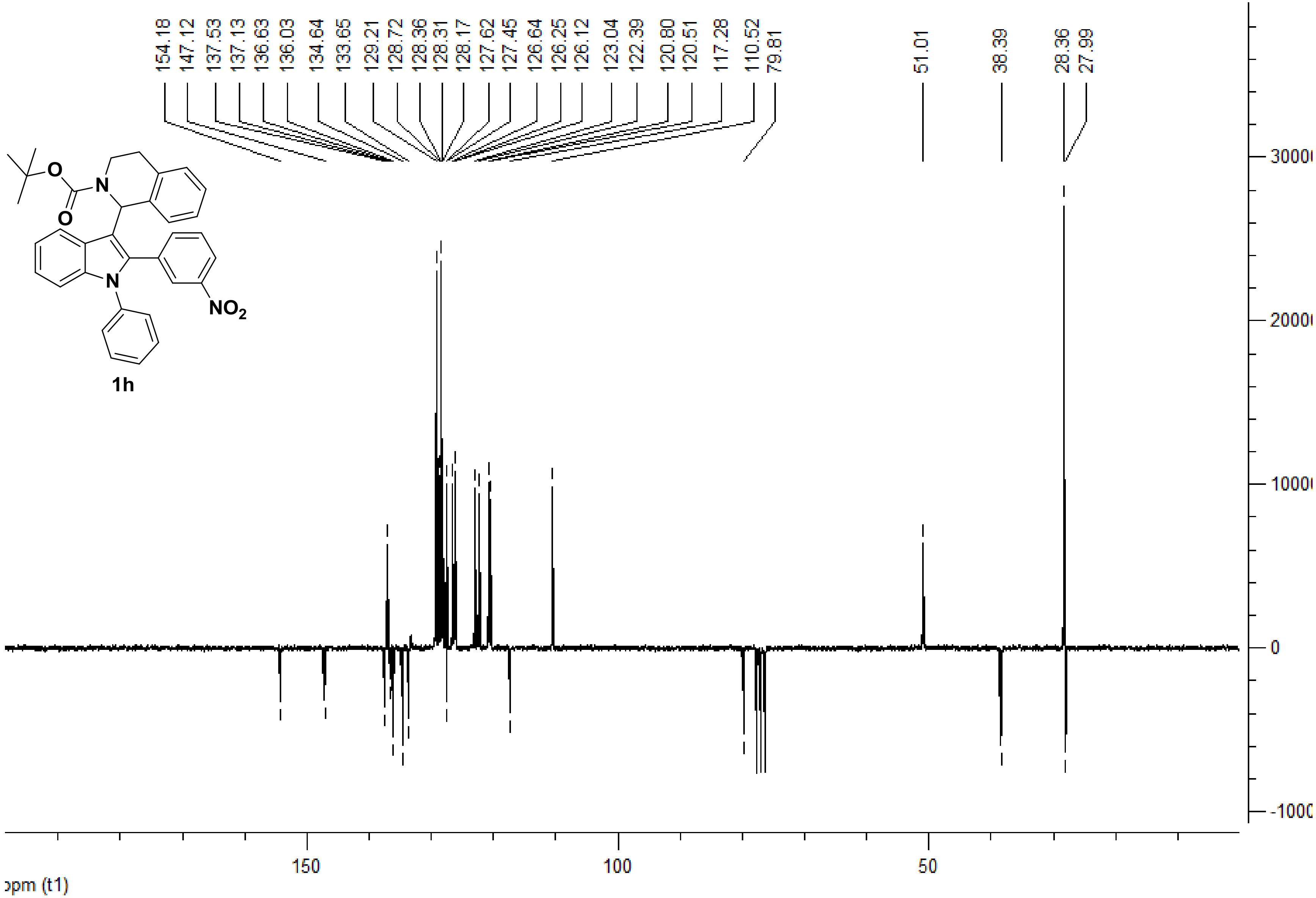
O
N
O
N
NO2
1h
154.18
147.12
137.53
137.13
136.63
136.03
134.64
133.65
129.21
128.72
128.36
128.31
128.17
127.62
127.45
126.64
126.25
126.12
123.04
122.39
120.80
120.51
117.28
110.52
79.81
51.01
38.39
28.36
27.99
30000
20000
10000
0
-1000
150
100
50
ppm (t1)

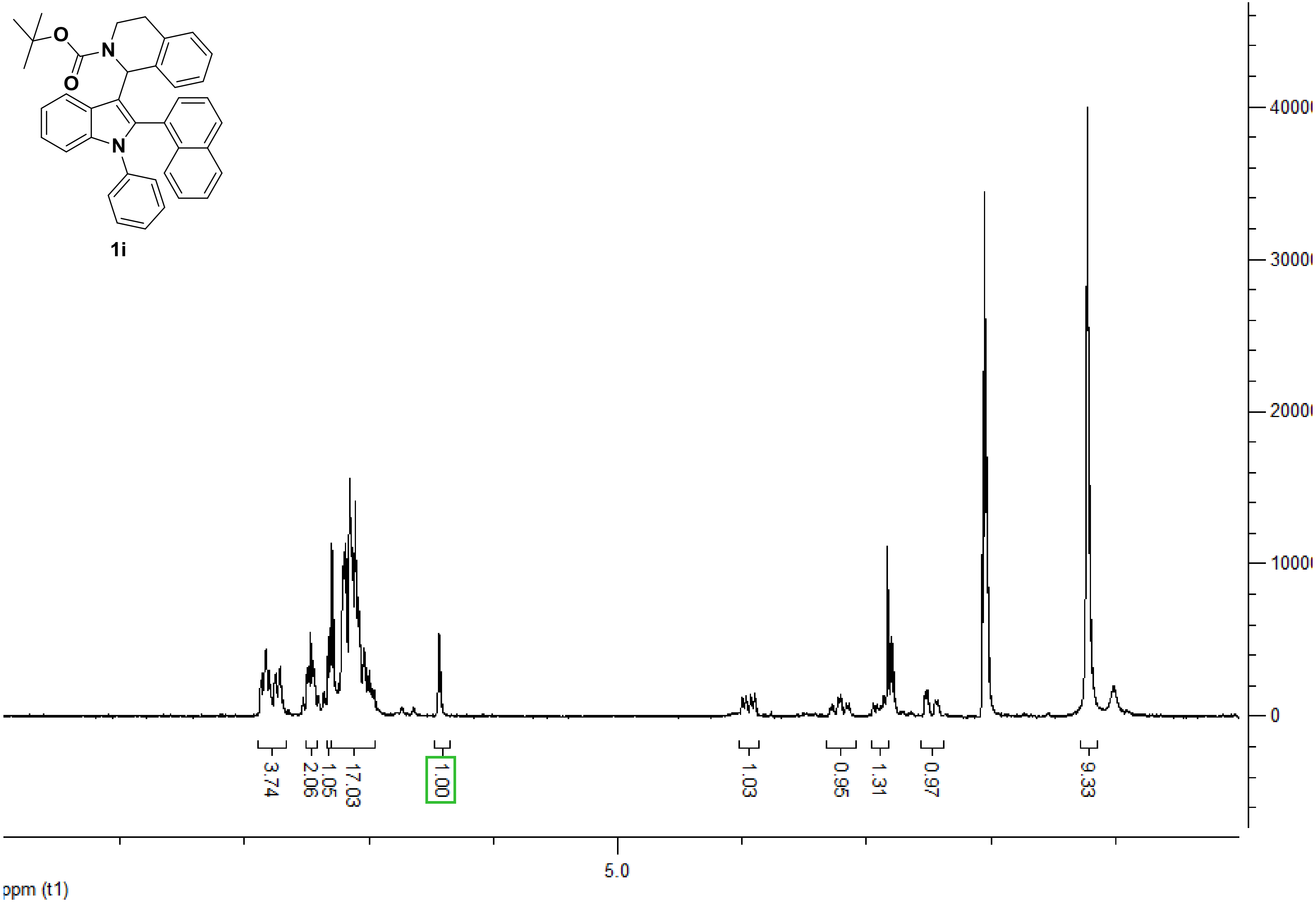
O
N
O
N
1i
40000
30000
20000
10000
0
3.74
2.06
1.05
17.03
1.00
1.03
0.95
1.31
0.97
9.33
5.0
ppm (t1)

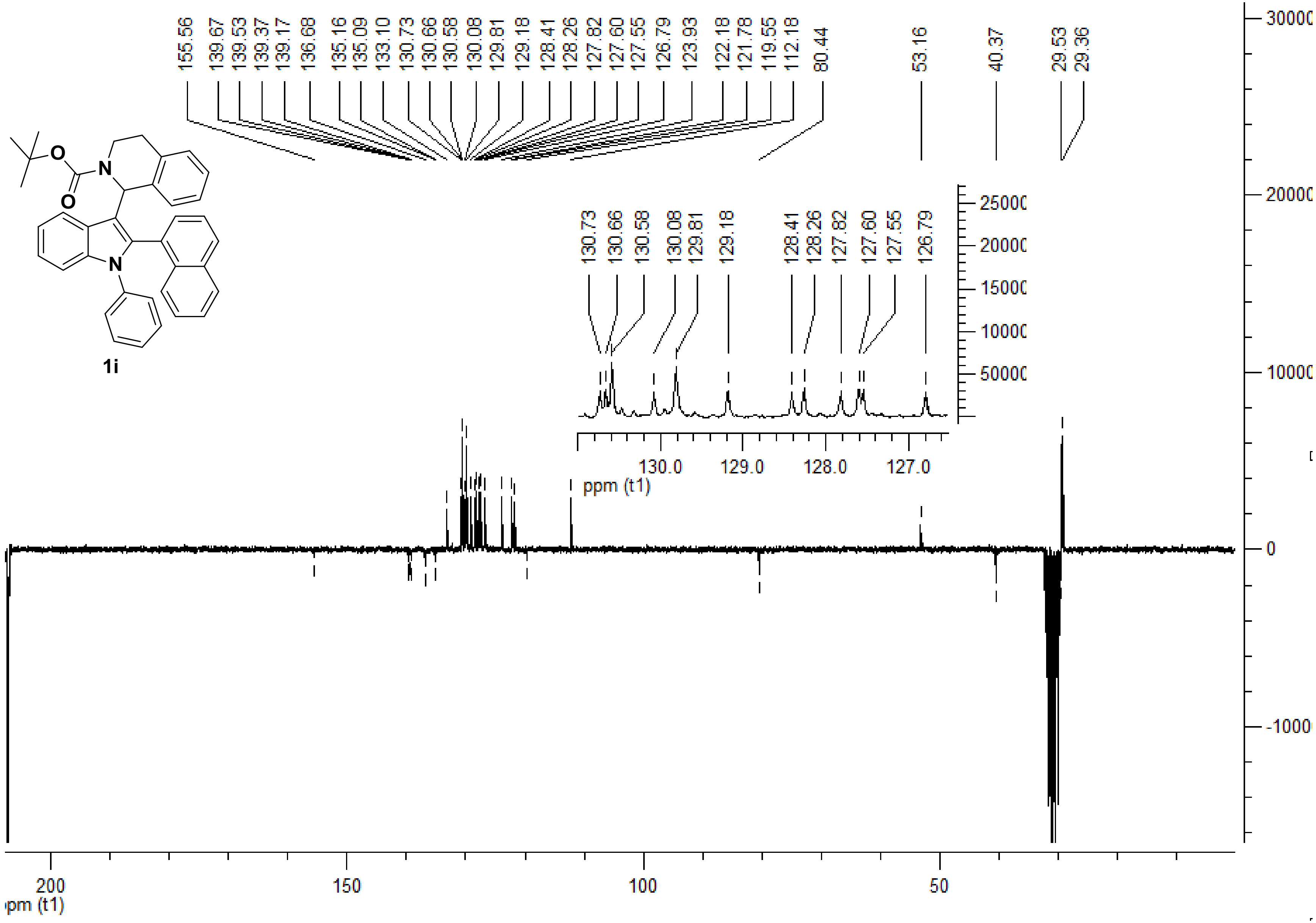
155.56
139.67
139.53
139.37
139.17
136.68
135.16
135.09
133.10
130.73
130.66
130.58
130.08
129.81
129.18
128.41
128.26
127.82
127.60
127.55
126.79
123.93
122.18
121.78
119.55
112.18
80.44
53.16
40.37
29.53
29.36
1i
ppm (t1)

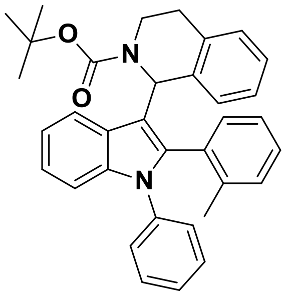

1j

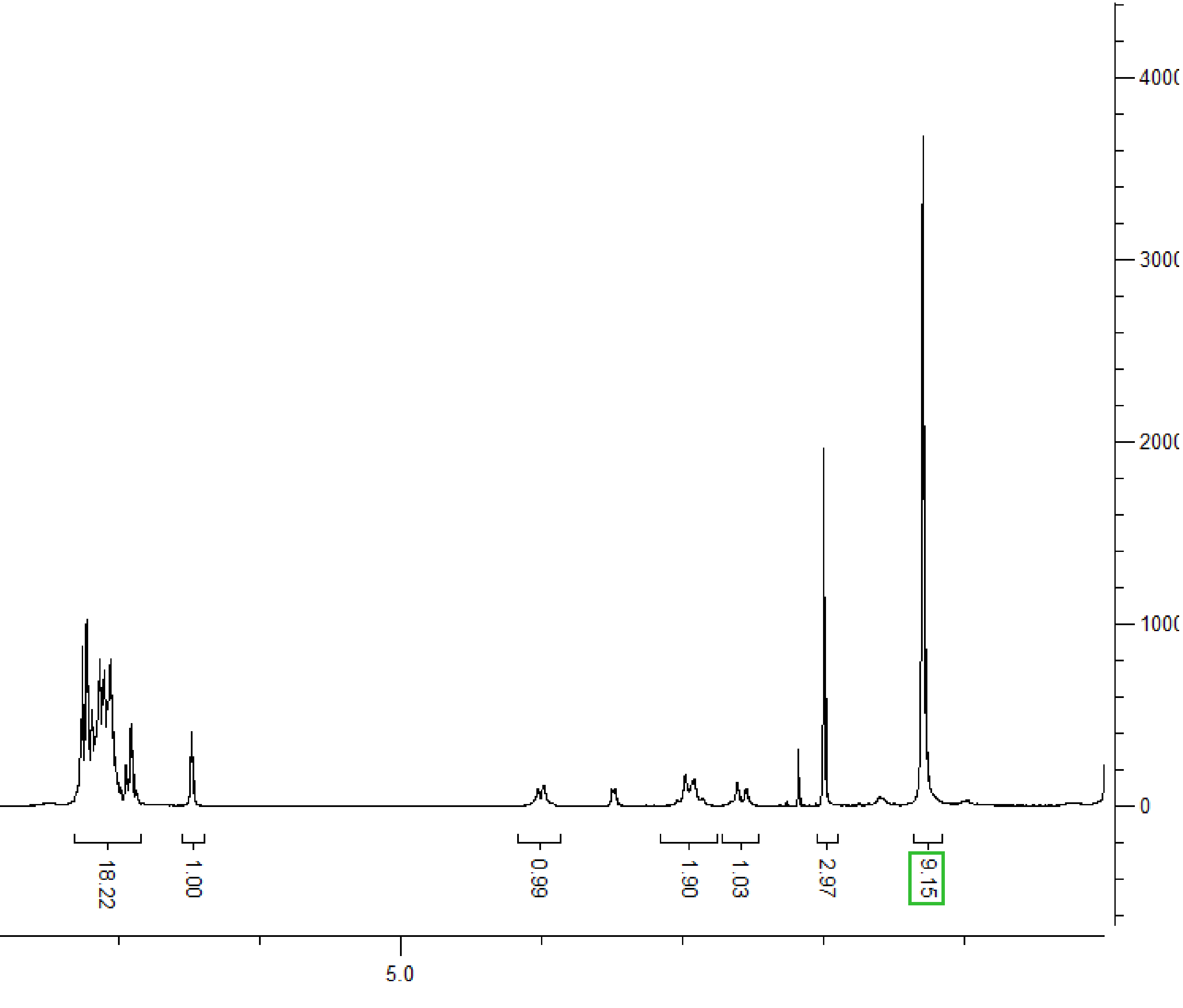

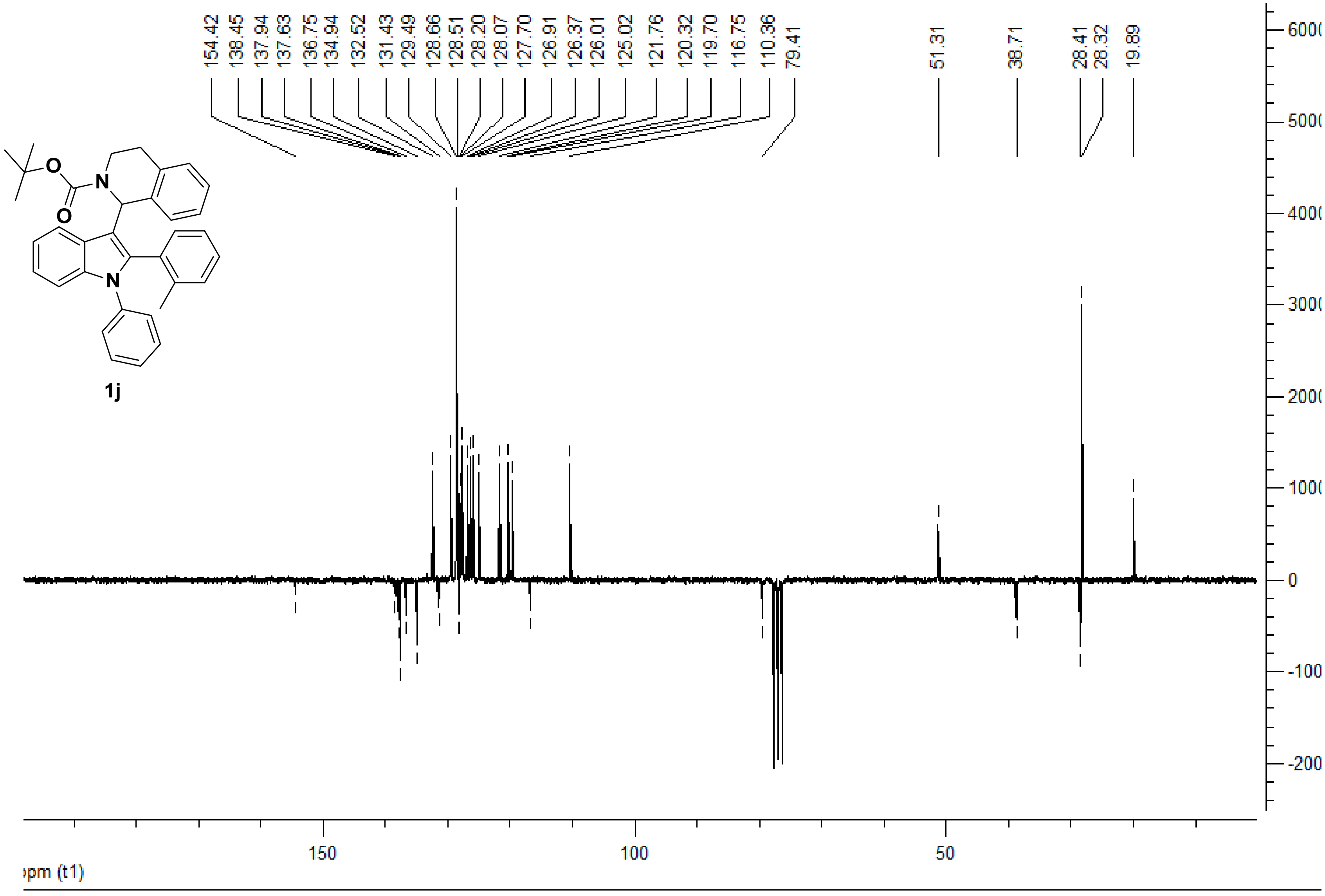

154.42
138.45
137.94
137.63
136.75
134.94
132.52
131.43
129.49
128.66
128.51
128.20
128.07
127.70
126.91
126.37
126.01
125.02
121.76
120.32
119.70
116.75
110.36
79.41
51.31
38.71
28.41
28.32
19.89
O
N
O
N
150
100
50
ppm (t1)

1j

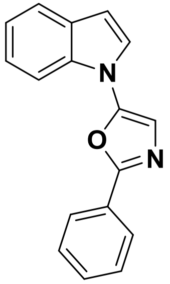

5m

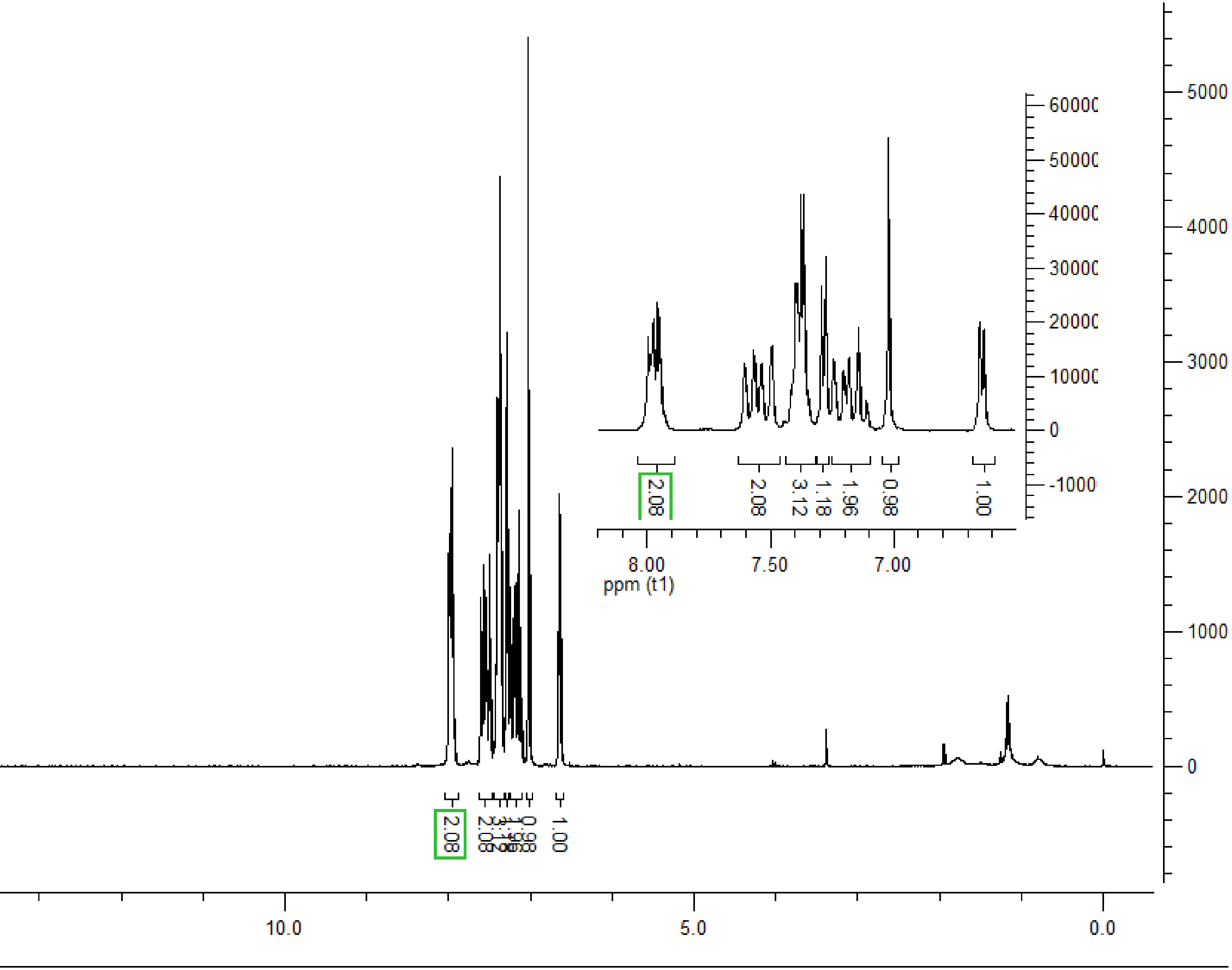

5000
4000
3000
2000
1000
0
60000
50000
40000
30000
20000
10000
0
-1000
2.08
2.08
3.12
1.18
1.96
0.98
1.00
8.00
7.50
7.00
ppm (t1)
15.0
10.0
5.0
0.0
ppm (t1)

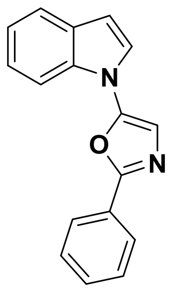

5m

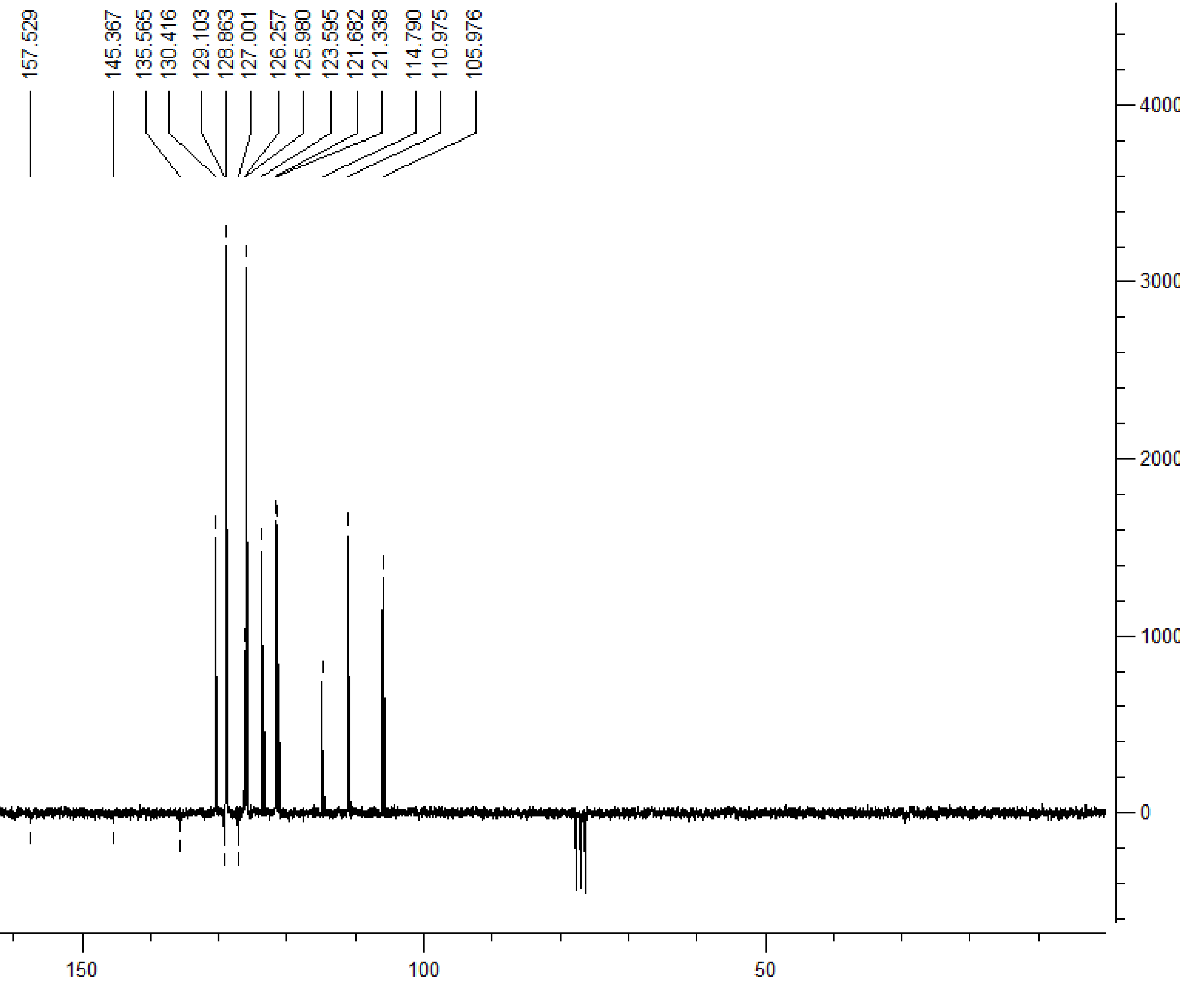
157.529
145.367
135.565
130.416
129.103
128.863
127.001
126.257
125.980
123.595
121.682
121.338
114.790
110.975
105.976
4000
3000
2000
1000
0
150
100
50
ppm (t1)

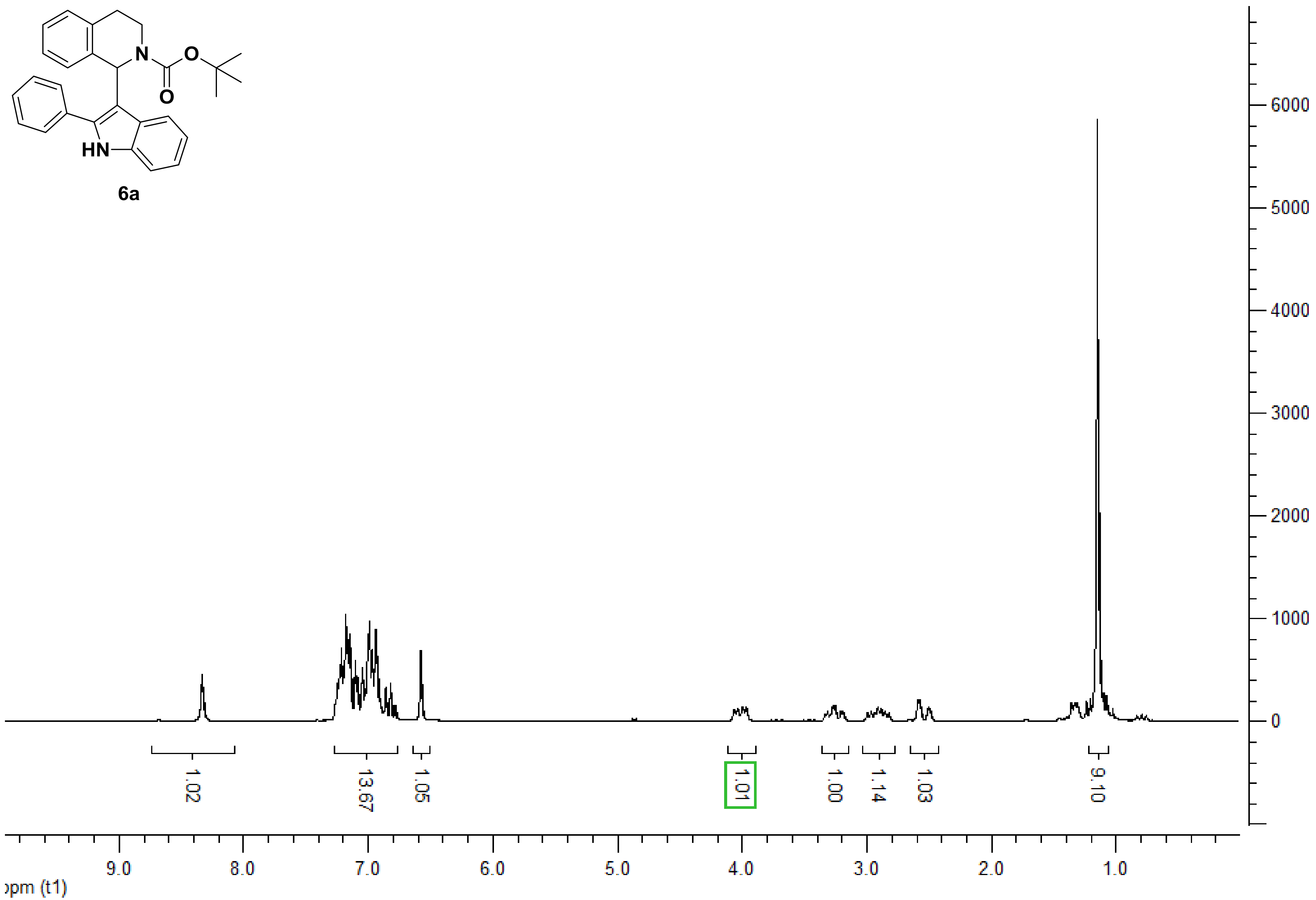
N
O
O
HN
6a
6000
5000
4000
3000
2000
1000
0
1.02
13.67
1.05
1.01
1.00
1.14
1.03
9.10
9.0
8.0
7.0
6.0
5.0
4.0
3.0
2.0
1.0
ppm (t1)

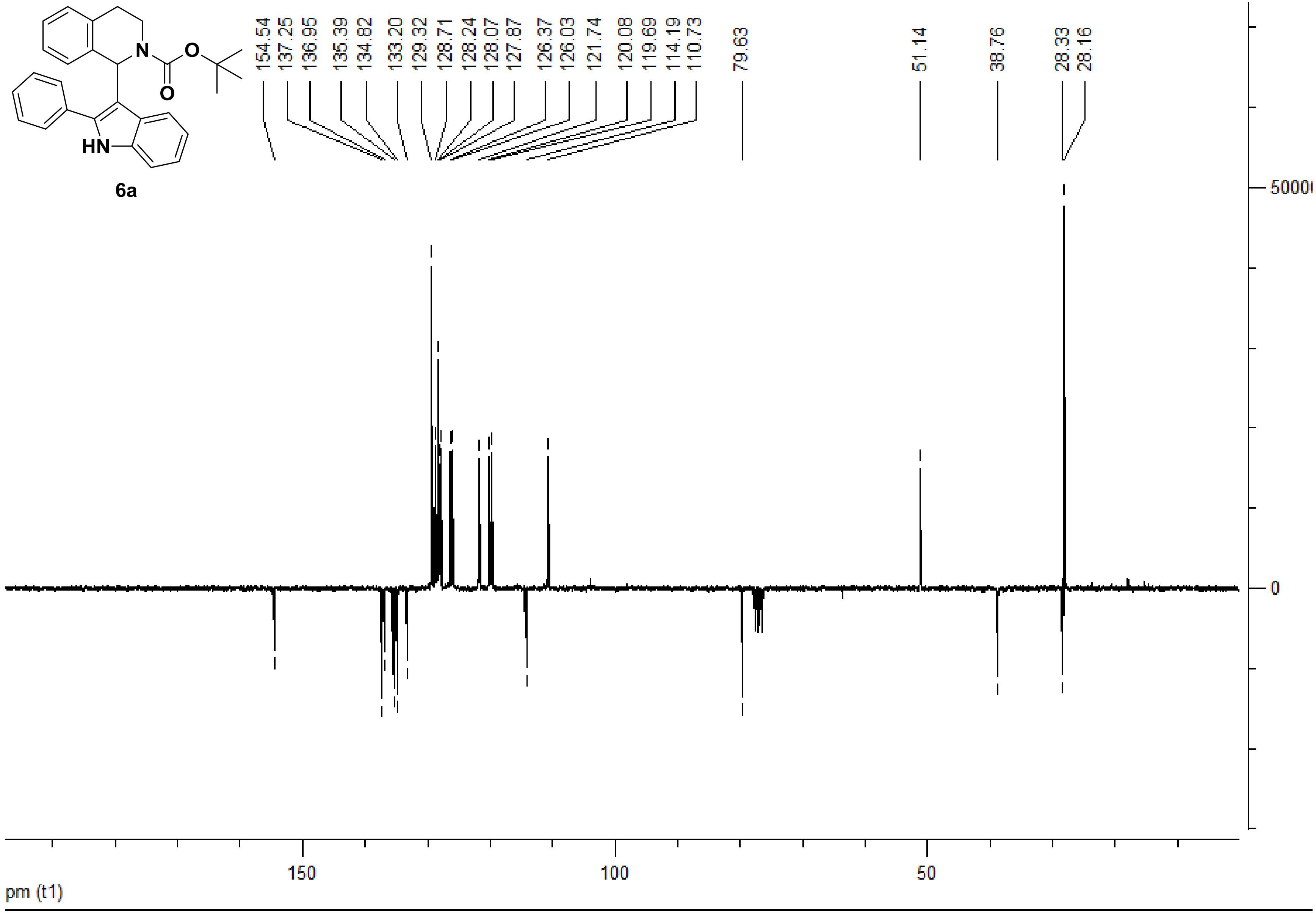
6a
154.54
137.25
136.95
135.39
134.82
133.20
129.32
128.71
128.24
128.07
127.87
126.37
126.03
121.74
120.08
119.69
114.19
110.73
79.63
51.14
38.76
28.33
28.16
50000
0
150
100
50
pm (t1)

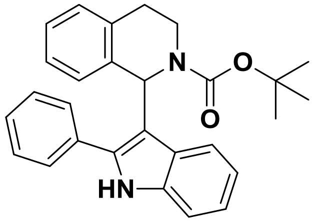

6a

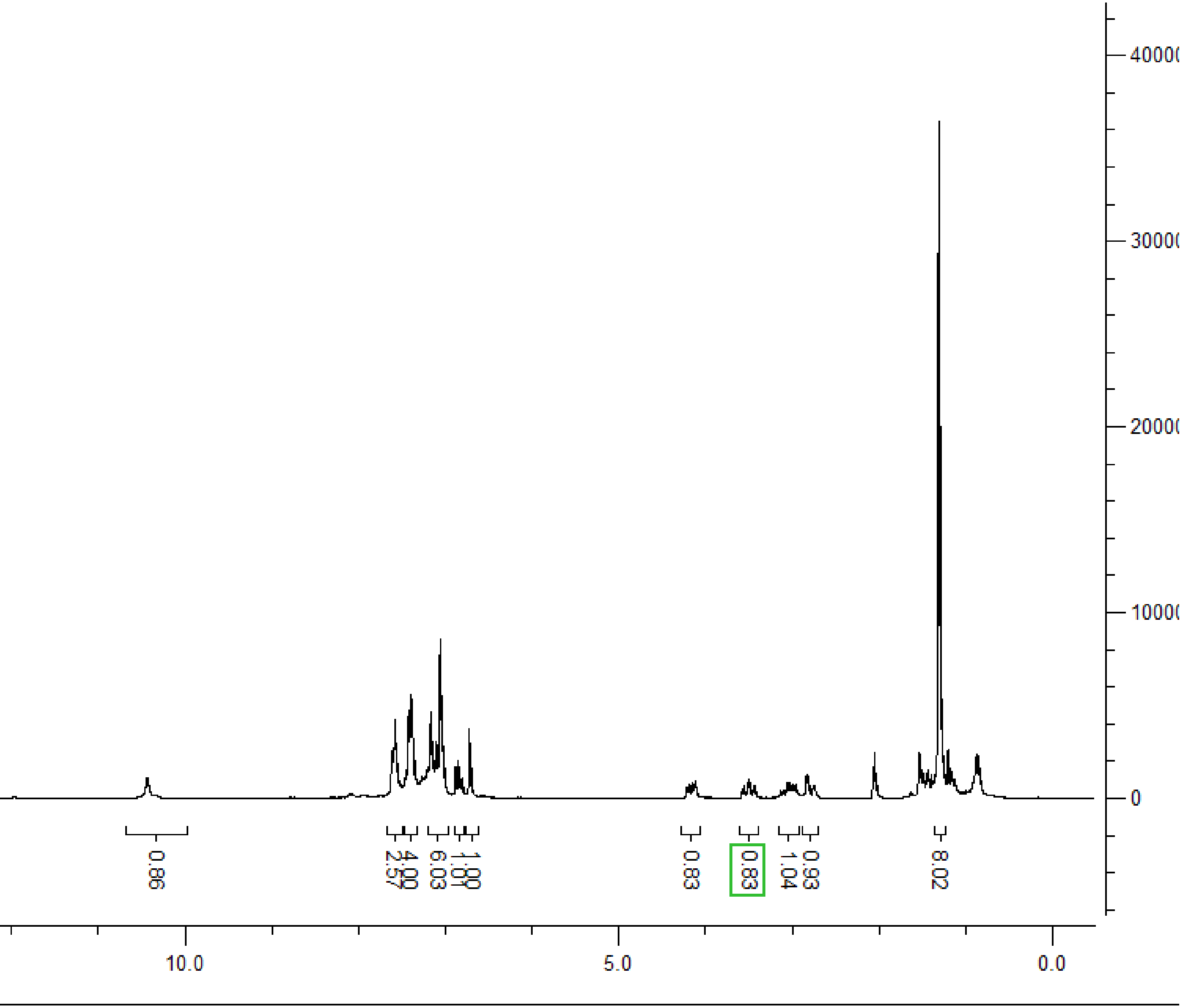

40000
30000
20000
10000
0
0.86
2.57
4.00
6.03
1.07
1.00
0.83
0.83
1.04
0.93
8.02
15.0
10.0
5.0
0.0
ppm (t1)

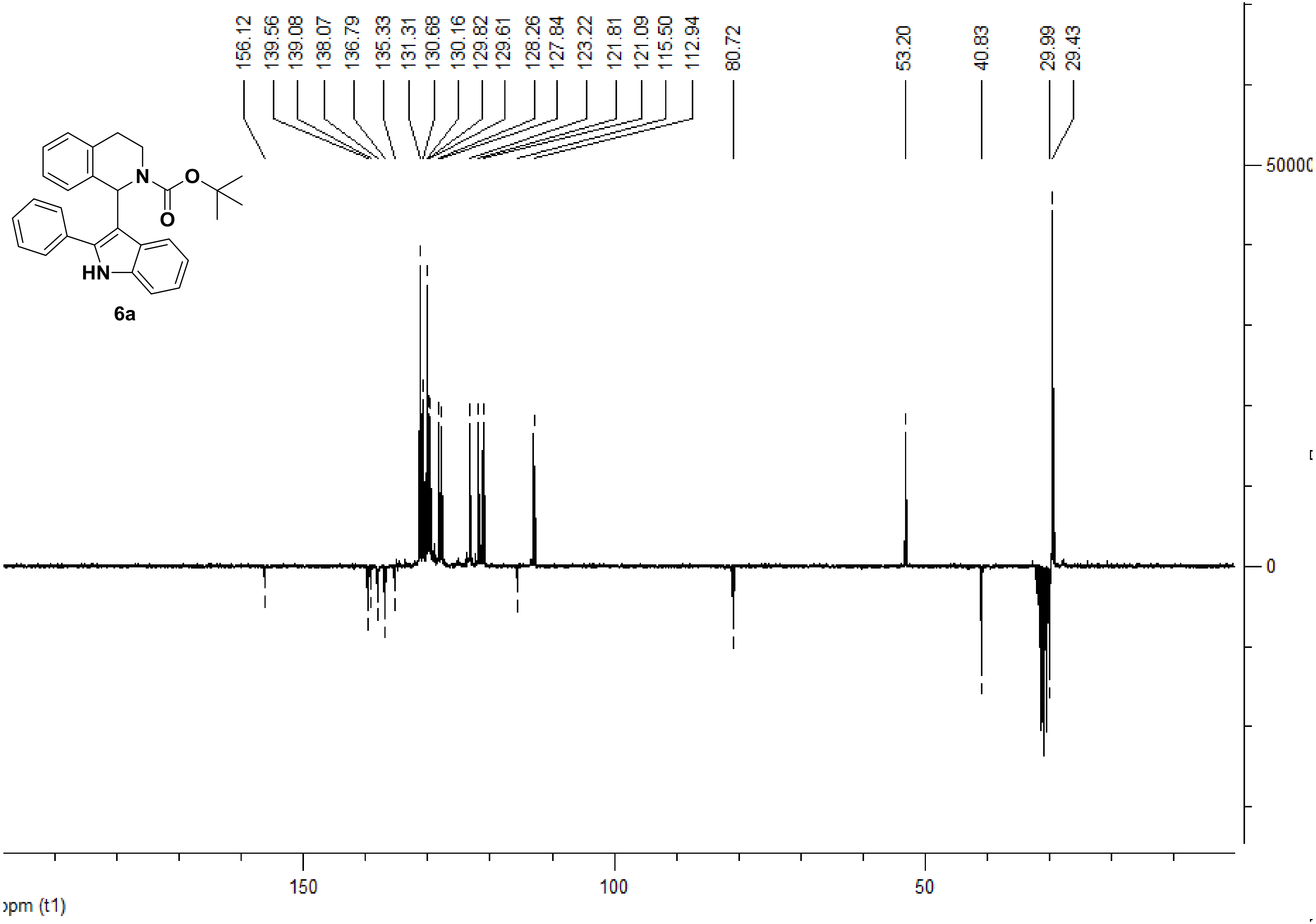
156.12
139.56
139.08
138.07
136.79
135.33
131.31
130.68
130.16
129.82
129.61
128.26
127.84
123.22
121.81
121.09
115.50
112.94
80.72
53.20
40.83
29.99
29.43
N
O
O
HN
6a
50000
0
150
100
50
ppm (t1)

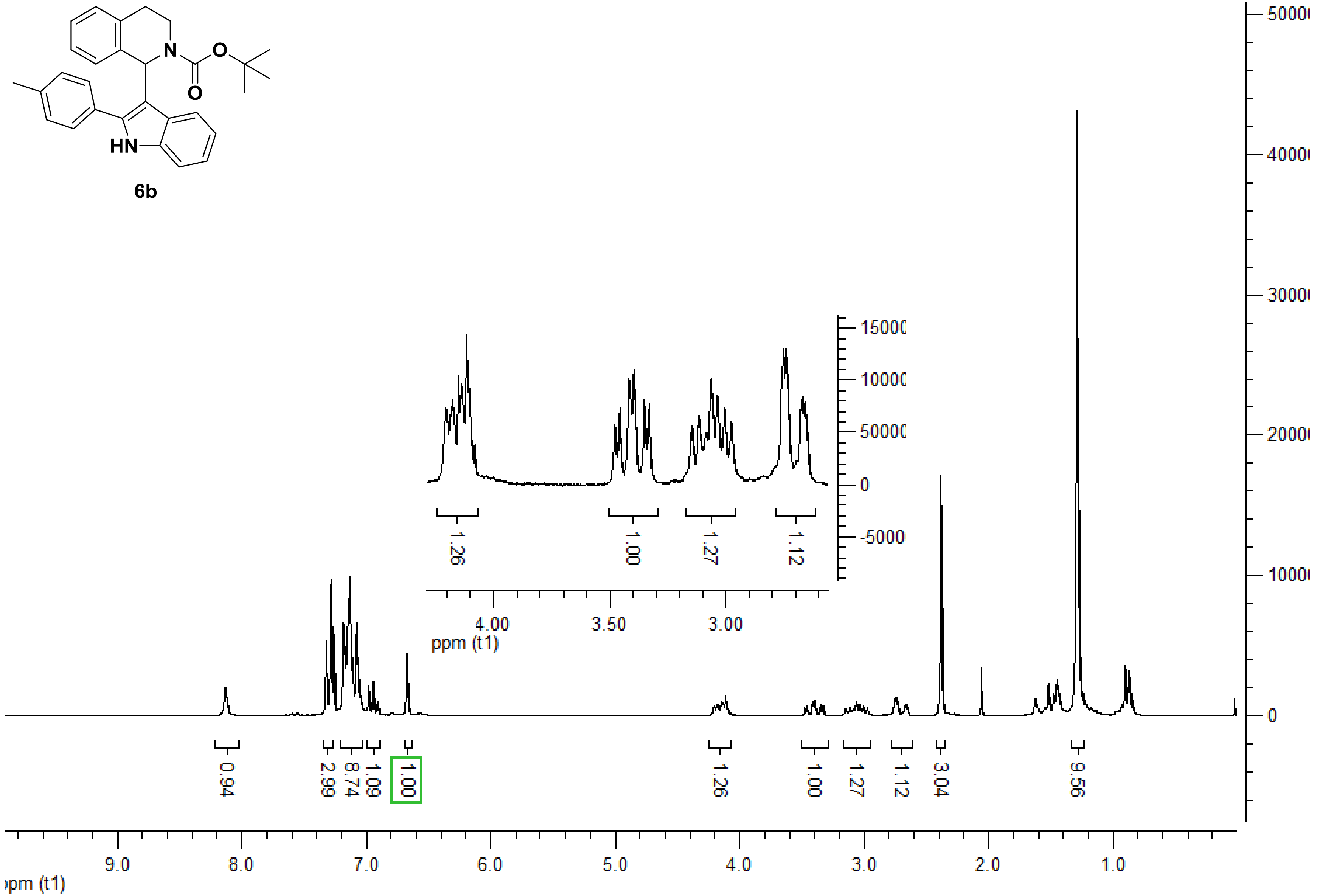

6b
0.94
2.99
8.74
1.09
1.00
1.26
1.00
1.27
1.12
3.04
9.56
ppm (t1)

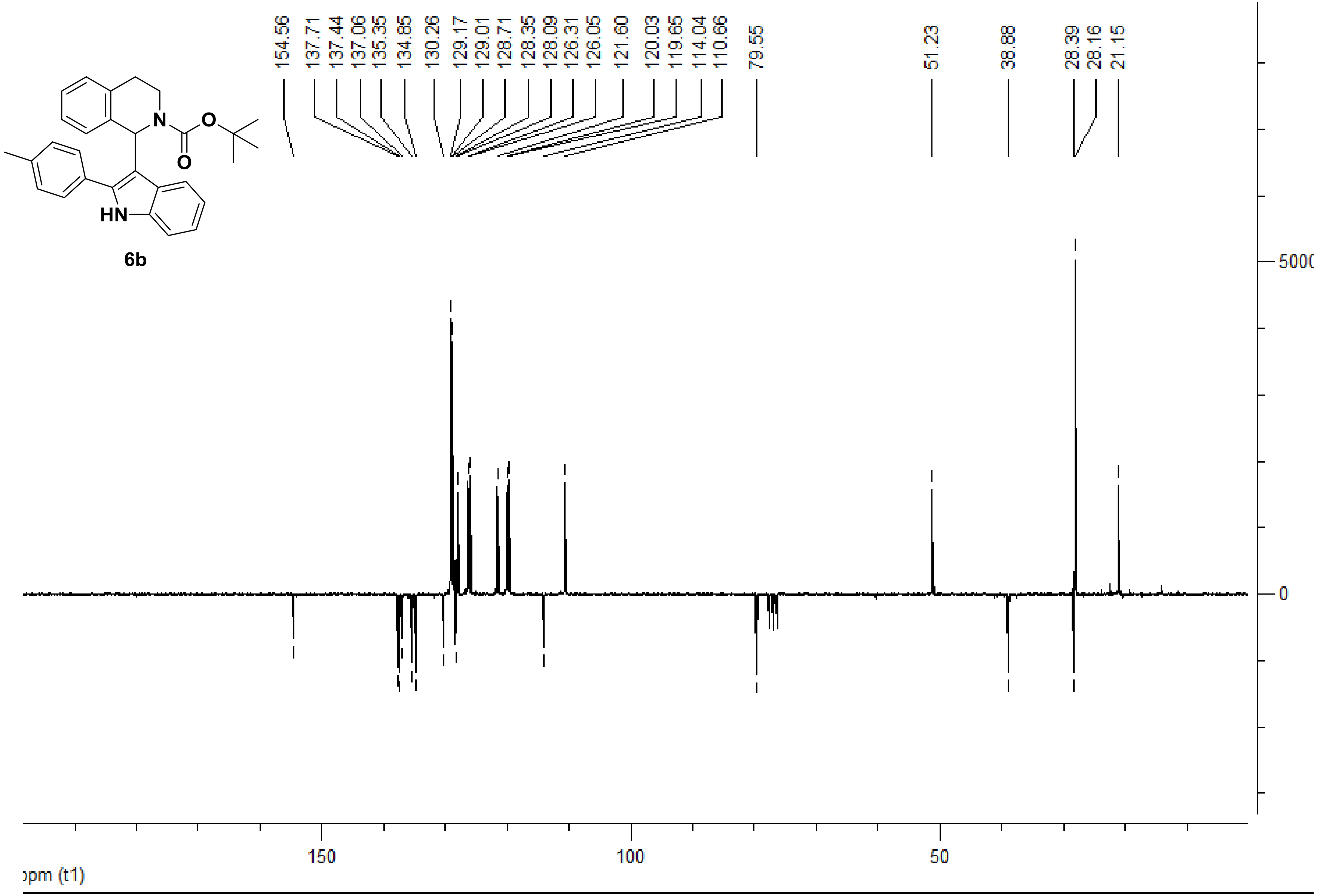
154.56
137.71
137.44
137.06
135.35
134.85
130.26
129.17
129.01
128.71
128.35
128.09
126.31
126.05
121.60
120.03
119.65
114.04
110.66
79.55
51.23
38.88
28.39
28.16
21.15
N
O
O
HN
6b
5000
0
150
100
50
ppm (t1)

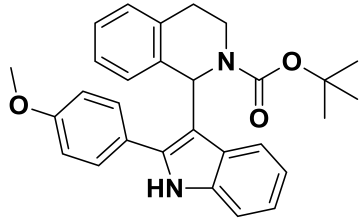

6c

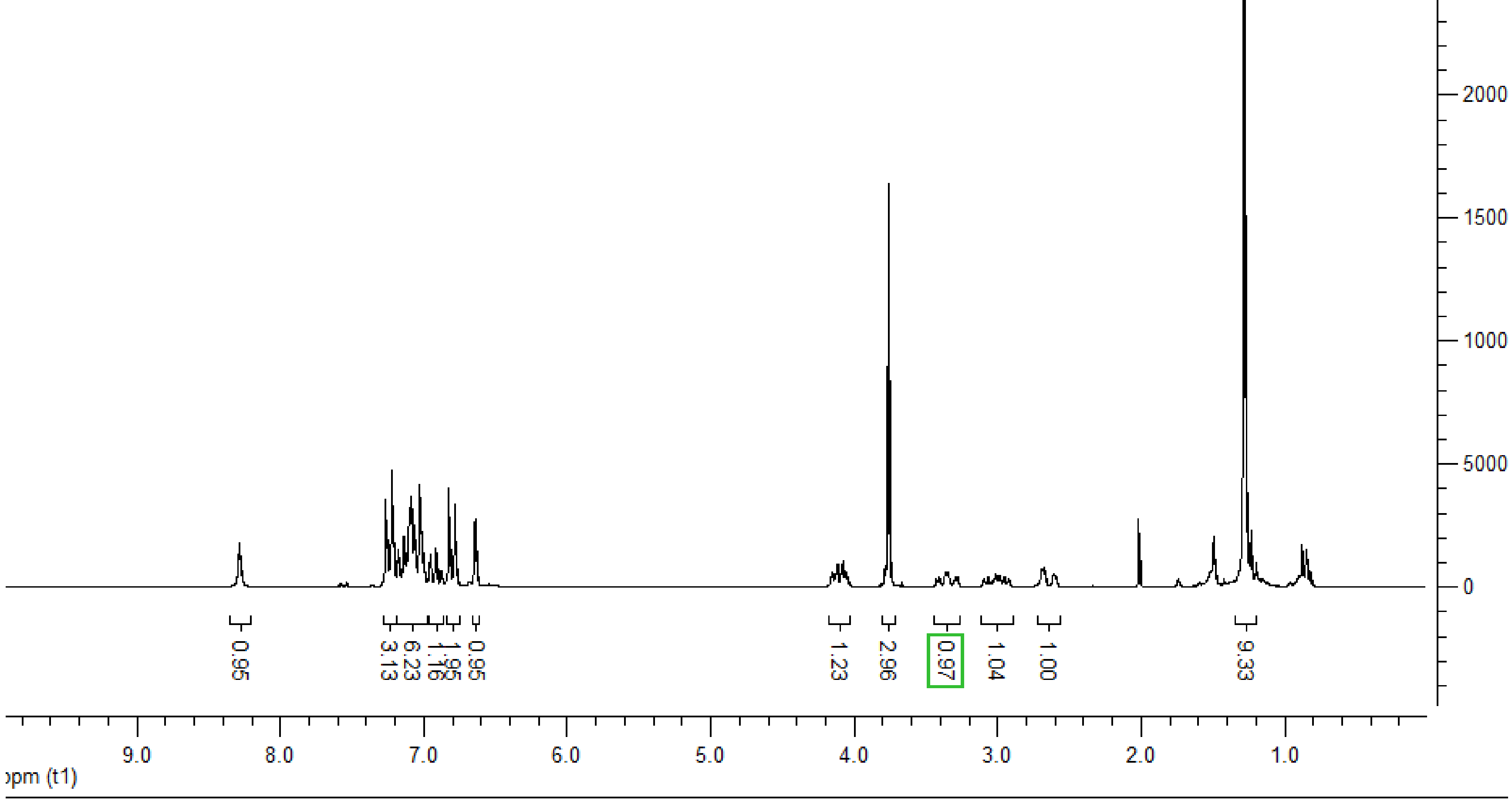

0.95
3.13
6.23
1.16
1.95
0.95
1.23
2.96
0.97
1.04
1.00
9.33
9.0
8.0
7.0
6.0
5.0
4.0
3.0
2.0
1.0
ppm (t1)
3000
2500
2000
1500
1000
5000
0

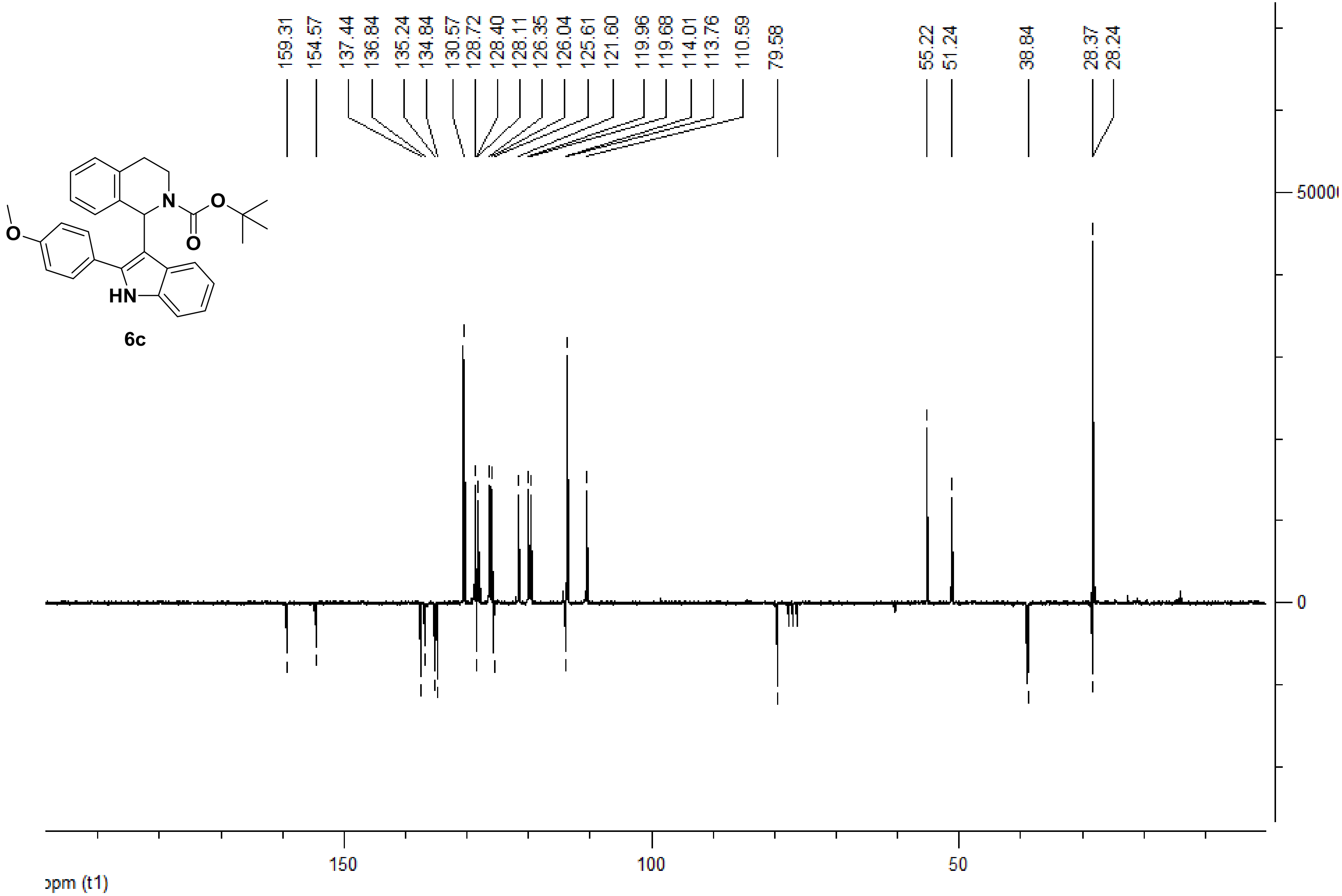

N
O
O
O
HN
6c
159.31
154.57
137.44
136.84
135.24
134.84
130.57
128.72
128.40
128.11
126.35
126.04
125.61
121.60
119.96
119.68
114.01
113.76
110.59
79.58
55.22
51.24
38.84
28.37
28.24
50000
0
150
100
50
ppm (t1)

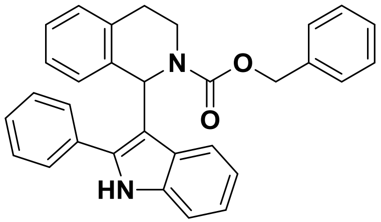

N
O
O
HN

6f

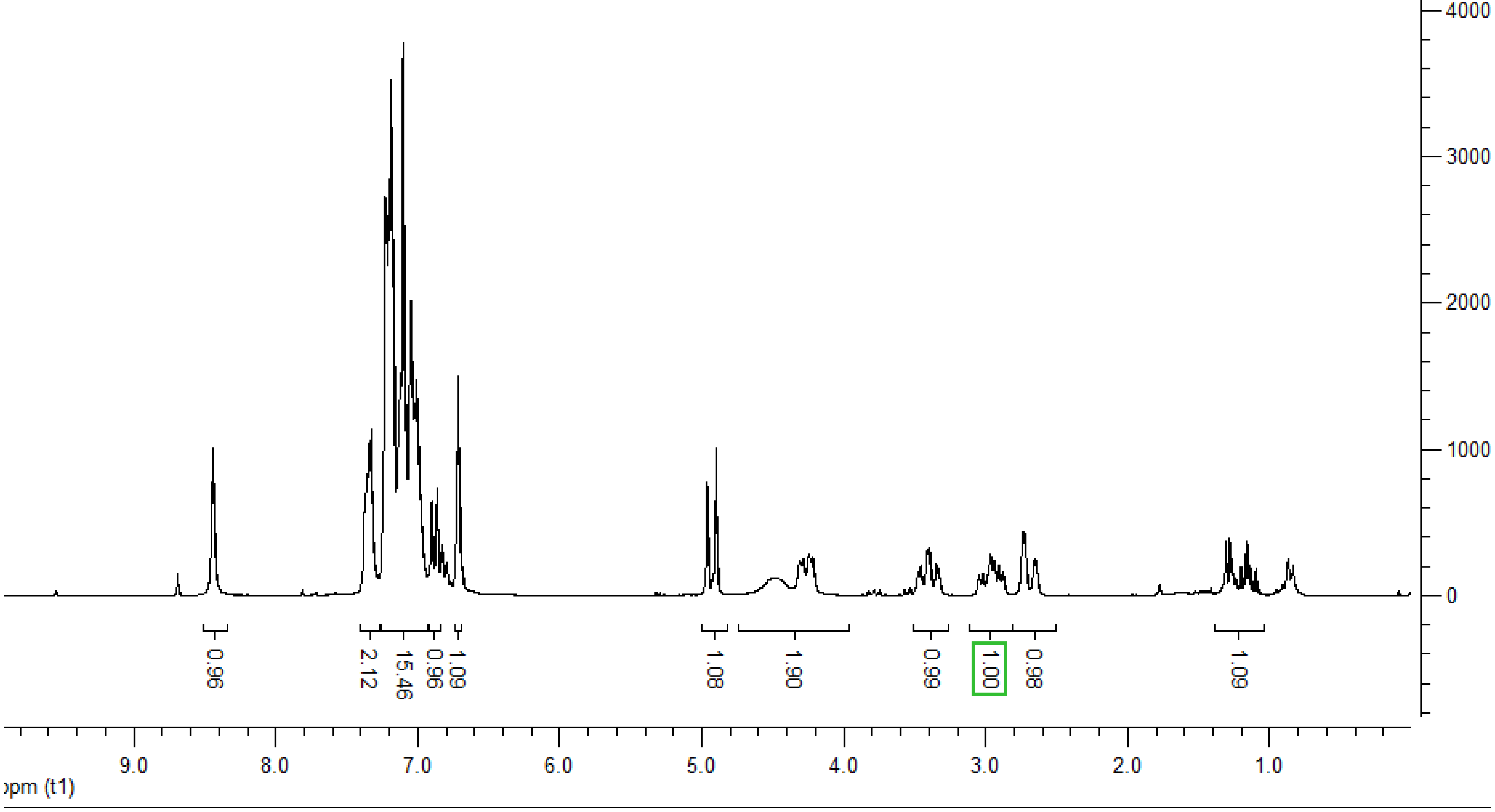

5000
4000
3000
2000
1000
0
0.96
2.12
15.46
0.96
1.09
1.08
1.90
0.99
1.00
0.98
1.09
9.0
8.0
7.0
6.0
5.0
4.0
3.0
2.0
1.0
ppm (t1)

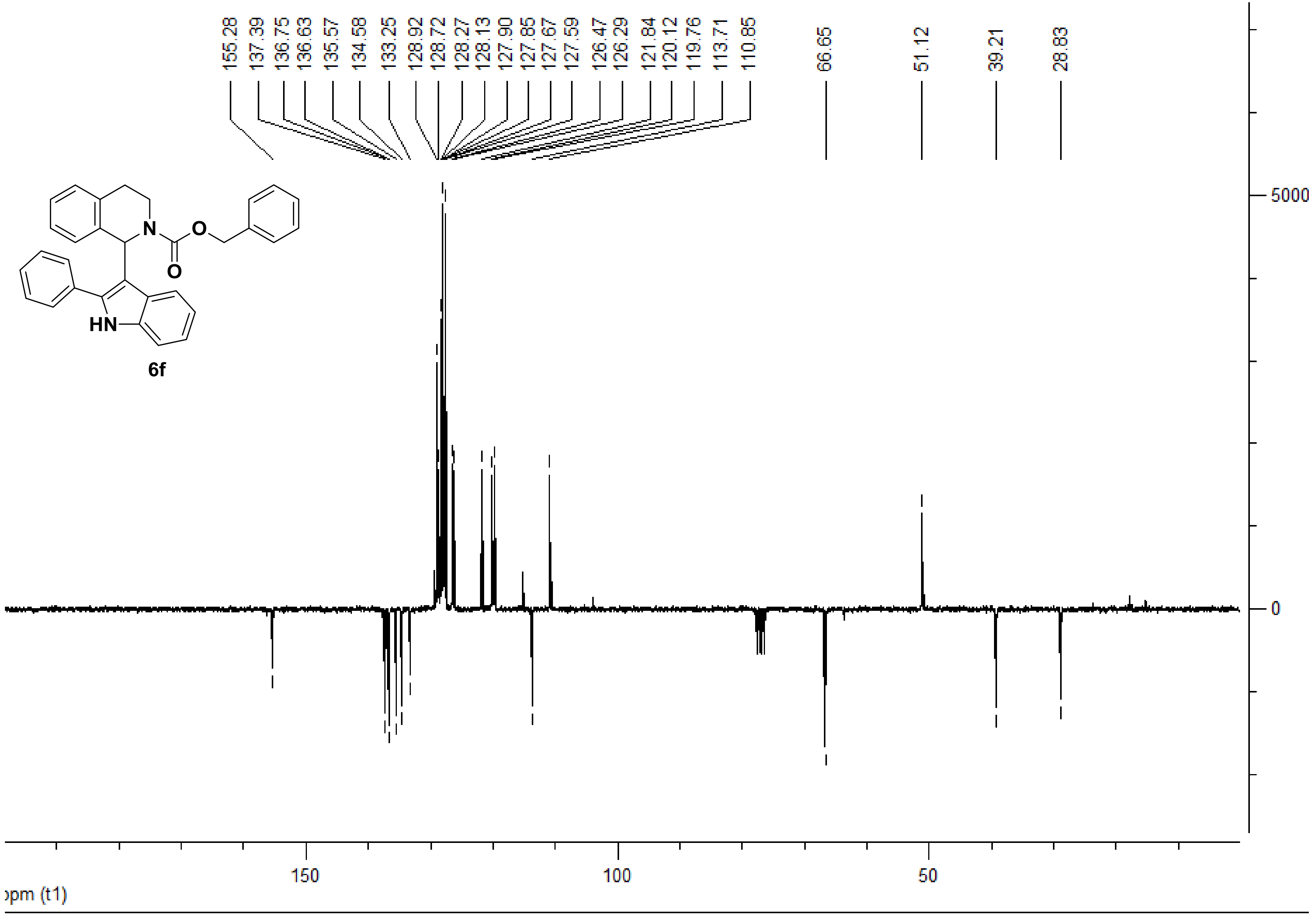
155.28
137.39
136.75
136.63
135.57
134.58
133.25
128.92
128.72
128.27
128.13
127.90
127.85
127.67
127.59
126.47
126.29
121.84
120.12
119.76
113.71
110.85
66.65
51.12
39.21
28.83
N
O
O
HN
6f
5000
0
150
100
50
ppm (t1)

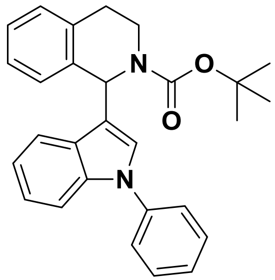

7a

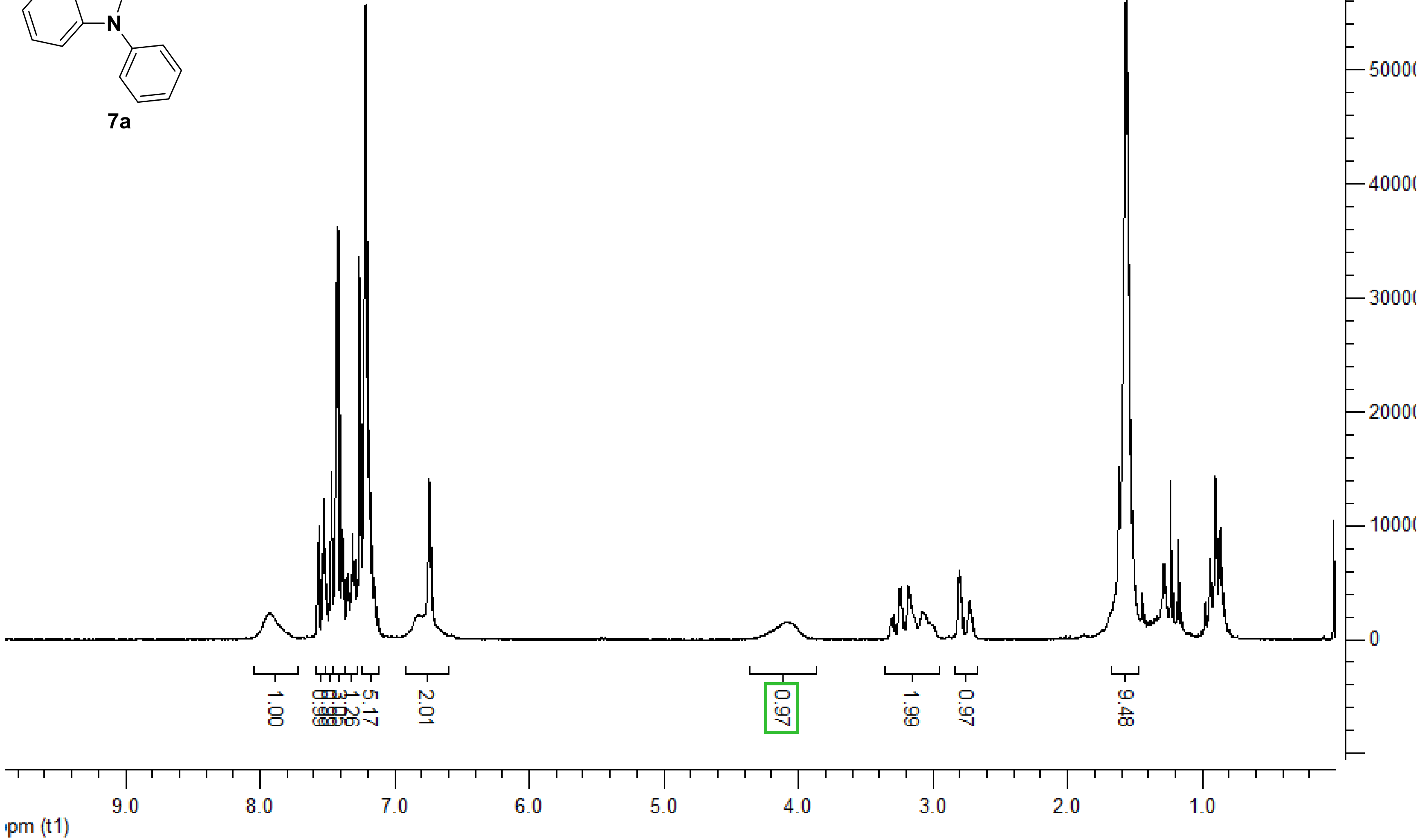

60000
50000
40000
30000
20000
10000
0
1.00
1.26
5.17
2.01
0.97
1.99
0.97
9.48
9.0
8.0
7.0
6.0
5.0
4.0
3.0
2.0
1.0
pm (t1)

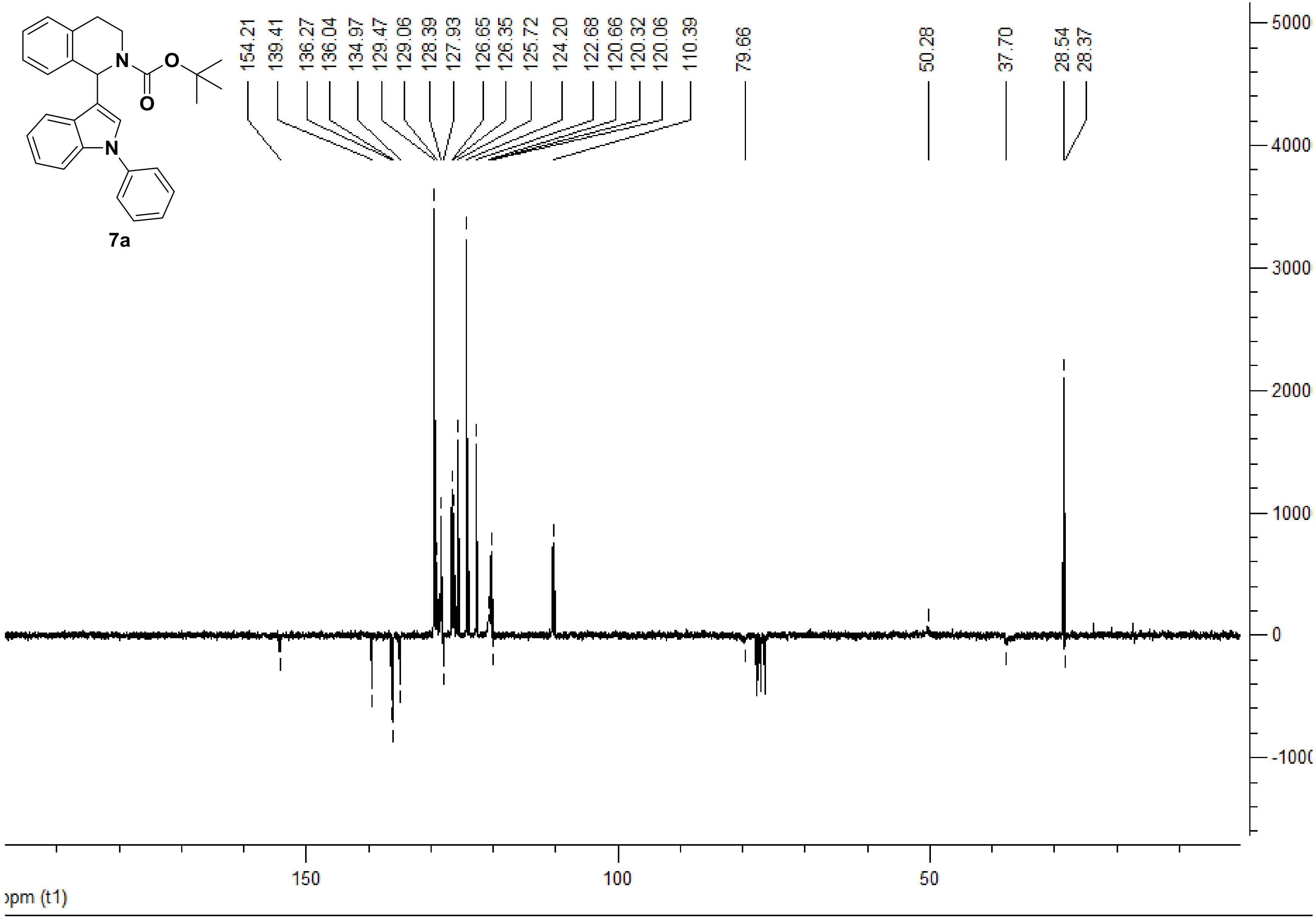
7a
154.21
139.41
136.27
136.04
134.97
129.47
129.06
128.39
127.93
126.65
126.35
125.72
124.20
122.68
120.66
120.32
120.06
110.39
79.66
50.28
37.70
28.54
28.37
5000
4000
3000
2000
1000
0
150
100
50
ppm (t1)

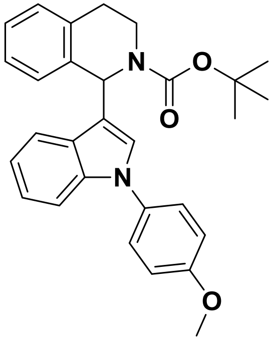

7b

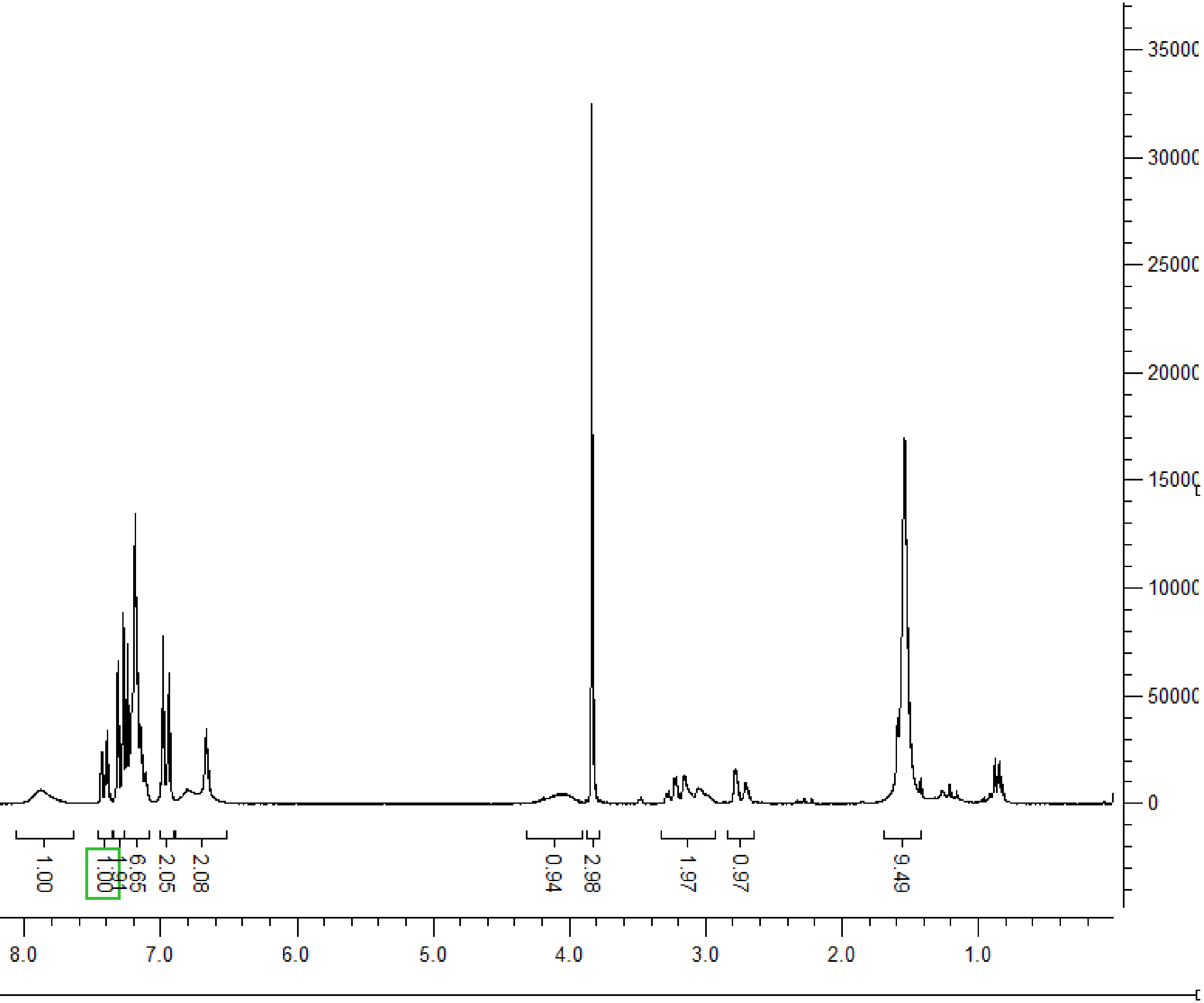

1.00
1.00
6.65
2.05
2.08
0.94
2.98
1.97
0.97
9.49
9.0
8.0
7.0
6.0
5.0
4.0
3.0
2.0
1.0
ppm (t1)

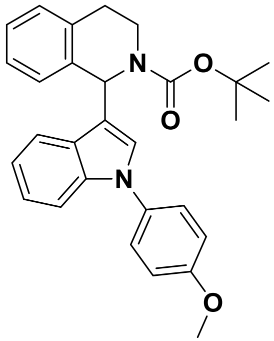

7b

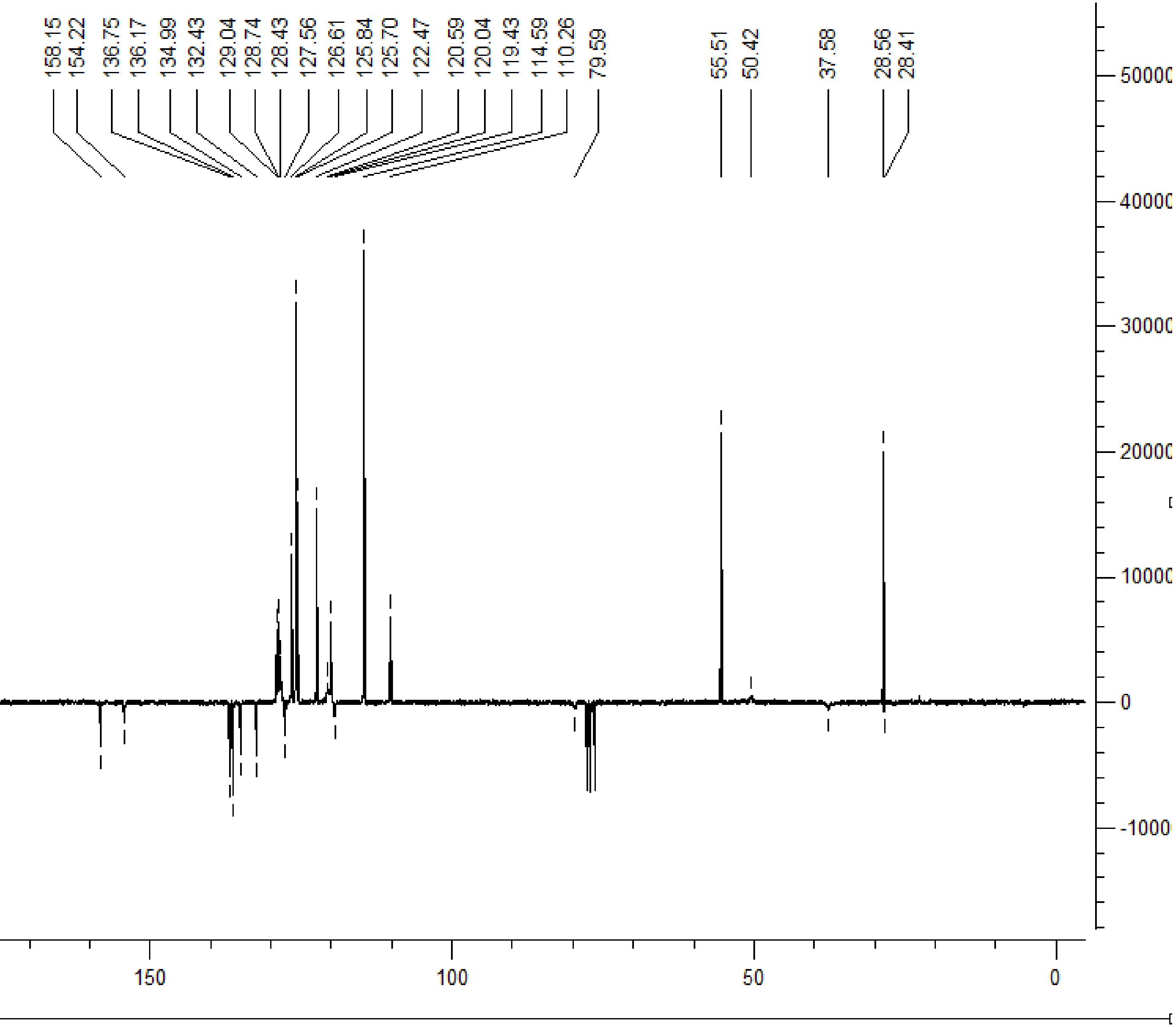
158.15
154.22
136.75
136.17
134.99
132.43
129.04
128.74
128.43
127.56
126.61
125.84
125.70
122.47
120.59
120.04
119.43
114.59
110.26
79.59
55.51
50.42
37.58
28.56
28.41
50000
40000
30000
20000
10000
0
-1000
200
150
100
50
0
ppm (t1)

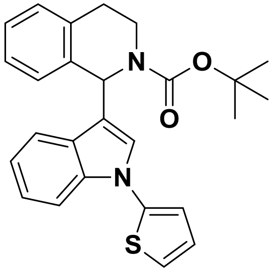

7c

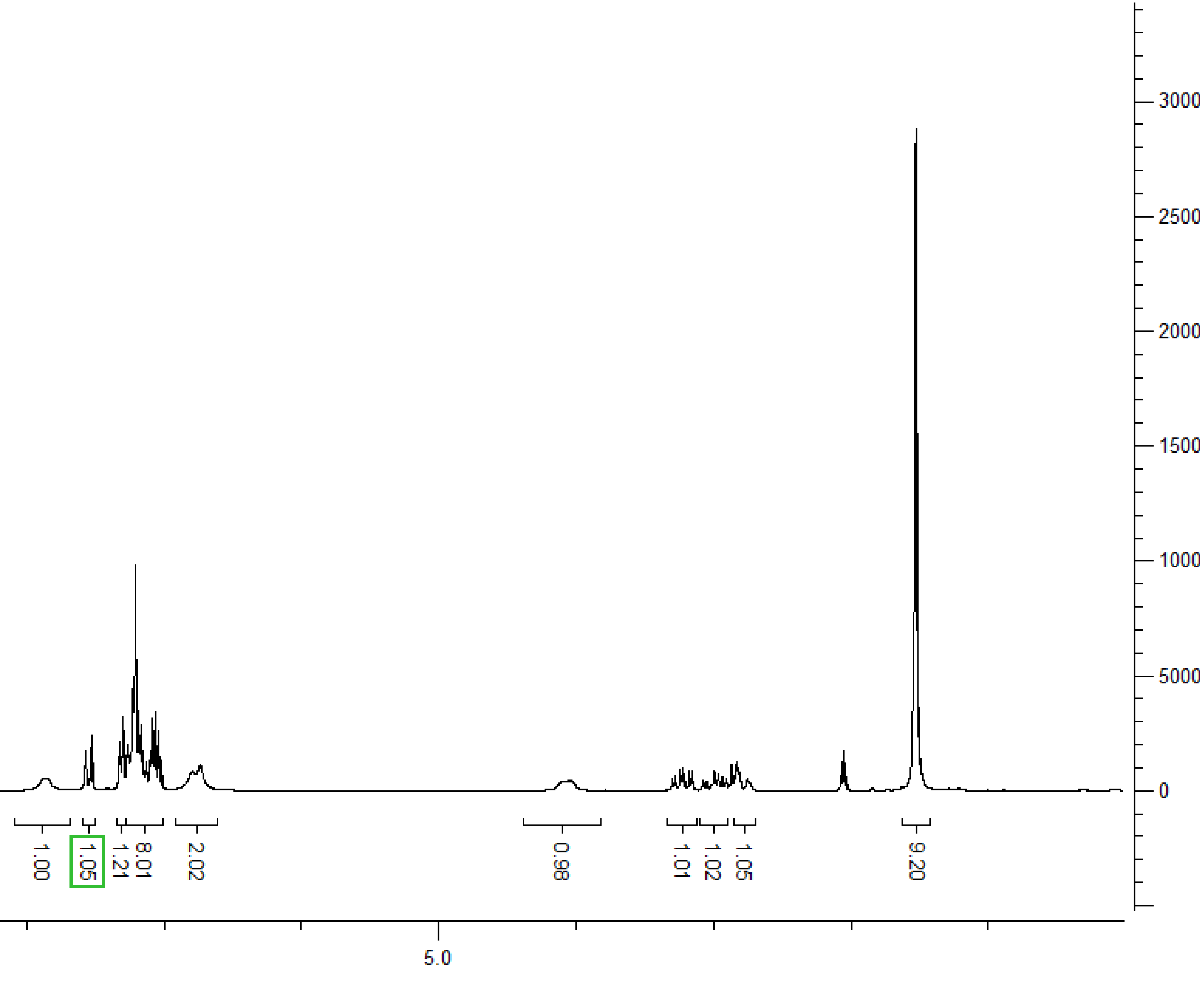
3000
2500
2000
1500
1000
5000
0
1.00
1.05
1.21
8.01
2.02
0.98
1.01
1.02
1.05
9.20
5.0
ppm (t1)

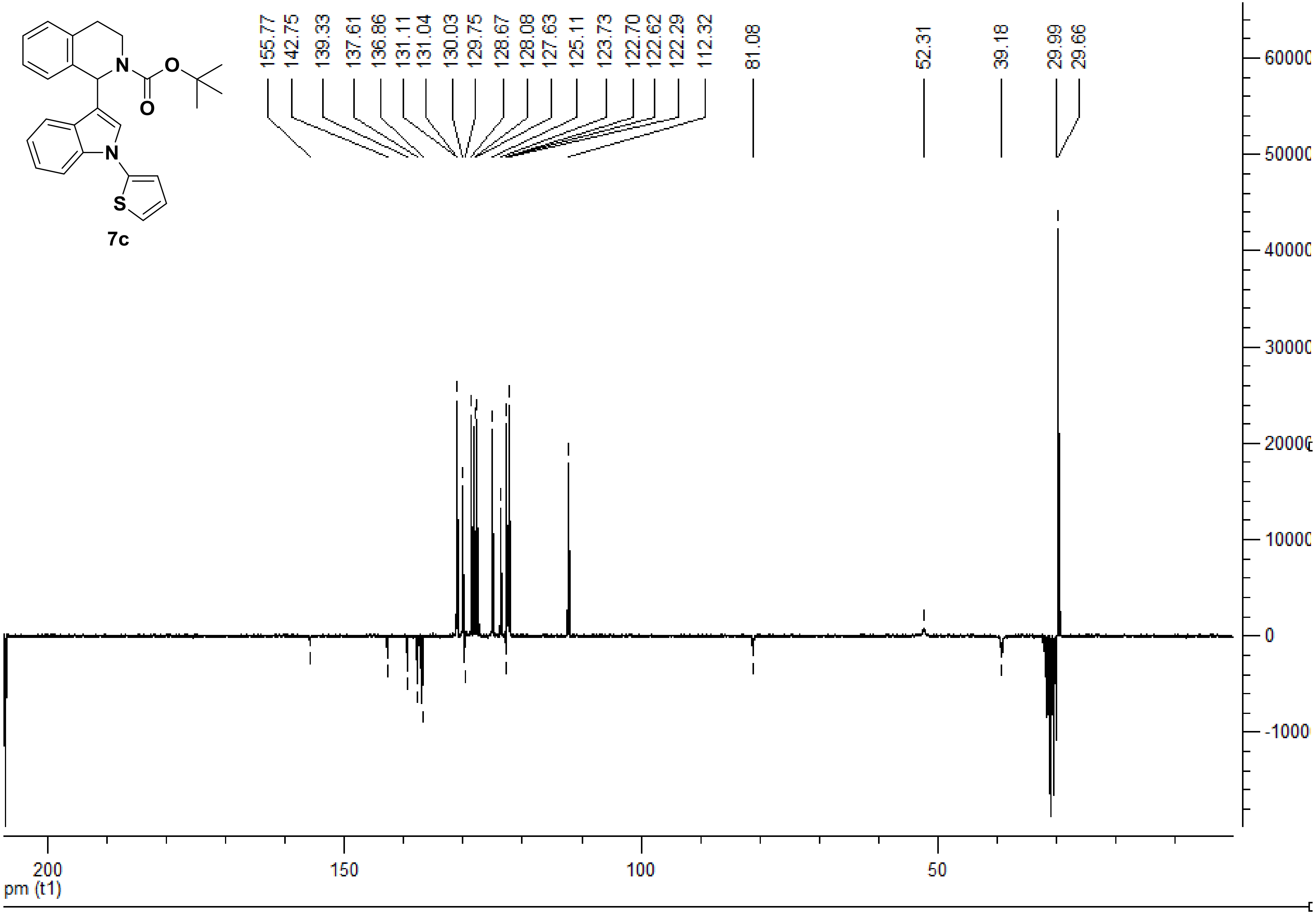
7c
155.77
142.75
139.33
137.61
136.86
131.11
131.04
130.03
129.75
128.67
128.08
127.63
125.11
123.73
122.70
122.62
122.29
112.32
81.08
52.31
39.18
29.99
29.66
60000
50000
40000
30000
20000
10000
0
-1000
200
150
100
50
pm (t1)

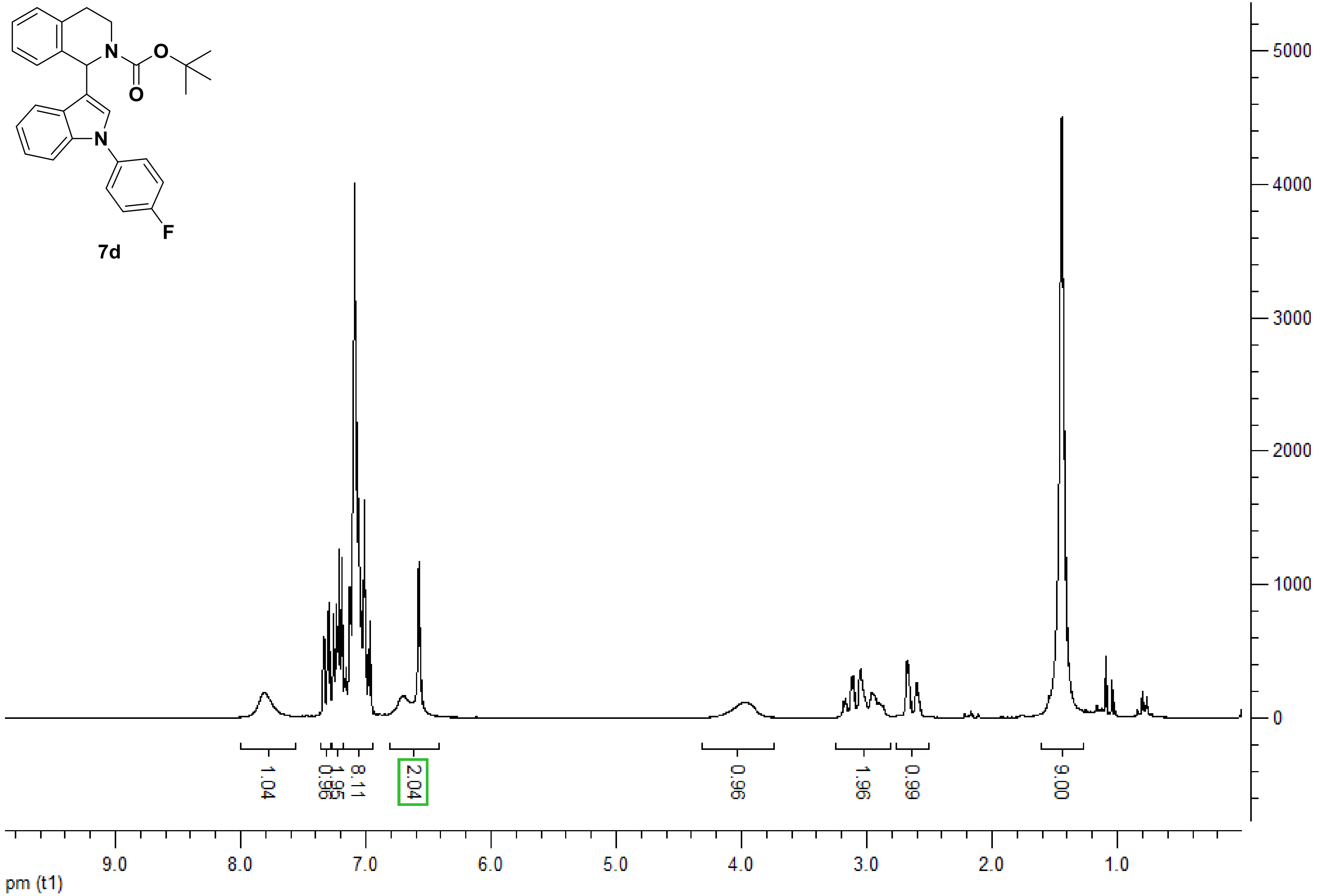
7d
5000
4000
3000
2000
1000
0
1.04
8.11
2.04
0.96
1.96
0.99
9.00
9.0
8.0
7.0
6.0
5.0
4.0
3.0
2.0
1.0
pm (t1)

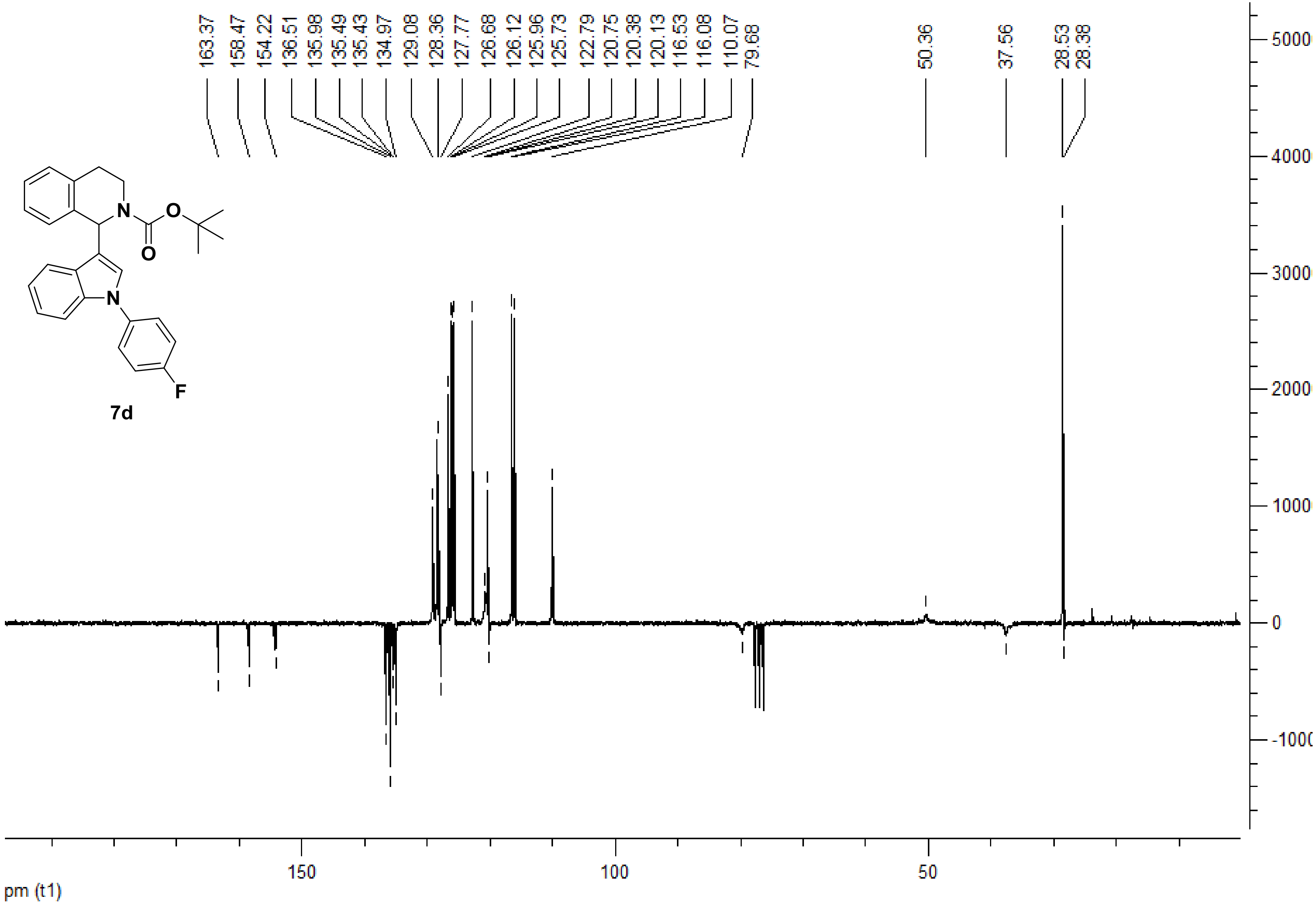
163.37
158.47
154.22
136.51
135.98
135.49
135.43
134.97
129.08
128.36
127.77
126.68
126.12
125.96
125.73
122.79
120.75
120.38
120.13
116.53
116.08
110.07
79.68
50.36
37.56
28.53
28.38
N
O
O
N
F
7d
5000
4000
3000
2000
1000
0
-1000
150
100
50
pm (t1)

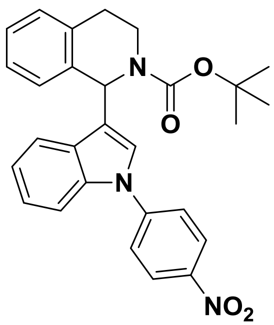

N
O
O
N
NO2

7e

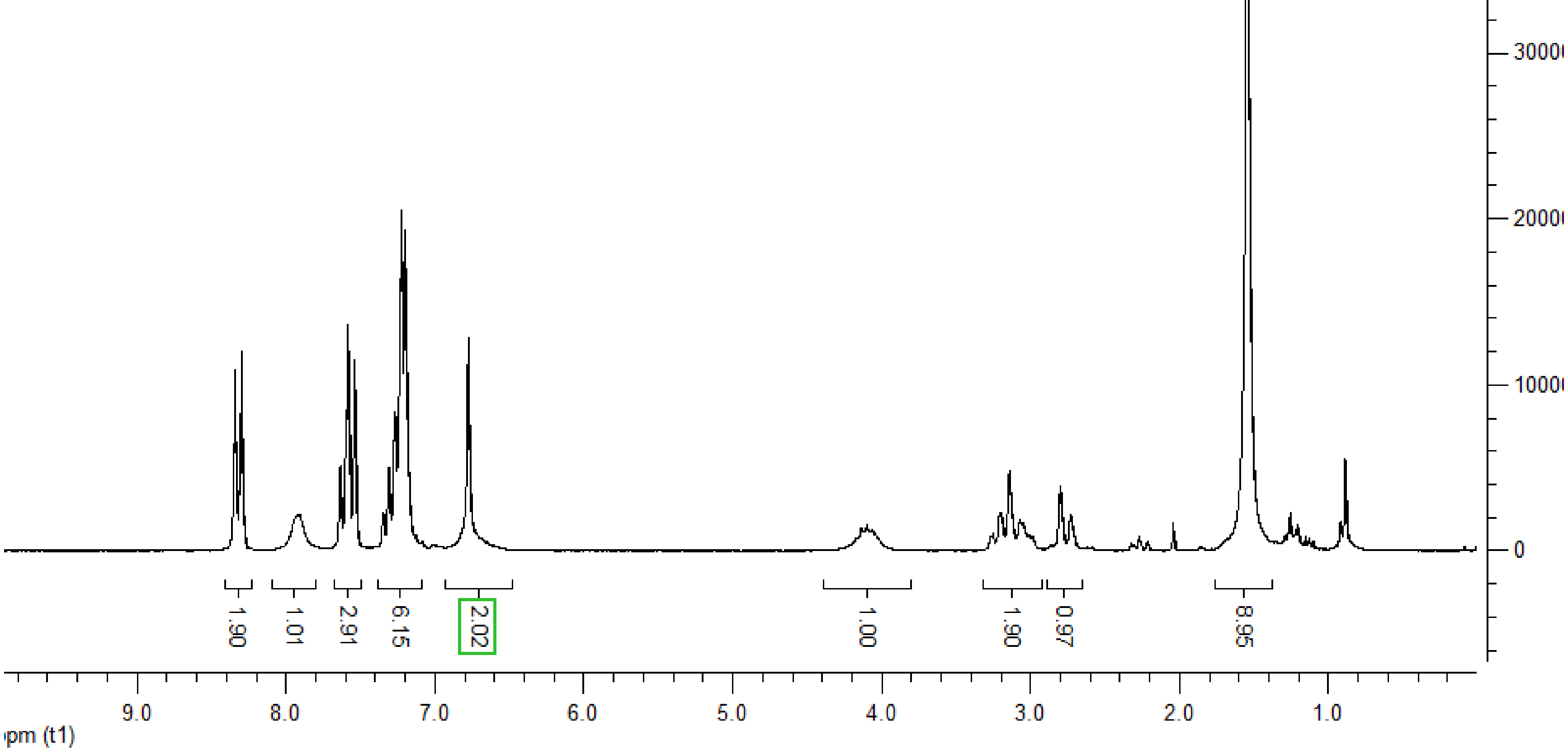

50000
40000
30000
20000
10000
0
1.90
1.01
2.91
6.15
2.02
1.00
1.90
0.97
8.95
9.0
8.0
7.0
6.0
5.0
4.0
3.0
2.0
1.0
ppm (t1)

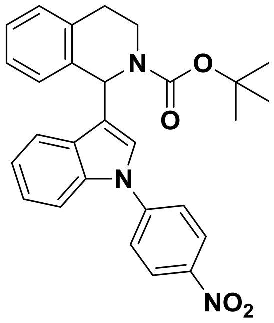
N
O
O
N
NO2

7e

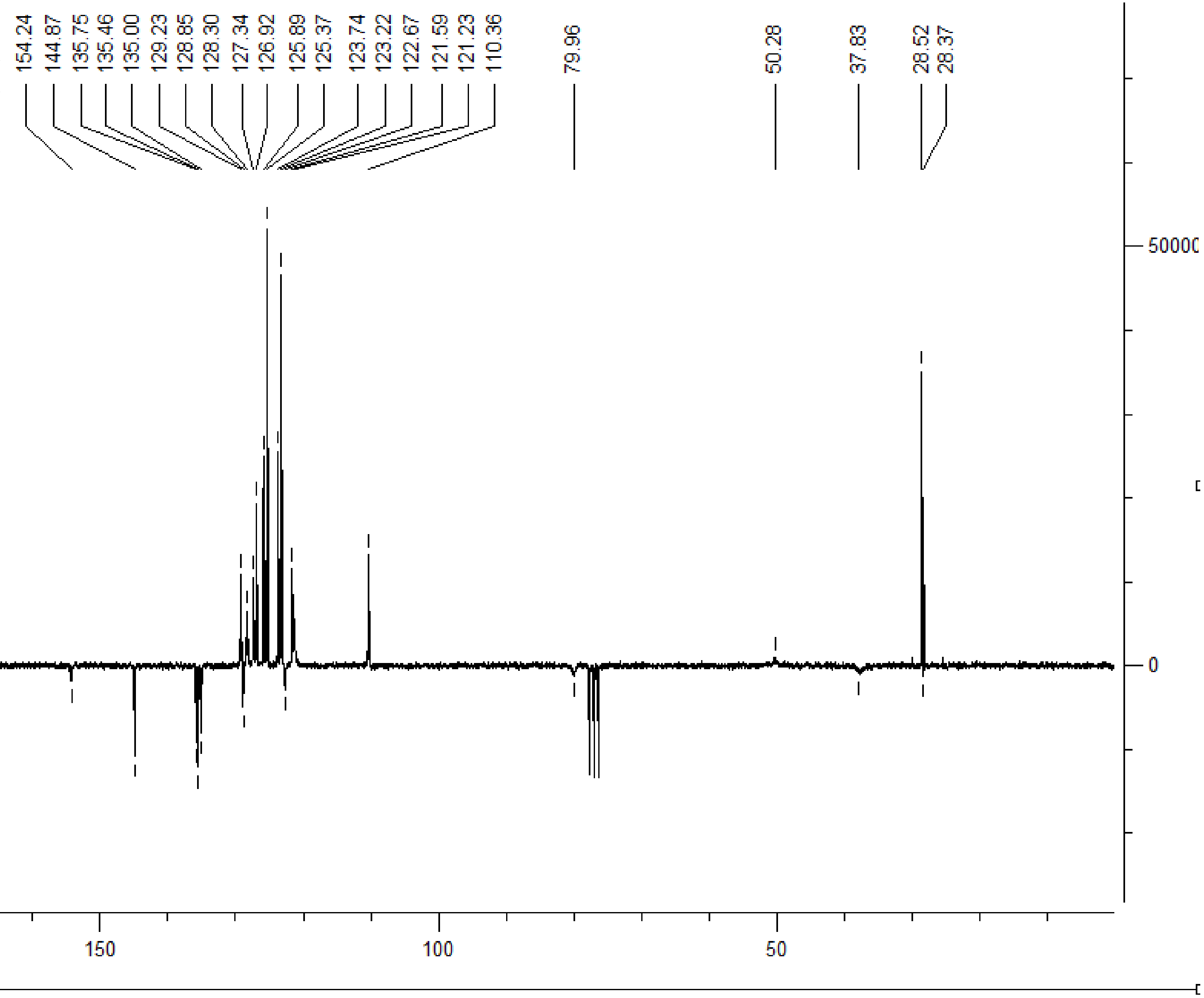
154.24
144.87
135.75
135.46
135.00
129.23
128.85
128.30
127.34
126.92
125.89
125.37
123.74
123.22
122.67
121.59
121.23
110.36
79.96
50.28
37.83
28.52
28.37
50000
0
150
100
50
ppm (t1)

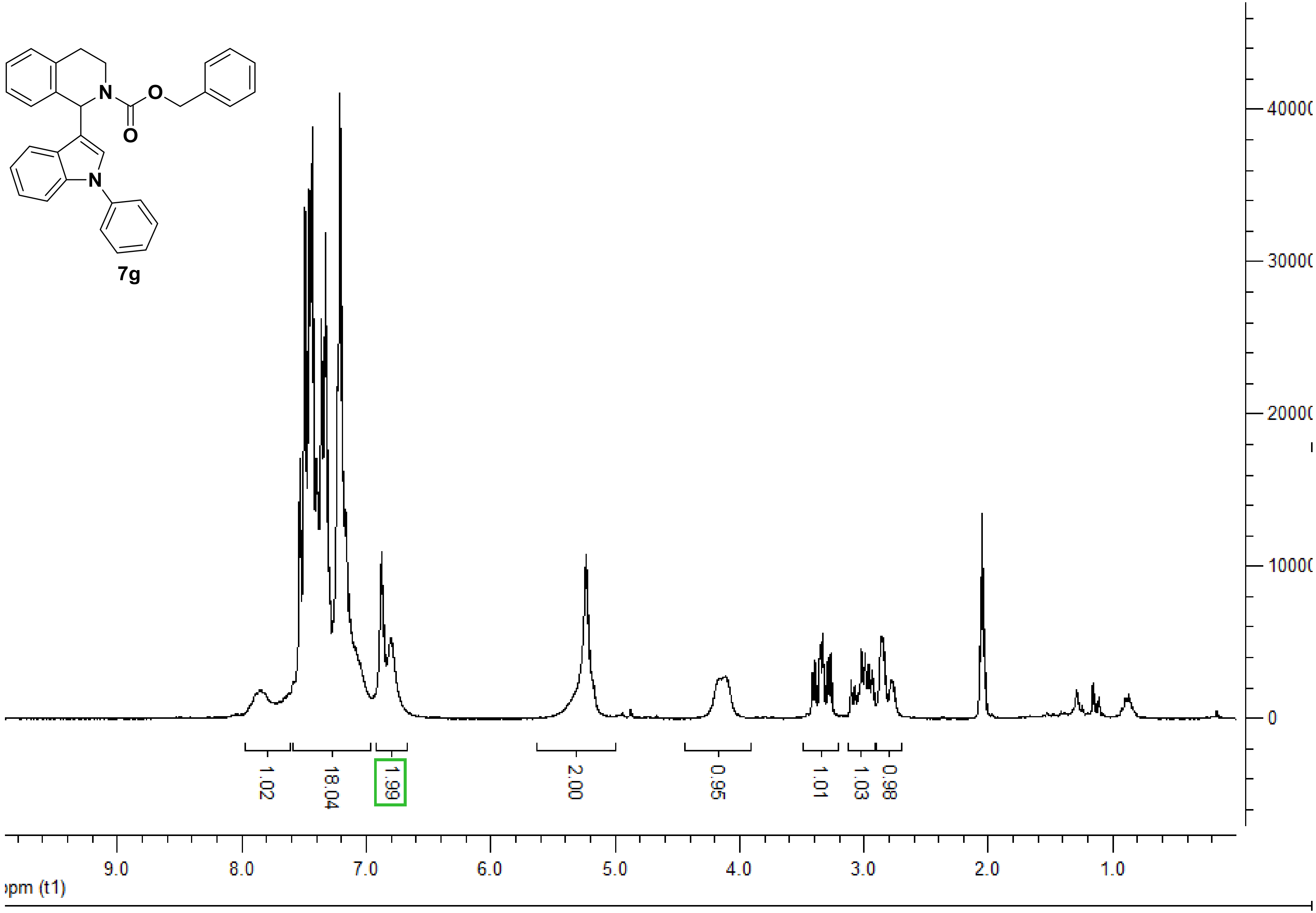

7g
40000
30000
20000
10000
0
1.02
18.04
1.99
2.00
0.95
1.01
1.03
0.98
9.0
8.0
7.0
6.0
5.0
4.0
3.0
2.0
1.0
ppm (t1)

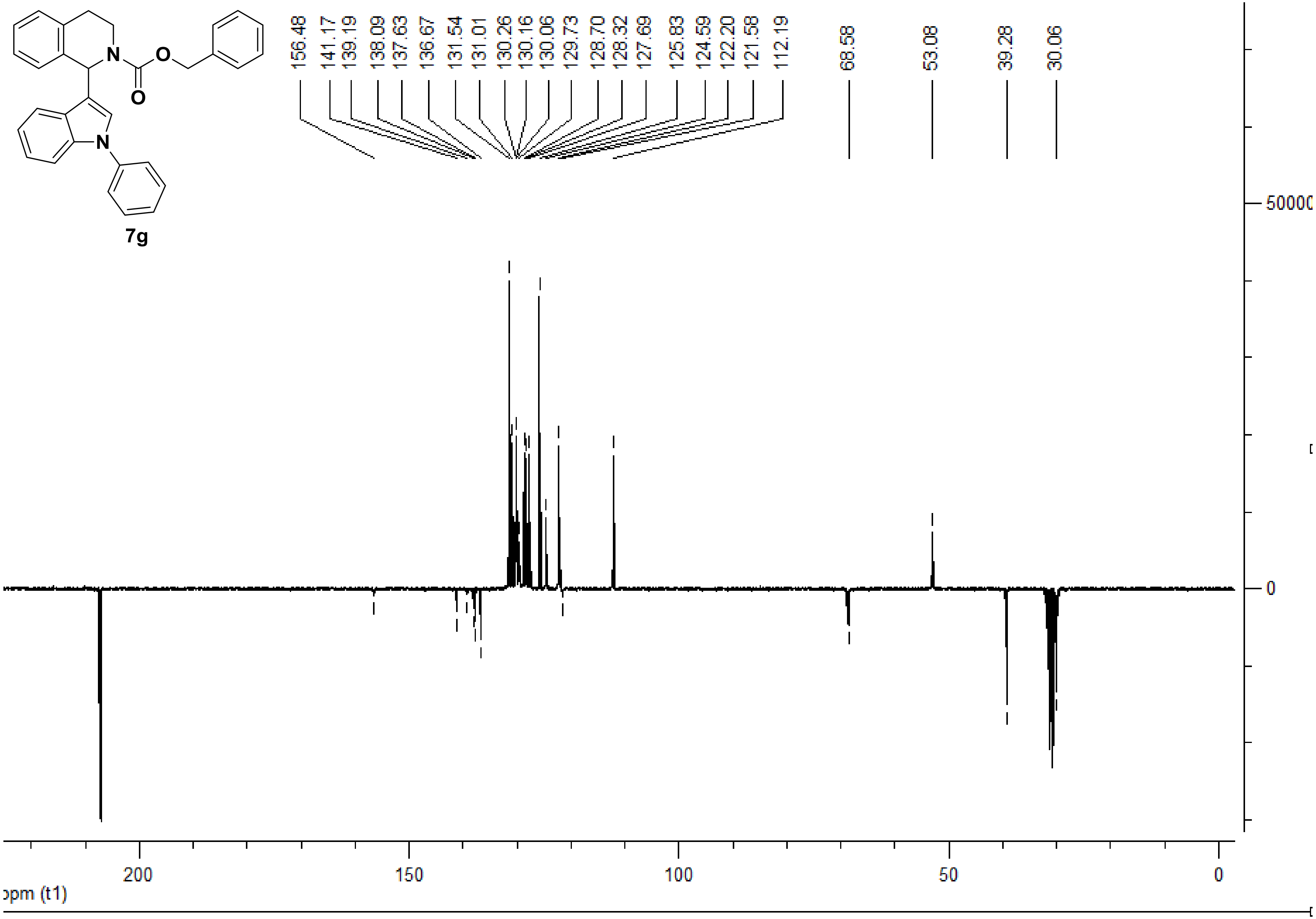
156.48
141.17
139.19
138.09
137.63
136.67
131.54
131.01
130.26
130.16
130.06
129.73
128.70
128.32
127.69
125.83
124.59
122.20
121.58
112.19
68.58
53.08
39.28
30.06
50000
0
200
150
100
50
0
ppm (t1)

7g

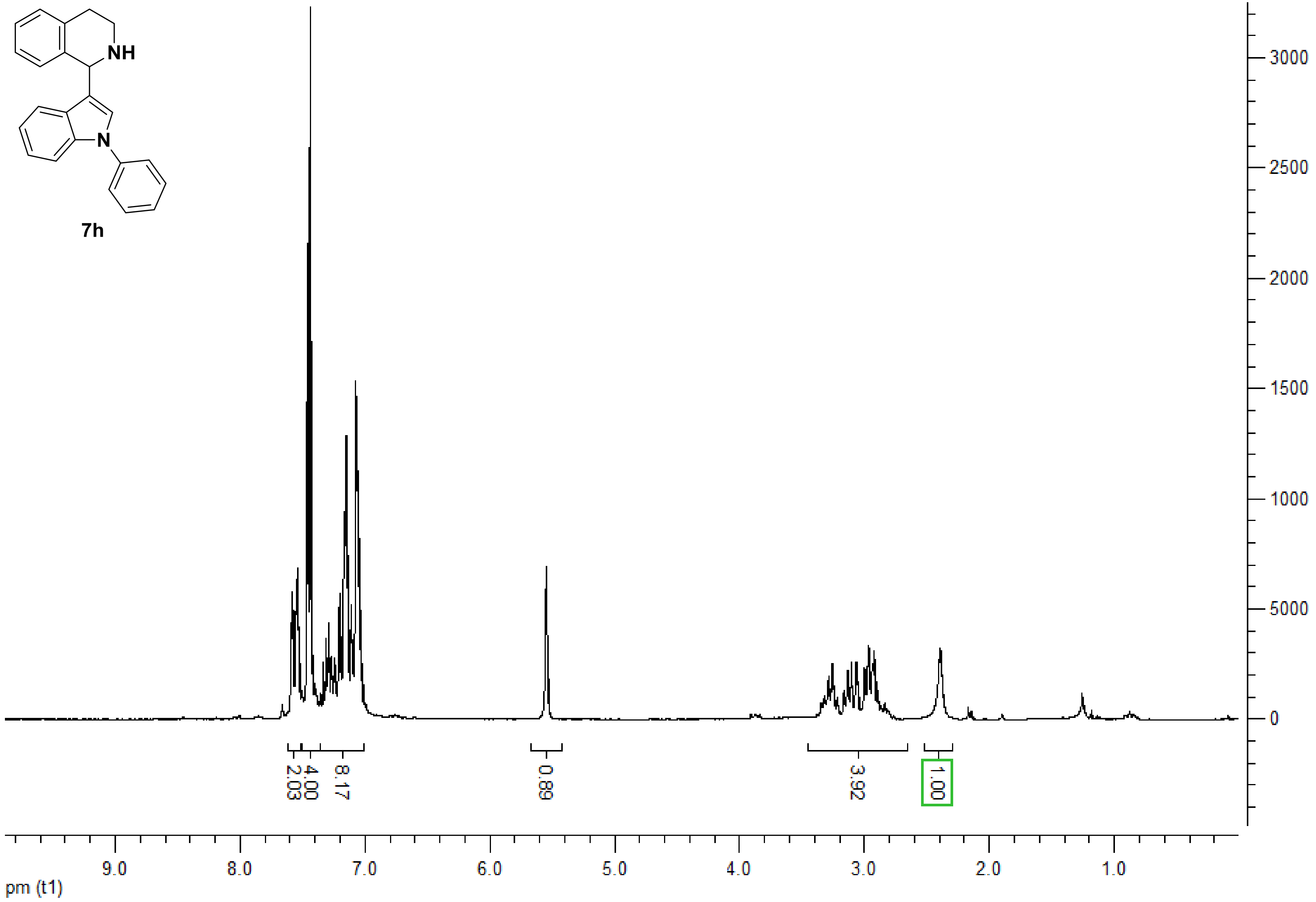

NH
N
7h
3000
2500
2000
1500
1000
5000
0
2.03
4.00
8.17
0.89
3.92
1.00
9.0
8.0
7.0
6.0
5.0
4.0
3.0
2.0
1.0
pm (t1)

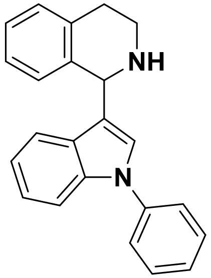

7h

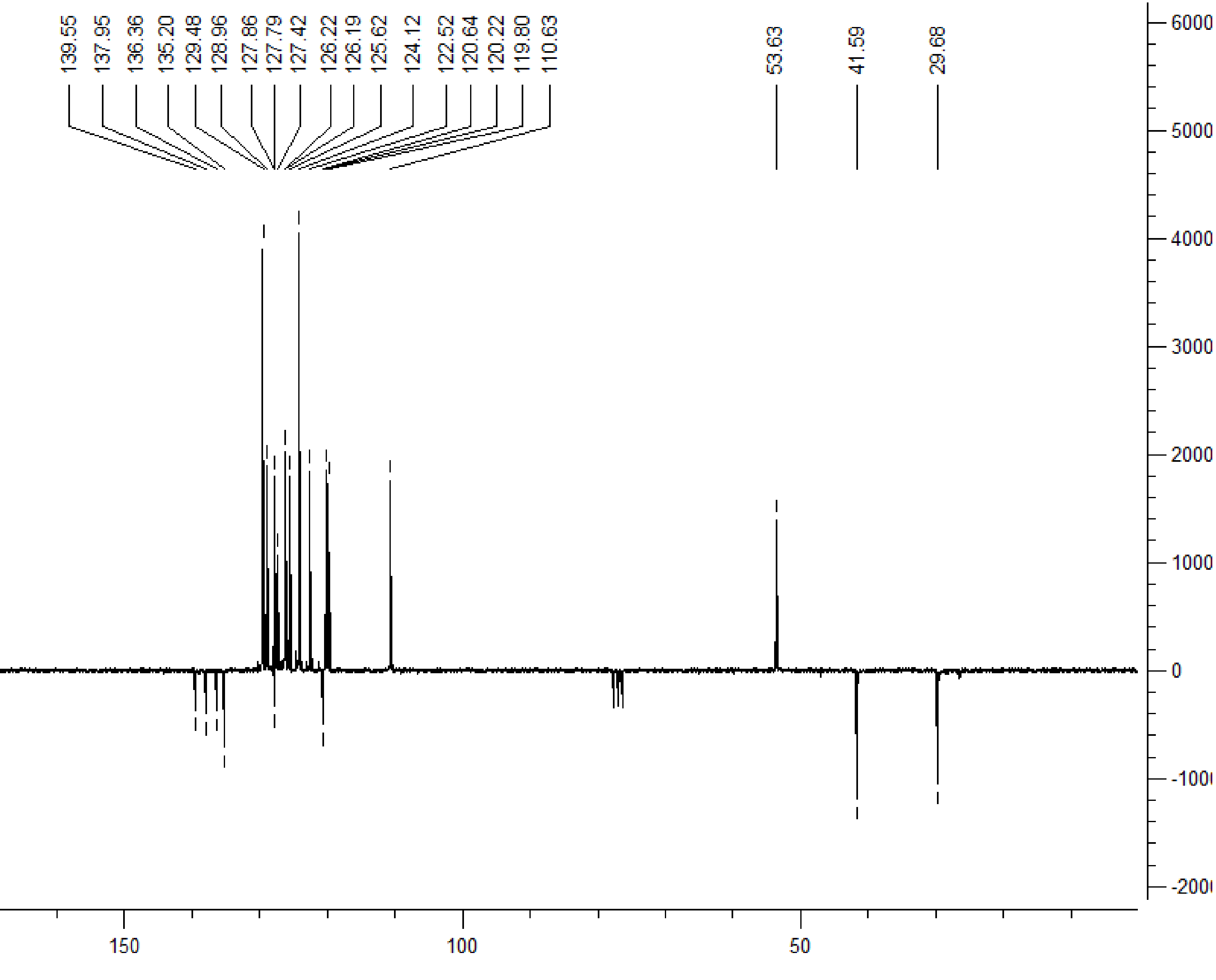

139.55
137.95
136.36
135.20
129.48
128.96
127.86
127.79
127.42
126.22
126.19
125.62
124.12
122.52
120.64
120.22
119.80
110.63
53.63
41.59
29.68
6000
5000
4000
3000
2000
1000
0
-100
-200
150
100
50
pm (t1)

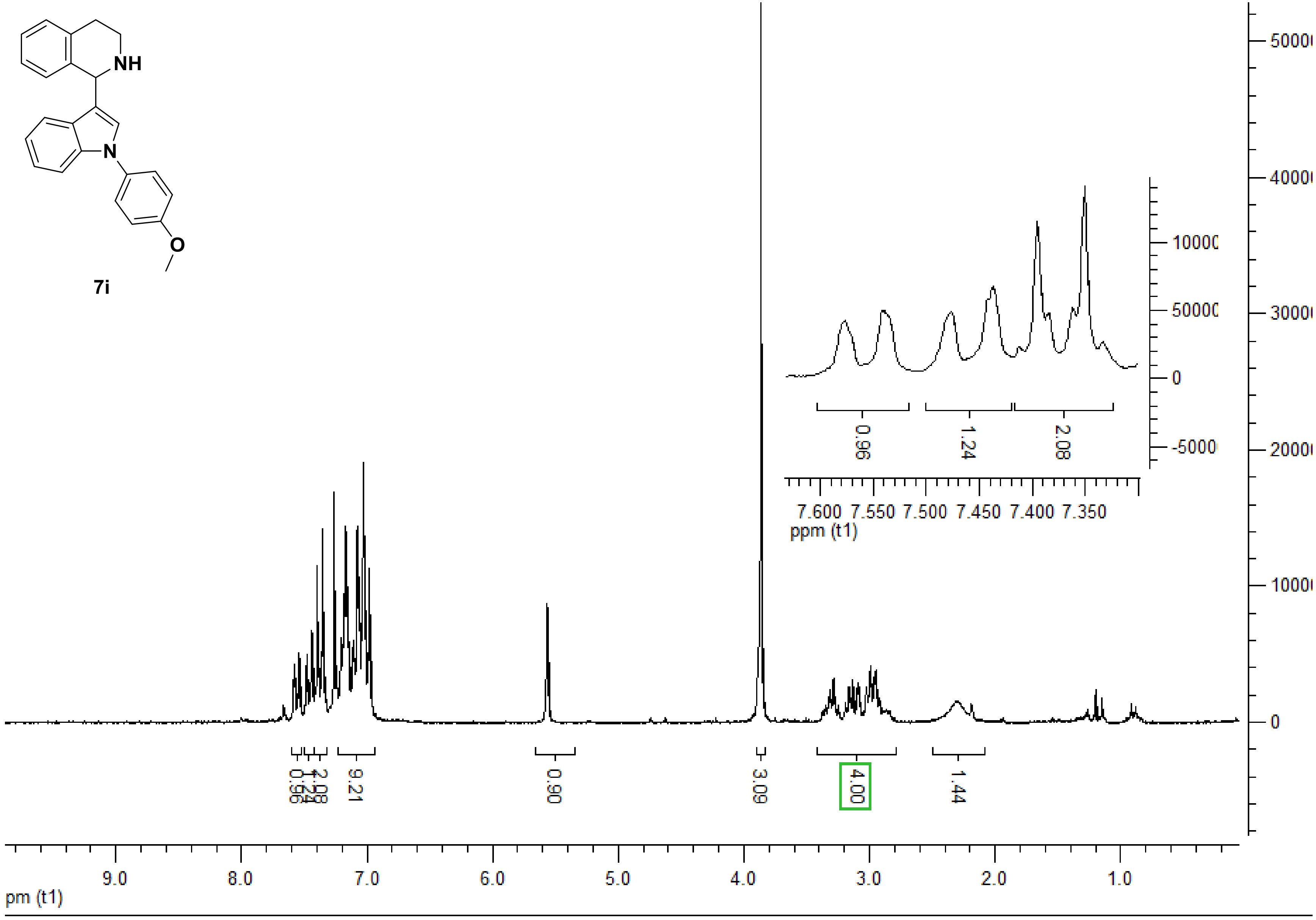
NH
N
O
7i
7.600 7.550 7.500 7.450 7.400 7.350
ppm (t1)
0.96
1.24
2.08
9.0 8.0 7.0 6.0 5.0 4.0 3.0 2.0 1.0
pm (t1)
0.96
1.24
2.08
9.21
0.90
3.09
4.00
1.44

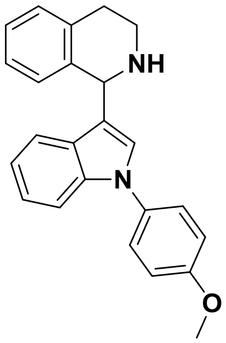

7i

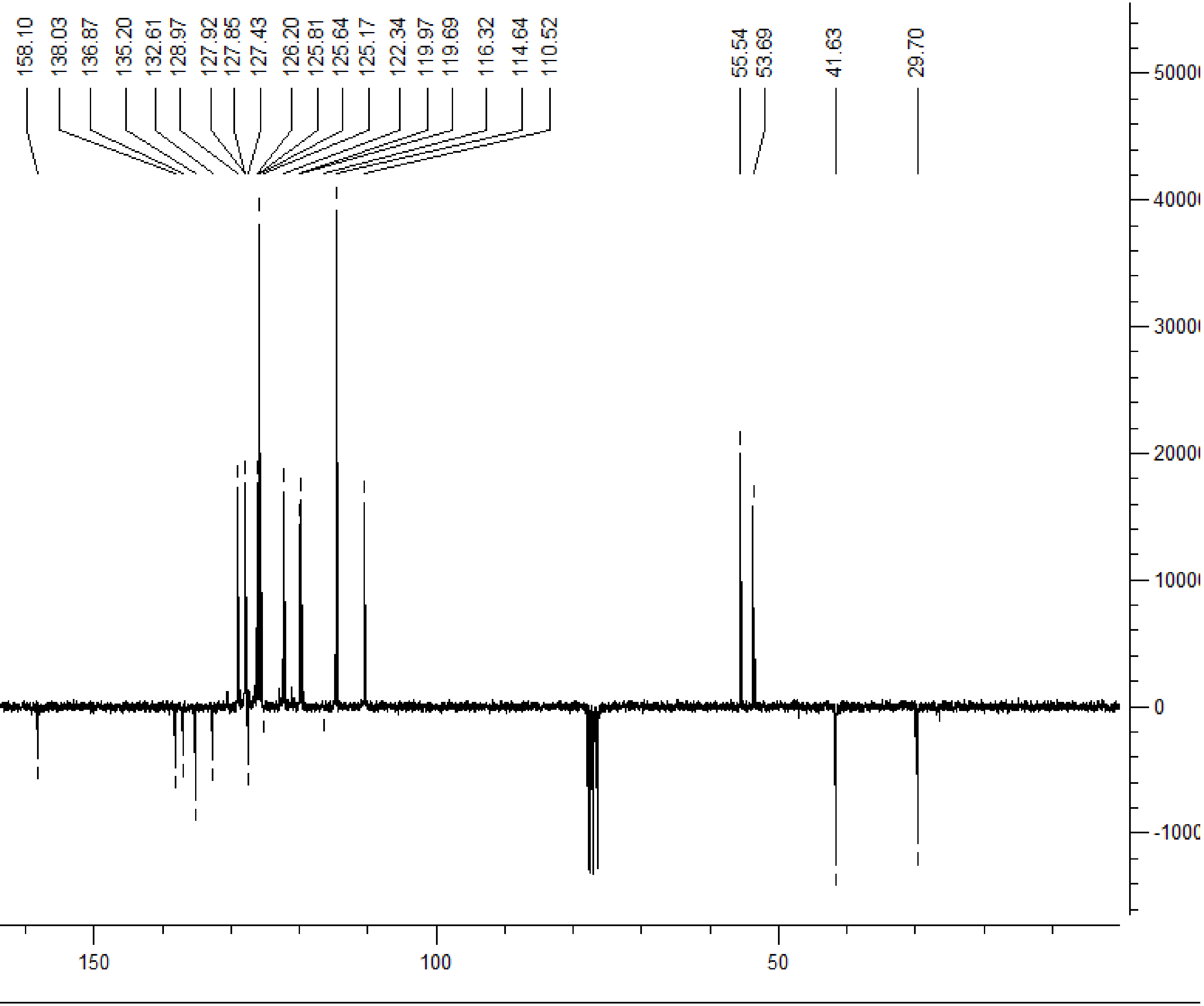
158.10
138.03
136.87
135.20
132.61
128.97
127.92
127.85
127.43
126.20
125.81
125.64
125.17
122.34
119.97
119.69
116.32
114.64
110.52
55.54
53.69
41.63
29.70
150
100
50
ppm (t1)

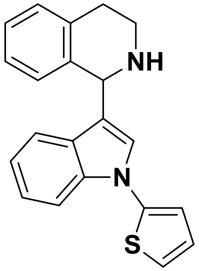

7j

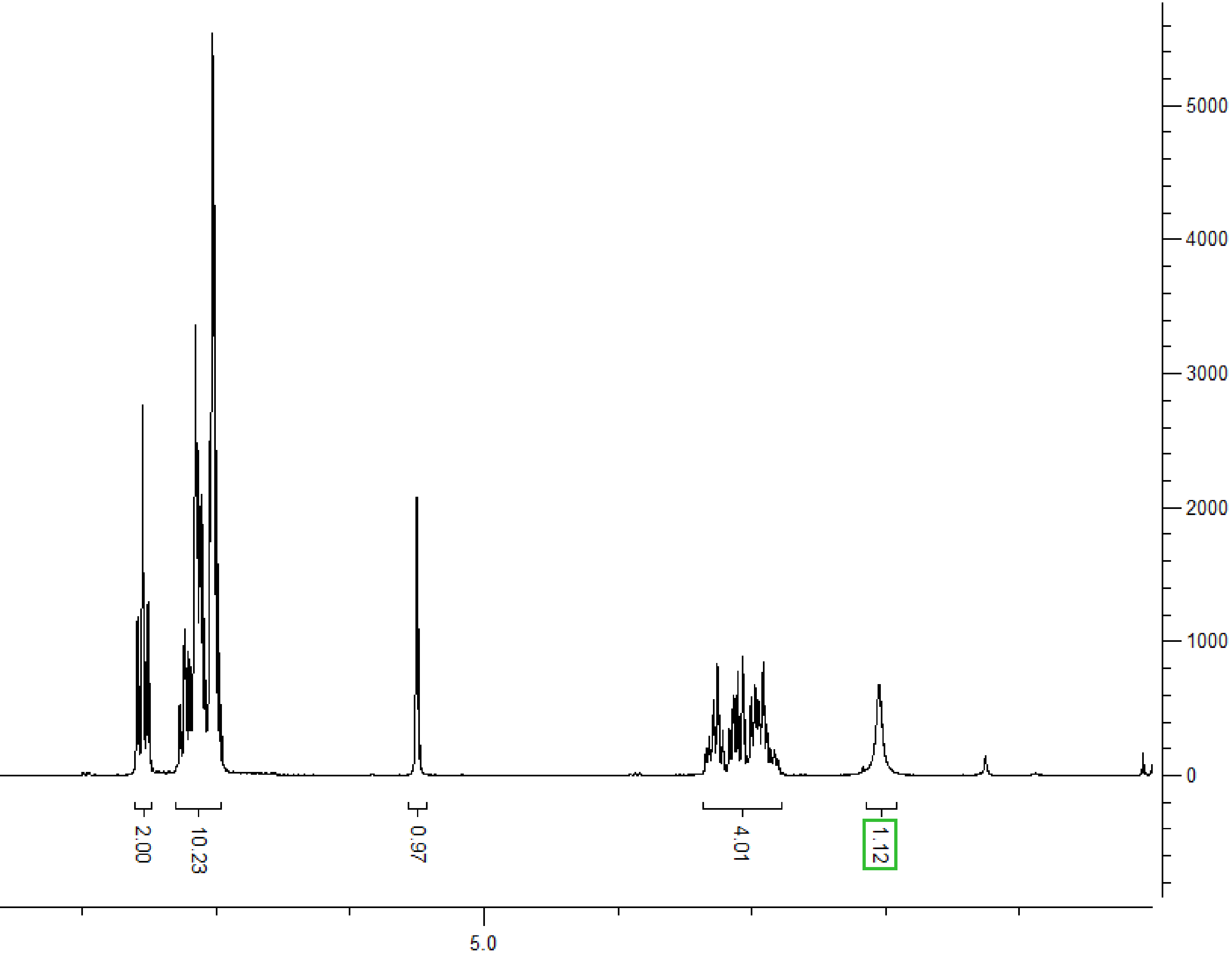

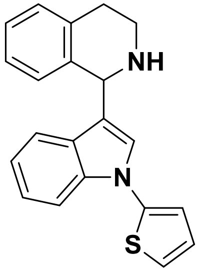
7j
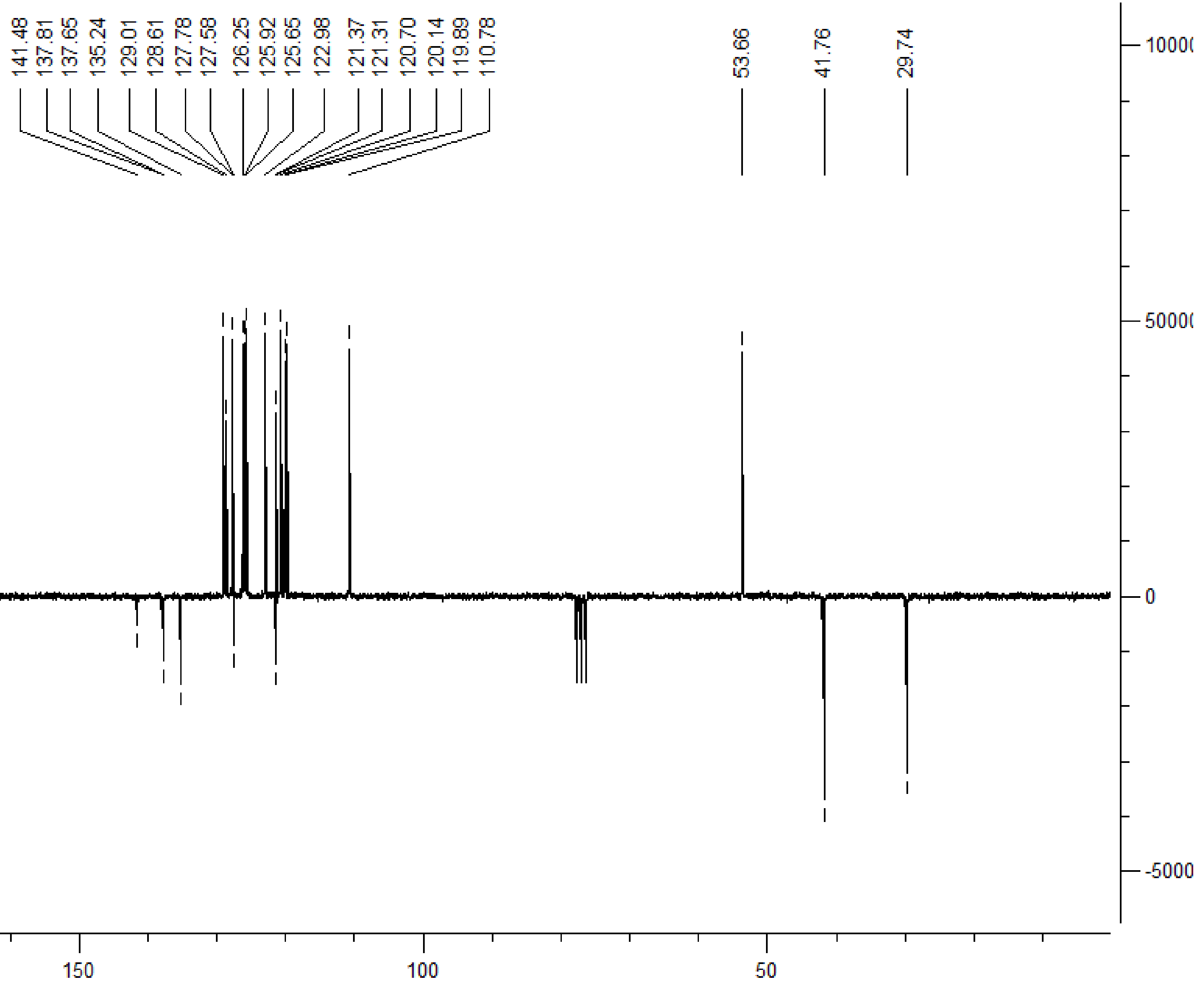
141.48
137.81
137.65
135.24
129.01
128.61
127.78
127.58
126.25
125.92
125.65
122.98
121.37
121.31
120.70
120.14
119.89
110.78
53.66
41.76
29.74
10000
50000
0
-5000
150
100
50
ppm (t1)

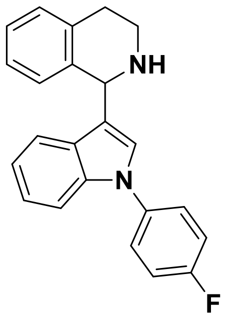
NH
N
F

7k

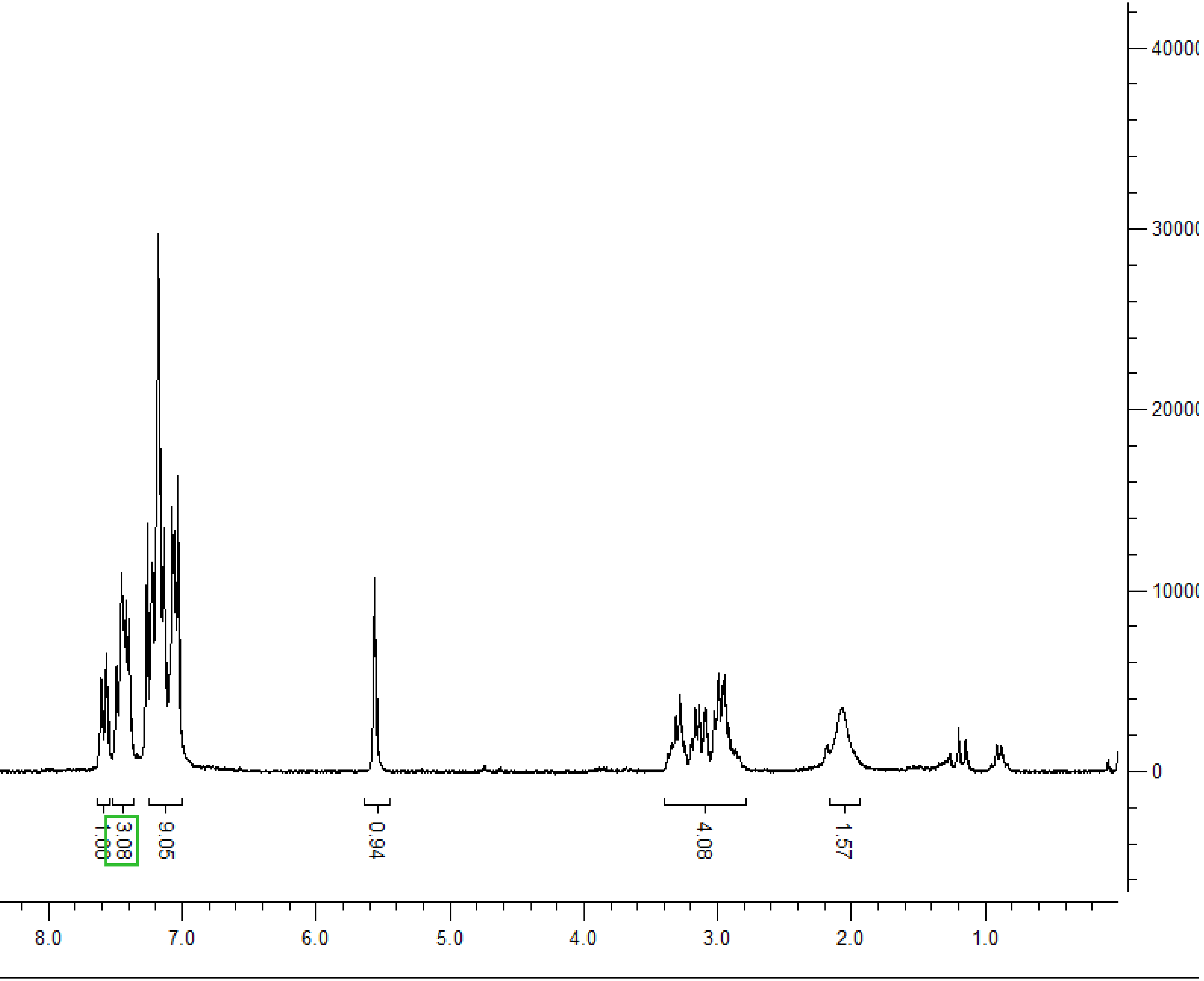
40000
30000
20000
10000
0
1.00
3.08
9.05
0.94
4.08
1.57
9.0
8.0
7.0
6.0
5.0
4.0
3.0
2.0
1.0
ppm (t1)

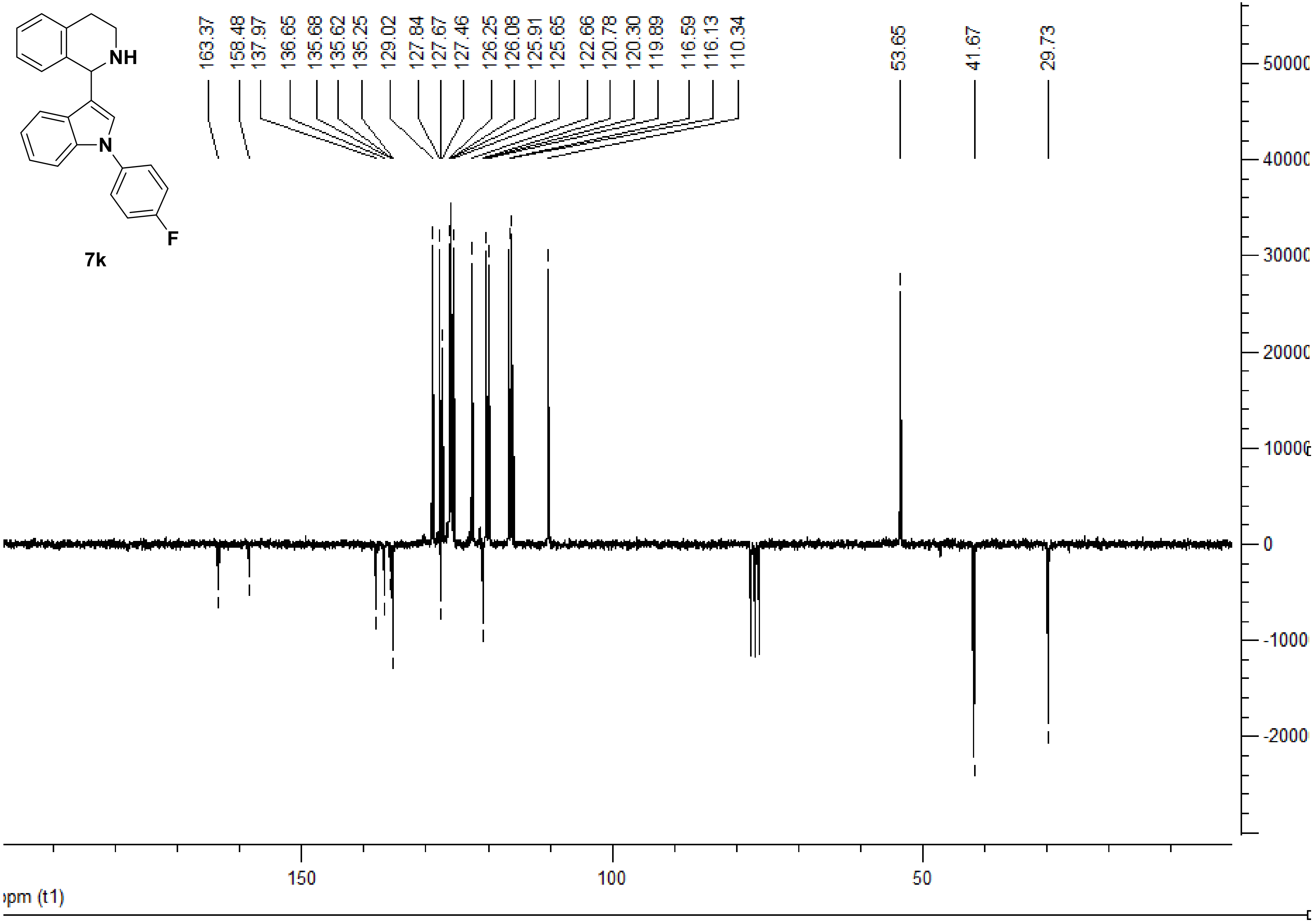
NH
N
F
7k
163.37
158.48
137.97
136.65
135.68
135.62
135.25
129.02
127.84
127.67
127.46
126.25
126.08
125.91
125.65
122.66
120.78
120.30
119.89
116.59
116.13
110.34
53.65
41.67
29.73
50000
40000
30000
20000
10000
0
-1000
-2000
150
100
50
ppm (t1)

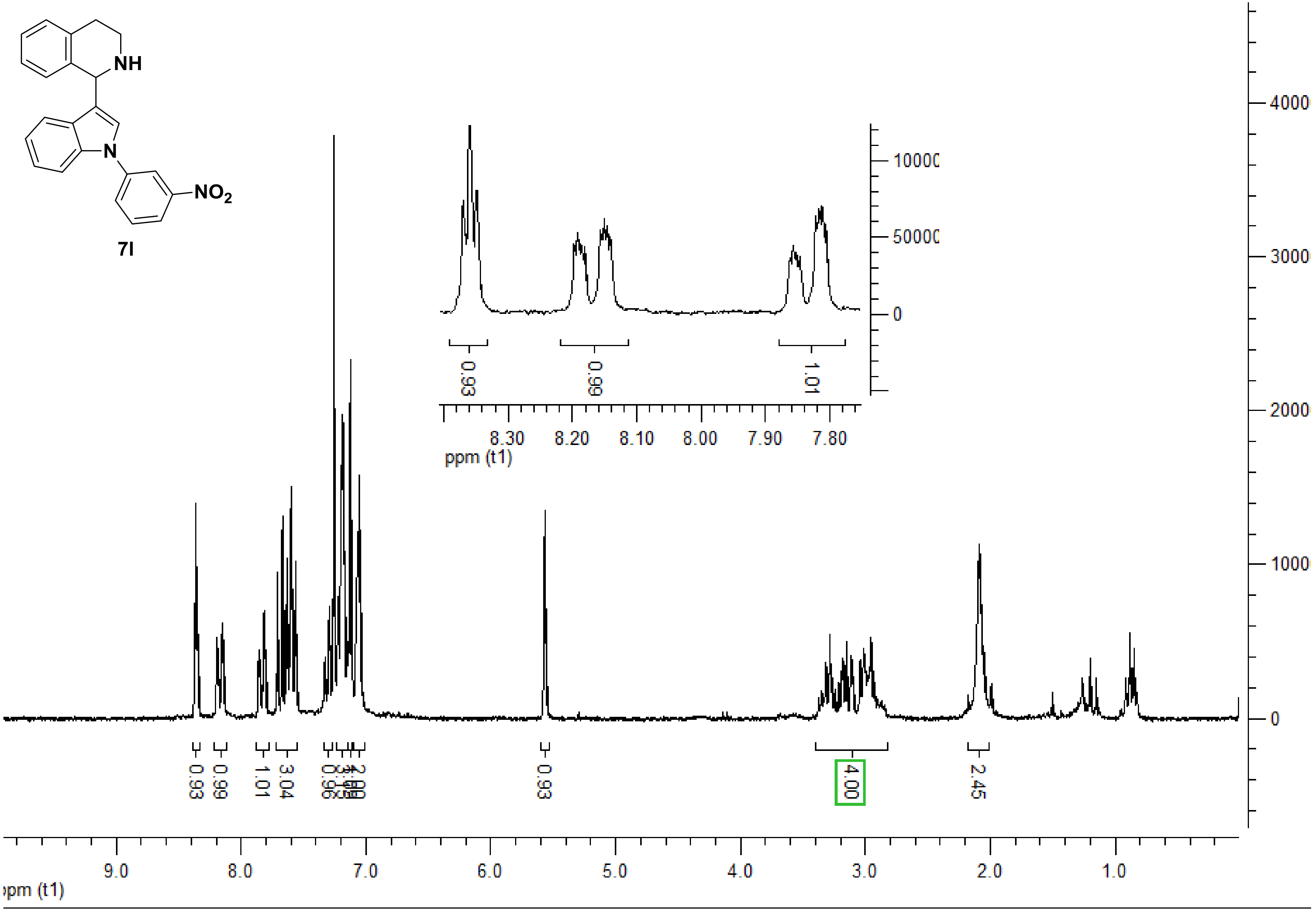
NH
N
NO2
7l
0.93
0.99
1.01
8.30
8.20
8.10
8.00
7.90
7.80
ppm (t1)
0.93
0.99
1.01
3.04
0.96
0.93
4.00
2.45
9.0
8.0
7.0
6.0
5.0
4.0
3.0
2.0
1.0
ppm (t1)
4000
3000
2000
1000
0

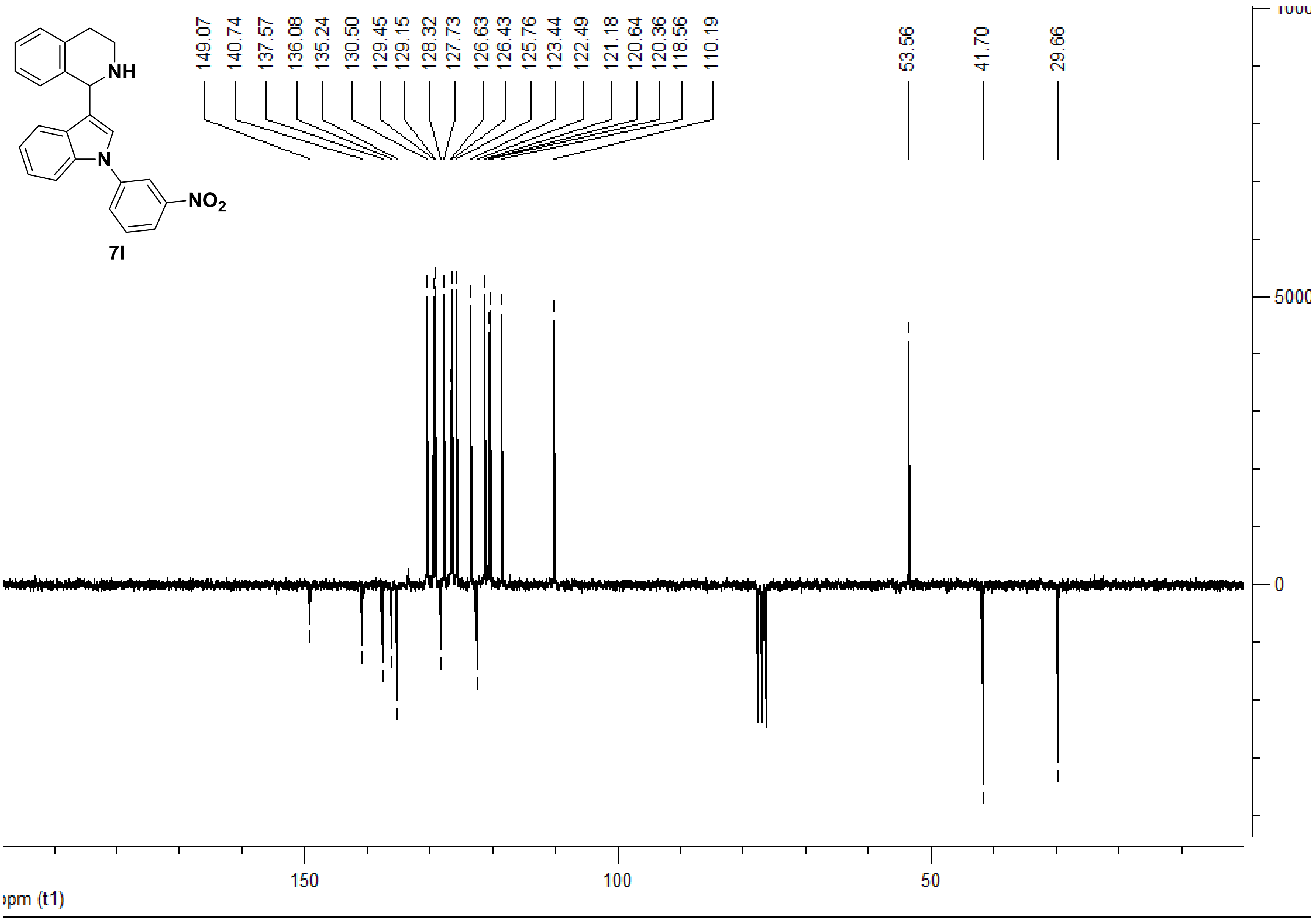

NH
N
NO2
7l
149.07
140.74
137.57
136.08
135.24
130.50
129.45
129.15
128.32
127.73
126.63
126.43
125.76
123.44
122.49
121.18
120.64
120.36
118.56
110.19
53.56
41.70
29.66
5000
0
150
100
50
ppm (t1)

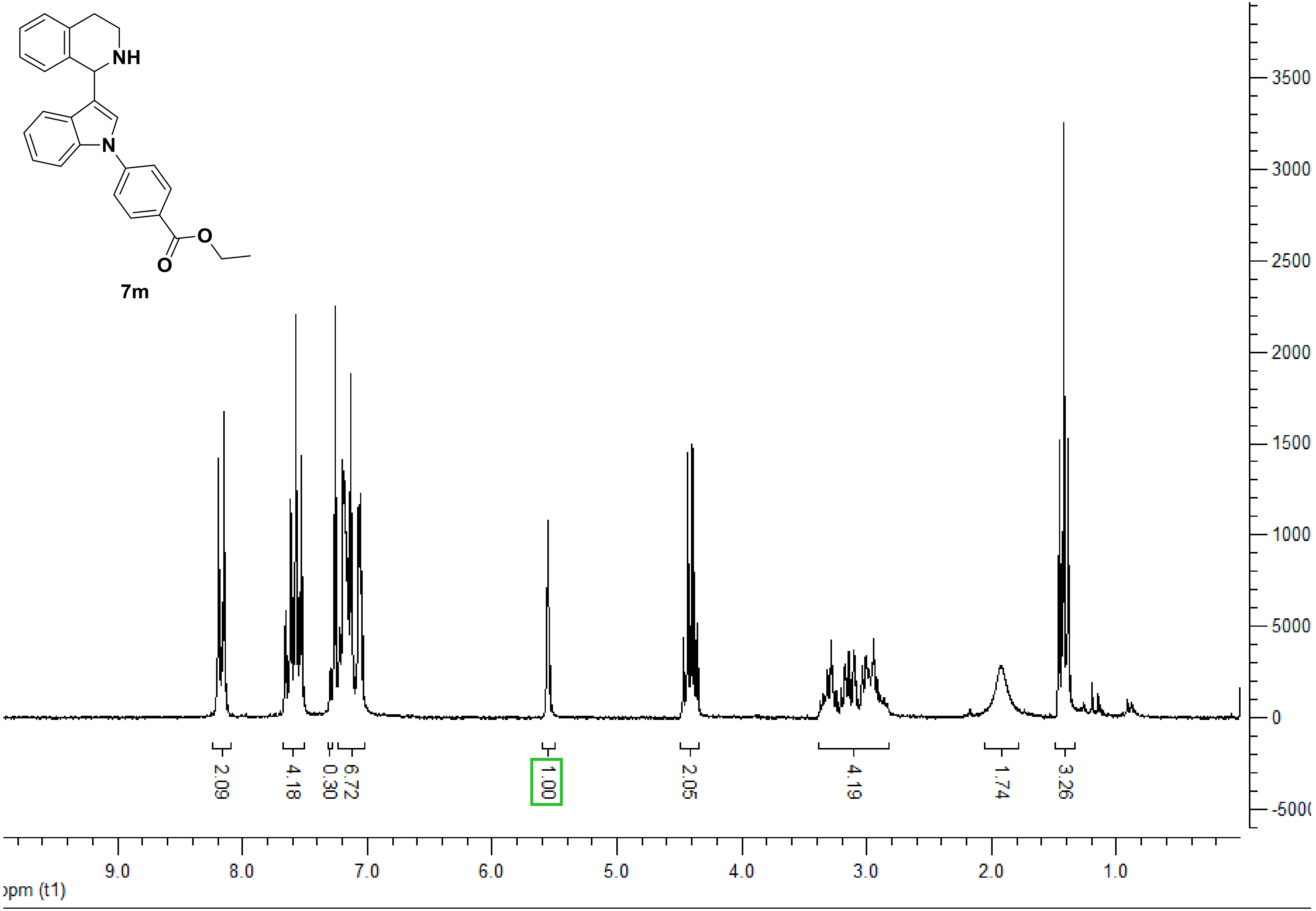
NH
N
O
O
7m
3500
3000
2500
2000
1500
1000
5000
0
-500(
2.09
4.18
0.30
6.72
1.00
2.05
4.19
1.74
3.26
9.0
8.0
7.0
6.0
5.0
4.0
3.0
2.0
1.0
ɔpm (t1)

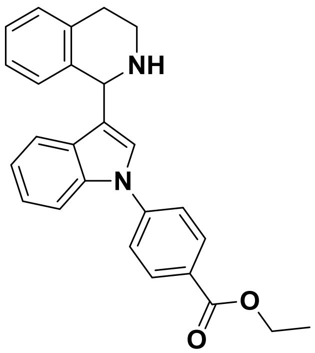
NH
N
O
O

7m

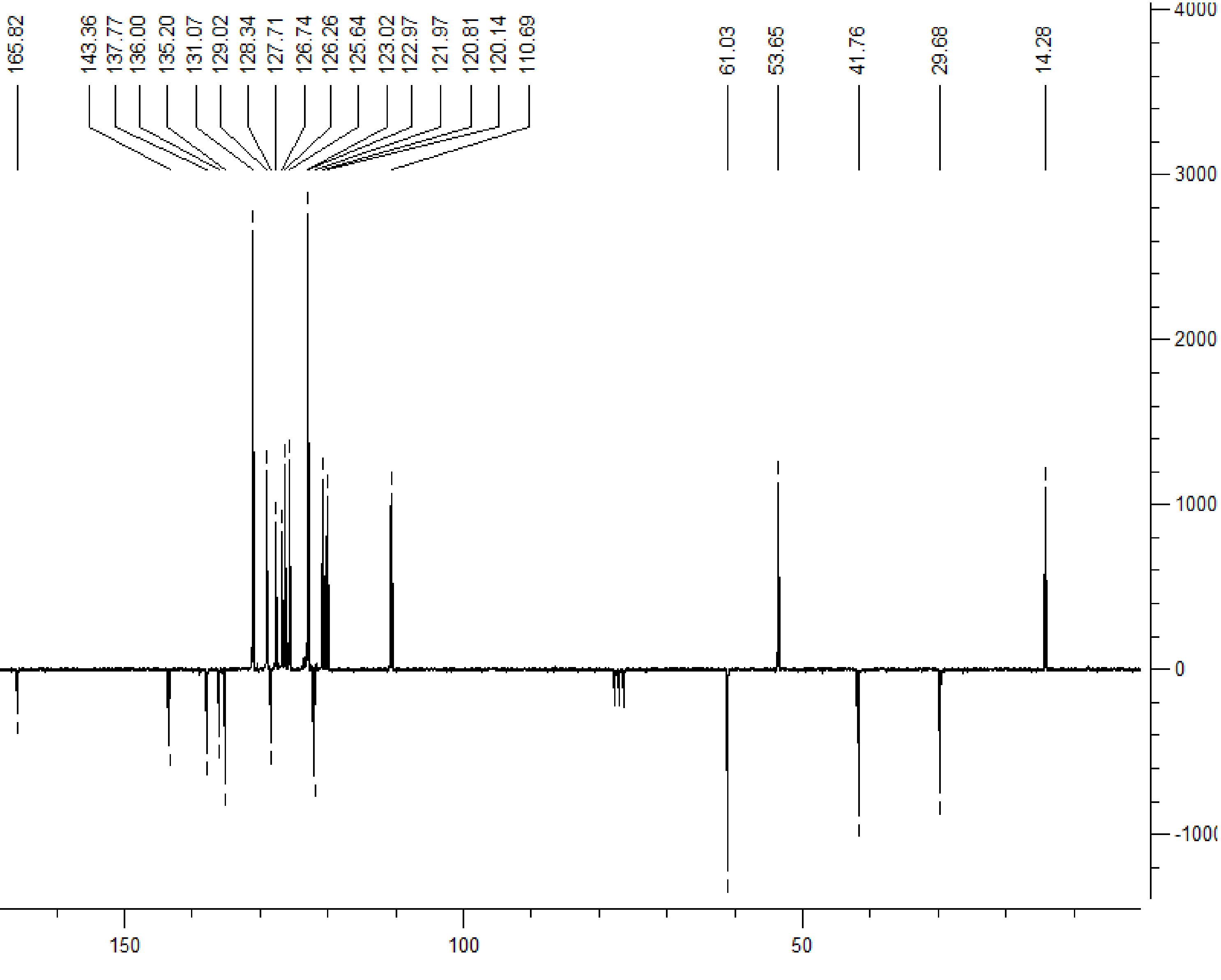
165.82
143.36
137.77
136.00
135.20
131.07
129.02
128.34
127.71
126.74
126.26
125.64
123.02
122.97
121.97
120.81
120.14
110.69
61.03
53.65
41.76
29.68
14.28
4000
3000
2000
1000
0
-100(
150
100
50
ppm (t1)

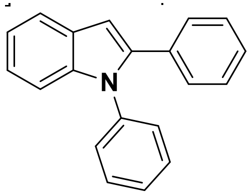

8a

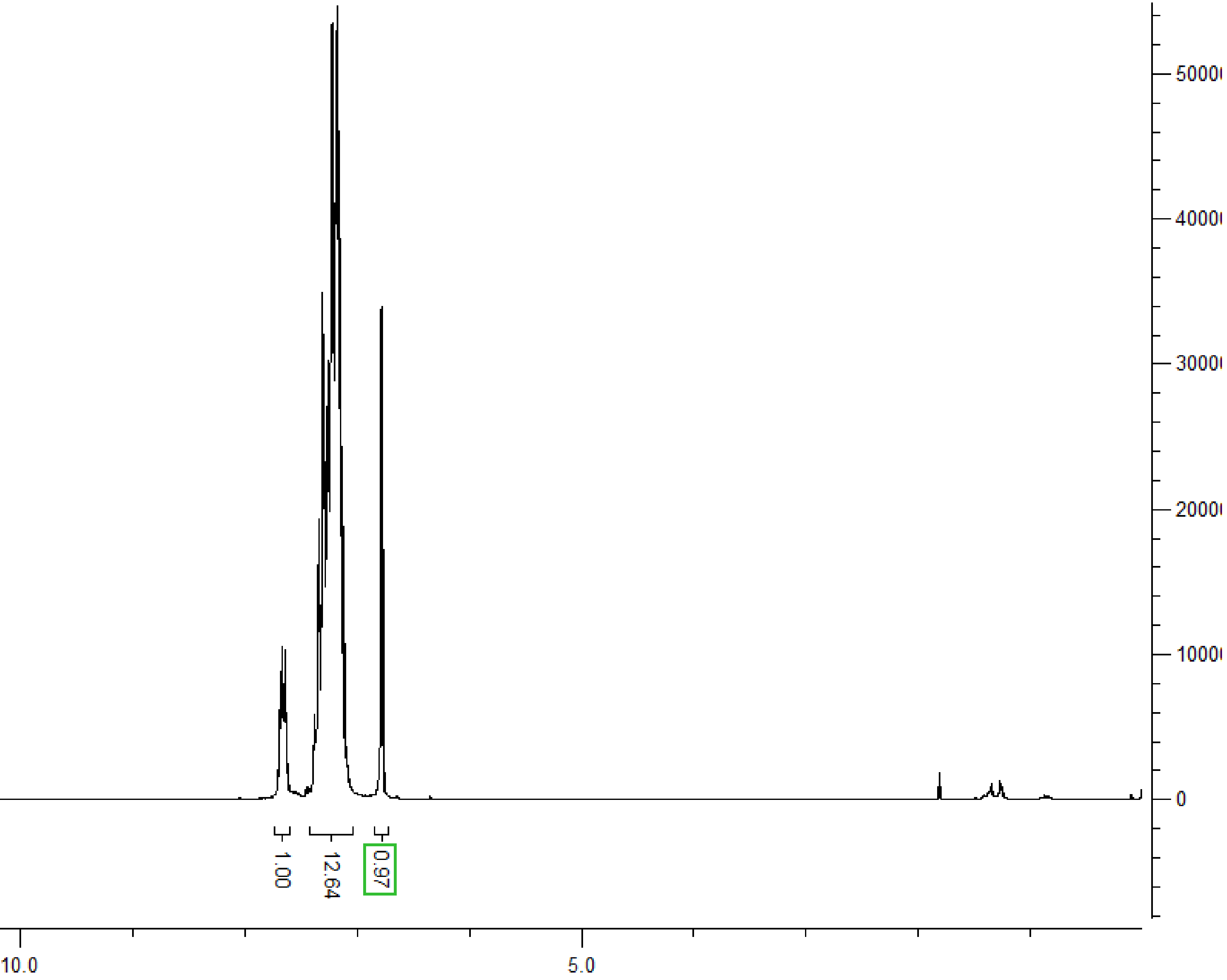

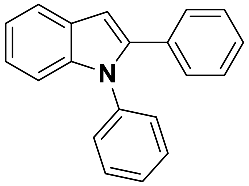

8a

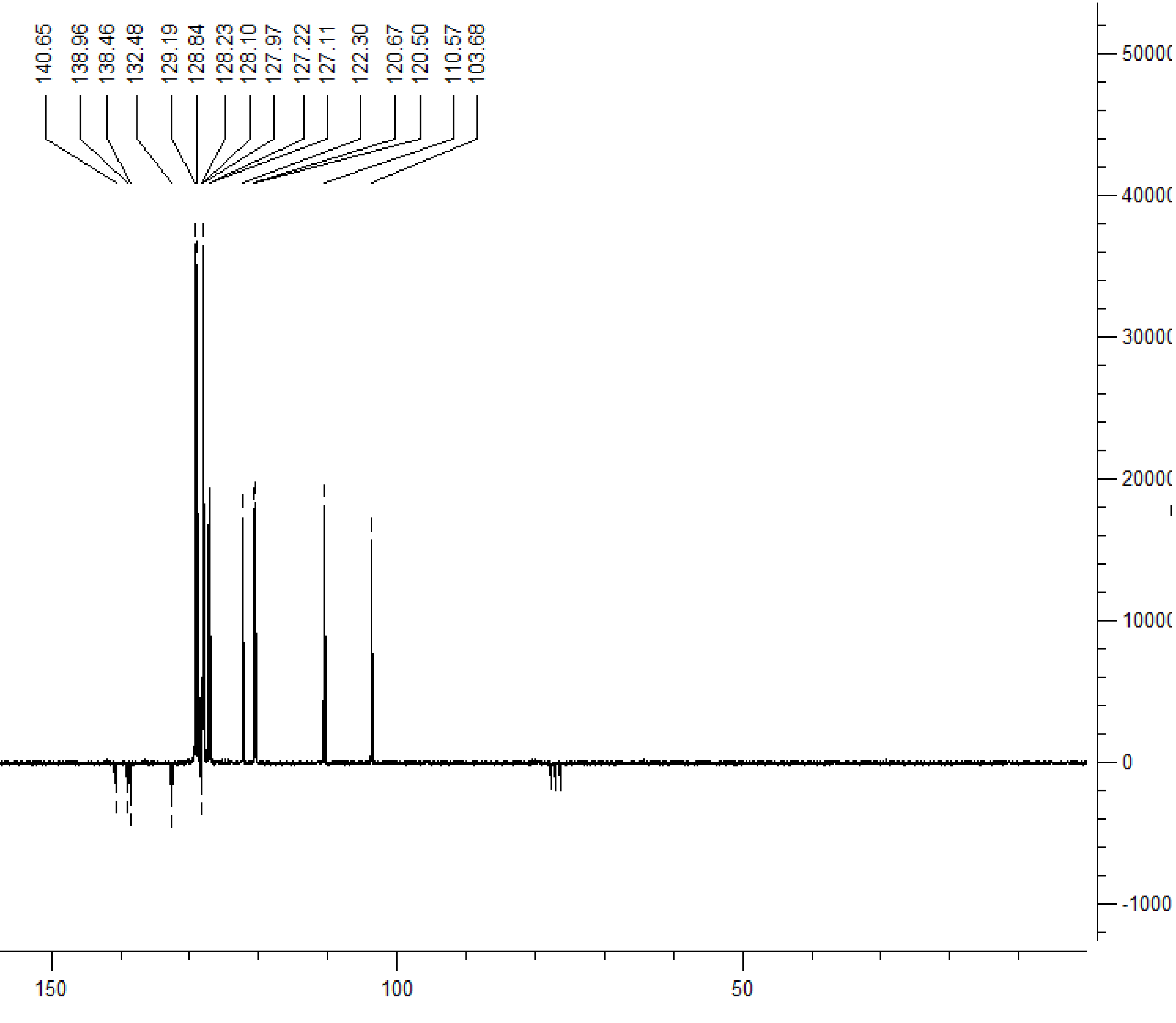
140.65
138.96
138.46
132.48
129.19
128.84
128.23
128.10
127.97
127.22
127.11
122.30
120.67
120.50
110.57
103.68
50000
40000
30000
20000
10000
0
-1000
150
100
50
ppm (t1)

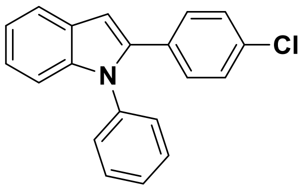

8b

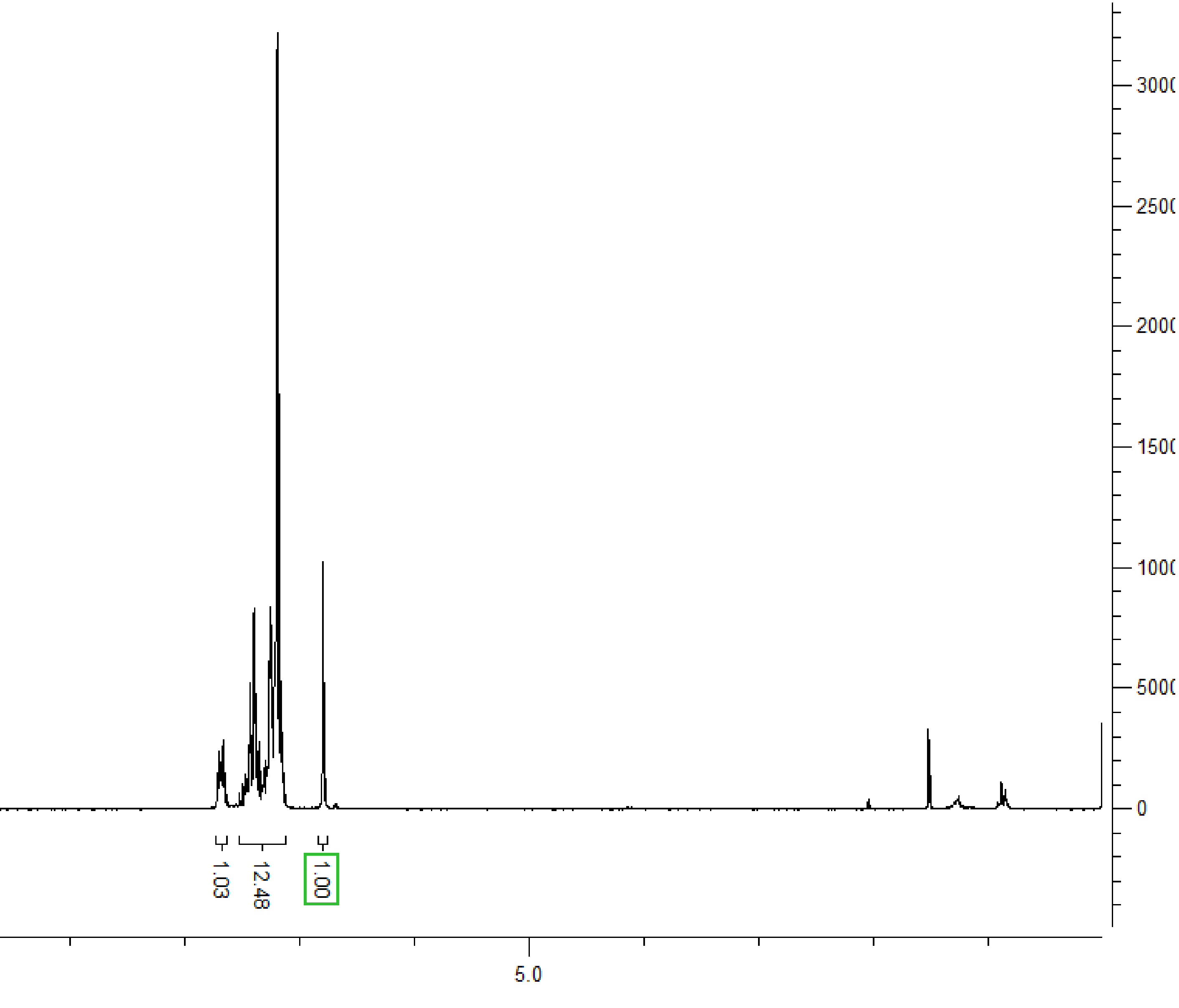

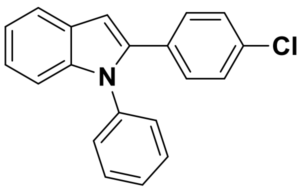

8b

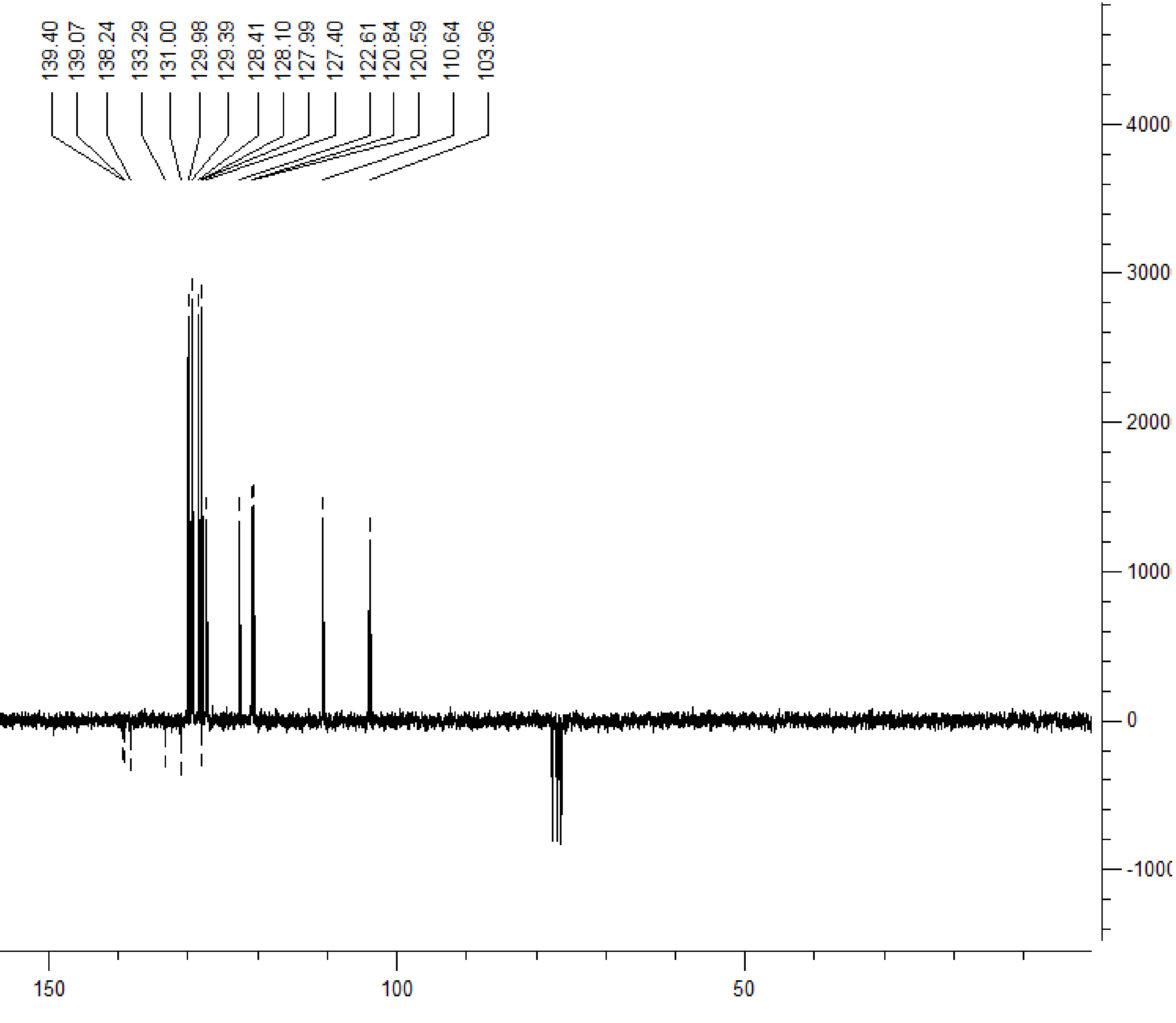
139.40
139.07
138.24
133.29
131.00
129.98
129.39
128.41
128.10
127.99
127.40
122.61
120.84
120.59
110.64
103.96
4000
3000
2000
1000
0
-100(
150
100
50
ɔpm (t1)

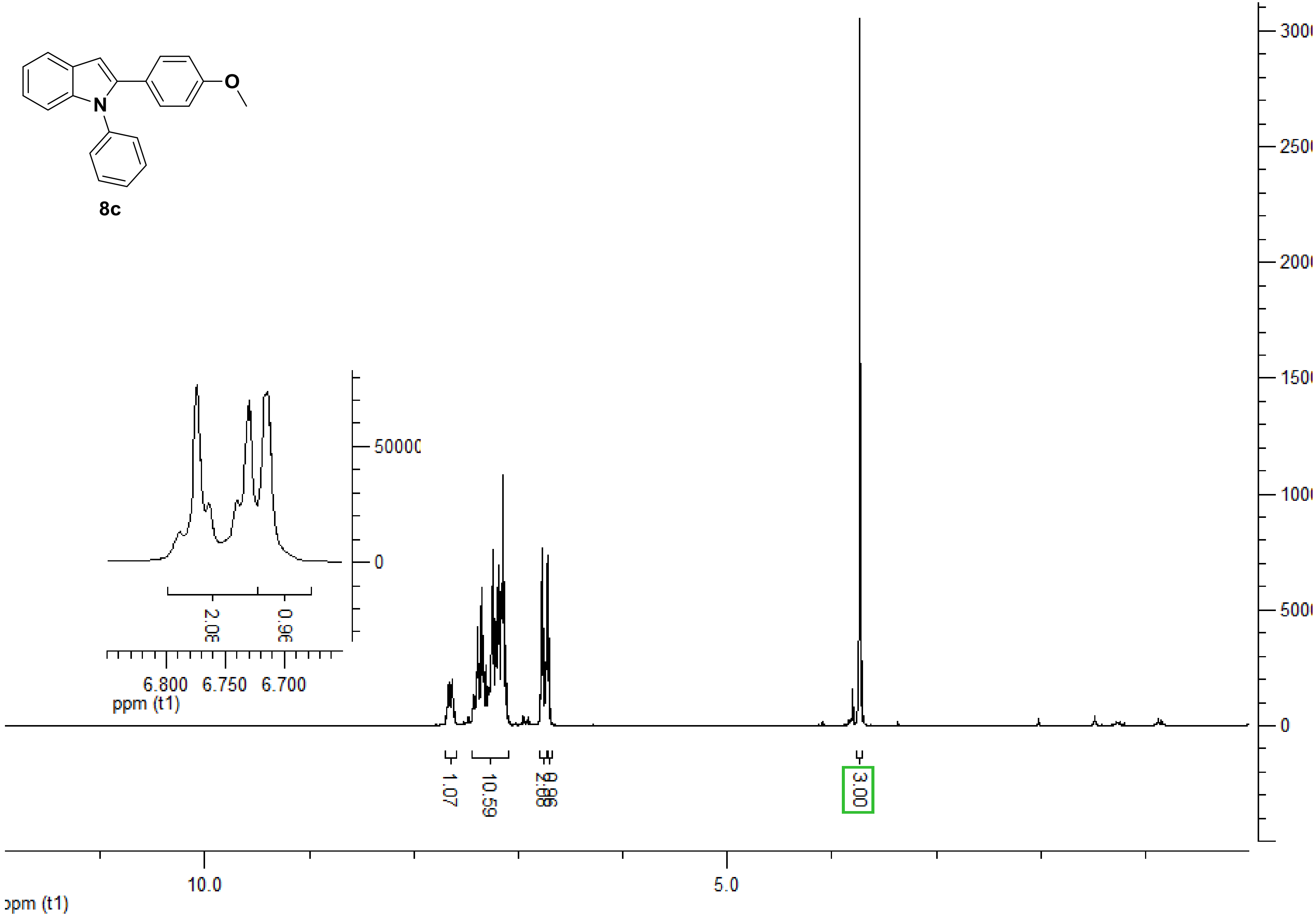
O
N
8c
3000
2500
2000
1500
1000
500
0
50000
0
2.08
0.96
6.800
6.750
6.700
ppm (t1)
1.07
10.59
2.06
0.98
3.00
10.0
5.0
ppm (t1)

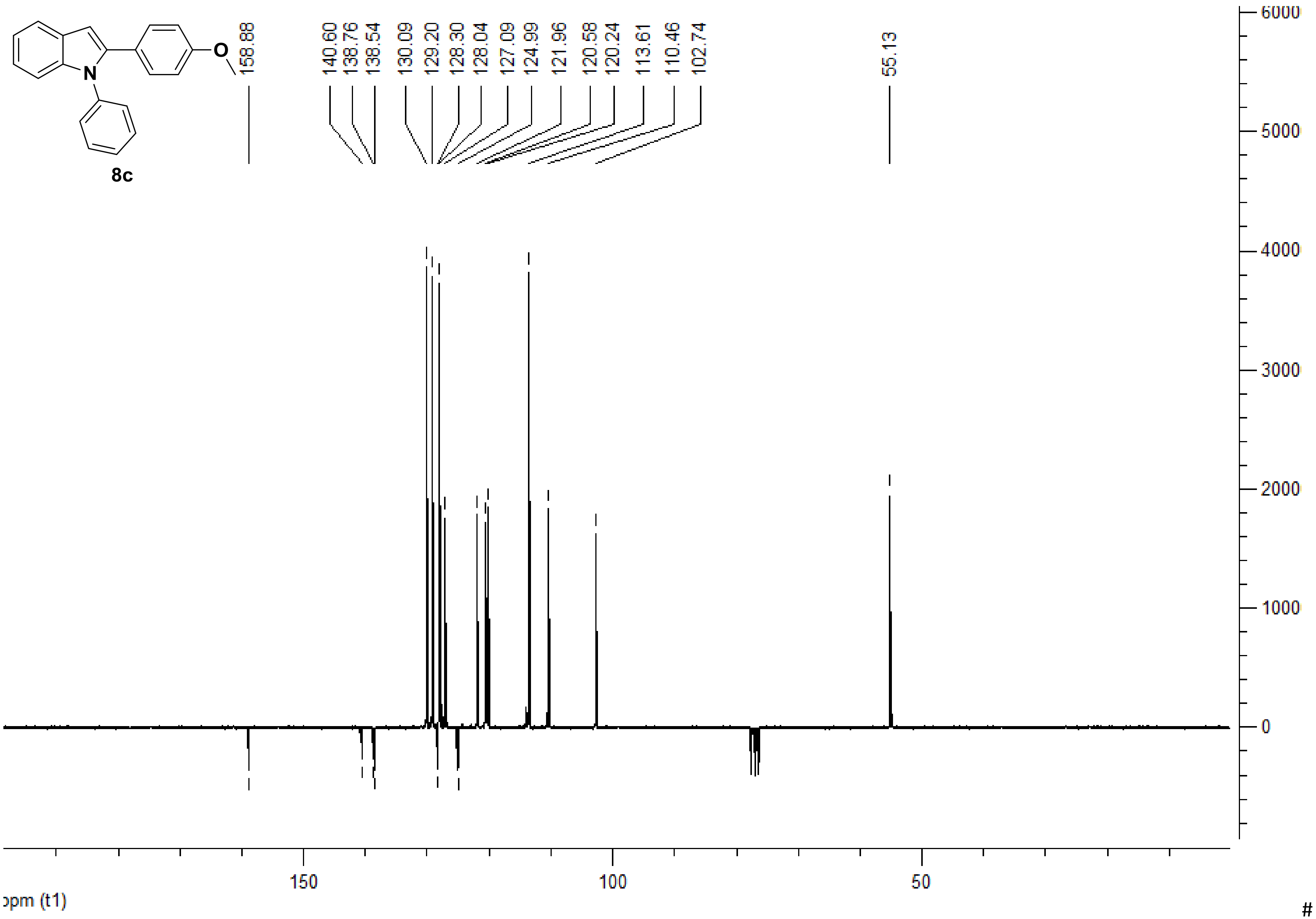

8c
158.88
140.60
138.76
138.54
130.09
129.20
128.30
128.04
127.09
124.99
121.96
120.58
120.24
113.61
110.46
102.74
55.13
6000
5000
4000
3000
2000
1000
0
150
100
50
ppm (t1)

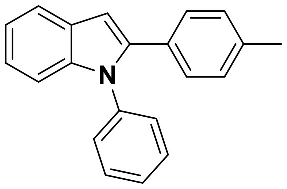

8d

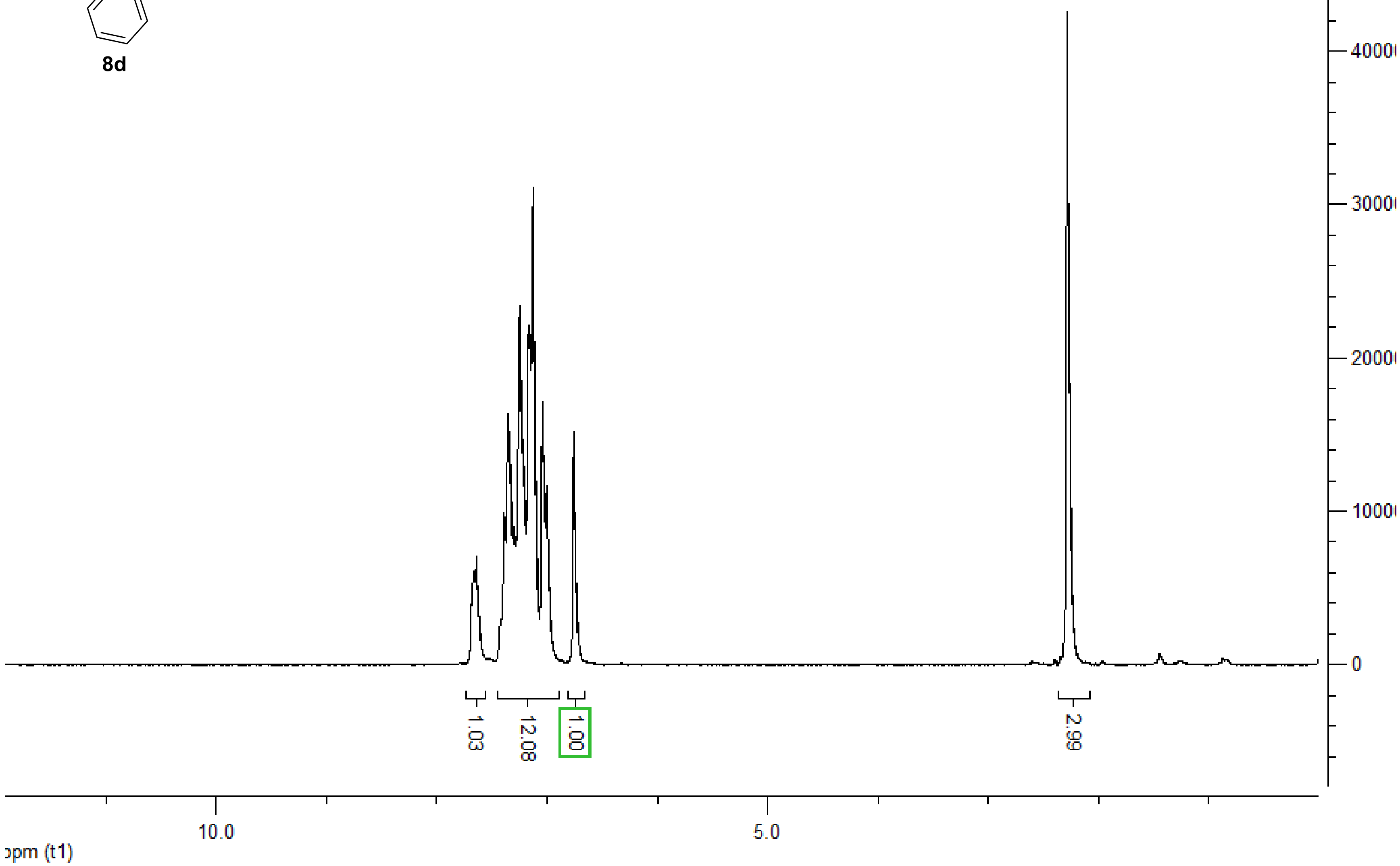

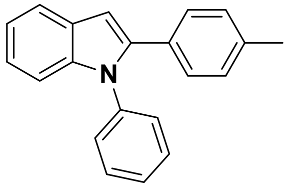

8d

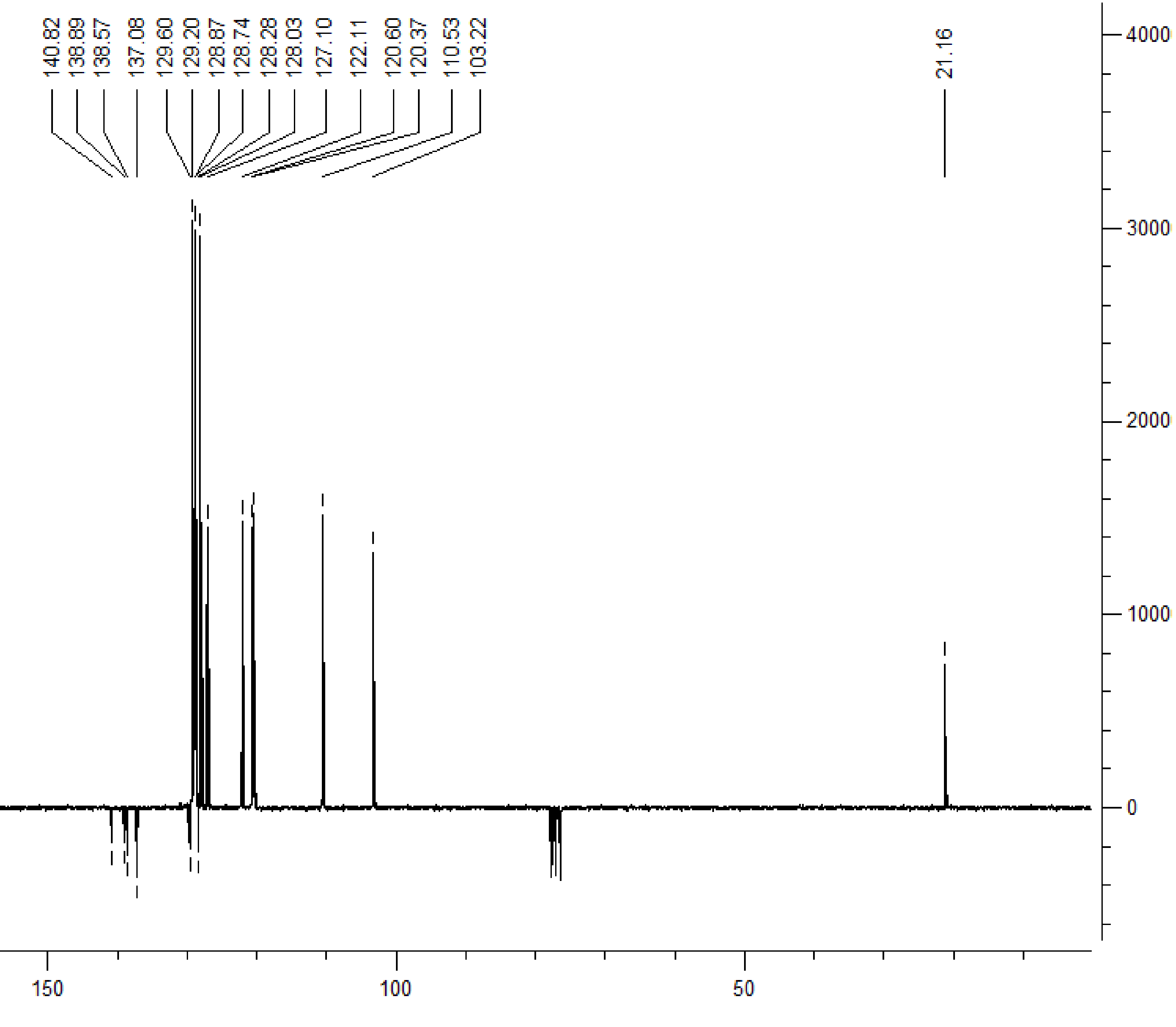

140.82
138.89
138.57
137.08
129.60
129.20
128.87
128.74
128.28
128.03
127.10
122.11
120.60
120.37
110.53
103.22
21.16
4000
3000
2000
1000
0
150
100
50
ppm (t1)

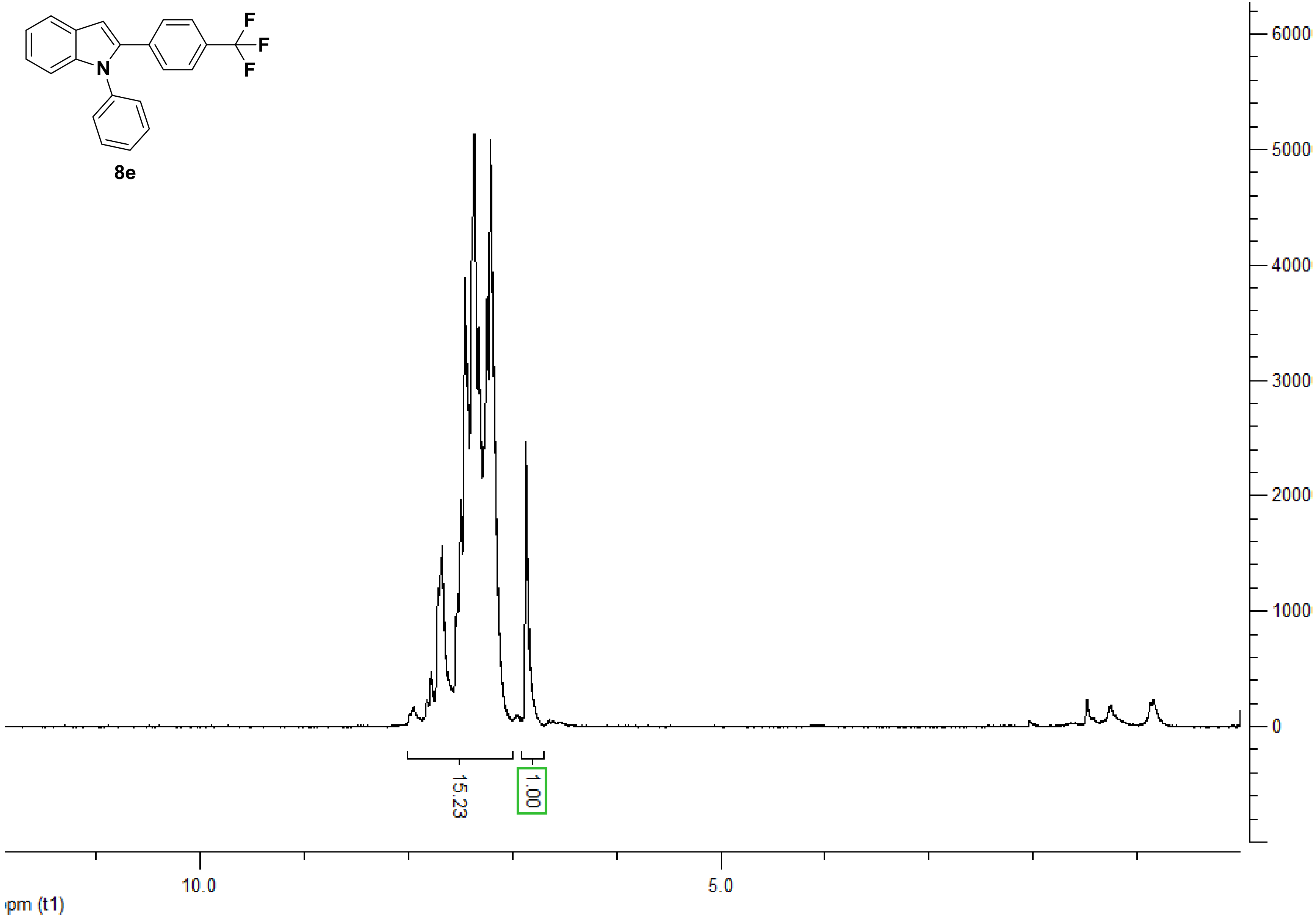
8e
F
F
F
N
6000
5000
4000
3000
2000
1000
0
15.23
1.00
10.0
5.0
ppm (t1)

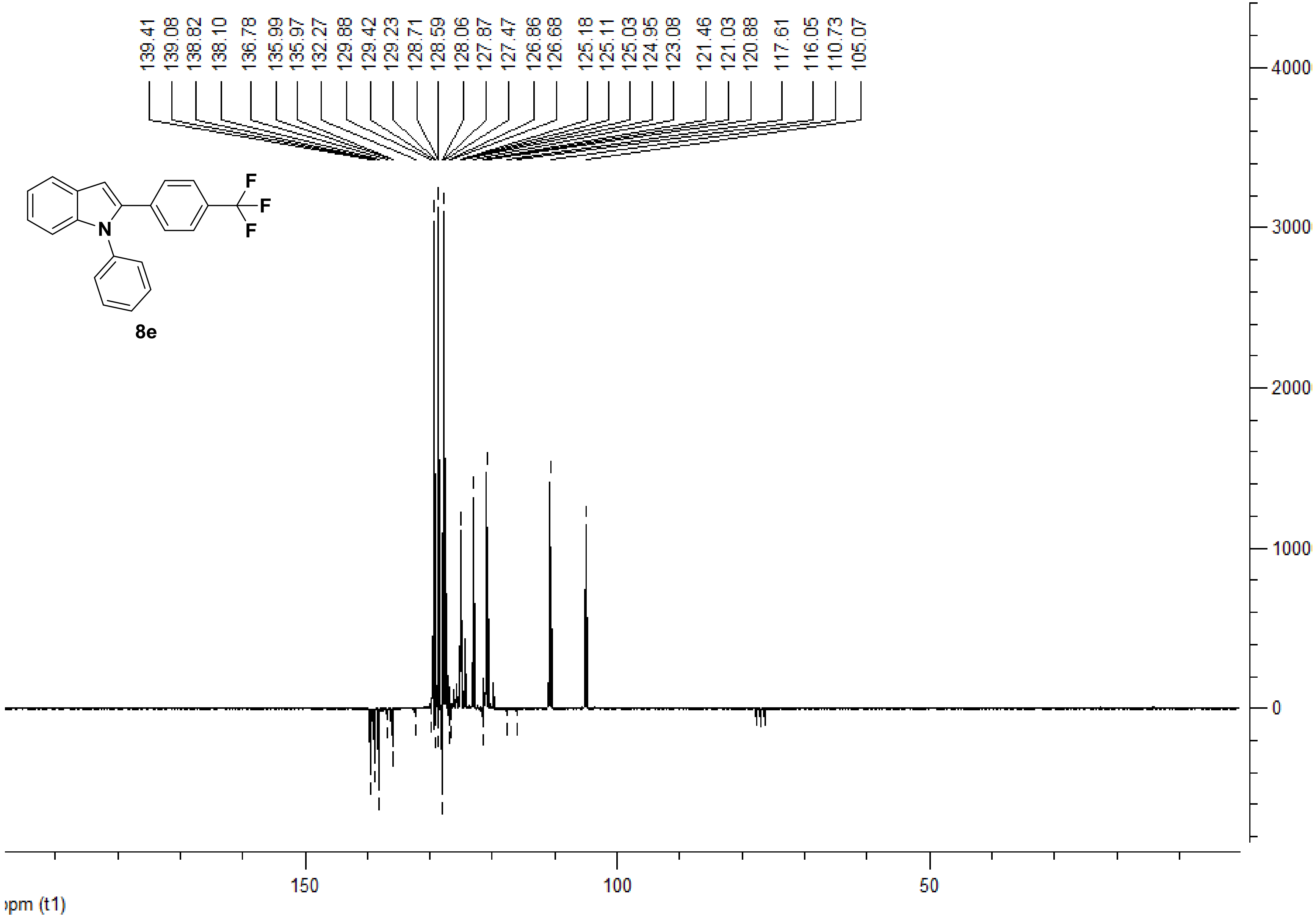
139.41
139.08
138.82
138.10
136.78
135.99
135.97
132.27
129.88
129.42
129.23
128.71
128.59
128.06
127.87
127.47
126.86
126.68
125.18
125.11
125.03
124.95
123.08
121.46
121.03
120.88
117.61
116.05
110.73
105.07
4000
3000
2000
1000
0
150
100
50
ppm (t1)

8e

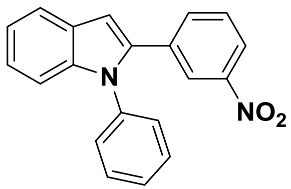

N
NO2

8f

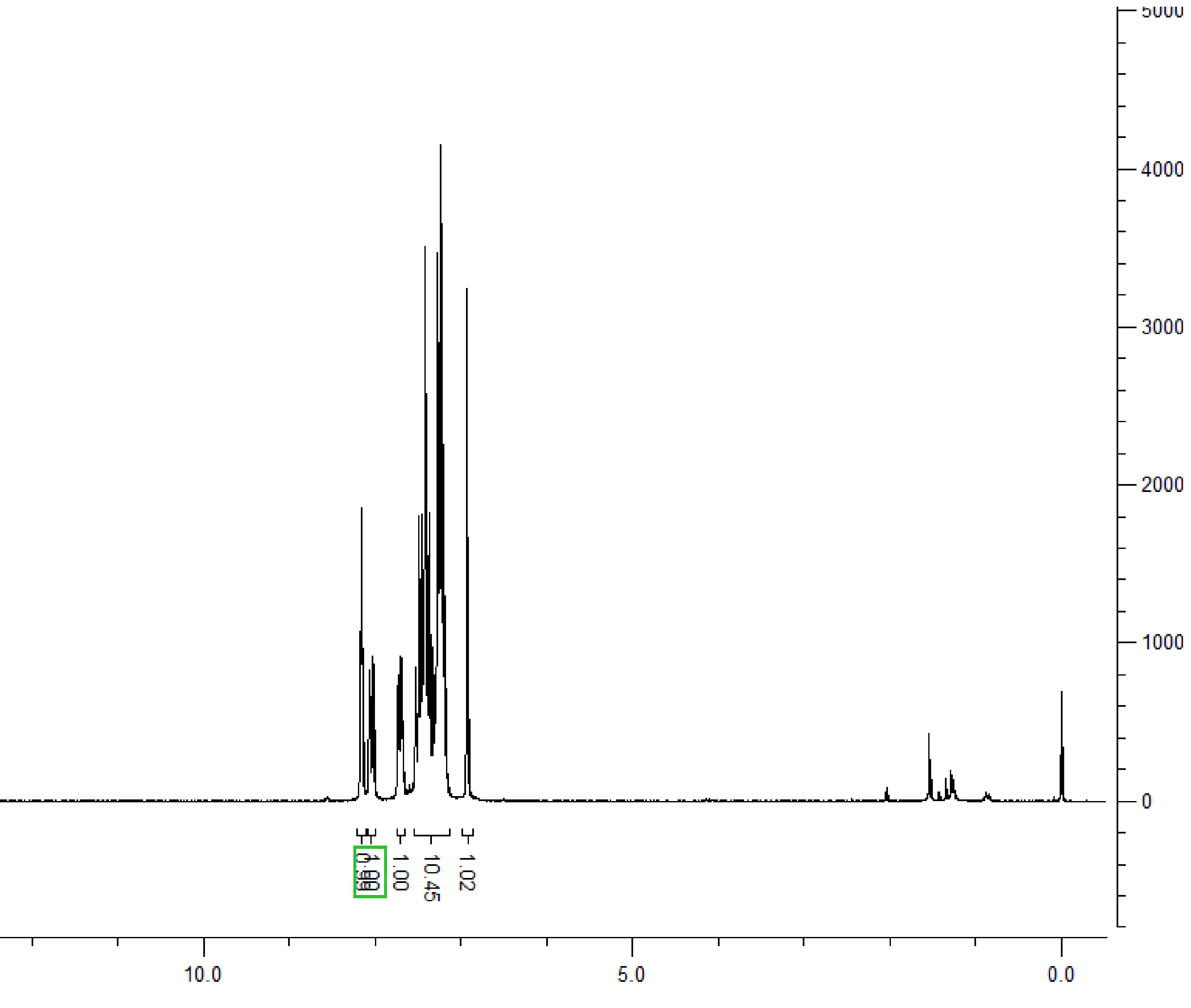

5000
4000
3000
2000
1000
0
1.00
10.45
1.02
15.0
10.0
5.0
0.0
ppm (t1)

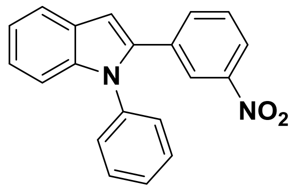

8f

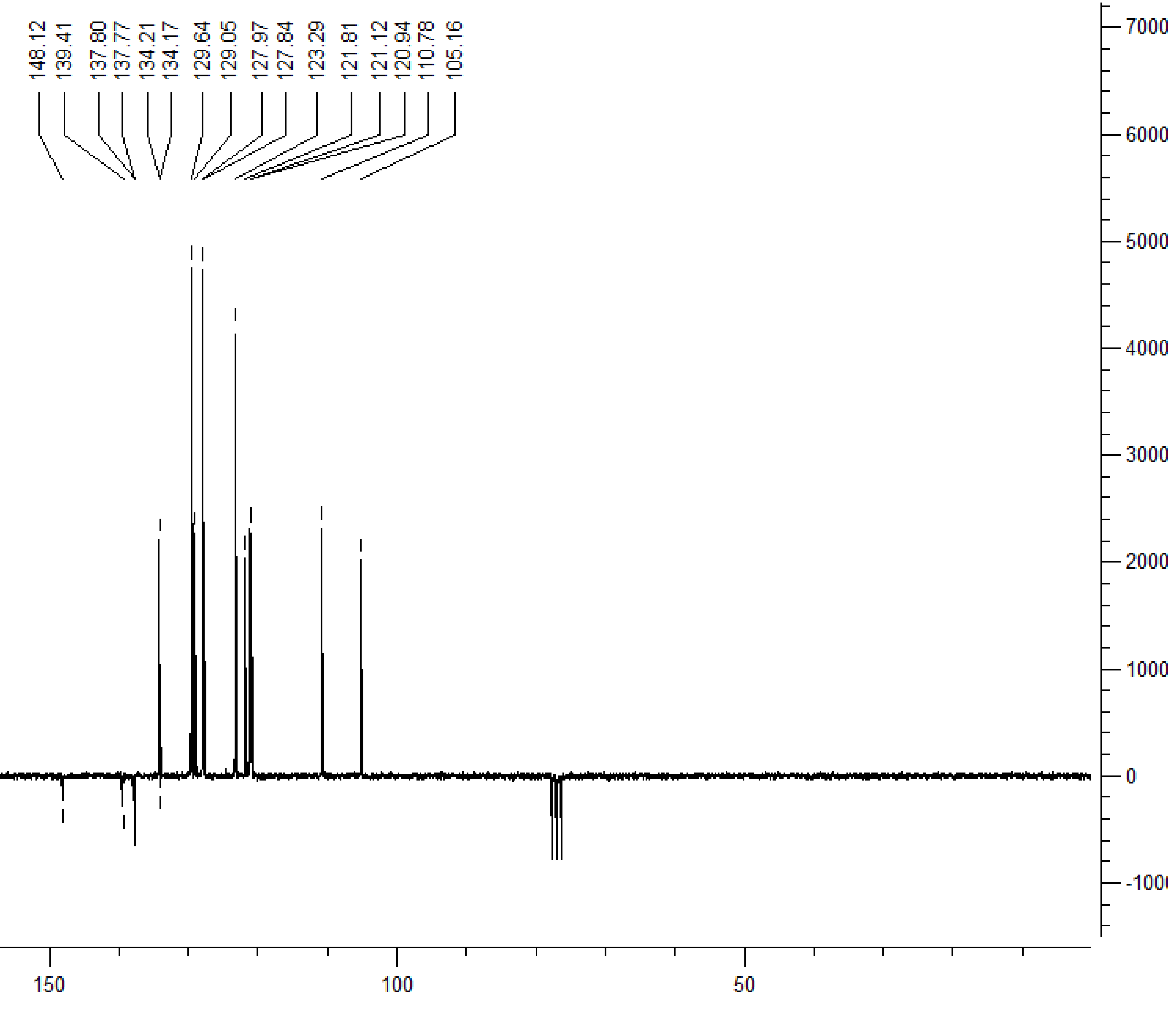

148.12
139.41
137.80
137.77
134.21
134.17
129.64
129.05
127.97
127.84
123.29
121.81
121.12
120.94
110.78
105.16
7000
6000
5000
4000
3000
2000
1000
0
150
100
50
ppm (t1)

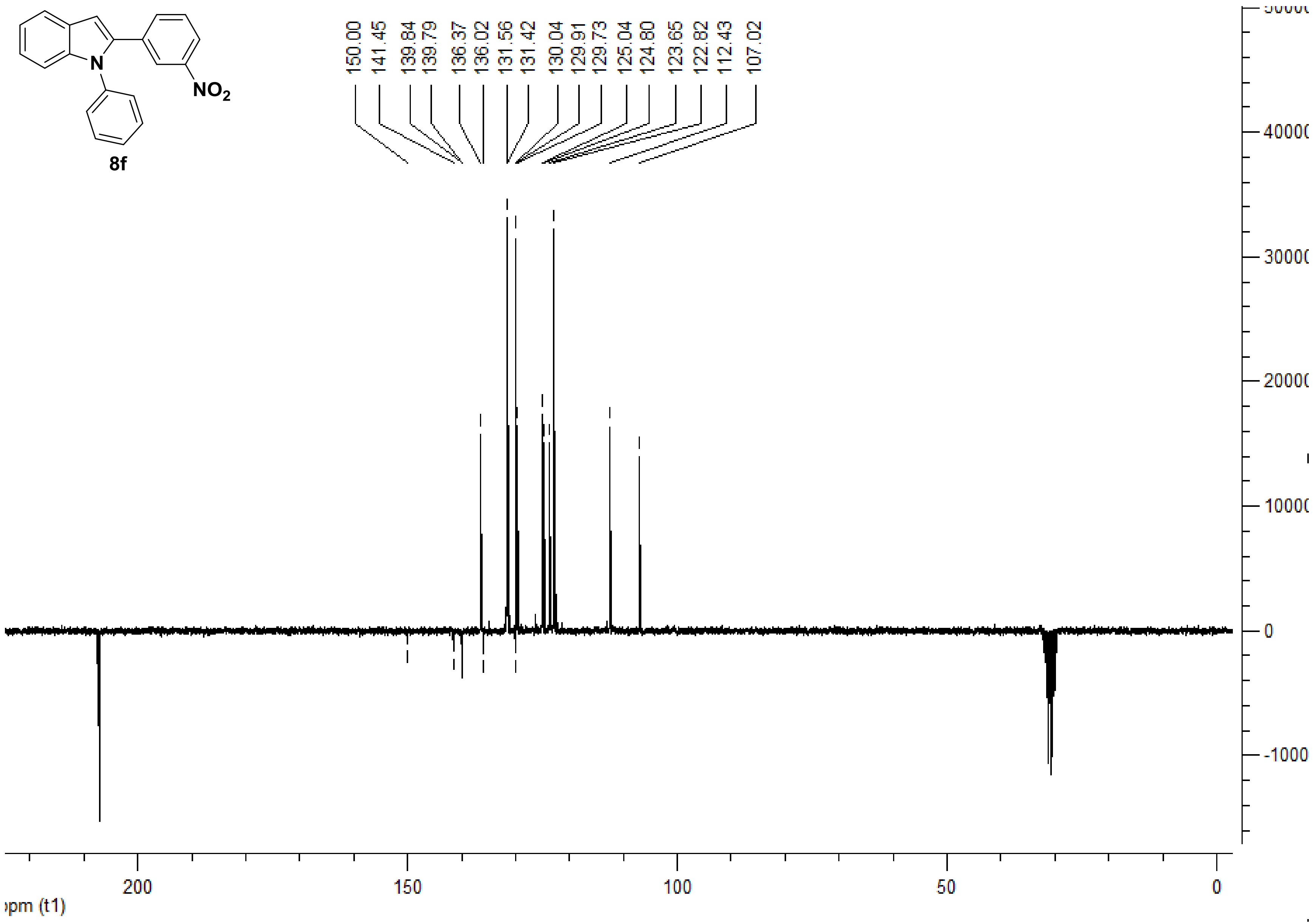
NO2
N
8f
150.00
141.45
139.84
139.79
136.37
136.02
131.56
131.42
130.04
129.91
129.73
125.04
124.80
123.65
122.82
112.43
107.02
200
150
100
50
0
ppm (t1)
0
-1000
10000
20000
30000
40000

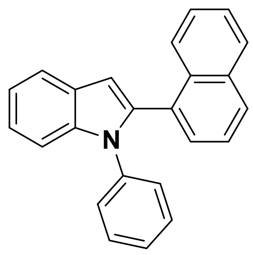

8g

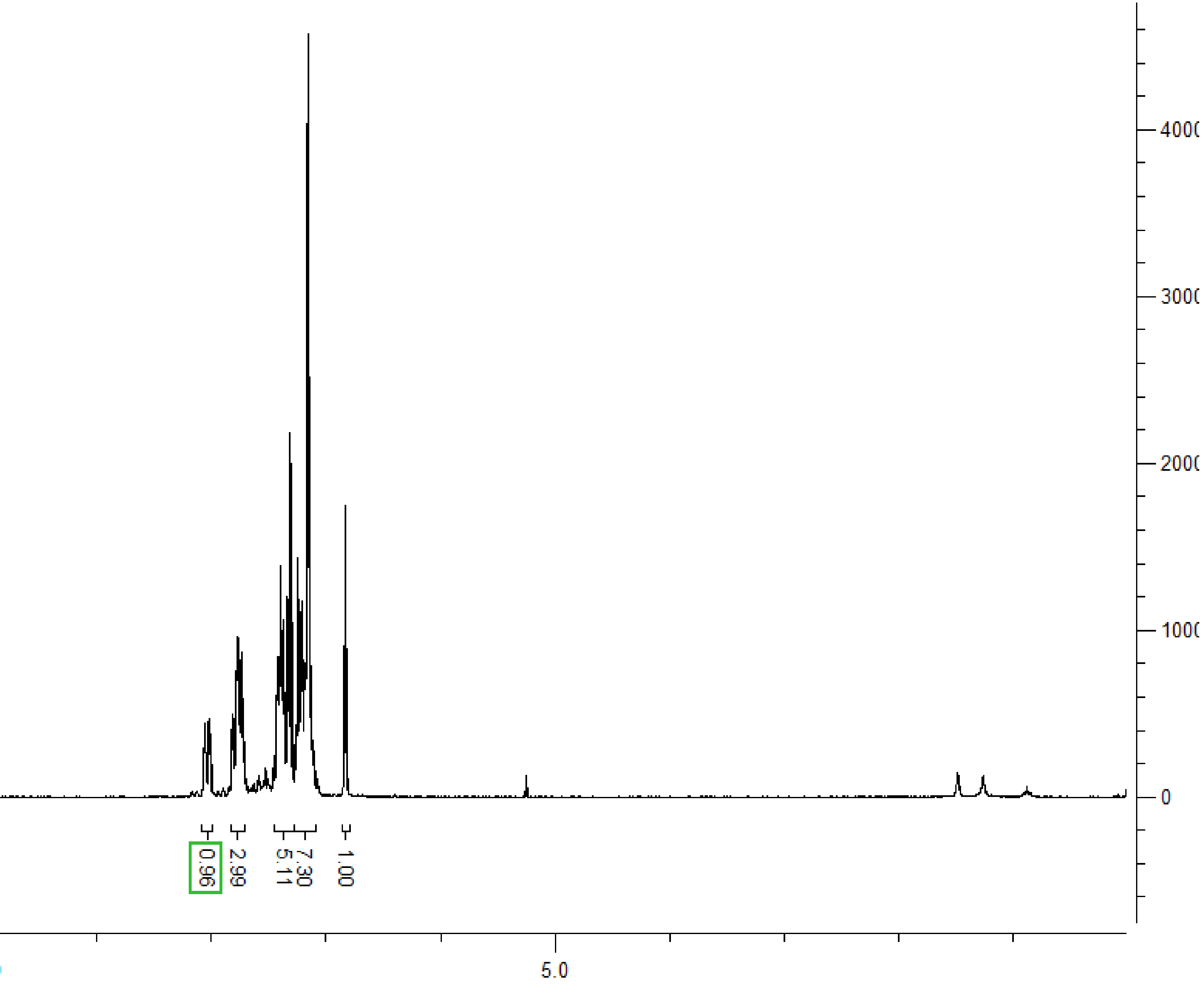

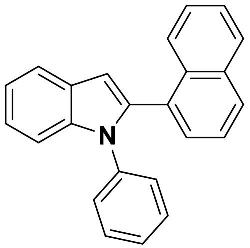

8g

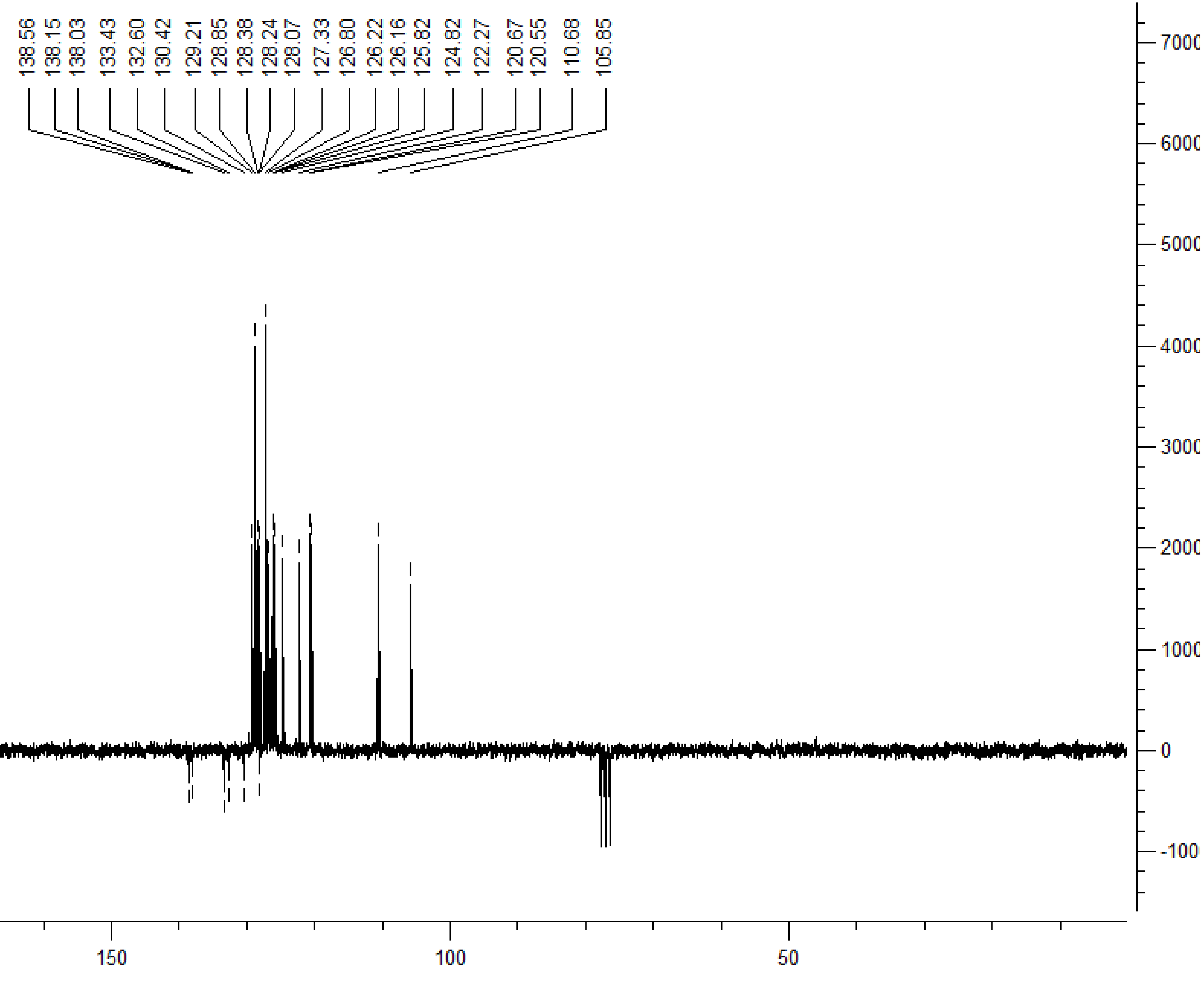
138.56
138.15
138.03
133.43
132.60
130.42
129.21
128.85
128.38
128.24
128.07
127.33
126.80
126.22
126.16
125.82
124.82
122.27
120.67
120.55
110.68
105.85
7000
6000
5000
4000
3000
2000
1000
0
-100
150
100
50
ppm (t1)

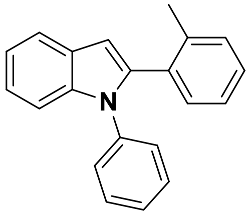

8h

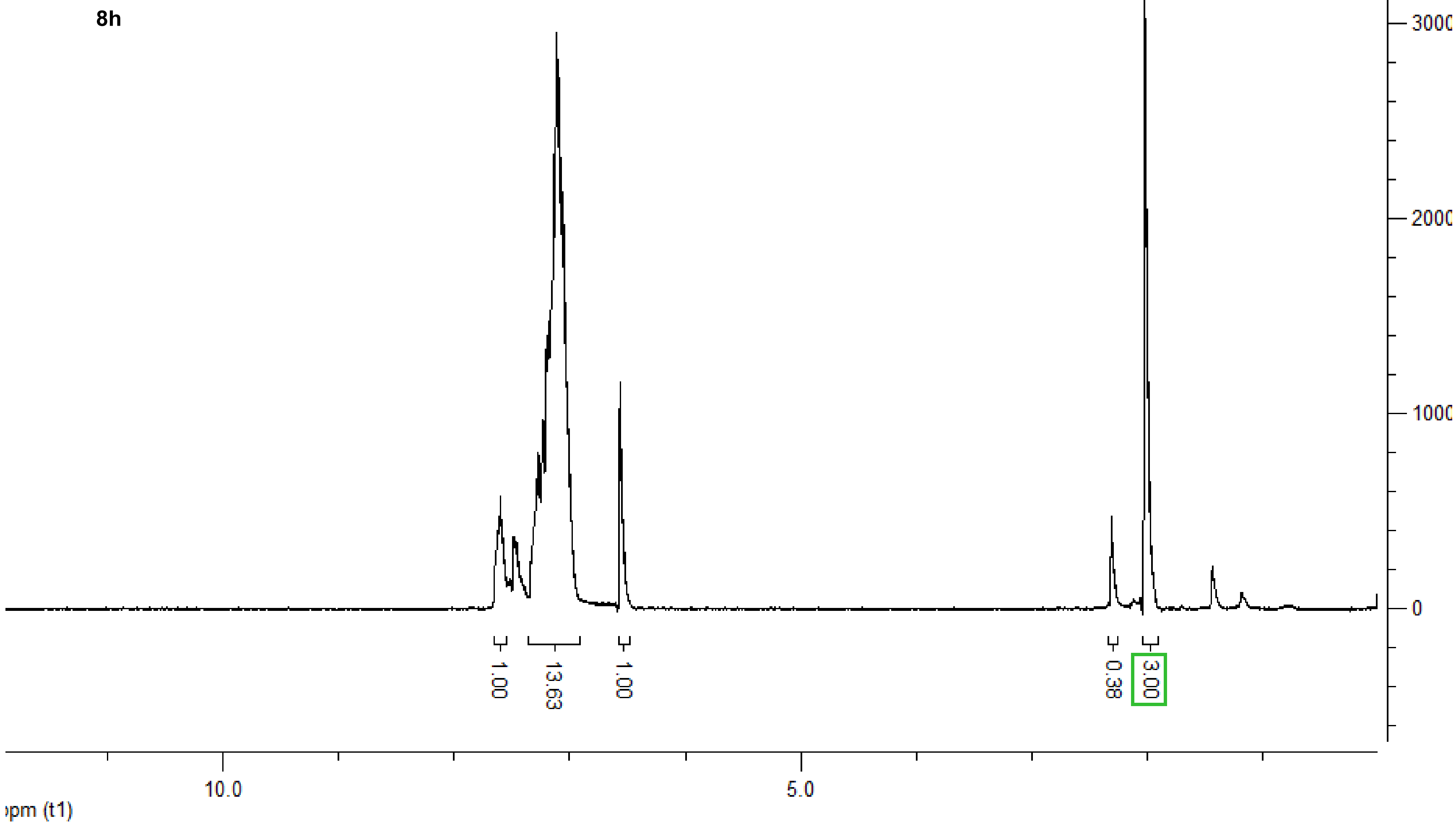

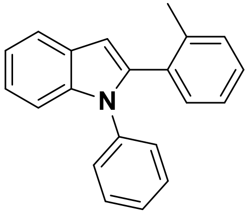

8h

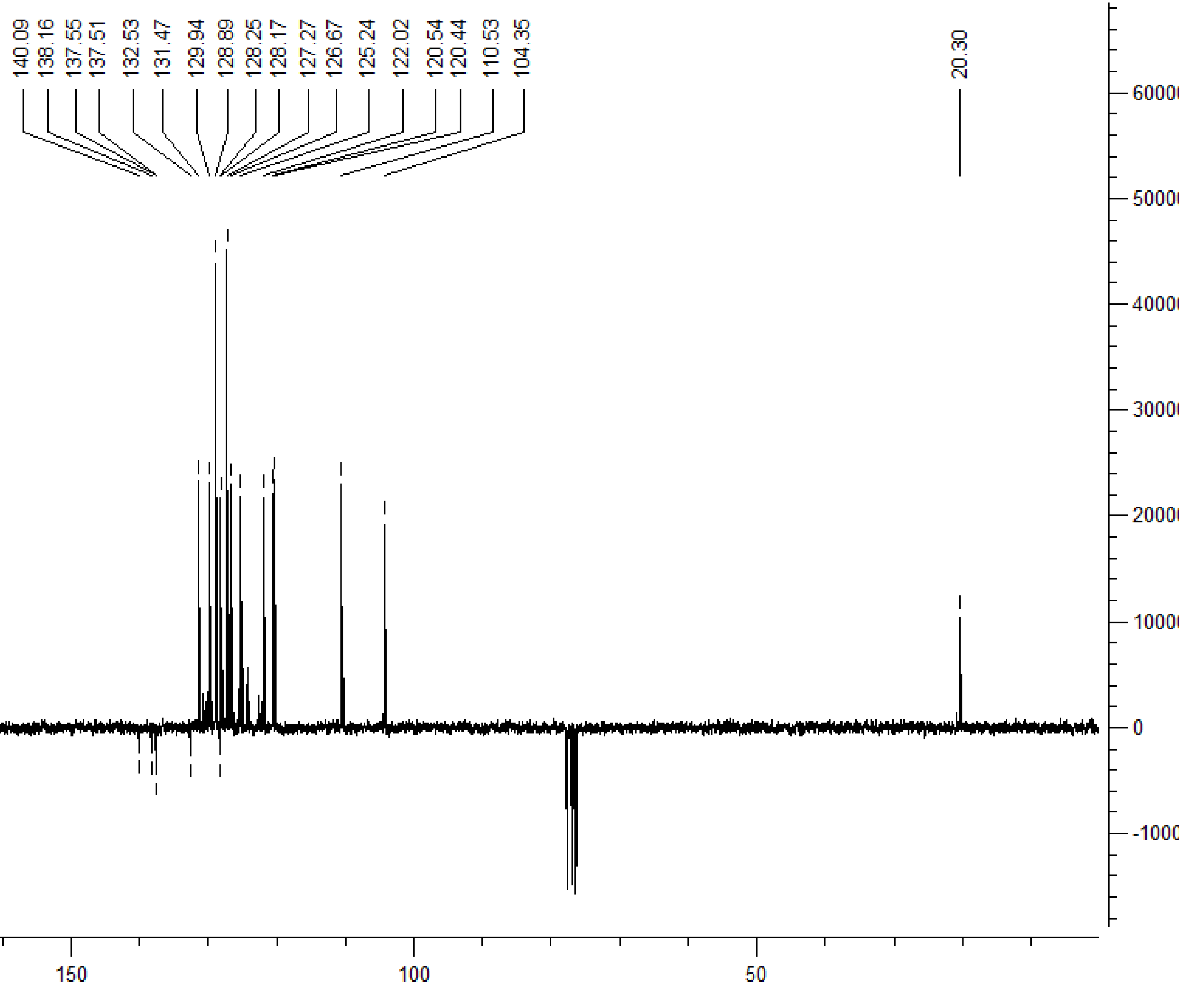
140.09
138.16
137.55
137.51
132.53
131.47
129.94
128.89
128.25
128.17
127.27
126.67
125.24
122.02
120.54
120.44
110.53
104.35
20.30
60000
50000
40000
30000
20000
10000
0
-1000
150
100
50
ppm (t1)

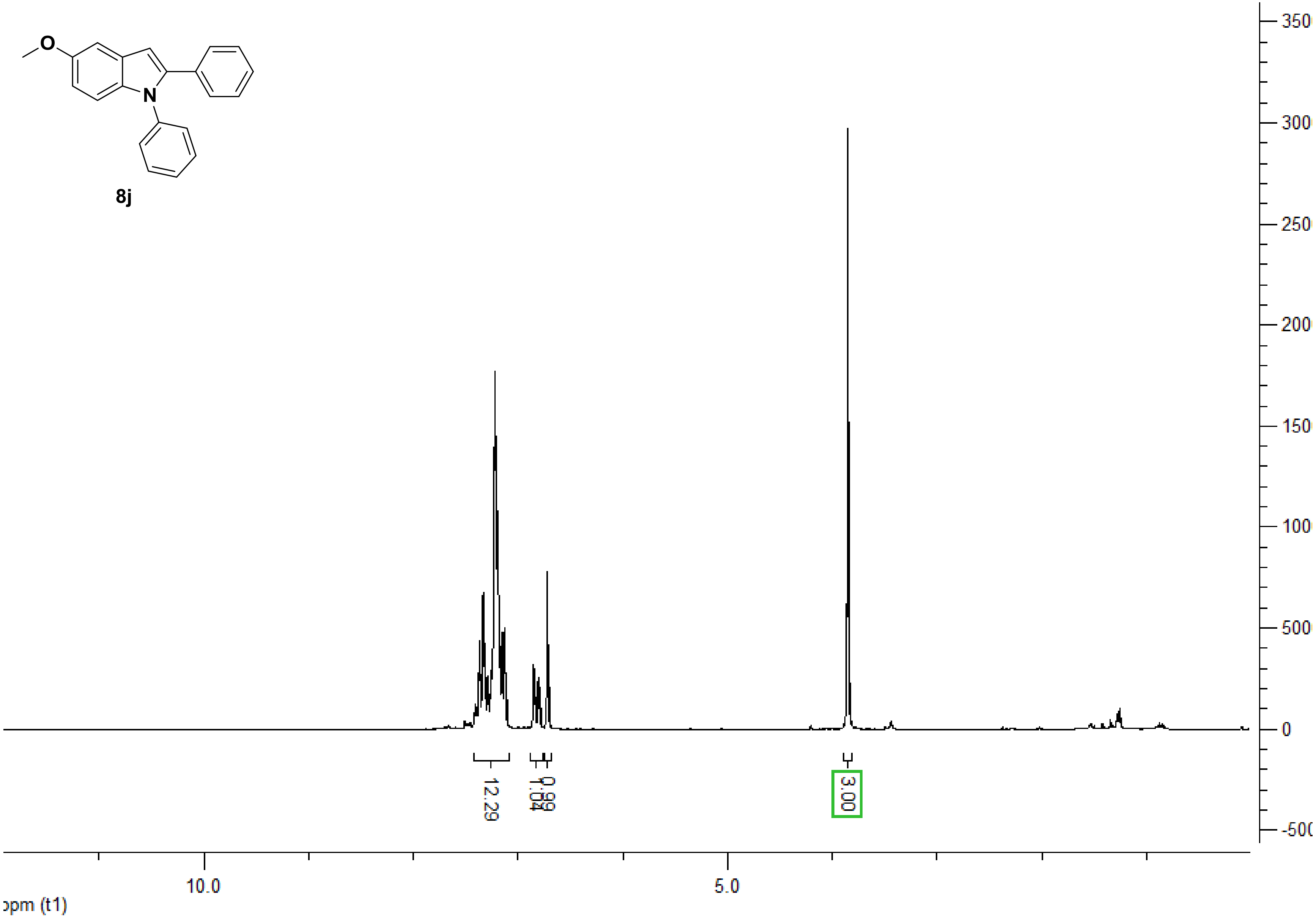

8j

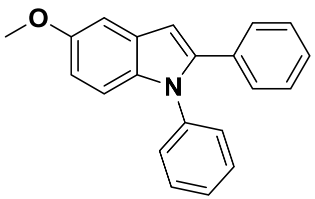

8j

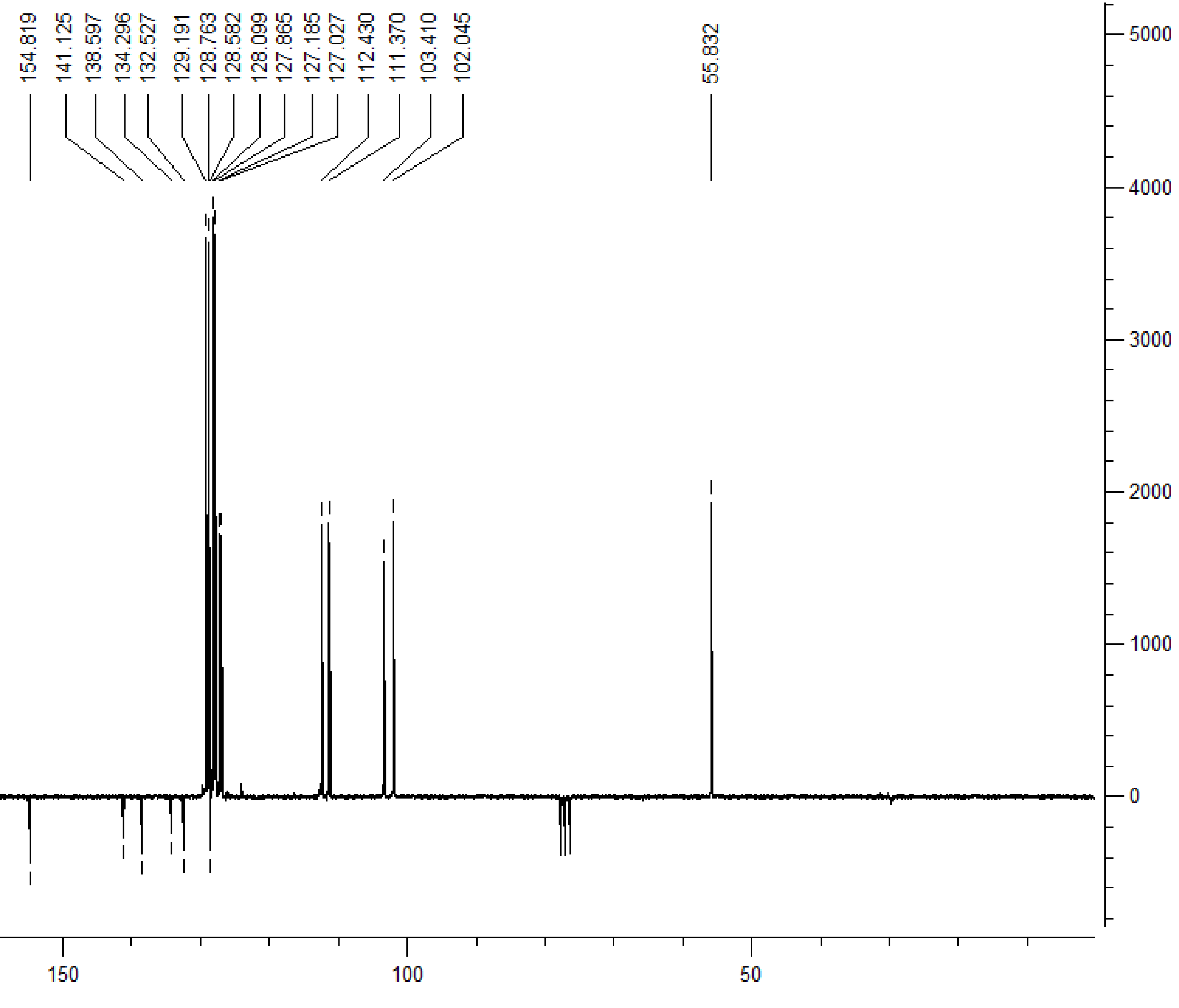

154.819
141.125
138.597
134.296
132.527
129.191
128.763
128.582
128.099
127.865
127.185
127.027
112.430
111.370
103.410
102.045
55.832
5000
4000
3000
2000
1000
0
150
100
50
ppm (t1)

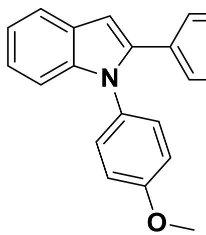

8k

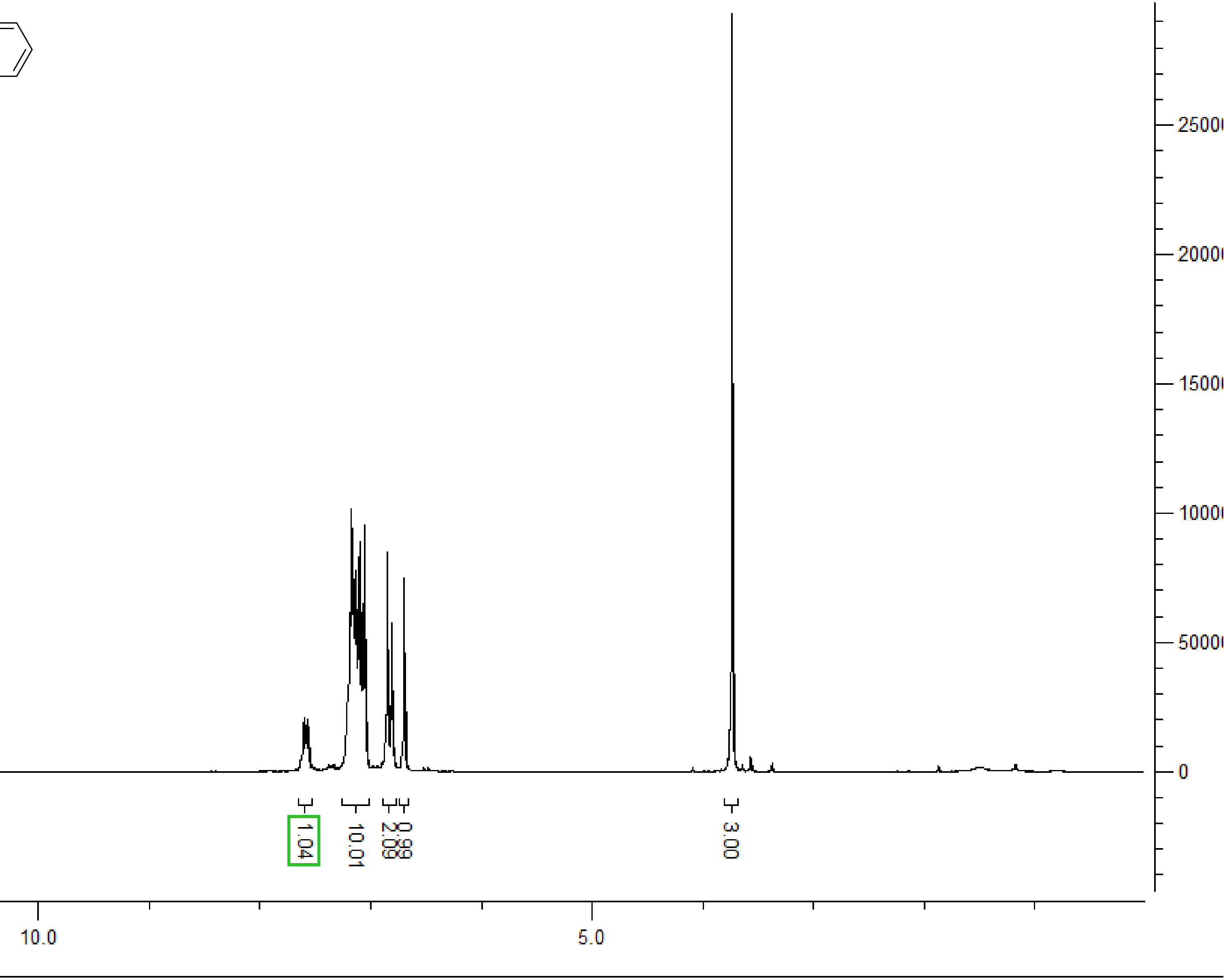

25000
20000
15000
10000
5000
0
1.04
10.01
2.09
0.99
3.00
10.0
5.0
ppm (t1)

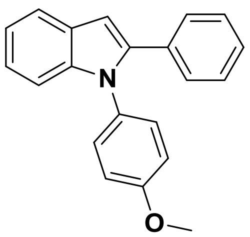
N
O

8k

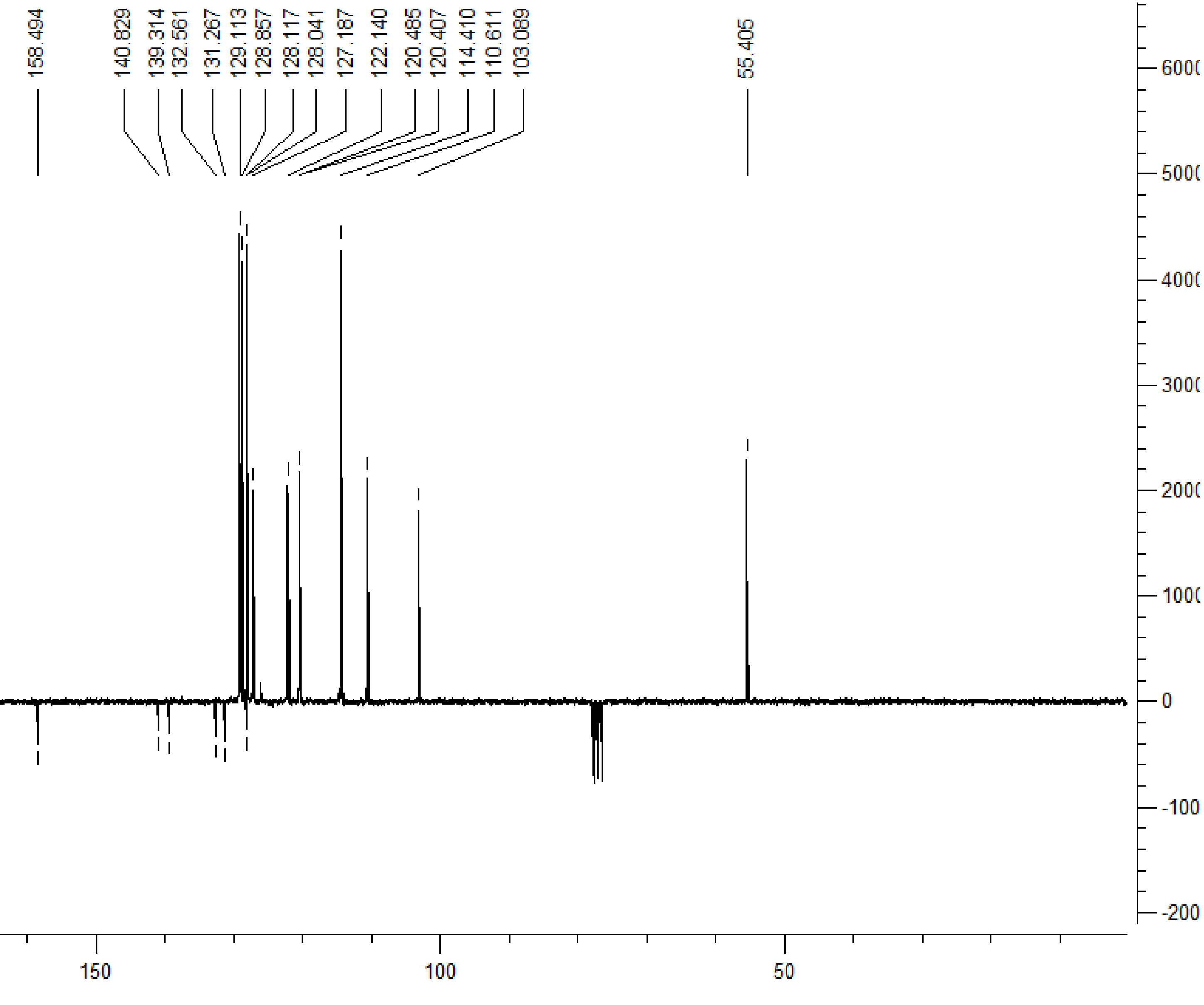
158.494
140.829
139.314
132.561
131.267
129.113
128.857
128.117
128.041
127.187
122.140
120.485
120.407
114.410
110.611
103.089
55.405
600(
500(
400(
300(
200(
100(
0
-100
-200
150
100
50
ppm (t1)

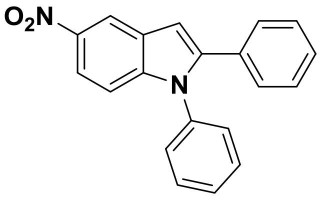
O_2N
N

8n

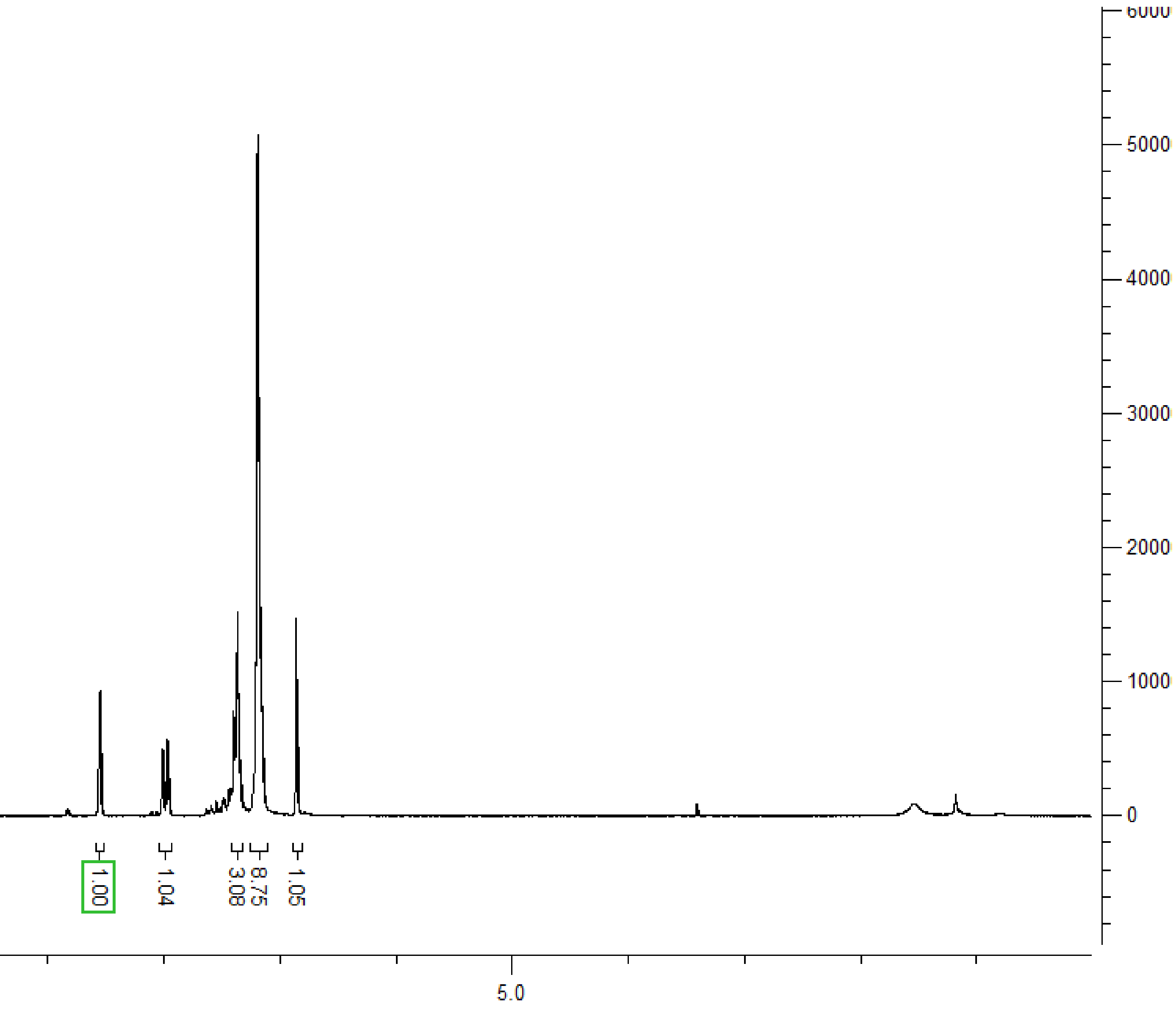
6000
5000
4000
3000
2000
1000
0
1.00
1.04
3.08
8.75
1.05
10.0
5.0
ppm (t1)

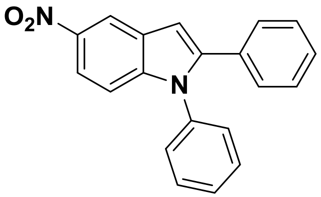

8n

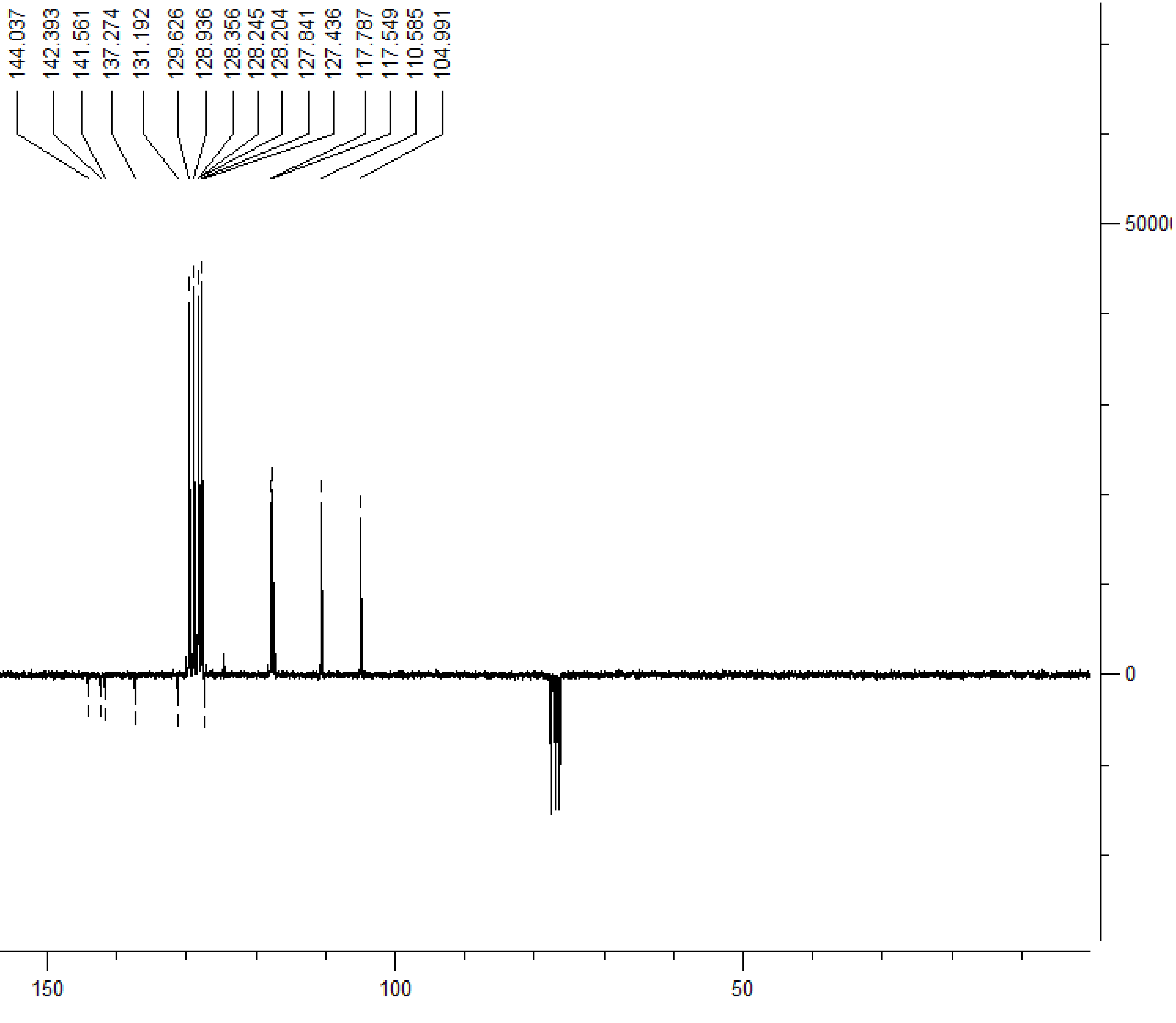
144.037
142.393
141.561
137.274
131.192
129.626
128.936
128.356
128.245
128.204
127.841
127.436
117.787
117.549
110.585
104.991
50000
0
150
100
50
ppm (t1)

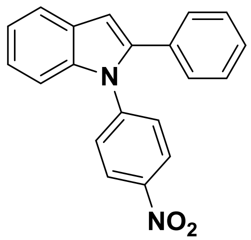
N
NO2

8o

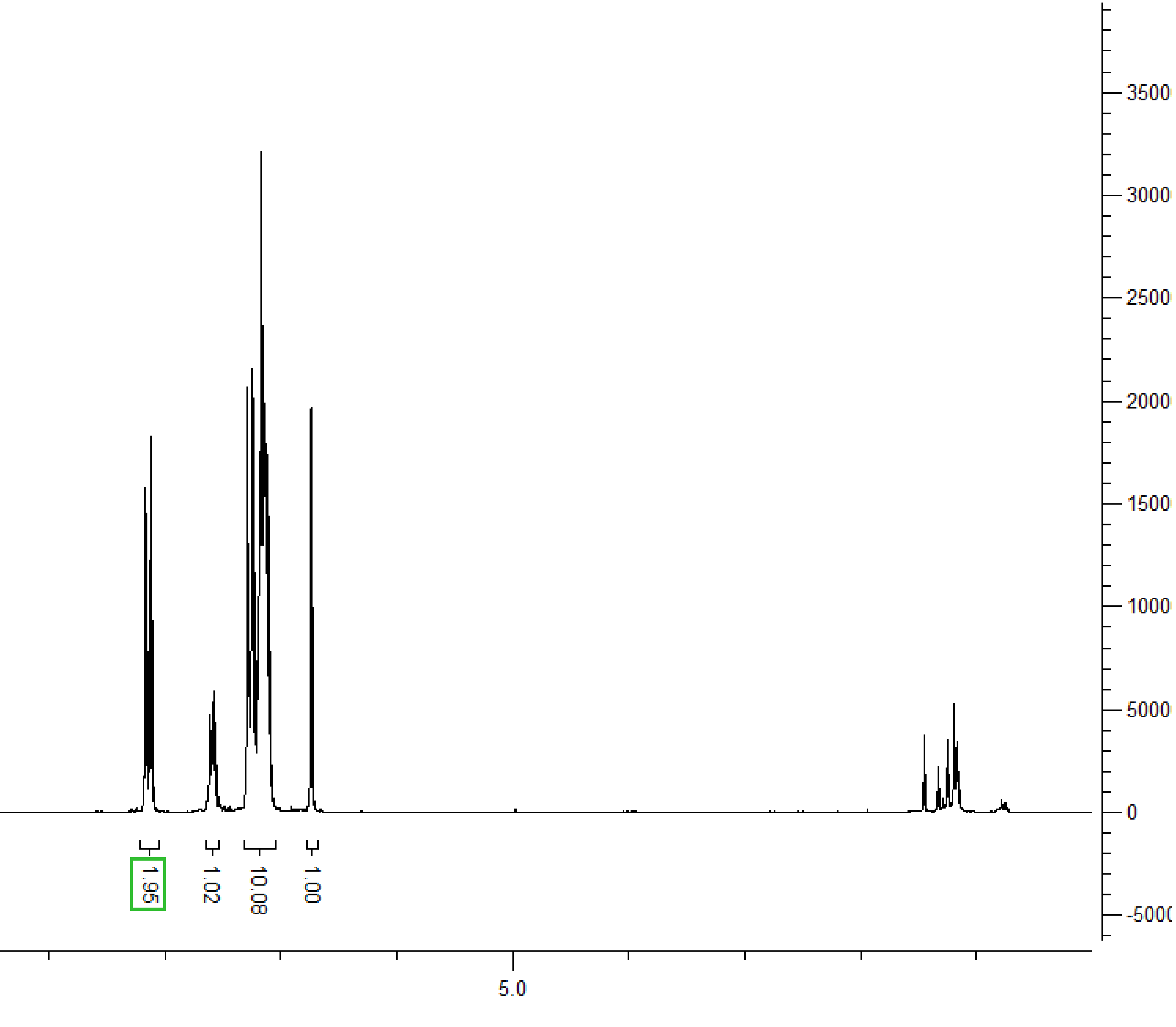
3500
3000
2500
2000
1500
1000
5000
0
-5000
1.95
1.02
10.08
1.00
10.0
5.0
ppm (t1)

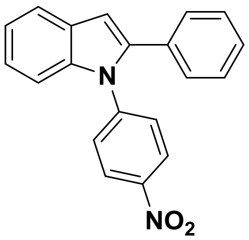

8o

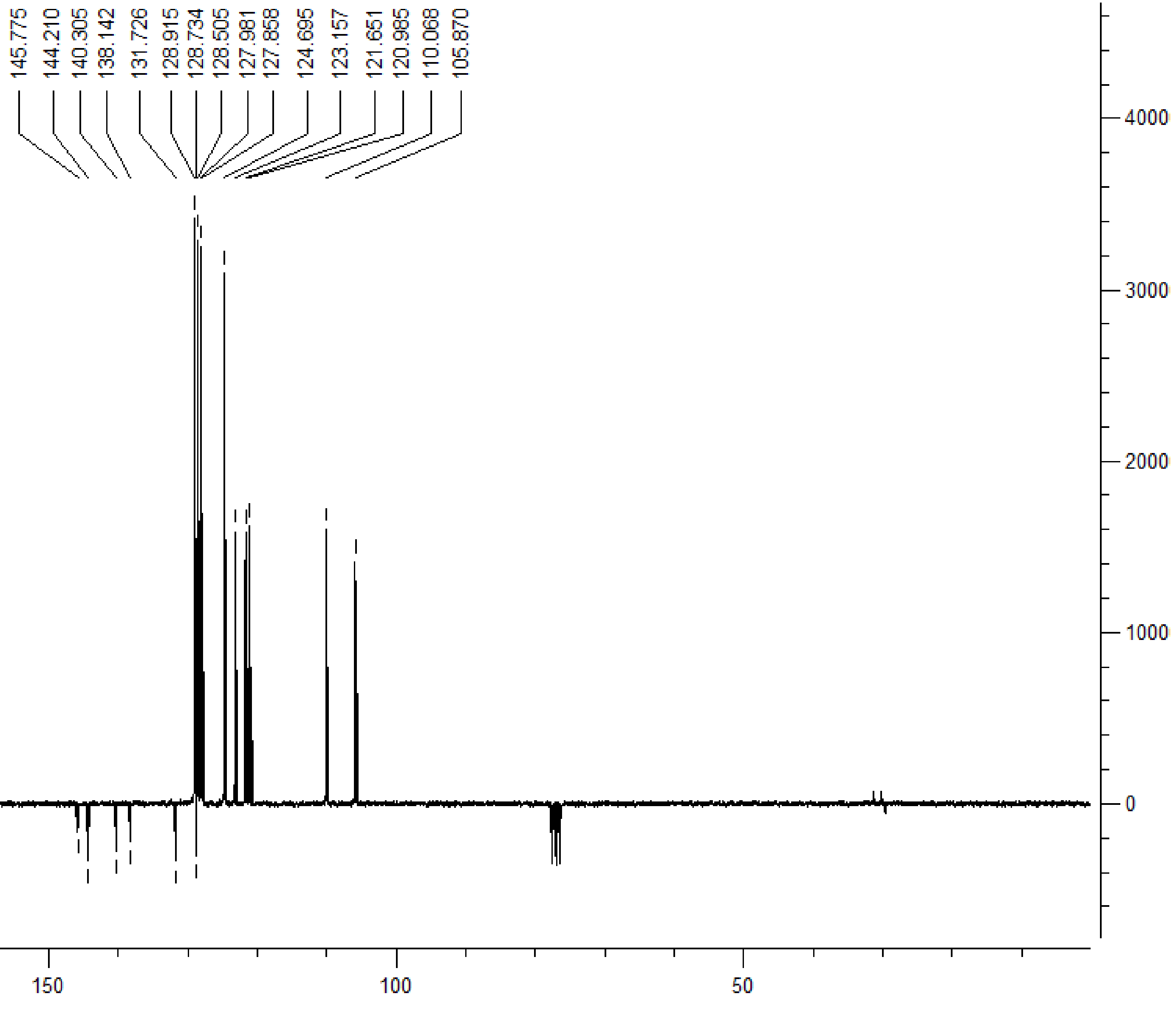

145.775
144.210
140.305
138.142
131.726
128.915
128.734
128.505
127.981
127.858
124.695
123.157
121.651
120.985
110.068
105.870
4000
3000
2000
1000
0
150
100
50
ppm (t1)

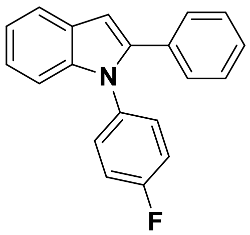

8p

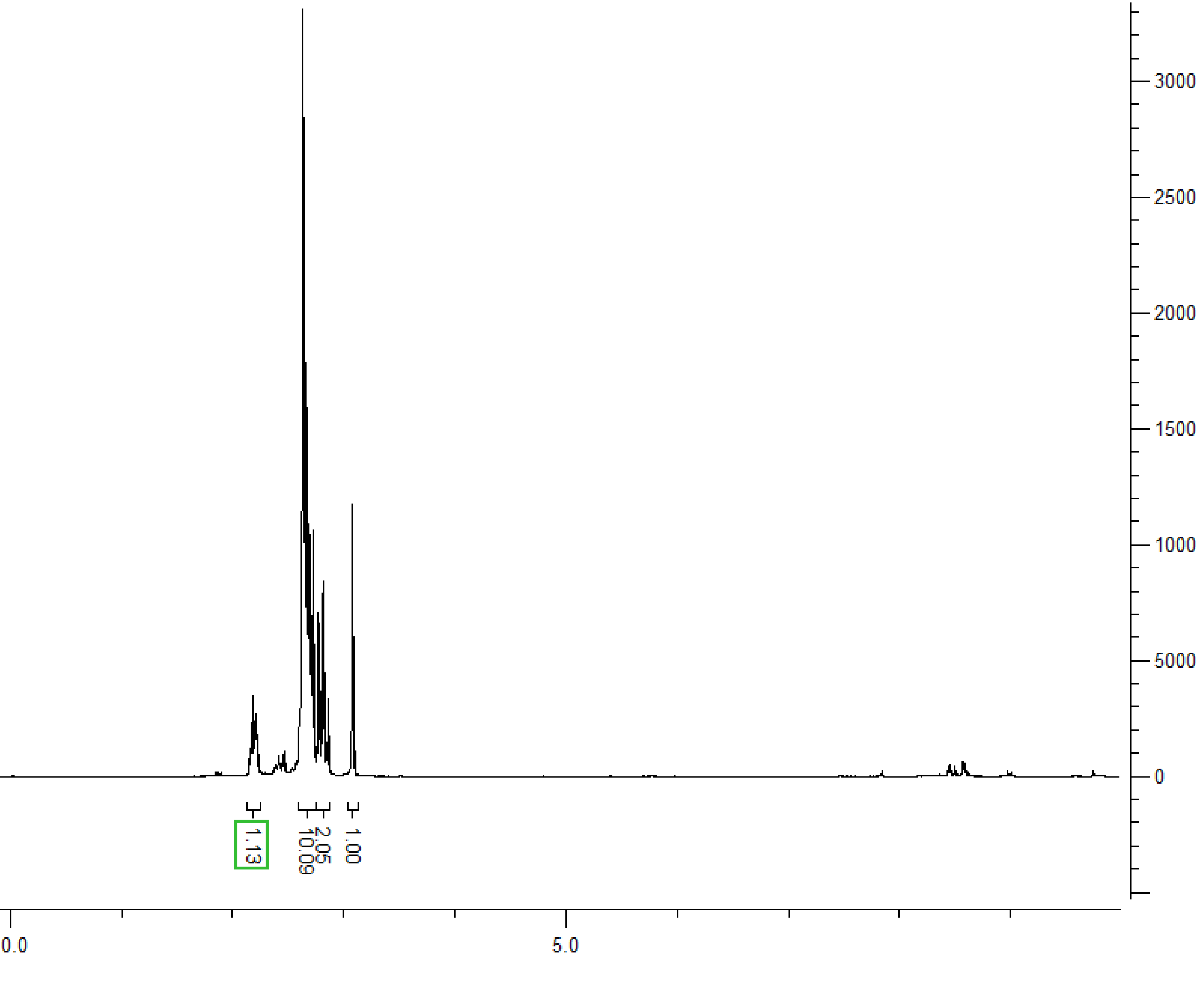

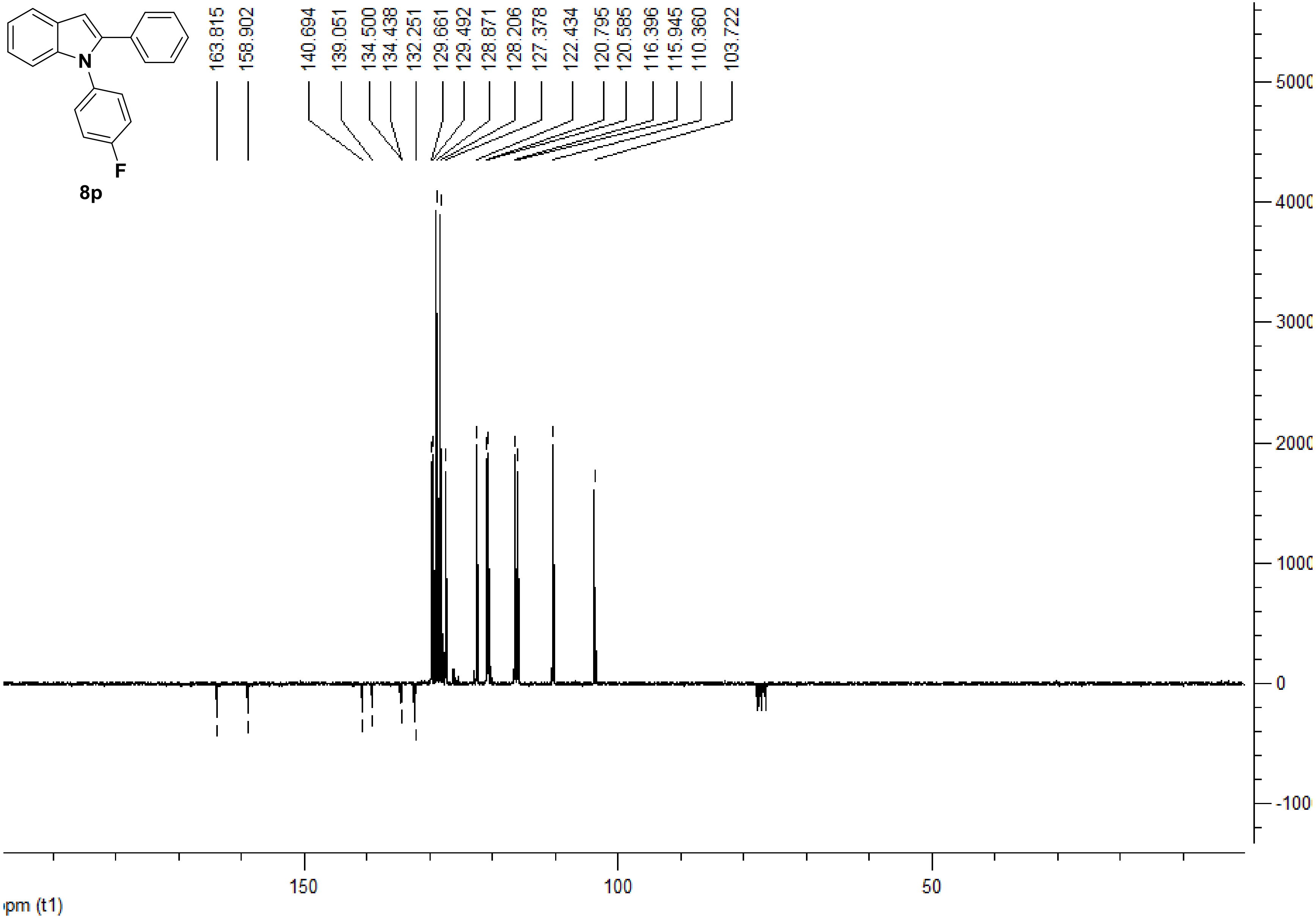
8p
163.815
158.902
140.694
139.051
134.500
134.438
132.251
129.661
129.492
128.871
128.206
127.378
122.434
120.795
120.585
116.396
115.945
110.360
103.722
5000
4000
3000
2000
1000
0
-100
150
100
50
ıpm (t1)

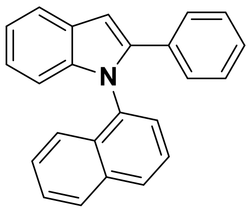

8q

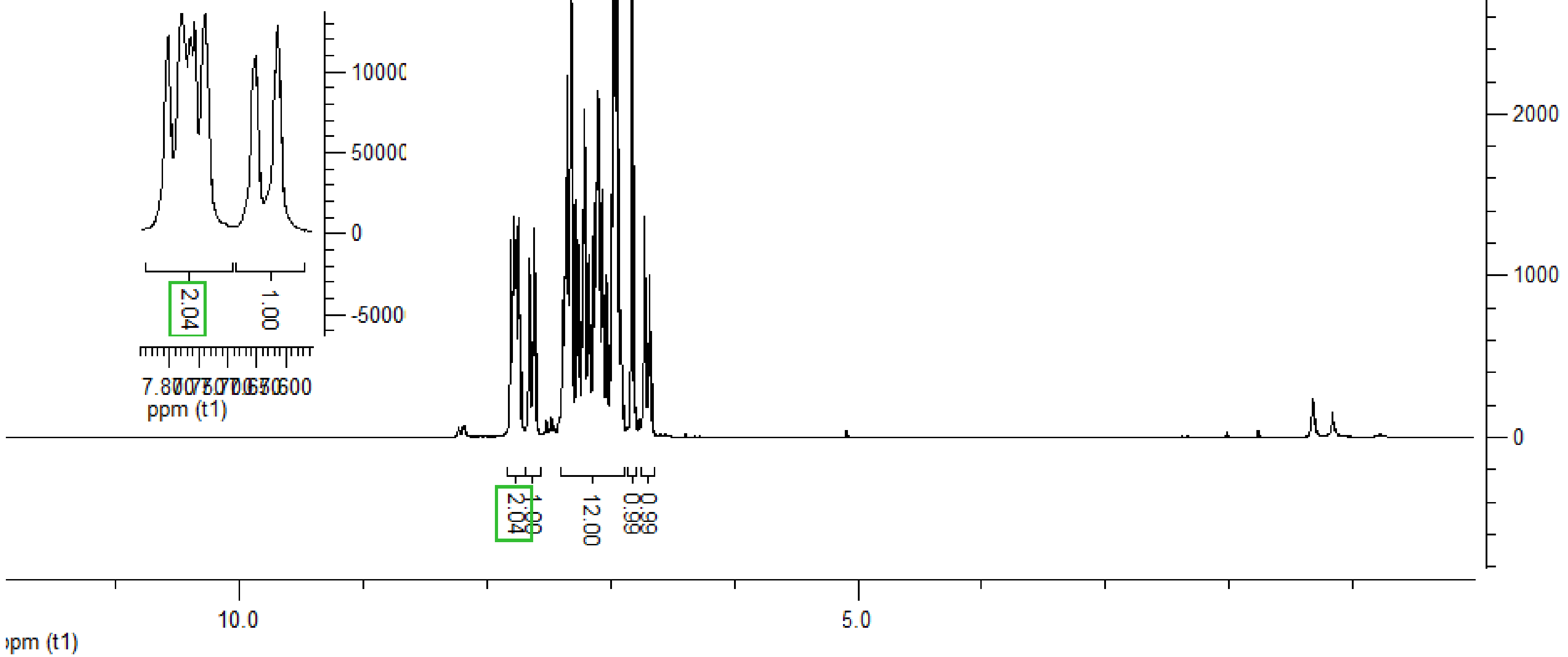

10000
50000
0
-5000
2.04
1.00
7.80 7.75 7.70 7.65 7.60
ppm (t1)
5000
4000
3000
2000
1000
0
2.04
1.00
12.00
0.99
0.99
10.0
5.0
ppm (t1)

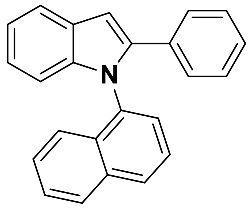

8q

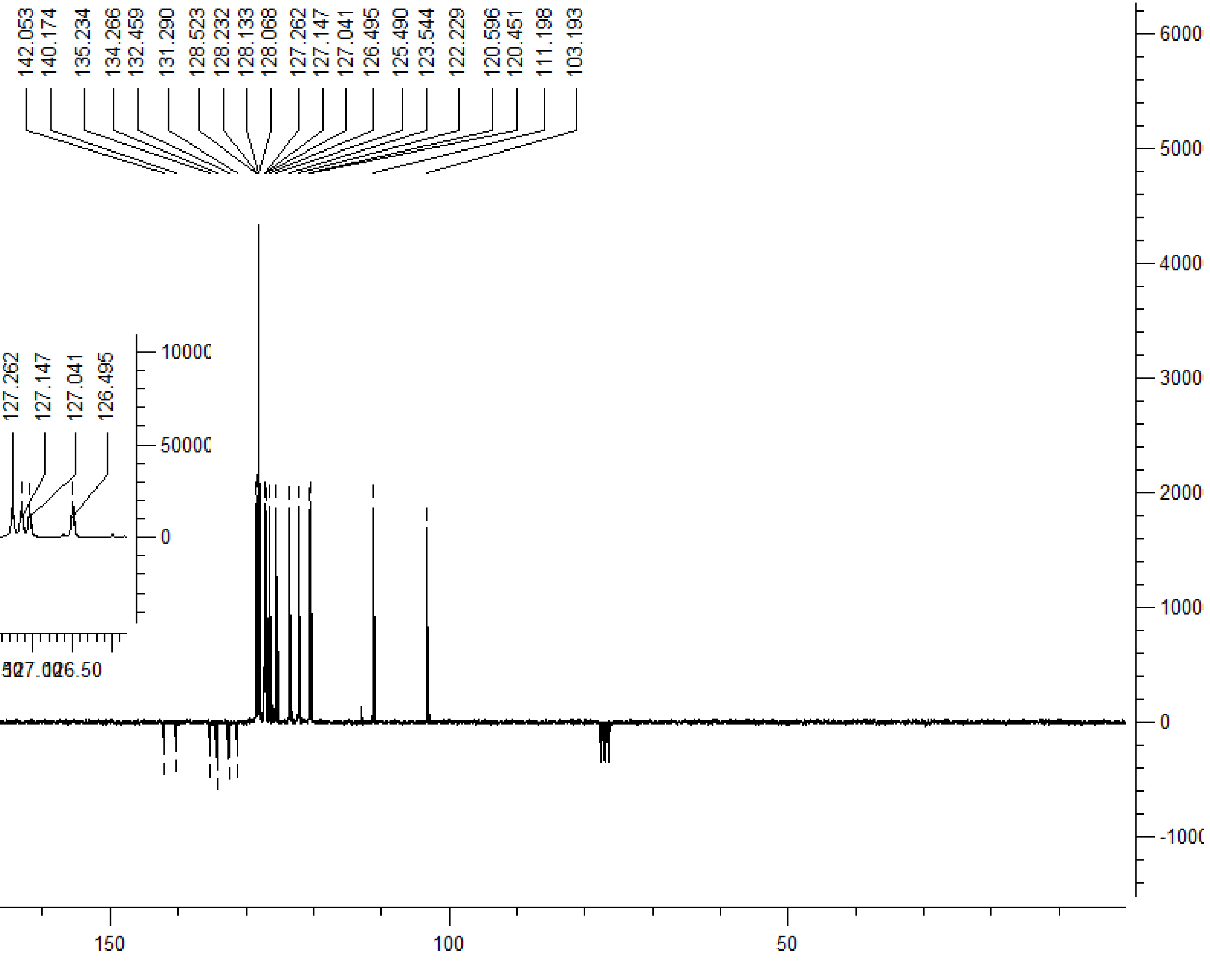
142.053
140.174
135.234
134.266
132.459
131.290
128.523
128.232
128.133
128.068
127.262
127.147
127.041
126.495
125.490
123.544
122.229
120.596
120.451
111.198
103.193
128.523
128.232
128.133
128.068
127.262
127.147
127.041
126.495
ppm (t1)
ppm (t1)
150
100
50

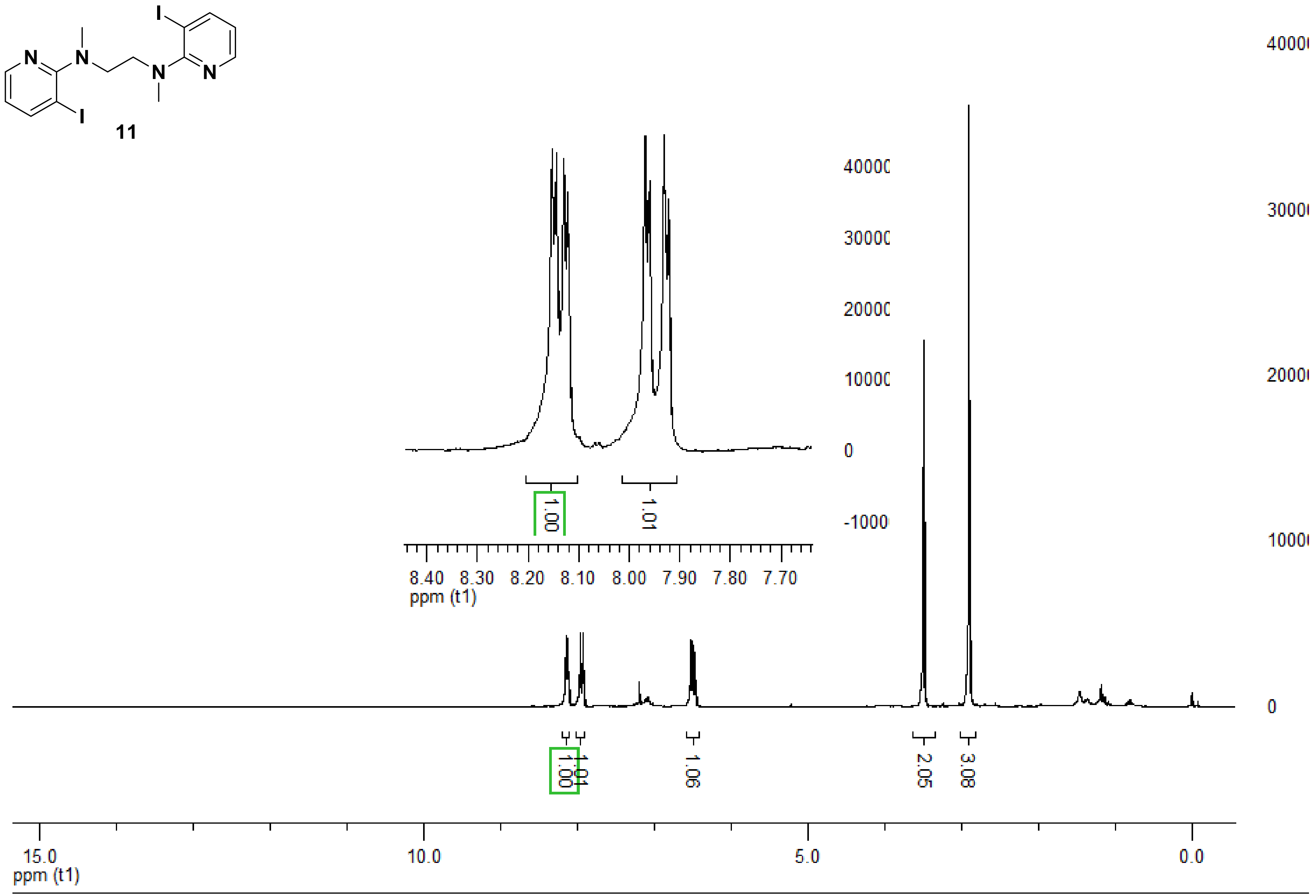

11
40000
30000
20000
10000
0
-1000
1.00
1.01
8.40 8.30 8.20 8.10 8.00 7.90 7.80 7.70
ppm (t1)
1.00
1.01
1.06
2.05
3.08
15.0
10.0
5.0
0.0
ppm (t1)

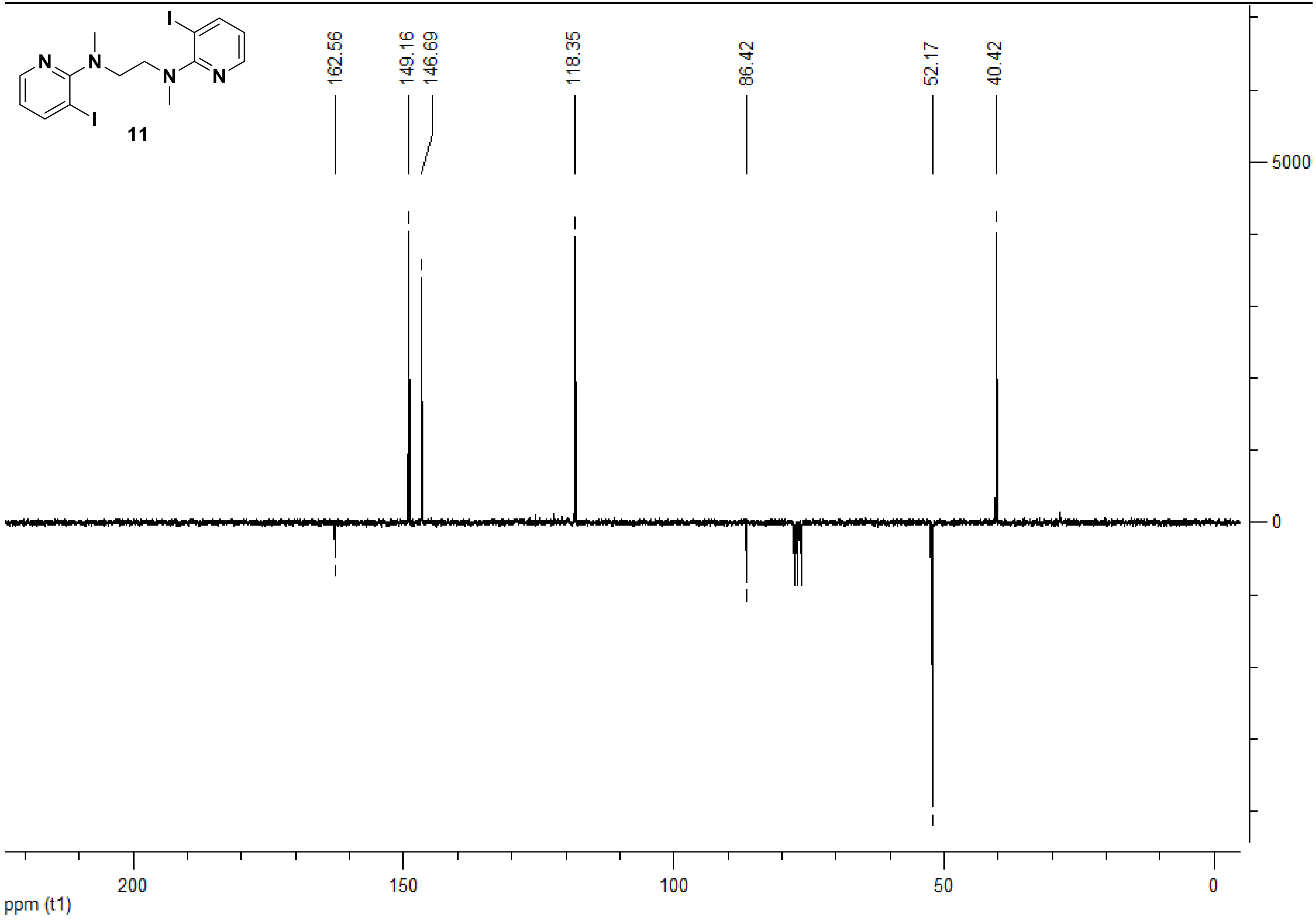

11
162.56
149.16
146.69
118.35
86.42
52.17
40.42
5000
0
200
150
100
50
0
ppm (t1)

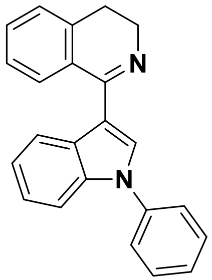

12

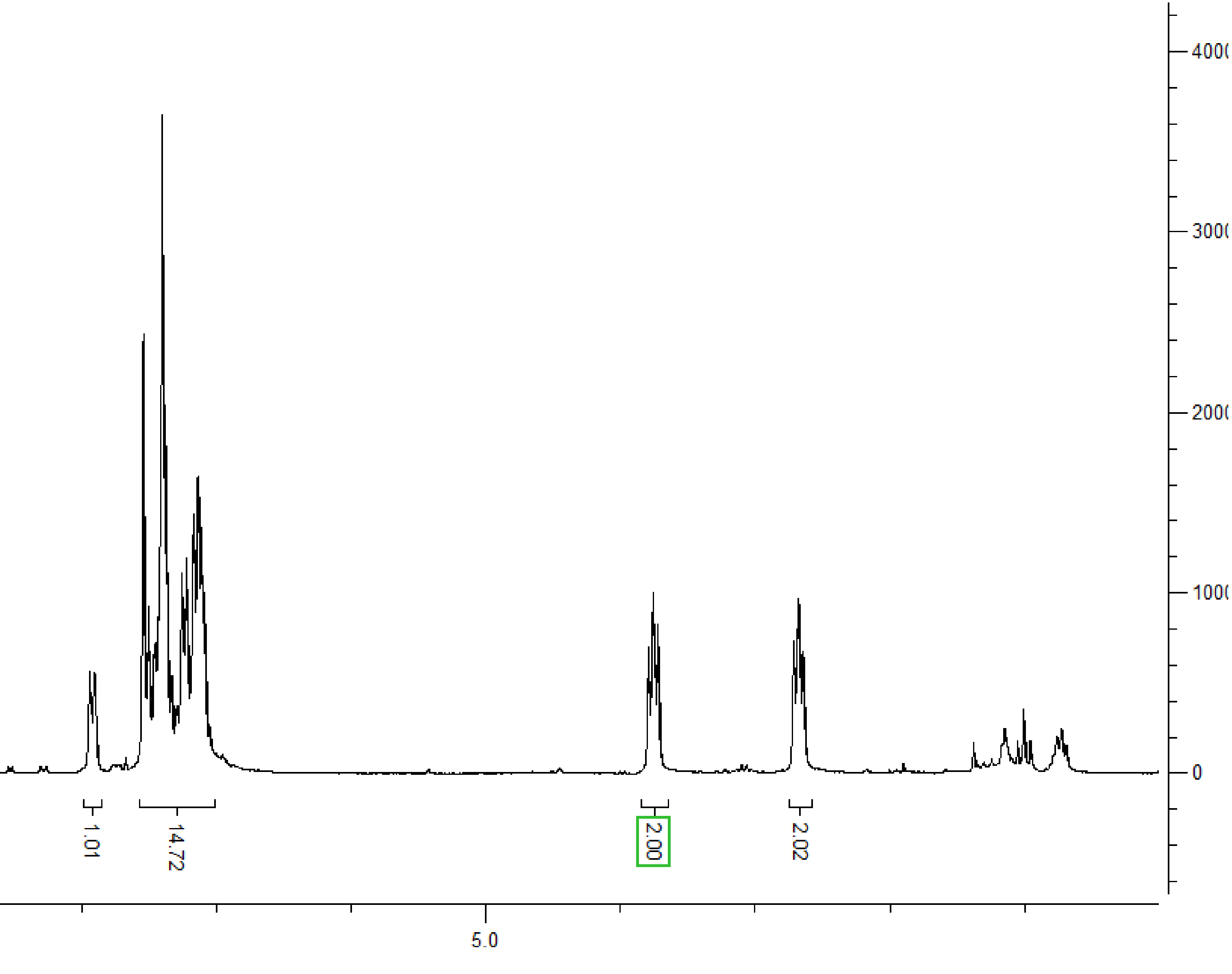

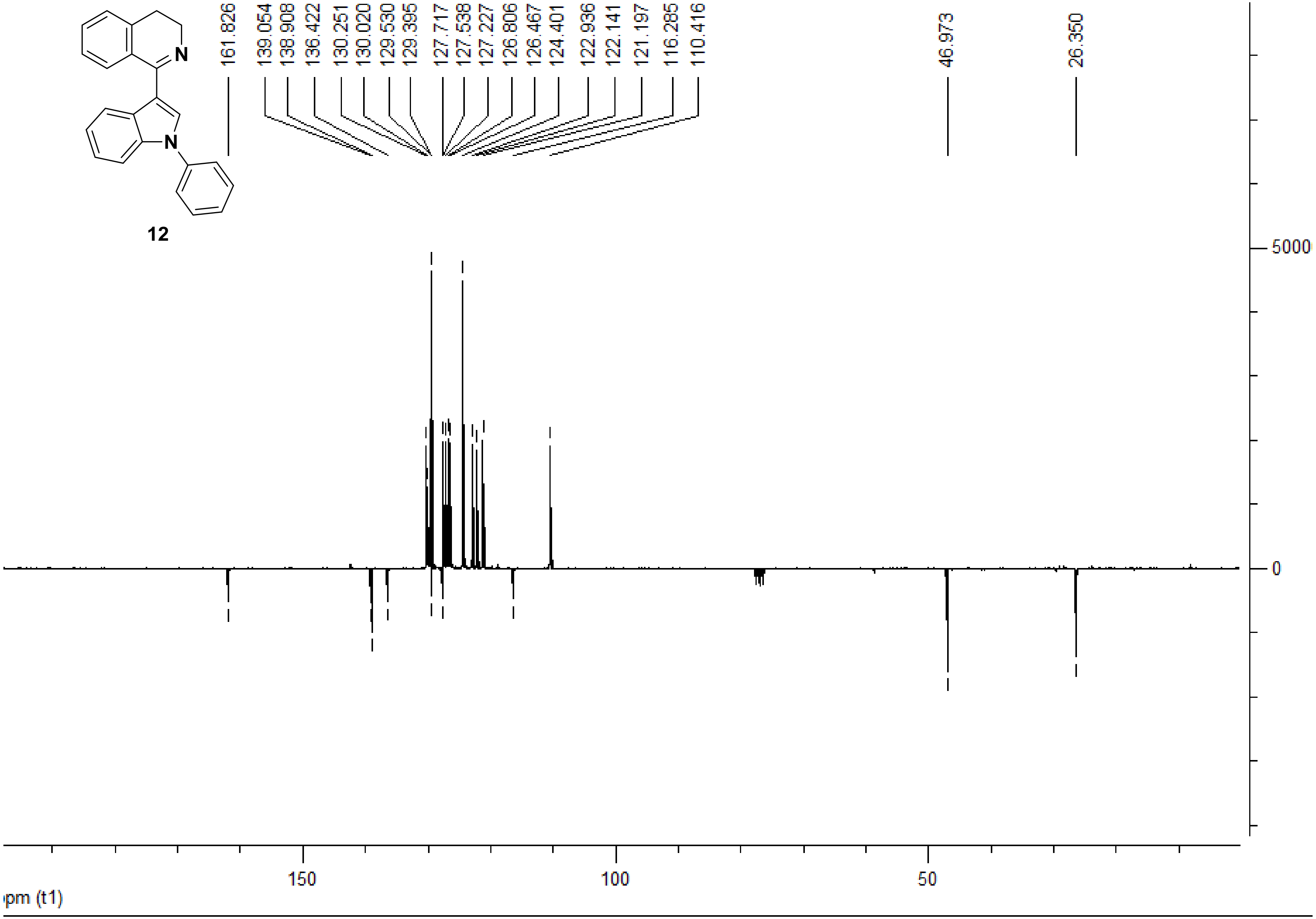
12
161.826
139.054
138.908
136.422
130.251
130.020
129.530
129.395
127.717
127.538
127.227
126.806
126.467
124.401
122.936
122.141
121.197
116.285
110.416
46.973
26.350
5000
0
150
100
50
ppm (t1)

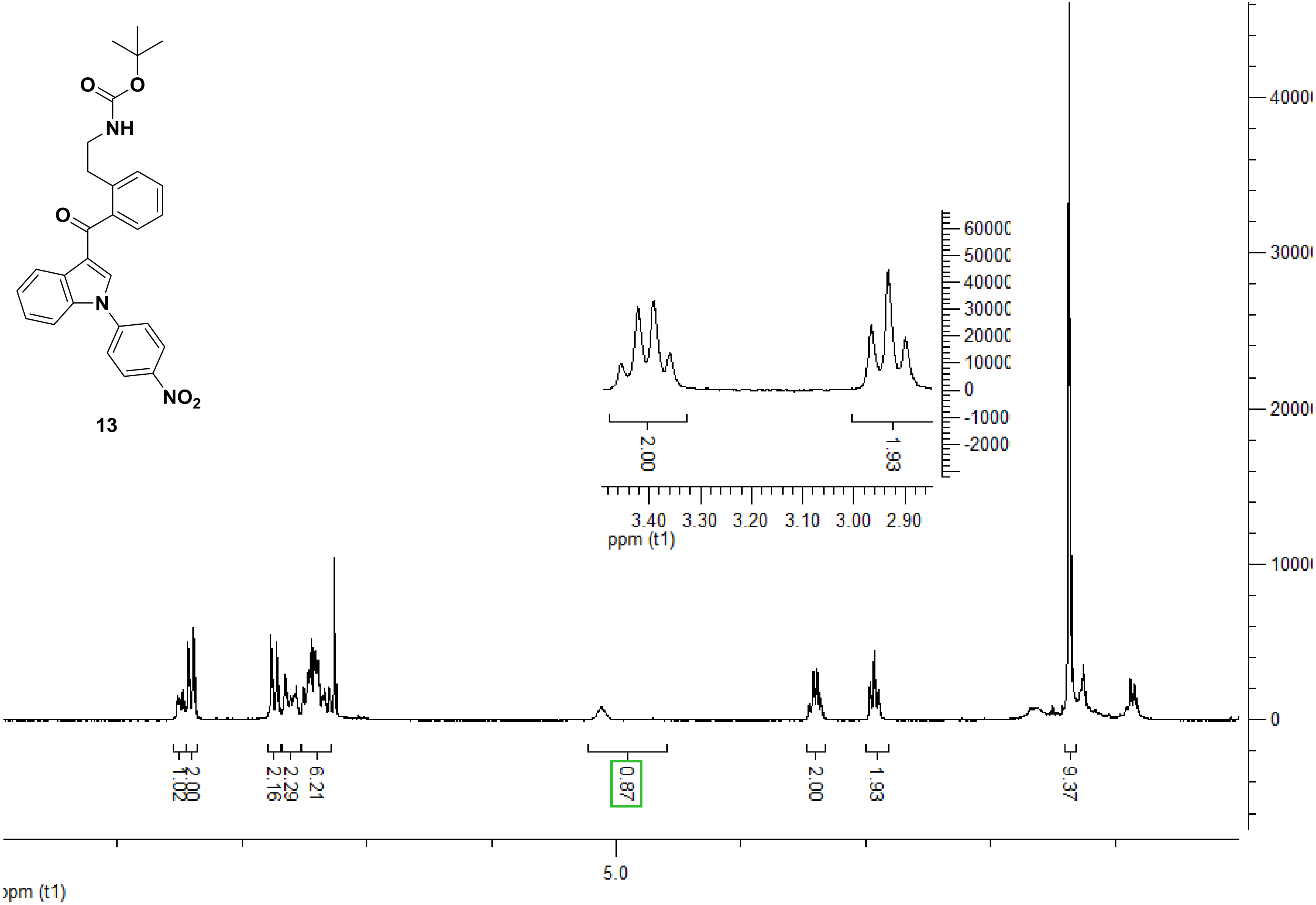
O
O
NH
O
N
NO2
13
2.00
1.93
3.40 3.30 3.20 3.10 3.00 2.90
ppm (t1)
60000
50000
40000
30000
20000
10000
0
-1000
-2000
1.02
2.00
2.16
2.29
6.21
0.87
2.00
1.93
9.37
5.0
ppm (t1)
40000
30000
20000
10000
0

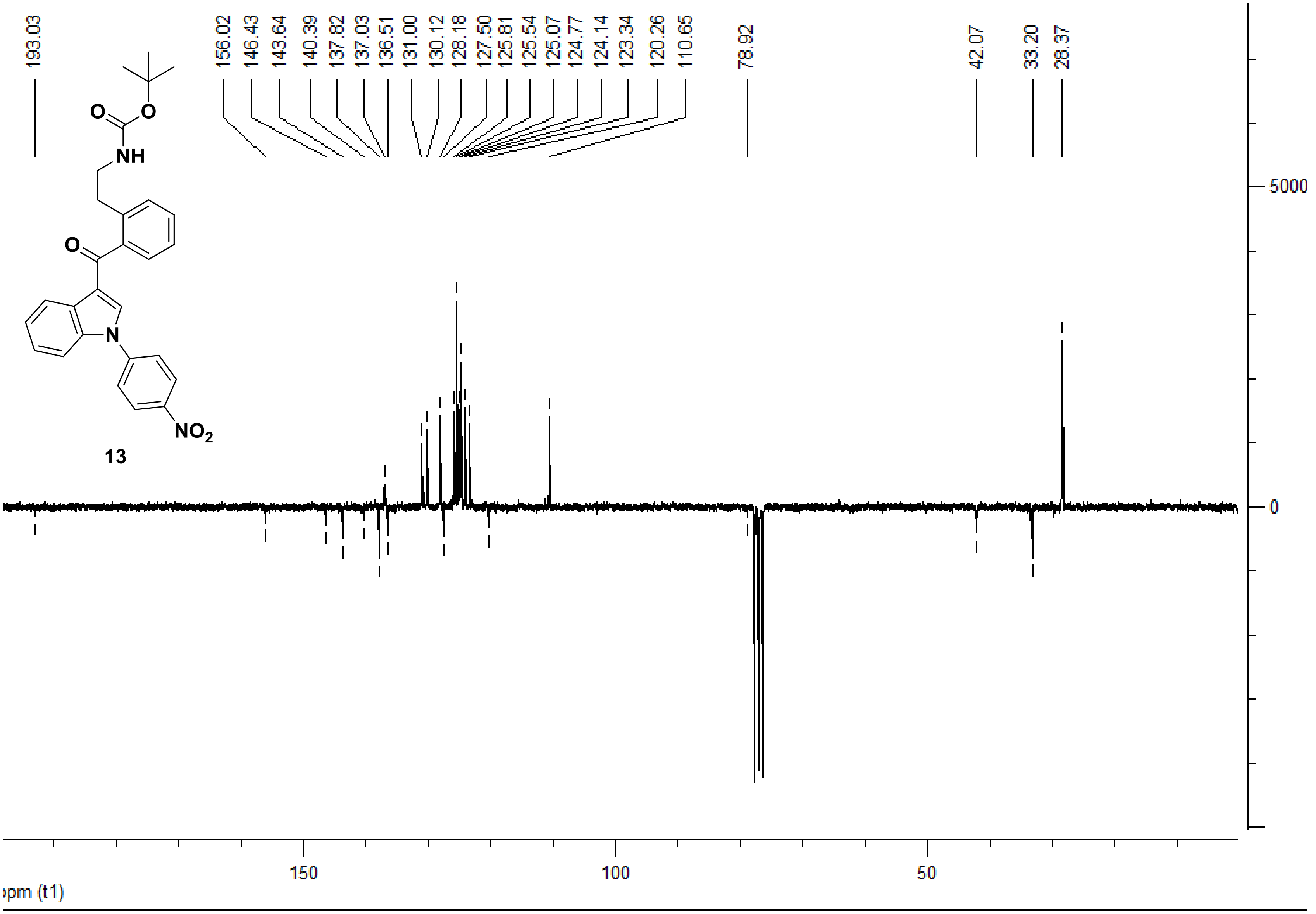
193.03
156.02
146.43
143.64
140.39
137.82
137.03
136.51
131.00
130.12
128.18
127.50
125.81
125.54
125.07
124.77
124.14
123.34
120.26
110.65
78.92
42.07
33.20
28.37
O
O
NH
O
N
NO_2
13
5000
0
150
100
50
ppm (t1)

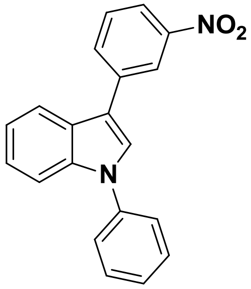
NO2
N

14f

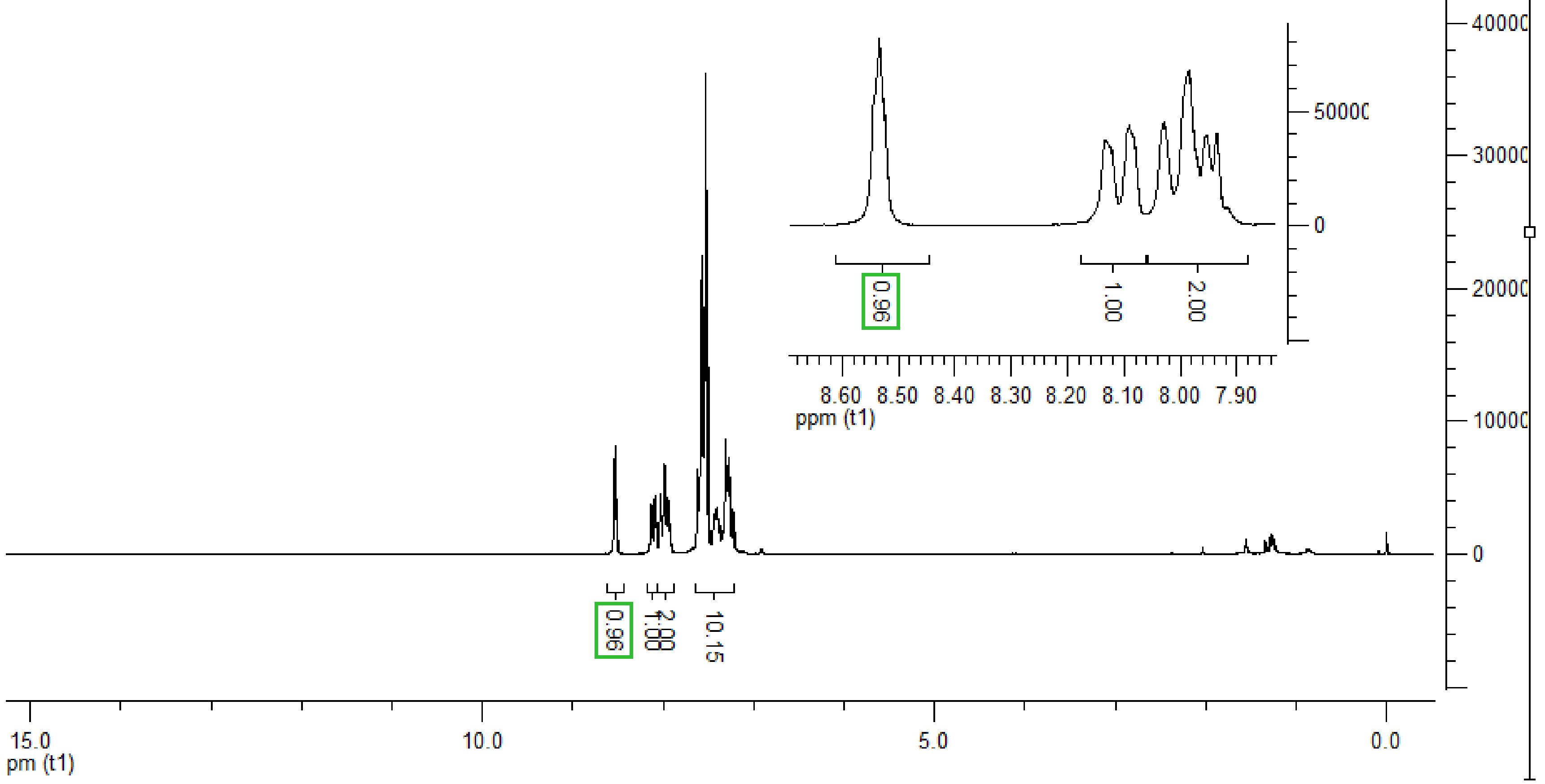
0.96
1.00
2.00
8.60
8.50
8.40
8.30
8.20
8.10
8.00
7.90
ppm (t1)
10.15
15.0
10.0
5.0
0.0
pm (t1)

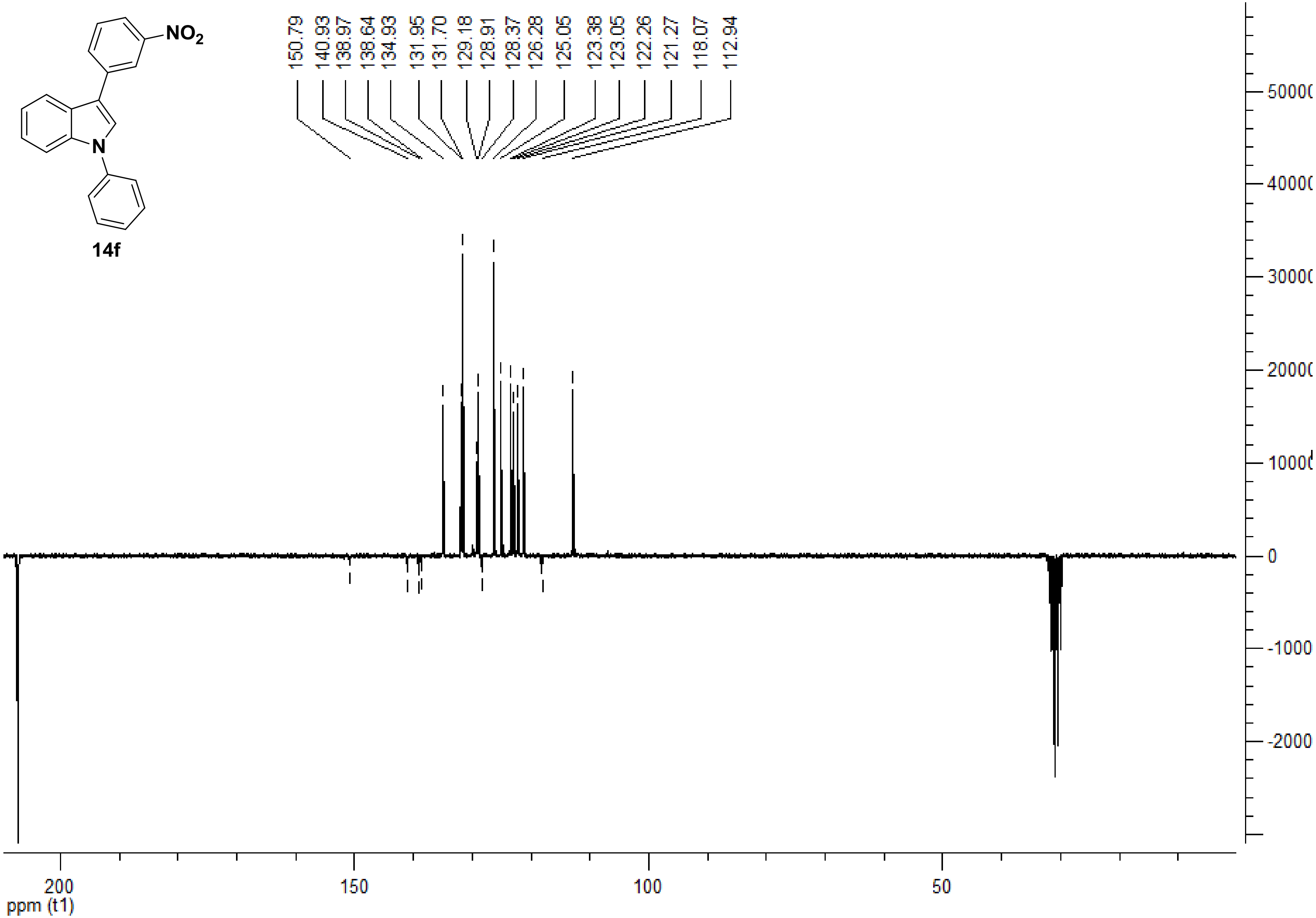

NO2
N
14f
150.79
140.93
138.97
138.64
134.93
131.95
131.70
129.18
128.91
128.37
126.28
125.05
123.38
123.05
122.26
121.27
118.07
112.94
200
150
100
50
ppm (t1)

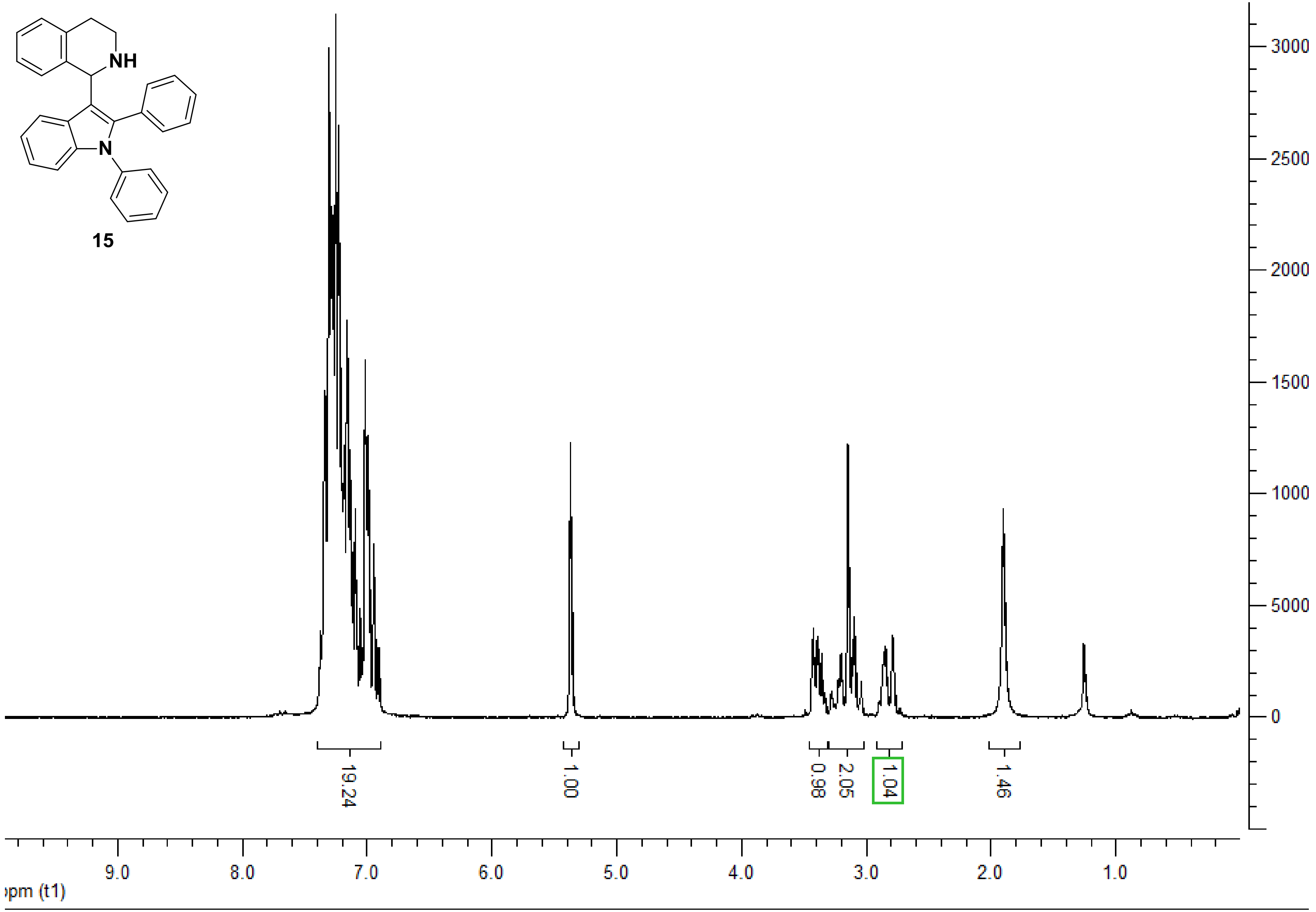
NH
N
15
19.24
1.00
0.98
2.05
1.04
1.46
9.0
8.0
7.0
6.0
5.0
4.0
3.0
2.0
1.0
ppm (t1)
3000
2500
2000
1500
1000
5000
0

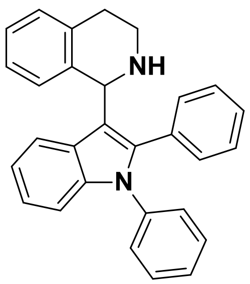

15

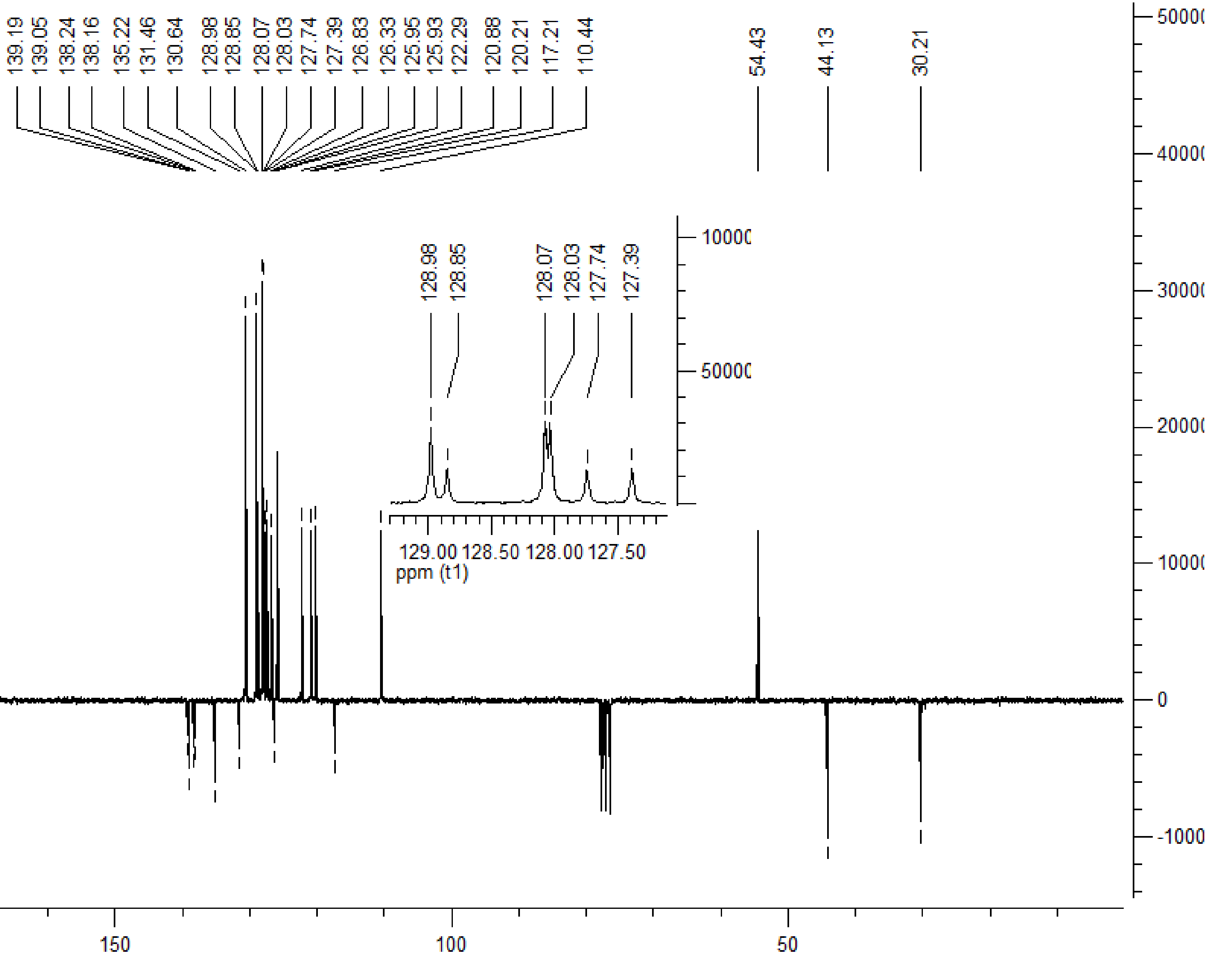

139.19
139.05
138.24
138.16
135.22
131.46
130.64
128.98
128.85
128.07
128.03
127.74
127.39
126.83
126.33
125.95
125.93
122.29
120.88
120.21
117.21
110.44
54.43
44.13
30.21
129.00 128.50 128.00 127.50
ppm (t1)
150
100
50
ppm (t1)
